# Supplementary material for: Outcomes of first emergency admissions for alcohol-related liver disease in England over a 10-year period: retrospective observational cohort study using linked electronic databases
Source: BMJ Open. 2023 Nov 22;13(11):e076955. doi: 10.1136/bmjopen-2023-076955 (PMC10668174; doi:10.1136/bmjopen-2023-076955)
Supplement: Supplementary data [file bmjopen-2023-076955supp001.pdf]

Supplementary Materials & Appendices: BMJ Open

Outcomes of first emergency admissions for alcohol-related liver disease in England over a ten year period: Retrospective observational cohort study using linked electronic databases

Supplementary Figure S1. Kaplan-Meier survival probability of first emergency admissions with a diagnosis of alcohol-related liver disease, stratified by patient-level covariates

Supplementary Table S1. Demographics, clinical characteristics, care processes and outcomes of patients admitted as an emergency for the first time with alcohol-related liver disease between 2008/9 and 2017/18. Stratified by stage of liver disease.

Supplementary Table S2. Demographics, clinical characteristics, care processes and outcomes of patients admitted as an emergency for the first time with alcohol-related liver disease between 2008/9 and 2017/18. Stratified by 5-year period of discharge.

Supplementary Table S3. Demographics, clinical characteristics, care processes and outcomes of patients admitted as an emergency for the first time with alcohol-related liver disease between 2008/9 and 2017/18. Stratified by fiscal year of discharge.

Supplementary Table S4. Demographics and clinical characteristics of those who survived or died following first emergency admission with alcohol-related liver disease between 2008/09 and 2017/18.

Supplementary Figure S2. Sensitivity analyses of main findings exploring alternative covariates for risk-adjustment models

Supplementary Table S5. Demographics, clinical characteristics, care processes and outcomes of patients admitted as an emergency for the first time with alcohol-related liver disease between 2008/9 and 2017/18. Stratified by region of residence.

Supplementary Figure S3. Funnel plot showing regional variation in crude (unadjusted) case fatality rate following first emergency admission for alcohol-related liver disease among people registered with CPRD practices in England, 2008/9 to 2017/18.

Supplementary Table S6. Exploratory Analysis: Association between region of residence and death following first emergency admission for alcohol-related liver disease among people registered with CPRD practices in England, 2008/9 to 2017/18.

Supplementary Appendix 1. Medcodes and corresponding readterms used for primary care records to identify entries relating to alcohol misuse and/or alcohol-related liver disease (ARLD). Codes for ARLD are marked with an asterix (*).

Supplementary Appendix 2. used for screening all diagnoses listed on the death certificate in patients dying during index admission for ARLD.

Supplementary Appendix 3. Higher definition images for time serices (**Figure 3**).

Bodger K, *et al. BMJ Open* 2023; 13:e076955. doi: 10.1136/bmjopen-2023-076955

Bodger *et al*, 2023

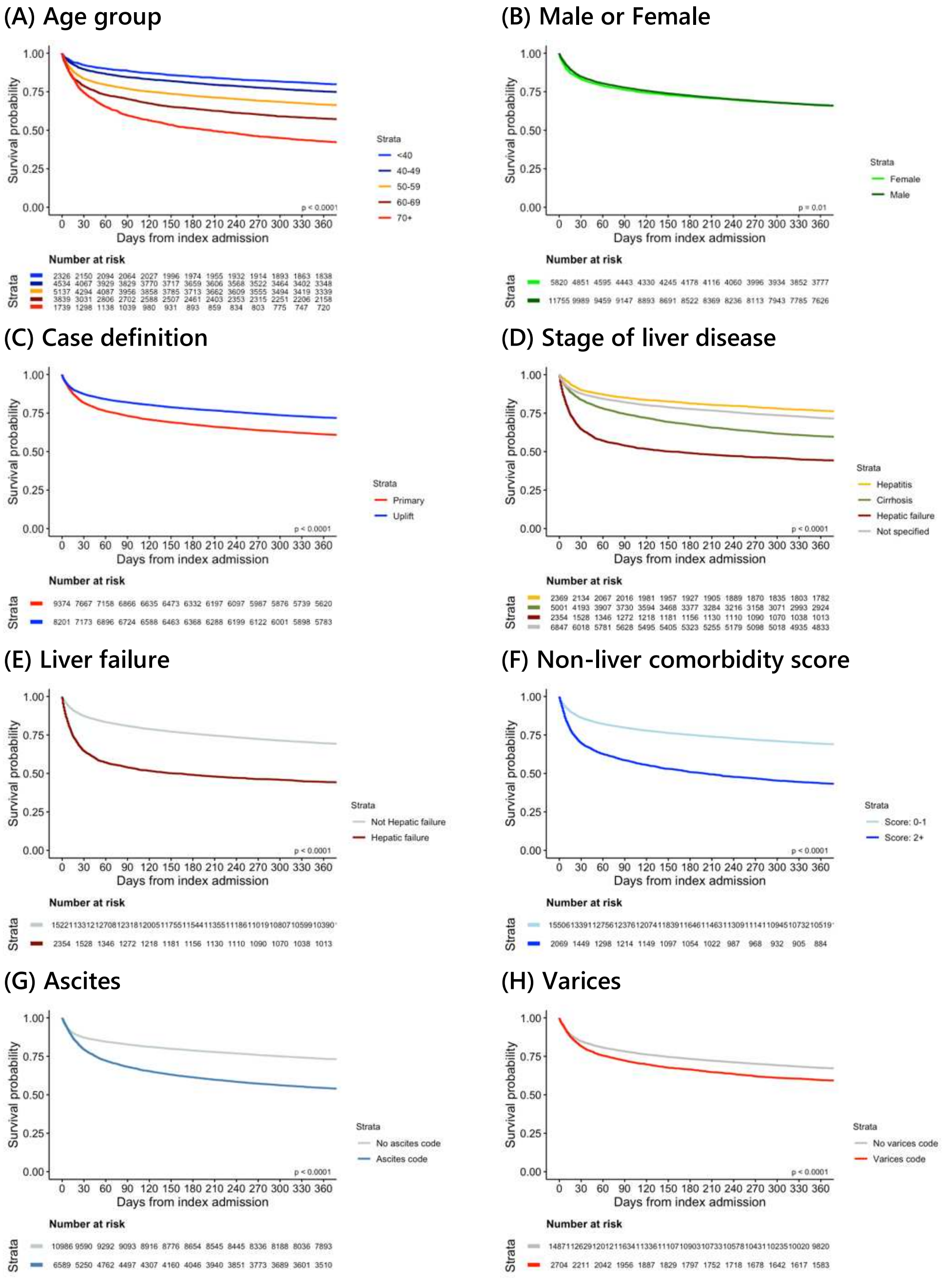

Supplementary Figure S1. Kaplan-Meier survival probability of first emergency admissions with a diagnosis of alcohol-related liver disease, stratified by patient-level covariates. (A) Age group, (B) Sex, (C) Case definition, (D) Stage of liver disease (selected stages only), (E) Liver failure or Not, (F) Non-liver comorbidity score, (G) Presence of codes for ascites, (H) Presence of codes for varices. All p values were significant between strata by log rank test.

Supplementary Table S1. Demographics, clinical characteristics, care processes and outcomes of patients admitted as an emergency for the first time with alcohol-related liver disease between 2008/9 and 2017/18. Patients registered with an English CPRD practice. Stratified by stage of liver disease. Excludes fatty liver (n=942) and fibrosis & sclerosis (n=62).

Characteristic	Hepatitis (n=2,369)	Cirrhosis (n=5,001)	Hepatic failure (n=2,354)	Not specified (n=6,847)	p
Age, mean (sd)	47.7 (12)	56.8 (12)	54.6 (12)	53.0 (12)	**
Sex					
Female	848 (35.8%)	1,562 (31.2%)	839 (35.6%)	2,227 (32.5%)	**
Male	1,521 (64.2%)	3,439 (68.8%)	1,515 (64.4%)	4,620 (67.5%)	
Deprivation quintile					
1 - Least deprived	332 (14.0%)	769 (15.4%)	307 (13.0%)	883 (12.9%)	**
2	337 (14.2%)	831 (16.6%)	357 (15.2%)	1,054 (15.4%)	
3	404 (17.1%)	882 (17.6%)	404 (17.2%)	1,176 (17.2%)	
4	560 (23.6%)	1,084 (21.7%)	533 (22.6%)	1,437 (21.0%)	
5 - Most deprived	736 (31.1%)	1,435 (28.7%)	753 (32.0%)	2,297 (33.5%)	
Case definition					
Primary	1,729 (73.0%)	2,759 (55.2%)	1,982 (84.2%)	2,694 (39.3%)	**
Uplift	640 (27.0%)	2,242 (44.8%)	372 (15.8%)	4,153 (60.7%)	
Ascites	531 (22.4%)	2,472 (49.4%)	1,383 (58.8%)	2,109 (30.8%)	**
Varices	154 (6.5%)	1,261 (25.2%)	415 (17.6%)	845 (12.3%)	**
Comorbidity score (non-liver)					
0-1	2,255 (95.2%)	4,150 (83.0%)	2,040 (86.7%)	6,132 (89.6%)	**
2+	114 (4.8%)	851 (17.0%)	314 (13.3%)	715 (10.4%)	
Five-year period of discharge					
2008/9 – 2012/13	981 (41.4%)	1,894 (37.9%)	815 (34.6%)	3,464 (50.6%)	**
2013/14 – 2017/18	1,388 (58.6%)	3,107 (62.1%)	1,539 (65.4%)	3,383 (49.4%)	
GP contact in last year	1,268 (53.5%)	2,921 (58.4%)	1,336 (56.8%)	3,887 (56.8%)	**
With alcohol codes recorded §	661 (52.1%)	1302 (44.6%)	619 (46.3%)	1849 (47.6%)	**
With liver codes recorded §	94 (7.4%)	593 (20.3%)	228 (17.1%)	514 (13.2%)	*ns*
Emergency admission in last year	918 (38.8%)	2,011 (40.2%)	832 (35.3%)	3,029 (44.2%)	**
With alcohol codes recorded ¶	771 (84.0%)	1,407 (70.0%)	593 (71.3%)	2,352 (77.7%)	**
Length of stay, mean (sd)	12 (15)	12 (16)	16 (18)	10 (13)	**
Higher level care during admission	138 (5.8%)	508 (10.2%)	496 (21.1%)	556 (8.1%)	**
Died in-hospital	221 (9.3%)	781 (15.6%)	912 (38.7%)	761 (11.1%)	**
Died within 365 days of admission	557 (23.5%)	2,003 (40.1%)	1,309 (55.6%)	1,931 (28.2%)	**

* p<0.05; **p<0.001 between groups; ns = not significant; na = not applicable; § = percentages based on those with a GP contact within the last year; ¶ = percentages based on those with an emergency admission within the last year.

Supplementary Table S2. Demographics, clinical characteristics, care processes and outcomes of patients admitted as an emergency for the first time with alcohol-related liver disease between 2008/9 and 2017/18. Patients registered with an English CPRD practice. Stratified by 5-year period of discharge.

Characteristic	Total	5 Year Period of Discharge		p
	08/09 to 17/18 (n=17,575)	08/09 to 12/13 (n=7,566)	13/14 to 17/18 (n=10,009)	
Age, *mean (sd)*	53.4 (12)	52.5 (12)	54.1 (12)	**
Sex				
Female	5,820 (33.1%)	2,477 (33%)	3,343 (33%)	*ns*
Male	11,755 (66.9%)	5,089 (67%)	6,666 (67%)	
Deprivation quintile				
1 - Least deprived	2,435 (13.9%)	977 (13%)	1,458 (15%)	**
2	2,735 (15.6%)	1,162 (15%)	1,573 (16%)	
3	3,028 (17.2%)	1,223 (16%)	1,805 (18%)	
4	3,837 (21.8%)	1,674 (22%)	2,163 (22%)	
5 - Most deprived	5,540 (31.5%)	2,530 (33%)	3,010 (30%)	
Case definition				
Primary	7,595 (51.1%)	4,102 (54%)	5,272 (53%)	*
Uplift	7,272 (48.9%)	3,464 (46%)	4,737 (47%)	
Stage of liver disease				
Fatty liver	942 (5.4%)	385 (5.1%)	557 (5.6%)	**
Hepatitis	2,369 (13.5%)	981 (13%)	1,388 (14%)	
Fibrosis and sclerosis	62 (0.4%)	27 (0.4%)	35 (0.3%)	
Cirrhosis	5,001 (28.5%)	1,894 (25%)	3,107 (31%)	
Hepatic failure	2,354 (13.4%)	815 (11%)	1,539 (15%)	
Not specified	6,847 (39.0%)	3,464 (46%)	3,383 (34%)	
Ascites	6,589 (37.5%)	2,799 (37%)	3,790 (38%)	*ns*
Varices	2,704 (15.4%)	1,113 (15%)	1,591 (16%)	*
Comorbidity score (non-liver)				
0-1	15,506 (88.2%)	6,773 (90%)	8,733 (87%)	**
2+	2,069 (11.8%)	793 (10%)	1,276 (13%)	
GP contact in last year	9,936 (56.5%)	4,513 (59.6%)	5,423 (54.2%)	**
With alcohol codes recorded §	4702 (47.3%)	2,173 (48.1%)	2,529 (46.6%)	*ns*
With liver codes recorded §	1465 (14.7%)	724 (16.0%)	741 (13.7%)	**
Emergency admission in last year	7,265 (41.3%)	3,127 (41%)	4,138 (41%)	*ns*
With alcohol codes recorded ¶	5,525 (76.0%)	2,398 (76.7%)	3,127 (75.6%)	*ns*
Length of stay, mean (sd)	12 (15)	12 (15)	11 (14)	**
Higher level care during admission	1,750 (10.0%)	652 (8.6%)	1,098 (11%)	**
Died in-hospital	2,708 (15.4%)	1,222 (16%)	1,486 (15%)	*
Died within 365 days of admission	5,938 (33.8%)	2,614 (35%)	3,324 (33%)	*ns*

* p<0.05; **p<0.001 between time periods; ns = not significant; § = percentages based on those with a GP contact within the last year; ¶ = percentages based on those with an emergency admission within the last year.

Supplementary Table S3. Demographics, clinical characteristics, care processes and outcomes of patients admitted as an emergency for the first time with alcohol-related liver disease between 2008/9 and 2017/18. Patients registered with an English CPRD practice. Stratified by fiscal year of discharge.

**Characteristic**	**2008/09**	**2009/10**	**2010/11**	**2011/12**	**2012/13**	**2013/14**	**2014/15**	**2015/16**	**2016/17**	**2017/18**
	N = 1,494	N = 1,512	N = 1,645	N = 1,626	N = 1,692	N = 1,741	N = 1,771	N = 1,992	N = 2,044	N = 2,058
**Age**, mean (sd)	52 (12)	52 (12)	52 (12)	53 (13)	53 (12)	54 (12)	54 (12)	54 (12)	54 (12)	55 (13)
**Age group**										
<40	220 (14.7%)	232 (15.3%)	257 (15.6%)	244 (15.0%)	236 (13.9%)	209 (12.0%)	225 (12.7%)	228 (11.4%)	236 (11.5%)	239 (11.6%)
40-49	429 (28.7%)	419 (27.7%)	439 (26.7%)	431 (26.5%)	451 (26.7%)	440 (25.3%)	444 (25.1%)	486 (24.4%)	518 (25.3%)	477 (23.2%)
50-59	423 (28.3%)	403 (26.7%)	491 (29.8%)	433 (26.6%)	490 (29.0%)	542 (31.1%)	536 (30.3%)	580 (29.1%)	629 (30.8%)	610 (29.6%)
60-69	293 (19.6%)	337 (22.3%)	326 (19.8%)	352 (21.6%)	361 (21.3%)	390 (22.4%)	382 (21.6%)	486 (24.4%)	451 (22.1%)	461 (22.4%)
70+	129 (8.6%)	121 (8.0%)	132 (8.0%)	166 (10.2%)	154 (9.1%)	160 (9.2%)	184 (10.4%)	212 (10.6%)	210 (10.3%)	271 (13.2%)
**Sex**										
Female	505 (33.8%)	488 (32.3%)	527 (32.0%)	526 (32.3%)	559 (33.0%)	533 (30.6%)	607 (34.3%)	669 (33.6%)	697 (34.1%)	709 (34.5%)
Male	989 (66.2%)	1,024 (67.7%)	1,118 (68.0%)	1,100 (67.7%)	1,133 (67.0%)	1,208 (69.4%)	1,164 (65.7%)	1,323 (66.4%)	1,347 (65.9%)	1,349 (65.5%)
**Deprivation quintile**										
1 - Least deprived	174 (11.6%)	209 (13.8%)	201 (12.2%)	207 (12.7%)	243 (14.4%)	253 (14.5%)	240 (13.6%)	287 (14.4%)	320 (15.7%)	301 (14.6%)
2	236 (15.8%)	222 (14.7%)	243 (14.8%)	250 (15.4%)	264 (15.6%)	274 (15.7%)	267 (15.1%)	331 (16.6%)	289 (14.1%)	359 (17.4%)
3	264 (17.7%)	219 (14.5%)	282 (17.1%)	259 (15.9%)	264 (15.6%)	298 (17.1%)	336 (19.0%)	362 (18.2%)	376 (18.4%)	368 (17.9%)
4	334 (22.4%)	339 (22.4%)	361 (21.9%)	344 (21.2%)	398 (23.5%)	369 (21.2%)	365 (20.6%)	424 (21.3%)	438 (21.4%)	465 (22.6%)
5 - Most deprived	486 (32.5%)	523 (34.6%)	558 (33.9%)	566 (34.8%)	523 (30.9%)	547 (31.4%)	563 (31.8%)	588 (29.5%)	621 (30.4%)	565 (27.5%)
**Case definition**										
Primary	843 (56.4%)	819 (54.2%)	878 (53.4%)	870 (53.5%)	906 (53.5%)	897 (51.5%)	959 (54.2%)	1,054 (52.9%)	1,092 (53.4%)	1,056 (51.3%)
Uplift	651 (43.6%)	693 (45.8%)	767 (46.6%)	756 (46.5%)	786 (46.5%)	844 (48.5%)	812 (45.8%)	938 (47.1%)	952 (46.6%)	1,002 (48.7%)
**Stage of liver disease**										
Fatty liver	59 (3.9%)	82 (5.4%)	94 (5.7%)	75 (4.6%)	95 (5.6%)	103 (5.9%)	98 (5.5%)	113 (5.7%)	116 (5.7%)	107 (5.2%)
Hepatitis	180 (12.0%)	200 (13.2%)	221 (13.4%)	203 (12.5%)	230 (13.6%)	254 (14.6%)	250 (14.1%)	264 (13.3%)	295 (14.4%)	272 (13.2%)
Fibrosis and sclerosis	7 (0.5%)	3 (0.2%)	6 (0.4%)	6 (0.4%)	6 (0.4%)	5 (0.3%)	10 (0.6%)	5 (0.3%)	6 (0.3%)	8 (0.4%)
Cirrhosis	323 (21.6%)	341 (22.6%)	413 (25.1%)	441 (27.1%)	480 (28.4%)	518 (29.8%)	553 (31.2%)	631 (31.7%)	650 (31.8%)	651 (31.6%)
Hepatic failure	130 (8.7%)	156 (10.3%)	161 (9.8%)	199 (12.2%)	226 (13.4%)	203 (11.7%)	261 (14.7%)	309 (15.5%)	358 (17.5%)	351 (17.1%)
Not specified	795 (53.2%)	730 (48.3%)	750 (45.6%)	702 (43.2%)	655 (38.7%)	658 (37.8%)	599 (33.8%)	670 (33.6%)	619 (30.3%)	669 (32.5%)
**Ascites**	219 (14.7%)	205 (13.6%)	234 (14.2%)	263 (16.2%)	250 (14.8%)	294 (16.9%)	329 (18.6%)	305 (15.3%)	307 (15.0%)	298 (14.5%)
**Varices**	14 (0.9%)	16 (1.1%)	20 (1.2%)	27 (1.7%)	26 (1.5%)	36 (2.1%)	26 (1.5%)	31 (1.6%)	33 (1.6%)	29 (1.4%)
**Comorbidity Score (non-liver)**										
0-1	1,341 (89.8%)	1,357 (89.7%)	1,469 (89.3%)	1,456 (89.5%)	1,505 (88.9%)	1,515 (87.0%)	1,538 (86.8%)	1,746 (87.7%)	1,792 (87.7%)	1,787 (86.8%)
2+	153 (10.2%)	155 (10.3%)	176 (10.7%)	170 (10.5%)	187 (11.1%)	226 (13.0%)	233 (13.2%)	246 (12.3%)	252 (12.3%)	271 (13.2%)
**GP contact in last year**	940 (62.9%)	922 (61.0%)	967 (58.8%)	966 (59.4%)	950 (56.1%)	983 (56.5%)	959 (54.2%)	1,113 (55.9%)	1,078 (52.7%)	1,058 (51.4%)
With alcohol codes recorded §	433 (46.1%)	439 (47.6%)	486 (50.3%)	460 (47.6%)	465 (48.9%)	505 (51.4%)	449 (46.8%)	527 (47.3%)	480 (44.5%)	458 (43.3%)
With liver codes recorded §	164 (17.4%)	161 (17.5%)	164 (17.0%)	138 (14.3%)	137 (14.4%)	163 (16.6%)	129 (13.5%)	155 (13.9%)	143 (13.3%)	111 (10.5%)
**Emergency admission in last year**	583 (39.0%)	665 (44.0%)	689 (41.9%)	662 (40.7%)	702 (41.5%)	718 (41.2%)	740 (41.8%)	821 (41.2%)	837 (40.9%)	848 (41.2%)
With alcohol codes ¶	443 (76.0%)	506 (76.1%)	541 (78.5%)	502 (75.8%)	538 (76.6%)	556 (77.4%)	543 (73.4%)	616 (75.0%)	631 (75.4%)	649 (76.5%)
**Length of stay**, mean (sd)	13 (16)	13 (17)	12 (15)	11 (15)	12 (15)	12 (15)	12 (15)	11 (14)	11 (14)	11 (14)
**Higher level care during admission**	92 (6.2%)	149 (9.9%)	157 (9.5%)	146 (9.0%)	149 (8.8%)	192 (11.0%)	211 (11.9%)	198 (9.9%)	224 (11.0%)	232 (11.3%)
**Died in-hospital**	274 (18.3%)	267 (17.7%)	257 (15.6%)	237 (14.6%)	258 (15.2%)	259 (14.9%)	261 (14.7%)	270 (13.6%)	294 (14.4%)	331 (16.1%)
**Died with 365 days of admission**	550 (36.8%)	556 (36.8%)	532 (32.3%)	548 (33.7%)	574 (33.9%)	582 (33.4%)	624 (35.2%)	649 (32.6%)	661 (32.3%)	662 (32.2%)

§ = percentages based on those with a GP contact within the last year; ¶ = percentages based on those with an emergency admission within the last year.

Supplementary Table S4. Demographics and clinical characteristics of those who survived or died following first emergency admission with alcohol-related liver disease between 2008/09 and 2017/18. Data are shown for in-hospital deaths and cumulative deaths at 365 days from admission.

Characteristic	In-hospital			Within 365 days		
	Survived N=14,867	Died N=2,708	p	Survived N=11,637	Died N=5,939	p
Age, mean (sd)	52.6 (12)	58.1 (12)	**	51.4 (12)	57.5 (12)	**
Age Group						
<40	2,153 (14.5%)	173 (6.4%)	**	1,862 (16.0%)	464 (7.8%)	**
40-49	4,071 (27.4%)	463 (17.1%)		3,405 (29.3%)	1,129 (19.0%)	
50-59	4,318 (29.0%)	819 (30.2%)		3,421 (29.4%)	1,716 (28.9%)	
60-69	3,018 (20.3%)	821 (30.3%)		2,207 (19.0%)	1,632 (27.5%)	
70+	1,307 (8.8%)	432 (16.0%)		742 (6.4%)	997 (16.8%)	
Sex						
Female	4,844 (32.6%)	976 (36.0%)	**	3,857 (33.1%)	1,963 (33.1%)	ns
Male	10,023 (67.4%)	1,732 (64.0%)		7,780 (66.9%)	3,975 (66.9%)	
Deprivation quintile						
1 - Least deprived	2,040 (13.7%)	395 (14.6%)	*	1,544 (13.3%)	891 (15.0%)	**
2	2,289 (15.4%)	446 (16.5%)		1,767 (15.2%)	968 (16.3%)	
3	2,530 (17.0%)	498 (18.4%)		1,954 (16.8%)	1,074 (18.1%)	
4	3,251 (21.9%)	586 (21.6%)		2,582 (22.2%)	1,255 (21.1%)	
5 - Most deprived	4,757 (32.0%)	783 (28.9%)		3,790 (32.6%)	1,750 (29.5%)	
Case definition						
Primary	7,595 (51.1%)	1,779 (65.7%)	**	5,732 (49.3%)	3,642 (61.3%)	**
Uplift	7,272 (48.9%)	929 (34.3%)		5,905 (50.7%)	2,296 (38.7%)	
Stage of liver disease						
Fatty liver	916 (6.2%)	26 (1.0%)	**	818 (7.0%)	124 (2.1%)	**
Hepatitis	2,148 (14.4%)	221 (8.2%)		1,812 (15.6%)	557 (9.4%)	
Fibrosis and sclerosis	55 (0.4%)	7 (0.3%)		48 (0.4%)	14 (0.2%)	
Cirrhosis	4,220 (28.4%)	781 (28.8%)		2,998 (25.8%)	2,003 (33.7%)	
Hepatic failure	1,442 (9.7%)	912 (33.7%)		1,045 (9.0%)	1,309 (22.0%)	
Not specified	6,086 (40.9%)	761 (28.1%)		4,916 (42.2%)	1,931 (32.5%)	
Ascites	5,179 (34.8%)	1,410 (52.1%)	**	3,580 (30.8%)	3,009 (50.7%)	**
Varices	2,191 (14.7%)	513 (18.9%)	*	1,609 (13.8%)	1,095 (18.4%)	**
Comorbidity score (non-liver)						
0-1	13,368 (89.9%)	2,138 (79.0%)	**	10,736 (92.3%)	4,770 (80.3%)	**
2+	1,499 (10.1%)	570 (21.0%)		901 (7.7%)	1,168 (19.7%)	
Length of stay, mean (sd)	11 (15)	14 (16)	**	11 (14)	13 (16)	**
Higher level care	773 (5.2%)	977 (36.1%)	**	601 (5.2%)	1,149 (19.3%)	**

* p<0.05; **p<0.001 between deaths and survivors; ns= not statistically significant.

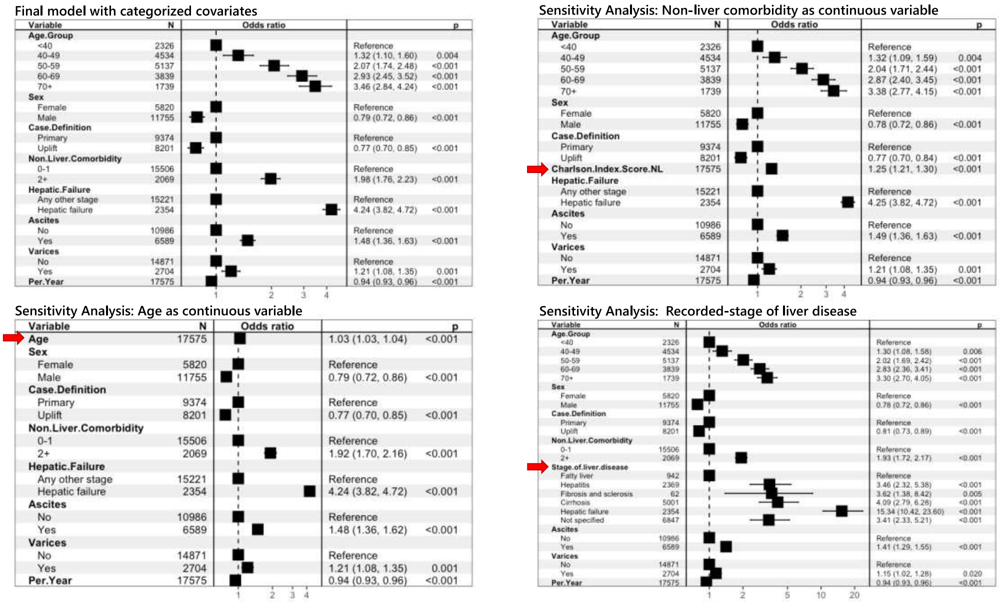

Supplementary Figure S2. Sensitivity analyses using alternative covariates for risk-adjustment models. Four alternative models are shown examining factors associated with in-hospital death. The final parsimonious model (upper left) included binary or categorized variables and demonstrates a year-on-year improvement in prognosis (OR 0.94 "per year" through the 10-year observation period). Substituting continuous variables for age (bottom left) or comorbidity score (top right) did not alter the findings (OR 0.94). Similarly, replacing the dichotomous "Hepatic Failure" variable with the individual categories of recorded-stage (bottom right) results in the same conclusions (OR 0.94).

Supplementary Table S5. Demographics, clinical characteristics, care processes and outcomes of patients admitted as an emergency for the first time with alcohol-related liver disease between 2008/9 and 2017/18. Stratified by region of residence.

Characteristic	London N = 2,395	North West N = 4,058	Yorkshire & The Humber N = 769	North East N = 1,113	West Midlands N = 3,174	East Midlands N = 416	East of England N = 642	South West N = 2,085	South Central N = 1,648	South East Coast N = 1,227
Age, mean (SD)	53.1 (12.0)	52.5 (12.0)	51.5 (12.9)	52.2 (11.9)	53.7 (12.2)	52.8 (12.9)	55.5 (12.5)	54.1 (12.4)	54.6 (12.4)	55.3 (12.9)
Female	717 (30%)	1,377 (34%)	259 (34%)	403 (36%)	1,052 (33%)	145 (35%)	207 (32%)	694 (33%)	544 (33%)	413 (34%)
Case definition										
Primary	1,219 (51%)	2,156 (53%)	406 (53%)	601 (54%)	1,760 (55%)	211 (51%)	350 (55%)	1,121 (54%)	850 (52%)	680 (55%)
Uplift	1,176 (49%)	1,902 (47%)	363 (47%)	512 (46%)	1,414 (45%)	205 (49%)	292 (45%)	964 (46%)	798 (48%)	547 (45%)
Non-liver comorbidity										
0-1	2,115 (88%)	3,634 (90%)	678 (88%)	976 (88%)	2,818 (89%)	372 (89%)	570 (89%)	1,812 (87%)	1,441 (87%)	1,050 (86%)
2+	280 (12%)	424 (10%)	91 (12%)	137 (12%)	356 (11%)	44 (11%)	72 (11%)	273 (13%)	207 (13%)	177 (14%)
Hepatic failure	285 (12%)	675 (17%)	92 (12%)	127 (11%)	438 (14%)	74 (18%)	78 (12%)	217 (10%)	154 (9.3%)	205 (17%)
Ascites	836 (35%)	1,381 (34%)	291 (38%)	402 (36%)	1,311 (41%)	143 (34%)	253 (39%)	804 (39%)	647 (39%)	505 (41%)
Varices	379 (16%)	561 (14%)	128 (17%)	134 (12%)	449 (14%)	66 (16%)	117 (18%)	353 (17%)	307 (19%)	205 (17%)
Died in hospital	321 (13.4%)	605 (14.9%)	120 (15.6%)	150 (13.5%)	549 (17.3%)	67 (16.1%)	102 (15.9%)	295 (14.2%)	246 (14.9%)	247 (20.1%)

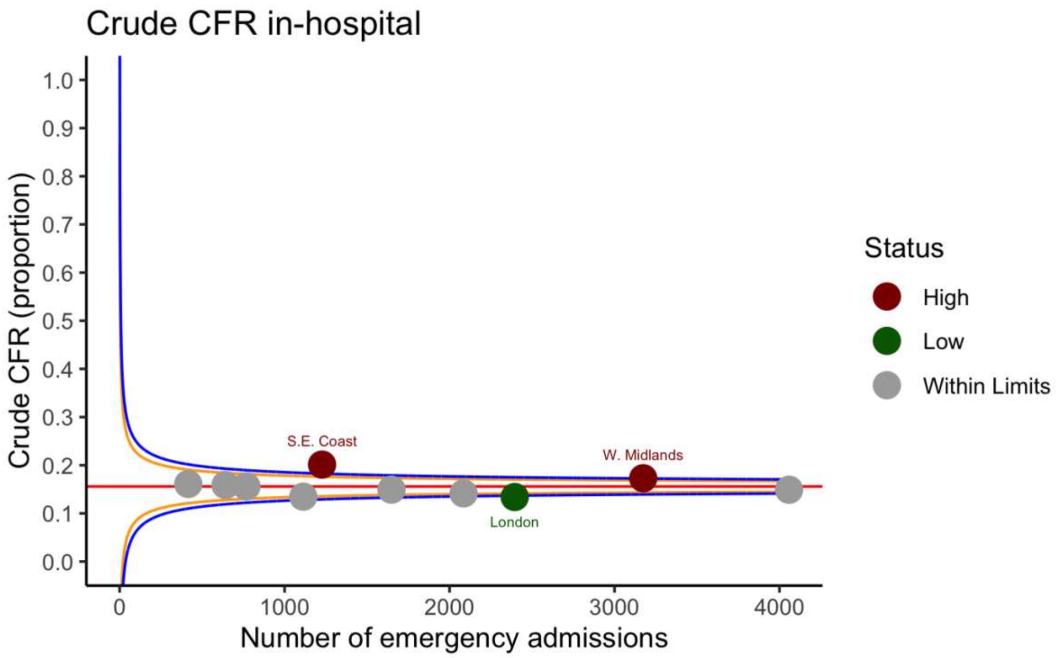

Supplementary Figure S3 (Exploratory Analysis): **Funnel chart showing crude case fatality rate (CFR) in-hospital by region of residence.** Regions marked in red are statistical "outliers" with a higher-than-average crude rate, those in grey are "within limits" and those in green are lower-than-average. The red horizontal line shows average performance across the ten regions. Inner yellow line shows 95% control limits, outer blue line shows 99% control limits based on Spiegelhalter's methodology. Data relate to patients admitted as an emergency for the first time with alcohol-related liver disease (2008/9 and 2017/18), registered with an English CPRD practice. Data tabulated below.

Supplementary Table S6 (Exploratory Analysis): Association between region of residence and death following first emergency admission for alcohol-related liver disease among people registered with CPRD practices in England, 2008/9 to 2017/18.

Characteristic	Crude CFR	OR	95% CI	p	aOR	95% CI	p
London (Ref)	13.4%	-	-	-	-	-	-
North West	14.9%	1.13	0.98, 1.31	0.10	1.06	0.91, 1.24	0.4
Yorkshire & The Humber	15.6%	1.19	0.95, 1.50	0.13	1.22	0.95, 1.54	0.11
North East	13.5%	1.01	0.82, 1.24	>0.9	1.02	0.81, 1.26	0.9
**West Midlands**	**17.3%**	**1.35**	**1.16, 1.57**	**<0.001**	**1.27**	**1.09, 1.49**	**0.003**
East Midlands	16.1%	1.24	0.93, 1.64	0.14	1.13	0.83, 1.53	0.4
East of England	15.9%	1.22	0.95, 1.55	0.11	1.11	0.86, 1.43	0.4
South West	14.1%	1.06	0.90, 1.26	0.5	1.02	0.85, 1.22	0.8
South Central	14.9%	1.13	0.95, 1.36	0.2	1.10	0.91, 1.33	0.3
**South East Coast**	**20.1%**	**1.63**	**1.36, 1.95**	**<0.001**	**1.36**	**1.12, 1.66**	**0.002**

OR=unadjusted odds ratio; aOR =risk-adjusted odds ratio; significant values in bold. Unadjusted: C-Statistic 0.536. Adjusted: C-Statistic 0.733. Adjusted for age group, sex, case definition, non-liver comorbidity, hepatic failure, ascites and varices.

Supplementary Appendix 1. Medcodes and corresponding readterms used for primary care records to identify entries relating to alcohol misuse and/or alcohol-related liver disease (ARLD). Codes for ARLD are marked with an asterix (*).

medcode	readterm
322	Moderate drinker - 3-6u/day
669	Nondependent alcohol abuse, unspecified
1399	Alcohol problem drinking
1618	Heavy drinker - 7-9u/day
2081	Alcoholism
2082	Alcohol withdrawal syndrome
2083	Alcohol detoxification
2084	Alcohol dependence syndrome
2925	Alcoholic polyneuropathy
3216	Acute alcoholic hepatitis*
3782	Intoxication – alcohol
3782	Intoxication – alcohol
4500	Korsakov's alcoholic psychosis
4506	Alcoholic gastritis
4743	Alcoholic cirrhosis of liver*
4915	Alcoholic cardiomyopathy
5611	[X]Mental and behavioural disorders due to use of alcohol
5740	Acute alcoholic intoxication in alcoholism
5758	[X]Chronic alcoholism
6169	Alcohol dependence syndrome NOS
6467	[X]Alcoholic hallucinosis
7123	[V]Personal history of alcoholism
7602	Chronic alcoholic hepatitis*
7692	Patient advised about alcohol
7746	Nondependent alcohol abuse
7885	Alcoholic liver damage unspecified
7943	Alcoholic hepatitis*
8030	[V]Alcohol abuse counselling and surveillance
8363	Oesophageal varices in alcoholic cirrhosis of the liver*
8388	[V]Alcohol rehabilitation
8430	H/O: alcoholism
8984	Ethyl alcohol causing toxic effect
8999	Heavy drinker
9169	[D]Alcohol blood level excessive
9489	Under care of community alcohol team
9508	[X]Acute alcoholic drunkenness
9849	Referral to community alcohol team
10161	O/E - alcoholic breath
10463	[X]Intent self poison/exposure to alcohol
10658	HoNOS item 3 - alcohol/drug problem

10691	Alcoholic fatty liver*
11106	Korsakov's alcoholic psychosis with peripheral neuritis
11140	Advice on alcohol consumption
11263	Wood alcohol causing toxic effect
11491	Health ed. – alcohol
11670	[X]Korsakov's psychosis, alcohol induced
11740	Alcohol misuse - enhanced services administration
12353	[X]Mental & behav dis due to use alcohol: psychotic disorder
12442	Alcohol disorder monitoring
12554	Referral to community drug and alcohol team
12974	Nondependent alcohol abuse, episodic
12976	Suspect alcohol abuse – denied
12977	Very heavy drinker - >9u/day
12982	Alcohol intake above recommended sensible limits
12984	Very heavy drinker
12985	Moderate drinker
13709	Blood ethanol level
14422	Urine ethanol
15503	Accidental poisoning by other ethyl alcohol and its products
16225	Alcohol withdrawal delirium
16237	Alcoholic psychoses
16587	[V]Problems related to lifestyle alcohol use
17149	[V]Blood-alcohol and blood-drug test
17259	[X]Delirium tremens, alcohol induced
17330	Alcoholic hepatic failure*
17607	[X]Alcoholic psychosis NOS
18156	Alcoholics anonymous
18252	Accidental poisoning by alcohol, NEC
18711	Lifestyle advice regarding alcohol
19217	Alcohol causing toxic effect
19401	Binge drinker
19489	Alcohol consumption screen
19494	Hazardous alcohol use
19754	Serum ethanol level
20514	[X]Mental and behav dis due to use alcohol: withdrawal state
20762	Alcohol amnestic syndrome
21624	Episodic acute alcoholic intoxication in alcoholism
21650	Admitted to alcohol detoxification centre
21713	Alcoholic fibrosis and sclerosis of liver*
21879	[X]Mental and behav dis due to use of alcohol: harmful use
22707	Drinking problem scale
23610	Nondependent alcohol abuse, continuous
23978	[X]Evid of alcohol involv determind by level of intoxication
24064	Continuous chronic alcoholism

24735	O/E - breath - alcohol smell
24984	Alcohol-induced chronic pancreatitis
25110	Alcohol withdrawal hallucinosis
26106	Episodic chronic alcoholism
26323	[X]Alcoholic dementia NOS
27342	Alcoholic dementia NOS
27518	Hangover (alcohol)
28150	Nondependent alcohol abuse NOS
28780	[X]Alcohol addiction
29691	Aversion therapy - alcoholism
30036	[X]Poisoning/exposure, ? intent, to alcohol
30162	[X]Alcoholic paranoia
30404	Alcoholic paranoia
30460	Alcoholism counselling
30604	Alcohol-induced epilepsy
30695	Harmful alcohol use
31443	Chronic alcoholism
31605	[X]Accident poisoning/exposure to alcohol
31742	Alcoholic myopathy
32850	Alcohol use disorders identification test
32927	[X]Alcohol withdrawal-induced seizure
32964	Alcohol abuse monitoring
33635	Chronic alcoholism NOS
33670	Other alcoholic psychosis
33839	Cerebellar ataxia due to alcoholism
35330	Alcohol consumption counselling
35859	Pregnancy alcohol advice
36296	Acute alcoholic intoxication in alcoholism NOS
36499	Alcohol causing toxic effect NOS
36714	Ethanol causing toxic effect
36748	Alcoholic encephalopathy
37264	Alcohol leaflet given
37691	[X]Chronic alcoholic brain syndrome
37946	Chronic alcoholic brain syndrome
38012	Accidental poisoning by ethanol, NOS
38061	Alcohol induced hallucinations
39327	[X]Mental and behav dis due to use alcohol: dependence syndr
39799	[X]Mental and behav dis due to use alcohol: amnesic syndrome
40530	Acute alcoholic intoxication, unspecified, in alcoholism
40541	Accidental poisoning by alcoholic beverages
40602	ADS - Alcohol dependence scale
41251	BMAST - Brief Michigan alcoholism screening test
41638	[X]Int self poison/exposure to alcohol at home
41920	Alcohol amnestic syndrome NOS

41983	Alcohol detoxification
42305	Severity of alcohol dependence questionnaire
43193	Unspecified chronic alcoholism
43813	Police:venesect-alcohol
44299	[X]Mental & behav dis due to use alcohol: acute intoxication
44783	Pain in lymph nodes after alcohol consumption
45169	[X]Men & behav dis due to use alcohol: oth men & behav dis
46677	Alcohol withdrawal regime
46684	Plasma ethanol level
47123	Alcohol counselling by other agencies
47555	Cerebral degeneration due to alcoholism
48514	Denatured alcohol causing toxic effect
48545	Alcohol questionnaire completed
50507	Alcohol dependence scale
53139	Accidental poisoning by other alcohols
54209	Advice to change alcohol intake
54505	Other alcoholic dementia
55415	Accidental poisoning by alcohol NOS
55536	Ethyl alcohol causing toxic effect NOS
56410	Delivery of rehabilitation for alcohol addiction
56441	Michigan alcoholism screening test
56947	Continuous acute alcoholic intoxication in alcoholism
57714	Alcohol dependence with acute alcoholic intoxication
57939	Pathological alcohol intoxication
59414	[X]Intent self poison alcohol unspecif place
59776	Last six months of drinking questionnaire
59873	AUDIT - Alcohol use disorders identification test
61187	[X]Accid poison/expos to alcohol unspecif place
61190	[X]Pois/expos ?intent to alcohol unspecif place
61583	[V]Medicolegal blood alcohol test
61750	Comprehensive drinker profile
62299	SADD - Short alcohol dependence data
62300	Short alcohol dependence data
63306	[X]Accident poison/exposure to alcohol at home
63457	CDP - Comprehensive drinker profile
63529	Alcohol misuse - enhanced service completed
63876	[X]Accid poison/expos alcohol in street/highway
64389	[X]Ment & behav dis due use alcohol: unsp ment & behav dis
65754	Alcohol-induced pseudo-Cushing's syndrome
65932	[X]Alcoholic jealousy
66831	SADQ - Severity of alcohol dependence questionnaire
67651	Alcoholic psychosis NOS
68111	Other alcoholic psychosis NOS
68159	[X]Poison/exposure ?intent, to alcohol at home

69331	MAST - Michigan alcoholism screening test
69407	[X]Pois/exp ?intent alcohol school/pub admin area
70939	Munich alcoholism test
84218	Disqualified from driving due to excess alcohol
90714	Alcohol screen - AUDIT completed
92908	[X]Accid poison/expos alcohol trade/service area
93415	Alcohol units per week
93624	Fast alcohol screening test
94485	Alcohol screen - AUDIT PC completed
94553	Referral to specialist alcohol treatment service
94669	Alcohol consumption screening test declined
94670	Alcohol misuse
94838	Alcohol use disorders identification test
94963	Alcohol screen - fast alcohol screening test completed
95181	Alcohol reduction programme
95650	Advice to change drink intake
95663	Alcohol screen - AUDIT C completed
95744	Alcohol use disorder identificatn test consumptn questionnre
95944	Alcohol assesment declined - enhanced services admin
96053	Brief intervention for excessive alcohol consumptn completed
96054	Extended intervention for excessive alcohol consumptn complt
96107	Single alcohol screening questionnaire
96219	[X]Pois/expos ?intent alcohol in street/highway
96259	Alcohol assessment declined
96993	Referral to alcohol brief intervention service
97085	[X]Int self poison alcohol other spec place
97126	Alcohol units consumed on heaviest drinking day
97163	Advice to change alcoholic drink intake
97163	Advice to change alcoholic drink intake
97261	Brief intervention for excessive alcohol consumptn declined
97309	Advised to contact primary care alcohol worker
97680	Declined referral to specialist alcohol treatment service
99877	Feels should cut down drinking
99985	Brief Michigan alcoholism screening test
100493	Five-shot questionnaire on heavy drinking
100989	Breath alcohol level
101426	Accidental poisoning by ethyl alcohol NOS
101543	Other alcohol causing toxic effect
101718	Drinks in morning to get rid of hangover
102121	Police:venesect-alcohol
102247	Extended interven for excessive alcohol consumption declined
102448	Higher risk drinking
102564	Advised to abstain from alcohol consumption
102577	Severity of alcohol dependence questionnaire

102665	Increasing risk drinking
102770	SADQ - Severity of alcohol dependence questionnaire
103459	Referral to community alcohol team declined
104458	Breath ethanol level
104611	Alcohol-induced acute pancreatitis
104702	[X]Accid pois/expos alcohol in sport/athletic area
104734	[X]Accid pois/expos to alcohol other spec place
108205	Clinical Institute Withdrawal Assessment for Alcohol, revisd
108644	In-house alcohol detoxification
109241	Alcohol Use Disorders Identification Test declined
109668	Specialist alcohol treatment service signposted
109675	Hospital alcohol liaison team report received
109800	Hospital attendance related to personal alcohol consumption
110494	Alcohol harm reduction programme
110624	Alcohol relapse prevention
110911	Accidental poisoning by denatured alcohol
111683	Removal of alcohol
111933	Family wellbeing discussion about alcohol
112448	[X]Pois/expos ?intent to alcohol at res institut
112645	[X]Intent self pois alcohol in street/highway
112648	[X]Acc pois/expos alcohol indust/construct area

Supplementary Appendix 2. Methods used for screening of "cause" fields listed on the death certificate among patients dying during index admission for ARLD.

Screening was applied to codes recorded in any "cause" field on the death certificates of in-hospital fatalities. To be flagged as a "cause-specific" death from ARLD, we required the presence of both liver and alcohol descriptor in one or more coded causes.

Hence, this included either one of K70.x codes or the appearance of a non-specific liver code in combination with at least one other alcohol-specific condition on the certificate (please refer to code lists for the LAA).

The commonest recorded K70.x code in the *first field* (cause_label) was K70.9 ALCOHOLIC LIVER DISEASE, UNSPECIFIED (accounting for 24.7% of in-hospital deaths), followed by K70.4 ALCOHOLIC HEPATIC FAILURE (20.6%), K70.3 ALCOHOLIC CIRRHOSIS OF LIVER (12.1%), K70.1 ALCOHOLIC HEPATITIS (6.0%) AND K70.0 ALCOHOLIC FATTY LIVER (0.4%). The commonest non-specific liver disease codes recorded in the *first field* (cause_label) were K74.6 OTHER AND UNSPECIFIED CIRRHOSIS OF LIVER (6.8%), K72.9 HEPATIC FAILURE, UNSPECIFIED (3%).
