# Supplementary material for: Outcomes of first emergency admissions for alcohol-related liver disease in England over a 10-year period: retrospective observational cohort study using linked electronic databases
Source: BMJ Open. 2023 Nov 22;13(11):e076955. doi: 10.1136/bmjopen-2023-076955 (PMC10668174; doi:10.1136/bmjopen-2023-076955)
Supplement: Author's manuscript [file bmjopen-2023-076955.draft_revisions.pdf]

BMJ Open

BMJ Open is committed to open peer review. As part of this commitment we make the peer review history of every article we publish publicly available.

When an article is published we post the peer reviewers' comments and the authors' responses online. We also post the versions of the paper that were used during peer review. These are the versions that the peer review comments apply to.

The versions of the paper that follow are the versions that were submitted during the peer review process. They are not the versions of record or the final published versions. They should not be cited or distributed as the published version of this manuscript.

BMJ Open is an open access journal and the full, final, typeset and author-corrected version of record of the manuscript is available on our site with no access controls, subscription charges or pay-per-view fees (http://bmjopen.bmj.com).

If you have any questions on BMJ Open's open peer review process please email info.bmjopen@bmj.com

BMJ Open

# Outcomes of first emergency admissions for alcohol-related liver disease in England over a ten year period: Retrospective observational cohort study using linked electronic databases

| | |
|---|---|
| Journal: | *BMJ Open* |
| Manuscript ID | bmjopen-2023-076955 |
| Article Type: | Original research |
| Date Submitted by the Author: | 22-Jun-2023 |
| Complete List of Authors: | Bodger, Keith; University of Liverpool, Dept. of Health Data Science, Insitute of Population Health; Liverpool University Hospitals NHS Foundation Trust, Gastroenterology Dept.<br>Mair, Thomas; University of Liverpool, Dept. of Health Data Science, Insitute of Population Health<br>Schofield, Peità; University of Liverpool, Department of Public Health & Policy<br>Silberberg, Benjamin; University of Liverpool, Dept. of Health Data Science, Insitute of Population Health<br>Hood, Steve; Liverpool University Hospitals NHS Foundation Trust, Gastroenterology Dept.<br>Fleming, Kate M; NHS England, Data & Analytics Transformation Directorate |
| Keywords: | Electronic Health Records, Hospitalization, EPIDEMIOLOGY, Hepatology < INTERNAL MEDICINE, Primary Care < Primary Health Care |
| | |

SCHOLARONE™
Manuscripts

*I, the Submitting Author has the right to grant and does grant on behalf of all authors of the Work (as defined in the below author licence), an exclusive licence and/or a non-exclusive licence for contributions from authors who are: i) UK Crown employees; ii) where BMJ has agreed a CC-BY licence shall apply, and/or iii) in accordance with the terms applicable for US Federal Government officers or employees acting as part of their official duties; on a worldwide, perpetual, irrevocable, royalty-free basis to BMJ Publishing Group Ltd ("BMJ") its licensees and where the relevant Journal is co-owned by BMJ to the co-owners of the Journal, to publish the Work in this journal and any other BMJ products and to exploit all rights, as set out in our licence.*

*The Submitting Author accepts and understands that any supply made under these terms is made by BMJ to the Submitting Author unless you are acting as an employee on behalf of your employer or a postgraduate student of an affiliated institution which is paying any applicable article publishing charge ("APC") for Open Access articles. Where the Submitting Author wishes to make the Work available on an Open Access basis (and intends to pay the relevant APC), the terms of reuse of such Open Access shall be governed by a Creative Commons licence – details of these licences and which Creative Commons licence will apply to this Work are set out in our licence referred to above.*

*Other than as permitted in any relevant BMJ Author's Self Archiving Policies, I confirm this Work has not been accepted for publication elsewhere, is not being considered for publication elsewhere and does not duplicate material already published. I confirm all authors consent to publication of this Work and authorise the granting of this licence.*

For peer review only

# Outcomes of first emergency admissions for alcohol-related liver disease in England over a ten year period: Retrospective observational cohort study using linked electronic databases

Keith Bodger[1,3], Thomas Mair[1], Pieta Schofield[2], Benjamin Silberberg[1], Steve Hood[3], Kate M Fleming[4]

1. Dept. of Health Data Science, Insitute of Population Health, University of Liverpool, UK
2. Dept. of Public Health & Policy, Insitute of Population Health, University of Liverpool, UK
3. Gastroenterology Dept., Liverpool University Hospitals NHS Foundation Trust, UK
4. National Disease Registration Service, Data & Analytics Transformation Directorate, NHS England, UK

## Abstract

**Objectives** To examine time trends in patient characteristics, care processes and case fatality of first emergency admission for alcohol-related liver disease (ARLD) in England.

**Design** National population-based, retrospective observational cohort study.

**Setting** Clinical Practice Research Datalink population of England, 2008/9-17/18. First emergency admissions were identified using the Liverpool ARLD Algorithm. We applied survival analyses and binary logistic regression to study prognostic trends.

**Outcome measures:** Patient characteristics; "recent" GP consultations and hospital admissions (preceding year); higher level care; deaths in-hospital (including certified cause) and within 365 days. Covariates were age, sex, deprivation status, coding pattern, ARLD stage, non-liver comorbidity, coding for ascites and varices.

**Results:** 17,575 first admissions (mean age: 53 years; 33% female; 32% from most deprived quintile). Almost half had codes suggesting advanced liver disease. In year before admission, only 47% of GP consulters had alcohol-related problems recorded; alcohol-specific diagnostic codes were absent in 24% of recent admission records. Overall, case fatality rate was 15% in-hospital and 34% at one year. Case-mix-adjusted odds of in-hospital death reduced by 6% per year (aOR: 0.94; 95% CI: 0.93-0.96) and 4% per year at 365 days (aOR: 0.96; 95% CI: 0.95-0.97).

**Conclusions:** Despite improving prognosis of first admissions, we found missed opportunities for earlier diagnosis in primary and secondary care. In 2017/18, one in seven were still dying during index admission, rising to one third within a year. Nationwide efforts are needed to promote earlier detection and intervention, and to minimise avoidable mortality after first presentation.

**Keywords:** Liver Diseases, Alcoholic; Algorithms; Epidemiology; Primary Health Care; Electronic Health Records; Hospitalization; Hospital Mortality; Emergency Service; Quality of Health Care

**Word count:** 4,724

**Corresponding author:** Keith Bodger, Reader & Consultant Gastroenterologist (Honorary), Department of Health Data Science, Institute of Population Health, University of Liverpool, kbodger@liverpool.ac.uk

## STRENGTHS AND LIMITATIONS OF THIS STUDY

- Nationwide population-based study sample covering all regions of England
- Application of a novel cohort discovery algorithm and the use of linked datasets covering primary and secondary care and death certification
- The datasets lack some important case mix information such as physiological or biochemical measures of disease severity which would allow for further risk-adjustment of case fatality rates.
- The cases are drawn from a large, representative sample of the English population but the findings may not be generalisable to all parts of the country.

For peer review only

## Introduction

Alcohol-related liver disease (ARLD) is a leading cause of premature death and is set to overtake ischaemic heart disease with respect to working years of life lost in the United Kingdom.[1] Major shortcomings in acute hospital treatment for ARLD in England were highlighted by the National Confidential Enquiry into Patient Outcome and Death (NCEPOD) in June 2013.[2] This widely publicised enquiry, combined with equally critical reports from the *Lancet Standing Commission* on Liver Disease,[3] prompted various UK initiatives to promote better care.[4–8] Similar calls for action have been raised in other countries.[9] However, in a final report in 2020, the commission's authors highlighted continuing concerns about late diagnosis and unacceptably high levels of mortality among patient admitted to hospital.[10]

A patient's first emergency hospital admission for ARLD is often the point of initial diagnosis.[11] High quality care is crucial to minimise early mortality and allow time for engagement of services promoting abstinence and recovery. However, knowledge of the characteristics and outcomes of first admissions for ARLD in England is limited. Nor is it known whether case-mix has changed or prognosis has improved over time.

Hospital administrative data is the main source of national statistics on admissions[12] and underpins proposed metrics for ARLD.[13] However, identifying and characterising cases using routine data is challenging.[11] Patients present to hospital with a wide range of clinical manifestations and with co-existing alcohol-related conditions.[14] Analysis of administrative data in our region showed that the standard case definition (based on a primary discharge diagnosis of ARLD)[12] identified only half of true admissions.[11] In response, we developed an algorithm to identify combinations of primary and secondary codes compatible with acute presentations of ARLD.[11] Recent national testing confirmed a doubling of estimates of annual admission volume using this method,[15] amounting to an under-recording of hospital activity attributable to ARLD of £70-127 million in NHS costs for 2020.[15] This has prompted calls for revision of methods for generating national statistics.[16]

We undertook a retrospective cohort study using linked electronic datasets and the Liverpool ARLD algorithm (LAA).[11,15] Our aims were to describe sociodemographics, clinical features, care processes (prior healthcare contacts, length of stay and provision of higher level care) and case fatality of patients presenting for the first time as an emergency with ARLD in England. We examined trends over a ten year period, spanning five years before and after the landmark NCEPOD report.[2]

## Methods

### Data Sources

The cohort was derived from the Clinical Practice Research Datalink (CPRD) which contains linked data from primary and secondary care and death certification (Office



For peer review only - http://bmjopen.bmj.com/site/about/guidelines.xhtml

for National Statistics).[17] CPRD is a database of anonymised electronic health records covering a broadly representative sample of UK residents. The primary care dataset contains records of consultations with diagnoses encoded as READ codes. Each case in the present study had linkage to Hospital Episode Statistics (HES), comprising administrative data for all inpatient care delivered in NHS hospitals. Care episodes in HES contain a list of diagnostic codes generated by clinical coders after discharge using the international classification of diseases (ICD-10) system. The dataset also included the supplementary Adult Critical Care dataset.

**Population of interest: First emergency admissions for ARLD**

We extracted data for all adult patients (≥18 years) with a first unplanned (non-elective) hospital admission (spell) for ARLD in their HES record. We defined a first (or "index") admission as having no such admission within the preceding ten years. We included all completed discharge records between 1st April 2008 and 31st March 2018, thereby covering ten fiscal years (2008/9 to 2017/18). The cohort is restricted to residents with a record of having been registered with a CPRD practice and with HES linkage.

**Case definitions for ARLD admissions**

We used the LAA to identify admissions for ARLD in HES, requiring discharge codes to comply with one of two alterntive case definitions ('primary' or 'uplift' subgroups). The primary definition selects cases with a K70 code recorded as primary diagnosis, as used for national statistics.[12] The 'uplift' definition identifies additional admissions with non-primary coding patterns, as previously reported.[11,15] Briefly, the uplift cases have a primary code for either a symptom, sign or complication of liver disease or for another alcohol-specific condition, accompanied by secondary codes compatible with ARLD (either a K70 code or a code for unspecified liver disease provided there are additional alcohol-specific conditions listed).

**Stage of liver disease**

In the present study, we further characterised LAA-identified cases by assigning the recorded stage of liver disease based on the K70 code present in the index admission (i.e. alcoholic fatty liver [K70.0], hepatitis [K70.1], sclerosis and fibrosis [K70.2], cirrhosis [K70.3], hepatic failure [K70.4] or "unspecified" [K70.9]). All primary and most uplift cases contain a K70.x code in their coding sequence. A minority of uplift cases lack a K70.x code, whereby a non-specific liver disease code is present (e.g. "Hepatic failure, unspecified" or "Other and unspecific cirrhosis of liver") and accompanied by other alcohol-specific conditions among discharge diagnoses. In these cases, the relevant stage was taken from the descriptor of the liver code.

**Patient-level covariates derived from the index admission**

Covariates were age, age group, sex, ethnicity, deprivation status, case definition (primary or uplift), stage of liver disease, the presence of codes for specific features of advanced liver disease (varices and ascites) and comorbidities. Socioeconomic



For peer review only - http://bmjopen.bmj.com/site/about/guidelines.xhtml

deprivation was defined by the English indices of deprivation 2015 associated with the lower layer super output area of residence and grouped into quintiles (1 = Least deprived, 5 = Most deprived), as described previously.[11,18] Comorbidities listed in the Charlson Comorbidity Index (CCI) were identified but we excluded liver disease codes and categorized cases into two groups (scores 0-1 and ≥2).

**Prior consultations in primary care**

All available primary care contacts within the year prior to each index admission were extracted and screened. For each contact, we created two binary variables. Firstly, a variable to indicate whether any alcohol-related code was present – signifying whether alcohol misuse or harms were suspected/recognised and recorded at the time. We used an extensive list of READ codes (See **Supplementary Table S5**). Second, whether a diagnosis of liver disease was recorded within the GP contact – a marker of prior recognition and elective diagnosis of ARLD.

**Prior emergency hospital admissions**

We identified all episodes of unplanned hospital care in the year prior to each patient's index admission for ARLD and screened the diagnosis fields for any alcohol-specific ICD-10 code. Hence, we determined whether any non-liver related alcohol problems were diagnosed and recorded during those prior emergency admissions.

**Care processes**

We determined length of index hospital stay. Using linkage to the intensive care dataset, we determined whether the patient received higher level care during their index admission, including high dependency (category 2) and intensive care (category 3).

**Time periods of interest**

We defined two 5-year time periods to allow comparison of cases discharged in the first and second half of the observation period (Period 1: April 2008 to March 2013; Period 2: April 2013 to March 2018). We also created a patient-level variable to indicate fiscal year of discharge.

**Outcomes**

We examined case fatality as reflected by all-cause death. This included any death in or out of hospital, identified from linkage to office of national statistics. We created binary variables for death during index admission (in-hospital death) and tracked all cases for death within 365-days from admission date. For survival analysis we calculated time-to-death measured in days from first admission. We used case fatality rates, as opposed to population-based relative mortality measures, since our purpose was to generate metrics focused on effectiveness of acute care after adjusting for severity of presenting illness.


For peer review only - http://bmjopen.bmj.com/site/about/guidelines.xhtml

## Certified cause of in-hospital death

We accessed linked records of death certification for all in-hospital deaths during index admissions to identify deaths attributed to ARLD based on finding codes for liver disease and/or its complications with specific reference to an alcohol aetiology.

## Statistical analyses

First, we summarised characteristics and care processes overall and for relevant strata and examined time trends. Categorical variables are reported as counts and percentages with group comparisons made using Chi Squared statistics. Continuous variables are reported as mean (sd), or median (IQR), as appropriate. We compared groups using relevant parametric (t-test or ANOVA) or non-parametric tests (Mann-Whitney U or Kruskal-Wallis).

Second, we calculated crude case fatality rates (cCFR) as the count of deaths (numerator) divided by the number of first admissions (denominator) per time period. We report deaths in-hospital and at 365 days from admission. We compared 5-year periods (Chi-square Test) and significance of monotonic year-on-year trends (Mann Kendall Trend Test). We undertook Kaplan-Meier survival analysis to compare cumulative probability of death up to 365 days, using log-rank test to determine significance between relevant strata (case-mix factors, period of discharge, region of residence). All cases had at least one year follow-up (no censoring).

Third, we determined factors associated with death using binary logistic regression. Models were used to adjust CFR for significant covariates. The dependent variables were all-cause death (either in-hospital or at 365 days). Covariates were added sequentially based on clinical relevance, previous research[11] and significant univariate associations ($p<0.10$), starting with age and sex and building a series of models to explore alternative risk-adjustment approaches. We generated time series of risk-adjusted CFRs by calculating standardised mortality ratios (SMR) for each time period, dividing the number of observed deaths by the number of expected deaths (sum of probabilities from relevant model). Each model used pooled data across the entire ten year period to allow comparisons over time. Risk-adjusted rates were calculated by multiplying the crude rate across 10 years by the SMR for each period (equivalent to indirect standardisation). The C-statistic and AIC values were calculated to assess model fit.

Fourth, we tested the significance of national time trends in adjusted case fatality by introducing five-year period or fiscal year of discharge into the relevant models. For year-on-year trends, the year variable was treated as continuous such that the odds ratios reflect the average change *per one-year increment* across the decade (assuming a linear trend). Sensitivity analyses using alternative variants of model covariates (e.g. absolute age as opposed to age group), selection of case-mix factors and potential

For peer review only

interactions were also explored. All analyses were undertaken in R (Version 4.1.2, R Core Team 2021, R Foundation for statistical computing, Vienna, Austria)

**Data Governance and Ethics Statements**

The CPRD has obtained ethical approval from the UK's National Research Ethics Service (NRES) Committee for observational research using anonymised data. Our study was approved by the Independent Scientific Advisory Committee (ISAC) for Medicines and Healthcare Products Regulatory Agency (MHRA) database research (protocol number 19_133). Patient consent for publication is not applicable.

## Results

**Characteristics of first emergency admissions**

The algorithm detected 17,575 first admissions. Their characteristics are summarised in **Table 1** (overall and stratified by case definition). Of the total, 9,374 (53%) were in the primary subgroup and 8,201 (47%) in the uplift subgroup (non-primary coding patterns). The magnitude of 'uplift' is consistent with previous findings.[11,15] The mean age at first admission was 53 years (range: 18-98), one third (33.1%) were female, 32% were resident in an area within the most deprived quintile of England. Less than 5% had non-white ethnicity recorded (hence, small numbers precluded more detailed reporting for individual groups).

**Prior primary care consultations** – 9,936 patients had a record of at least one GP consultation in the year before index admission (**Table 1**). Of this subgroup, just 4,702 (47.3%) had a READ code relating to either alcohol consumption or any alcohol-specific condition – suggesting that harmful drinking remained undetected or unrecorded in just over half of those with recent contact with their practice. Only 1,465 (14.7%) of the 9,936 patients had a code relevant to liver disease recorded, indicating that ARLD usually remained unsuspected or undiagnosed. These figures support the assertion that ARLD is rarely diagnosed electively in advance of an emergency admission to hospital.

**Prior emergency admissions** – forty one percent of the cohort (7,265 patients) had been admitted to hospital at least once as an emergency in the preceding year. Of these, 5,525 (76%) had a non-liver-related, alcohol-specific condition recorded in their list of discharge diagnoses. This leaves almost one in four with no preceding record of an alcohol-related diagnosis (despite subsequently being readmitted as an emergency with ARLD). This was equally true of cases with cirrhosis or liver failure at index admission, suggesting that serious alcohol-related harm may have gone unrecognised during these prior admissions (**Table 1**).

**Stage of liver disease** – stratification by stage of liver disease is shown in **Figure 1** with detailed characteristics provided in **Supplementary Table S1**. These data demonstrate

For peer review only

the high proportion of people having advanced liver disease at first hospital presentation (cirrhosis in 28% overall; hepatic failure in 13%). We found frequent use of K70.9 (alcoholic liver disease, unspecified) to encode first admissions in English HES data, whereby the disease stage was not specified (one in three admissions). The presence of additional codes for ascites (31%) and varices (12%) in many such cases suggests that a significant proportion of this 'unspecified' group also had severe disease (**Suppl. Table S1**).

Across the age bands, older groups had a lower the proportion with alcoholic hepatitis and a higher proportion with cirrhosis, as would be expected (**Fig. 1A**). The proportion with "severe liver disease" (codes for cirrhosis or hepatic failure) was similar among men and women (**Fig. 1B**). Interestingly, the proportion of first admissions with severe disease showed limited variation across deprivation quintiles and was slightly lower among those from most deprived areas (**Fig. 1C**).

**Time trends in case-mix –** we found small differences in sociodemographics between patients discharged in the second 5-year period compared with the first (**Suppl. Table S2**). Mean age was one year older (54 versus 53 years, $p<0.001$) and there was a slight reduction in proportion from most deprived areas (30% vs. 33%, $p<0.001$). There was an increased contribution of cirrhosis (31% vs. 25%, $p<0.001$) and hepatic failure (15% vs. 11%, $p<0.05$) but a reduction in unspecified stage (34% vs. 46%, $p<0.001$) (**Fig. 1D**). There was also a slight increase in proportion with codes for varices (16% vs. 15%, $p=0.031$). Levels of non-liver comorbidity were also higher (13% vs. 10% with score ≥2, $p<0.001$). Collectively, these findings suggest that patients at index admission were presenting with somewhat more advanced disease and greater burden of comorbidities. However, the possibility of improved coding over time needs to be considered (i.e. more precise recording of stage; more complete recording of comorbidities).

Concerningly, among patients with prior GP contact in the preceding year we found reductions in the proportion of consultations with a record of alcohol (46.6% vs. 48.1%, $p=0.13$) and liver disease codes (13.7% vs. 16.0%, $p<0.001$) – suggesting no improvement in screening, recognition or diagnosis in primary care. There was also a small reduction in recording of alcohol-specific codes in prior emergency admissions (75.6% vs. 76.7%, $p=0.3$). This implies no improvement in hospital-based diagnosis of alcohol-related harms among people destined to be readmitted acutely with ARLD within the year. The characteristics of patients discharged within each fiscal year are summarised in **Suppl. Table S3.**

**Care processes during first admission**

Across the ten years, the overall mean length of stay for first admissions was 12 days (**Table 1**), with a higher mean duration of hospitalisation for the hepatic failure subgroup at 16 days ($p<0.001$). The overall proportion who received higher level care



For peer review only - http://bmjopen.bmj.com/site/about/guidelines.xhtml

was 10%. Of those patients with hepatic failure recorded as their stage of liver disease, one in five (21%) received either high dependency or intensive care support during index admission. Mean length of stay was one day shorter in the second half of the decade than the first (11 days versus 12 days, p<0.001, **Suppl. Table S2**), and a significantly greater proportion of total cases received higher level care (11% versus 8.6%, p<0.001).

## Deaths following first admission

Overall, 2,708 deaths were recorded during 17,575 first admissions giving a crude case fatality rate (CFR) of 15.4% (**Table 1**). The median time to in-hospital death was nine days (mean: 14 days). One-in-five deaths occurred within just 48 hours of admission. Over one-in-three cases (39%) recorded as having hepatic failure at first presentation died during their first hospital admission. Within 365 days of first admission the cumulative death toll for the overall cohort had risen to 5,938 patients (34%).

Of the total in-hospital deaths, 2,569 (95%) had an ARLD-specific cause recorded on the death certificate. Of these verified deaths from ARLD, 1,738 (66.8%) were in the primary group and 831 (32.2%) in the uplift group. Hence, one in three cause-specific, certified deaths from ARLD during first admission were coded in an "uplift" pattern on discharge. These data provide further indirect evidence to support the validity of the LAA for cohort discovery and highlight the risk of missing a substantial mortality burden using HES-based statistics derived using the traditional primary definition.

Among those with a GP consultation within the past year (n=9,936), people who died during admission were less likely to have had coding of alcohol misuse or harms (39.3% vs 48.8%, p<0.01) or liver disease (10.1% vs 15.5%, p<0.05) than survivors. Patients with fatal first admission were less likely to have had an emergency admission in the past year than those who survived (35.9% vs 42.2%, p<0.01). Among those who had been previously hospitalized, alcohol-specific diagnoses had been recorded less often in those subsequently dying than in survivors (68.7% vs 77.2%, p<0.01).

Only 36.1% of in-hospital deaths received higher level care. Hence, almost two thirds of fatal cases were either not referred for high dependency or intensive care support or judged unlikely to benefit from escalation beyond ward level treatment.

## Factors associated with death

The demographic and clinical features of patients who had died, or survived, at the two binary end-points are shown in **Suppl. Table S4**. In bivariate analyses for both short and longer term deaths, we found significant associations with increasing age, female sex, primary (as opposed to uplift) case definition, increased levels of comorbidity, advanced stage of liver disease and the presence of codes for ascites or varices. Stratified survival analyses confirmed bivariate associations persisted throughout the year from admission (**Suppl. Fig. 1**). Multivariable models confirmed



For peer review only - http://bmjopen.bmj.com/site/about/guidelines.xhtml

independence of associations between the selected case-mix factors and odds of death in the cohort overall (**Table 3**). Interestingly, the marker for deprivation status (IMD quintile) was not independently associated with the outcomes and not retained in models. This suggests that severity of illness at the time of presentation dominated prognosis.

**Time trends in case fatality**

***Five-year periods*** – Comparison of unadjusted outcomes between the second and first five-year period revealed slightly lower crude in-hospital CFR (15% vs 16%, p=0.018). This did not quite reach significance for the 365 day end-point (33% vs 35%, p=0.063). Stage-specific crude analyses did show lower unadjusted rates for in-hospital death (hepatitis: 8.9% vs 9.9%, p=0.4; cirrhosis: 14.4% vs 17.7%, p=0.002; hepatic failure: 37.4% vs 41.2%, p=0.07; not specified: 9.7% vs 12.5%, p<0.001). Similarly, corresponding stage-specific rates at 365 days were lower for the second five-year period (hepatitis: 22.8% vs 24.5%, p=0.4; cirrhosis: 38.9% vs 41.9%, p=0.035; hepatic failure: 54.5% vs 57.7%, p=0.14; not specified: 26.0% vs 30.3%, p<0.001). Survival probabilities during the year after index admission were consistently higher for those discharged in the second period for the cohort as a whole and stage-specific analyses (**Fig. 2**). After adjusting for case-mix factors in relevant models, the adjusted odds of both in-hospital and 365 day death were significantly lower for those discharged in Period 2 (**Table 2**).

***Year-to-year trends*** – Time series are shown in **Fig. 3**. Overall, crude in-hospital CFR declined from 18.3% in 2008/9 to 16.1% in 2017/18 (p=0.03) (**Fig. 3A**). Corresponding changes in stage-specific crude rates were 45.4% to 41.0% for hepatic failure (p=0.13), 21.1% to 13.0% for cirrhosis (p=0.0006) and 16.1% to 8.8% for alcoholic hepatitis (p=0.47). Case-mix-adjusted rates for in-hospital death showed significant reductions overall and in stage-specific analyses (**Fig. 3B** and **Table 2**). In the final risk-adjusted model (**Table 2**), each one-year increment across the decade was associated (on average) with a 6% reduction in odds of in-hospital death overall (aOR: 0.94 [95% CI: 0.93-0.96], p<0.001). Stage-specific reductions in odds of dying during first admission were 3% per year for hepatic failure (p=0.004), 6% for cirrhosis (p<0.001) and 7% for alcoholic hepatitis.

Corresponding trends for death within 365 days are shown in **Fig. 3C** and **3D**. Overall crude rate reduced from 36.8% to 32.2% (p=0.02) and stage-specific crude rates showed downward trends – hepatic failure reducing from 59.2% to 53.9% (p=0.07), cirrhosis from 46.1% to 36.3% (p=0.02) and alcoholic hepatitis from 28.3% to 21.7% (p=0.15). After case-mix adjustment, each one-year increment was associated with a 4% reduction in odds of dying within a year of admission for the cohort as a whole (aOR: 0.96 [95% CI: 0.95-0.97], p<0.001). Stage-specific reductions in adjusted odds of death were also 4% per year for hepatic failure (p=0.011), cirrhosis (p<0.001) and alcoholic hepatitis (p=0.027).


For peer review only - http://bmjopen.bmj.com/site/about/guidelines.xhtml

## Sensitivity analyses

Key findings were unchanged in terms of the significance or magnitude of trends when risk adjustment models were fitted using variants of patient-level covariates (e.g. continuous variables for age or comorbidity scores, individual categories for each stage of liver disease).

# Discussion

The NCEPOD report in 2013 raised significant concerns about the quality of in-patient care received by people who died from ARLD in English hospitals.[2] The report and its recommendations were publicized widely and accompanied by parallel efforts from the *Lancet Commission* to drive improvements in policy and practice.[3,5,8,13] Despite these nationwide initiatives, the commission concluded in 2020 that little had improved.[10] However, the authors acknowledged a relative paucity of data and advocated improved metrics to monitor true workload and outcomes.

We believe that statistics focused on first admissions offer a suitable focus for actionable metrics derived from routine data.[11] Our study represents the first detailed description of characteristics and outcomes of index admissions for a representative sample of the English population. The predominance of males and disproportionate contribution of people living in more deprived areas is consistent with known epidemiological patterns for ARLD.[1] First admissions encompass a diversity of manifestations of liver disease and alcohol misuse, ranging from early-stage liver disease to rapidly fatal liver failure. However, we have shown that at least half of all cases within the CPRD population had advanced disease at first emergency admission.

Our data show that ARLD is frequently diagnosed for the first time during unplanned hospitalization. Only 14.7% of GP consulters in the preceding year had a prior code for liver disease recorded – suggesting liver disease was unsuspected and remained undiagnosed in the majority. A previous study examined the earliest instance of coding for cirrhosis (regardless of aetiology) within linked CPRD-HES records (1998-2009), reporting this occurred first within an emergency record in half the cases.[19] The latter study did not study codes for earlier stages of liver disease, as reported here. We also examined GP consultations in the preceding year for an extensive list of codes relating to alcohol excess, finding them absent from half those records – implying missed opportunities to screen for, or detect, alcohol-related harms.

Similar issues were apparent in secondary care. Alcohol-related codes were absent in almost a quarter of those with an unplanned hospitalisation in the last year. The corresponding figure was 31.3% for patients who died in-hospital. It seems unlikely that such patients lacked evidence of alcohol excess or alcohol-specific conditions during prior admissions. Across the decade we found no improvement in rates of recognition/recording of alcohol-related problems in prior contacts within primary or



For peer review only - http://bmjopen.bmj.com/site/about/guidelines.xhtml

secondary care among people subsequently hospitalised with ARLD. Deficiencies in early detection of ARLD have been recognised internationally.[20]

Over the ten year period we found improvements in short and medium term prognosis of first admissions. Although welcome, the absolute magnitude of changes was relatively small. In 2017/18, one in seven were still dying during their first hospitalisation, rising to one third within a year. Whilst survival was better in the post-NCEPOD era (Period 2), yearly time series suggested no step-change or accelerated reduction in case fatality during a sustained period of awareness-raising and national initiatives.[2–5,7] Directly comparable statistics for other countries are lacking.

Of those who died during first ARLD admission, 36% received higher level care. This is comparable to a figure of 39% in the NCEPOD audit of deaths in British hospitals during 2011.[2] In that report, reviewers found that 31.2% of those deemed eligible to benefit from higher level care did not receive it. This prompted recommendations for improved decision-making and access. Universal prognostic pessimism about the benefits of organ support in severe liver disease has been challenged.[21] We cannot judge suitability of cases for escalation in the present study but we observed a small increase in the proportion of fatalities receiving level two or three care over a ten year period. Nevertheless, it is striking to find that two thirds of patients dying during their first admission were deemed unsuitable for escalation.

Our findings confirm limitations of simple case definitions for defining workload and outcome of ARLD within administrative data.[11,15,16,19] Recent studies of international burden of "decompensated" cirrhosis have required relevant codes to appear as the primary discharge diagnosis.[22] Our analysis of linked death registrations shows that many of the sickest patients in English HES data have a non-primary coding pattern (a third of cause-specific in-hospital deaths). Additionally, our findings suggests caution in defining "severe" liver disease based on primary codes for cirrhosis or liver failure.[23] Patients with unspecified stage of ARLD (K70.9) accounted for 28% of first admissions and one in every four in-hospital deaths in our study. If administrative data are to be used to quantify burden, track the impact of interventions or benchmark provider performance then methods for case identification and risk-adjustment require care.[16] The LAA-based approach,[11,15] combined with supplementary methods for case-mix categorization reported here, provide a novel methodology that may support the development of improved metrics – building on recent recommendations from the *Lancet* commission.[13]

Our study has some limitations. First, HES data lacks granular information on disease severity (e.g. clinical scores or laboratory parameters), limiting case-mix adjustment. However, we mitigated this by applying condition-specific surrogate markers of stage/severity and adjusted for potential imbalance in a range of prognostically important baseline covariates. This goes beyond the traditional generic set of


For peer review only - http://bmjopen.bmj.com/site/about/guidelines.xhtml

demographic and comorbidity variables. Furthermore, we conducted multiple stratified analyses and alternative risk-adjustment models to improve confidence in conclusions. Over the ten year period, first admissions appeared to have somewhat more advanced disease and a higher burden of comorbidities. However, the possibility of changes to coding accuracy or completeness over time cannot be excluded. However, the fact that there were reductions in crude and adjusted CFRs suggests prognostic improvements are genuine rather than artefactual.

Second, whilst CPRD practices serve a representative population of English residents,[17] our results are not generalisable to every locality. Provider-level analyses are not possible using this general practice-based dataset. A key aim of our study was to examine prior events in primary care. Linked data are not available for the entire country. Further work on national HES is needed to explore regional and provider variation. Our algorithm-based case-finding approach (LAA),[11,15] combined with additional methods reported here for case-mix categorization and risk-adjustment, provides a novel methodology to support the development of improved national metrics – building on recommendations of the *Lancet* commission.[13]

Third, although LAA performance in detecting true cases of ARLD against hospital records has been reported previously,[11,15] we could not conduct formal validity testing within the present study (hospital data was limited to HES). However, linkage to death certification confirmed that the vast majority of in-hospital deaths in the uplift subgroup were related to alcoholic liver disease. The LAA is based on coding patterns observed in English HES data and it is unknown whether such variations exist in other countries.

In conclusion, we found evidence for gradual improvement in prognosis for patients admitted for the first time with ARLD in England over a ten year period. Nevertheless, at least one in seven patients were still dying during the index hospitalization and a third within a year. Many patients had contacts with health services in the year prior to admission but without recognition of alcohol-related harms, adding to concerns about missed opportunities for screening and intervention.[24] Our observations support much of the pessimism expressed in the *Lancet Commission*'s final report.[10] This highlights the need for concerted action in public health interventions and primary and secondary care to improve prevention, early detection and inpatient treatment of alcohol-related harms.

## Funding and acknowledgments

This work was funded by the UK Department of Health (Connected Health Cities programme) and delivered by the Northern Health Science Alliance. The funding sponsors had no role in the design and conduct of the study; in the collection, management, analysis and interpretation of the data; or in the preparation, review or approval of the manuscript.



For peer review only - http://bmjopen.bmj.com/site/about/guidelines.xhtml

## Data Availability Statement

This study is based in part on data from the Clinical Practice Research Datalink obtained under licence from the UK Medicines and Healthcare products Regulatory Agency. The data is provided by patients and collected by the NHS as part of their care and support. The interpretation and conclusions contained in this study are those of the author/s alone. Copyright ©2023, re-used with the permission of The Health & Social Care Information Centre. All rights reserved. CPRD data are not publicy or freely available but can be obtained under special licence (https://cprd.com/data-access)

## Patient and Public Involvement

Patients or the public were not involved in the design, conduct, reporting or dissemination plans of our research.

## Authorship

*Guarantor of the article*: K.B.

*Author contributions*: K.B. wrote the manuscript and all authors reviewed and approved the final submission. K.B., K.F., B.S. and P.S. designed the research with input from other authors. K.B, B.S. and T.M. curated and analysed the data, with input from P.S. and K.F.

Conflicts of interest: None declared.

## References

1. Williams R, Alexander G, Armstrong I, et al. Disease burden and costs from excess alcohol consumption, obesity, and viral hepatitis: fourth report of the Lancet Standing Commission on Liver Disease in the UK. Lancet Lond Engl 2018;391(10125):1097–107.

2. National confidential enquiry into patient outcome and death. Measuring the units: a review of patients who died with alcohol-related liver disease [Internet]. 2013 [cited 2019 Oct 1]. Available from: https://www.ncepod.org.uk/2013report1/downloads/MeasuringTheUnits_FullReport.pdf

3. Williams R, Horton R. Liver disease in the UK: a Lancet Commission. Lancet Lond Engl 2013;382(9904):1537–8.

4. Williams R, Aspinall R, Bellis M, et al. Addressing liver disease in the UK: a blueprint for attaining excellence in health care and reducing premature mortality from lifestyle issues of excess consumption of alcohol, obesity, and viral hepatitis. Lancet Lond Engl 2014;384(9958):1953–97.

5. Williams R, Ashton K, Aspinall R, et al. Implementation of the Lancet Standing Commission on Liver Disease in the UK. Lancet Lond Engl 2015;386(10008):2098–111.

6. Dyson JK, Rajasekhar P, Wetten A, et al. Implementation of a "care bundle" improves the management of patients admitted to hospital with decompensated cirrhosis. Aliment Pharmacol Ther 2016;44(10):1030–8.

For peer review only

7. McPherson S, Dyson J, Austin A, Hudson M. Response to the NCEPOD report: development of a care bundle for patients admitted with decompensated cirrhosis-the first 24 h. Frontline Gastroenterol 2016;7(1):16–23.

8. Kiat C, O'Mahony S. Tackling the epidemic: the NCEPOD report on alcohol-related liver disease and the Lancet Commission on Liver Disease. J R Coll Physicians Edinb 2018;48(4):291–2.

9. Kanwal F, Kramer JR, Buchanan P, et al. The quality of care provided to patients with cirrhosis and ascites in the Department of Veterans Affairs. Gastroenterology 2012;143(1):70–7.

10. Williams R, Aithal G, Alexander GJ, et al. Unacceptable failures: the final report of the Lancet Commission into liver disease in the UK. Lancet Lond Engl 2020;395(10219):226–39.

11. Kallis C, Dixon P, Silberberg B, et al. Reducing variation in hospital mortality for alcohol-related liver disease in North West England. Aliment Pharmacol Ther 2020;52(1):182–95.

12. 1.8 Emergency admissions for alcohol related liver disease [Internet]. NHS Digit. [cited 2022 Sep 28];Available from: https://digital.nhs.uk/data-and-information/publications/statistical/ccg-outcomes-indicator-set/june-2020/domain-1-preventing-people-from-dying-prematurely-ccg/1-8-emergency-admissions-for-alcohol-related-liver-disease

13. Williams R, Alexander G, Aspinall R, et al. New metrics for the Lancet Standing Commission on Liver Disease in the UK. Lancet Lond Engl 2017;389(10083):2053–80.

14. McPherson S, Lucey MR, Moriarty KJ. Decompensated alcohol related liver disease: acute management. BMJ 2016;352:i124.

15. Dhanda A, Bodger K, Hood S, et al. The Liverpool alcohol-related liver disease algorithm identifies twice as many emergency admissions compared to standard methods when applied to Hospital Episode Statistics for England. Aliment Pharmacol Ther 2022;(In Press).

16. King J, Walker K, Van Der Meulen J, Bernal W. Editorial: the burden of alcohol-related liver disease emergency admissions in England may be twice as high as previously thought. Aliment Pharmacol Ther 2022;

17. Wolf A, Dedman D, Campbell J, et al. Data resource profile: Clinical Practice Research Datalink (CPRD) Aurum. Int J Epidemiol 2019;48(6):1740–1740g.

18. Shawihdi M, Dodd S, Kallis C, et al. Nationwide improvement in outcomes of emergency admission for ulcerative colitis in England, 2005-2013. Aliment Pharmacol Ther 2019;50(2):176–92.

19. Ratib S, West J, Fleming KM. Liver cirrhosis in England-an observational study: are we measuring its burden occurrence correctly? BMJ Open 2017;7(7):e013752.

20. Shah ND, Ventura-Cots M, Abraldes JG, et al. Alcohol-Related Liver Disease Is Rarely Detected at Early Stages Compared With Liver Diseases of Other Etiologies Worldwide. Clin Gastroenterol Hepatol Off Clin Pract J Am Gastroenterol Assoc 2019;17(11):2320-2329.e12.

21. McPhail MJW, Parrott F, Wendon JA, Harrison DA, Rowan KA, Bernal W. Incidence and Outcomes for Patients With Cirrhosis Admitted to the United Kingdom Critical Care Units. Crit Care Med 2018;46(5):705–12.

For peer review only

22. GBD 2017 Cirrhosis Collaborators. The global, regional, and national burden of cirrhosis by cause in 195 countries and territories, 1990-2017: a systematic analysis for the Global Burden of Disease Study 2017. Lancet Gastroenterol Hepatol 2020;5(3):245–66.

23. Roberts SE, John A, Brown J, Napier DJ, Lyons RA, Williams JG. Early and late mortality following unscheduled admissions for severe liver disease across England and Wales. Aliment Pharmacol Ther 2019;49(10):1334–45.

24. Burton R, Sheron N. Missed opportunities for intervention in alcohol-related liver disease in the UK. Lancet Gastroenterol Hepatol 2017;2(7):469–71.

For peer review only

**Table 1. Demographics, clinical characteristics, care processes and outcomes of patients admitted as an emergency for the first time with alcohol-related liver disease between 2008/9 and 2017/18**. Patients registered with an English CPRD practice. Data are shown for the cohort overall ('Total') and by case definition based on the Liverpool ARLD Algorithm ('Primary' or 'Uplift').

| Characteristic | Total | Case definition | | p |
|---|---|---|---|---|
| | (n=17,575) | **Primary** (n=9,374) | **Uplift** (n=8,201) | |
| Age, *mean (sd)* | 53 (12) | 53 (12) | 54 (13) | * |
| Sex | | | | |
| *Female* | 5,820 (33.1%) | 3,335 (35.6%) | 2,485 (30.3%) | ** |
| *Male* | 11,755 (66.9%) | 6,039 (64.4%) | 5,716 (69.7%) | |
| Deprivation quintile | | | | |
| *1 - Least deprived* | 2,435 (13.9%) | 1,330 (14.2%) | 1,105 (13.5%) | *ns* |
| *2* | 2,735 (15.6%) | 1,481 (15.8%) | 1,254 (15.3%) | |
| *3* | 3,028 (17.2%) | 1,613 (17.2%) | 1,415 (17.3%) | |
| *4* | 3,837 (21.8%) | 2,077 (22.2%) | 1,760 (21.5%) | |
| *5 - Most deprived* | 5,540 (31.5%) | 2,873 (30.6%) | 2,667 (32.5%) | |
| Stage of liver disease | | | | |
| *Fatty liver* | 942 (5.4%) | 182 (1.9%) | 760 (9.3%) | ** |
| *Hepatitis* | 2,369 (13.5%) | 1,729 (18.4%) | 640 (7.8%) | |
| *Fibrosis and sclerosis* | 62 (0.4%) | 28 (0.3%) | 34 (0.4%) | |
| *Cirrhosis* | 5,001 (28.5%) | 2,759 (29.4%) | 2,242 (27.3%) | |
| *Hepatic failure* | 2,354 (13.4%) | 1,982 (21.1%) | 372 (4.5%) | |
| *Not specified* | 6,847 (39.0%) | 2,694 (28.7%) | 4,153 (50.6%) | |
| Ascites | 6,589 (37.5%) | 4,648 (49.6%) | 1,941 (23.7%) | ** |
| Varices | 2,704 (15.4%) | 1,508 (16.1%) | 1,196 (14.6%) | * |
| Comorbidity score (non-liver) | | | | |
| *0-1* | 15,506 (88.2%) | 8,440 (90.0%) | 7,066 (86.2%) | * |
| *2+* | 2,069 (11.8%) | 934 (10.0%) | 1,135 (13.8%) | |
| Five year period of discharge | | | | |
| *2008/9 - 2012/13* | 7,566 (43.0%) | 4,102 (43.8%) | 3,464 (42.2%) | * |
| *2013/14 - 2017/18* | 10,009 (57.0%) | 5,272 (56.2%) | 4,737 (57.8%) | |
| GP contact in last year | 9,936 (56.5%) | 5,346 (57.0%) | 4,590 (56.0%) | *ns* |
| *With alcohol codes recorded §* | 4702 (47.3%) | 2528 (47.3%) | 2174 (47.4%) | *ns* |
| *With liver codes recorded §* | 1465 (14.7%) | 865 (16.2%) | 600 (13.1%) | ** |
| Emergency admission in last year | 7,265 (41.3%) | 3,240 (34.6%) | 4,025 (49.1%) | * |
| *With alcohol codes recorded ¶* | 5,525 (76.0%) | 2,393 (73.9%) | 3,132 (77.8.%) | ** |
| Length of stay, mean (sd) | 12 (15) | 14 (16) | 9 (13) | ** |
| Higher level care during admission | 1,750 (10.0%) | 970 (10.3%) | 780 (9.5%) | *ns* |
| Died in-hospital | 2,708 (15.4%) | 1,779 (19.0%) | 929 (11.3%) | ** |
| Died within 365 days of admission | 5,938 (33.8%) | 3,642 (38.9%) | 2,296 (28.0%) | ** |

* p<0.05; **p<0.001 between primary and uplift groups; ns = not statistically significant; na = not applicable; § = percentages based on those with a GP contact within the last year; ¶ = percentages based on those with an emergency admission within the last year.

**Table 2. Factors associated with death following first emergency admission for alcohol-related liver disease among people registered with CPRD practices in England, 2008/9 to 2017/18.** The final case-mix adjustment models included seven patient level baseline covariates. Additional time-related variables were then added to investigate time trends, using either fiscal year of discharge (continuous) or 5-year time period. Figure 3 illustrates the time trends in crude and case-mix adjusted case fatality rates derived using these models.

| **Covariate** | **Death during first admission** | | | | **Death within 365 days** | | | |
|---|---|---|---|---|---|---|---|---|
| | OR | 95% CI | aOR | 95% CI | OR | 95% CI | aOR | 95% CI |
| **Age Group** *(Ref: <40 years)* | | | | | | | | |
| *40-49* | **1.42** | 1.18, 1.70 | **1.31** | 1.09, 1.58 | **1.33** | 1.18, 1.50 | **1.2** | 1.06, 1.37 |
| *50-59* | **2.36** | 1.99, 2.81 | **2.02** | 1.70, 2.43 | **2.01** | 1.79, 2.26 | **1.67** | 1.48, 1.88 |
| *60-69* | **3.39** | 2.85, 4.04 | **2.86** | 2.39, 3.43 | **2.97** | 2.63, 3.35 | **2.36** | 2.08, 2.68 |
| *70+* | **4.11** | 3.41, 4.98 | **3.33** | 2.73, 4.07 | **5.39** | 4.69, 6.20 | **4.14** | 3.57, 4.80 |
| **Sex** *(Ref: Female)* | | | | | | | | |
| *Male* | **0.86** | 0.79, 0.93 | **0.79** | 0.72, 0.87 | 1.00 | 0.94, 1.07 | **0.93** | 0.86, 0.99 |
| **Case Definition** *(Ref: Primary)* | | | | | | | | |
| *Uplift* | **0.55** | 0.50, 0.59 | **0.76** | 0.69, 0.84 | **0.61** | 0.57, 0.65 | **0.75** | 0.70, 0.81 |
| **Non-liver comorbidity** *(Ref: 0-1)* | | | | | | | | |
| *2+* | **2.38** | 2.14, 2.64 | **1.97** | 1.75, 2.21 | **2.92** | 2.66, 3.20 | **1.97** | 1.75, 2.21 |
| **Hepatic Failure** *(Ref: Any other stage)* | | | | | | | | |
| *Hepatic failure* | **4.73** | 4.29, 5.21 | **4.03** | 3.64, 4.48 | **2.87** | 2.62, 3.13 | **2.32** | 2.11, 2.55 |
| **Ascites** *(Ref: No)* | | | | | | | | |
| *Yes* | **2.03** | 1.87, 2.21 | **1.48** | 1.35, 1.62 | **2.31** | 2.17, 2.47 | **1.86** | 1.74, 2.00 |
| **Varices** *(Ref: No)* | | | | | | | | |
| *Yes* | **1.35** | 1.21, 1.50 | **1.2** | 1.07, 1.34 | **1.41** | 1.30, 1.53 | **1.21** | 1.11, 1.33 |
| | Model 1, C-statistic: 0.733 | | | | Model 2, C-statistic: 0.710 | | | |
| **Fiscal Year** *(per year)* | | | | | | | | |
| | **0.98** | 0.96, 0.99 | **0.94** | 0.93, 0.96 | **0.98** | 0.97, 0.99 | **0.96** | 0.95, 0.97 |
| | Fiscal year added to Model 1, C-statistic: 0.737 | | | | Fiscal year added to Model 2, C-statistic: 0.710 | | | |
| **Five Year Period** *(Ref: 2008/9 to 2012/13)* | | | | | | | | |
| *2013/14 to 2017/18* | **0.91** | 0.83, 0.98 | **0.76** | 0.69, 0.83 | **0.91** | 0.83, 0.98 | **0.83** | 0.77, 0.88 |
| | Five Year Period added to Model 1, C-statistic: 0.736 | | | | Five Year Period added to Model 2, C-statistic: 0.708 | | | |

OR=unadjusted odds ratio; aOR =risk-adjusted odds ratio; significant ORs are in bold text (p values <0.001 in all cases)

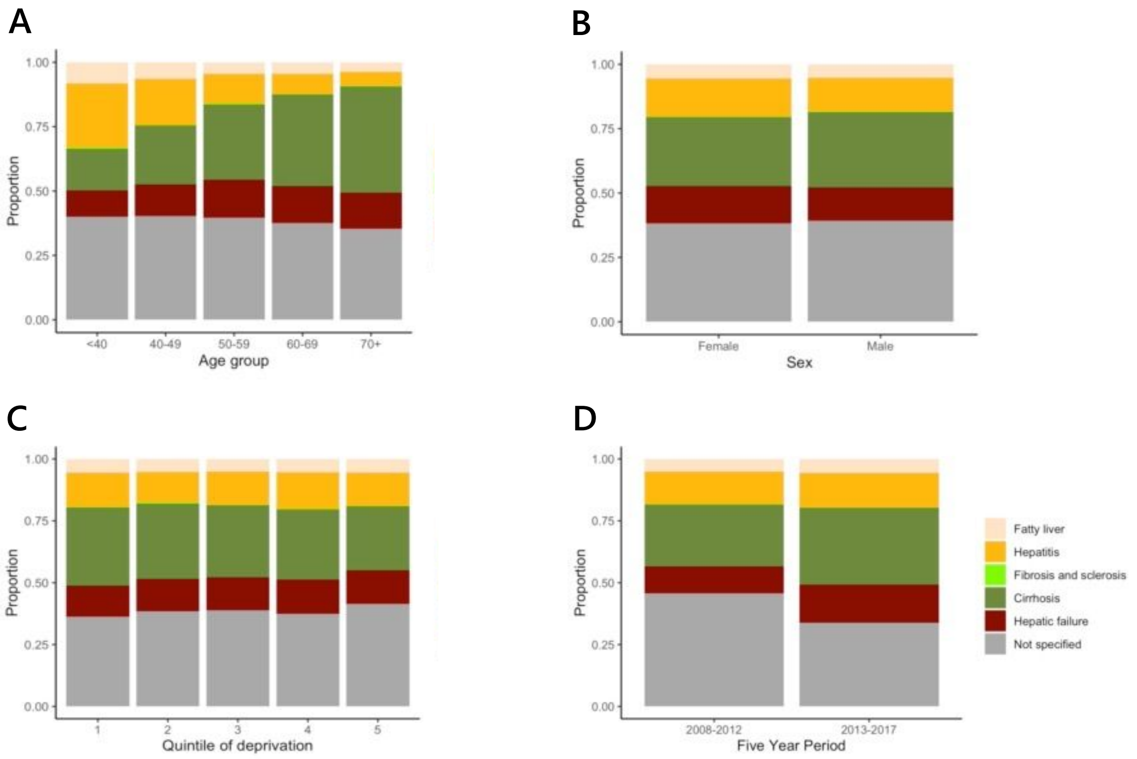


**Figure 1. Distribution of stage of alcohol-related liver disease (ARLD) at first emergency admission identified from the CPRD population of England.** Stage is based on the ICD-10 codes recorded on discharge (n=17,575 cases). Stratified by: **(A)** Age group, **(B)** Sex, **(C)** Deprivation Quintile (1=Least, 5=Most deprived areas) and **(D)** Five-year period of discharge.



For peer review only - http://bmjopen.bmj.com/site/about/guidelines.xhtml

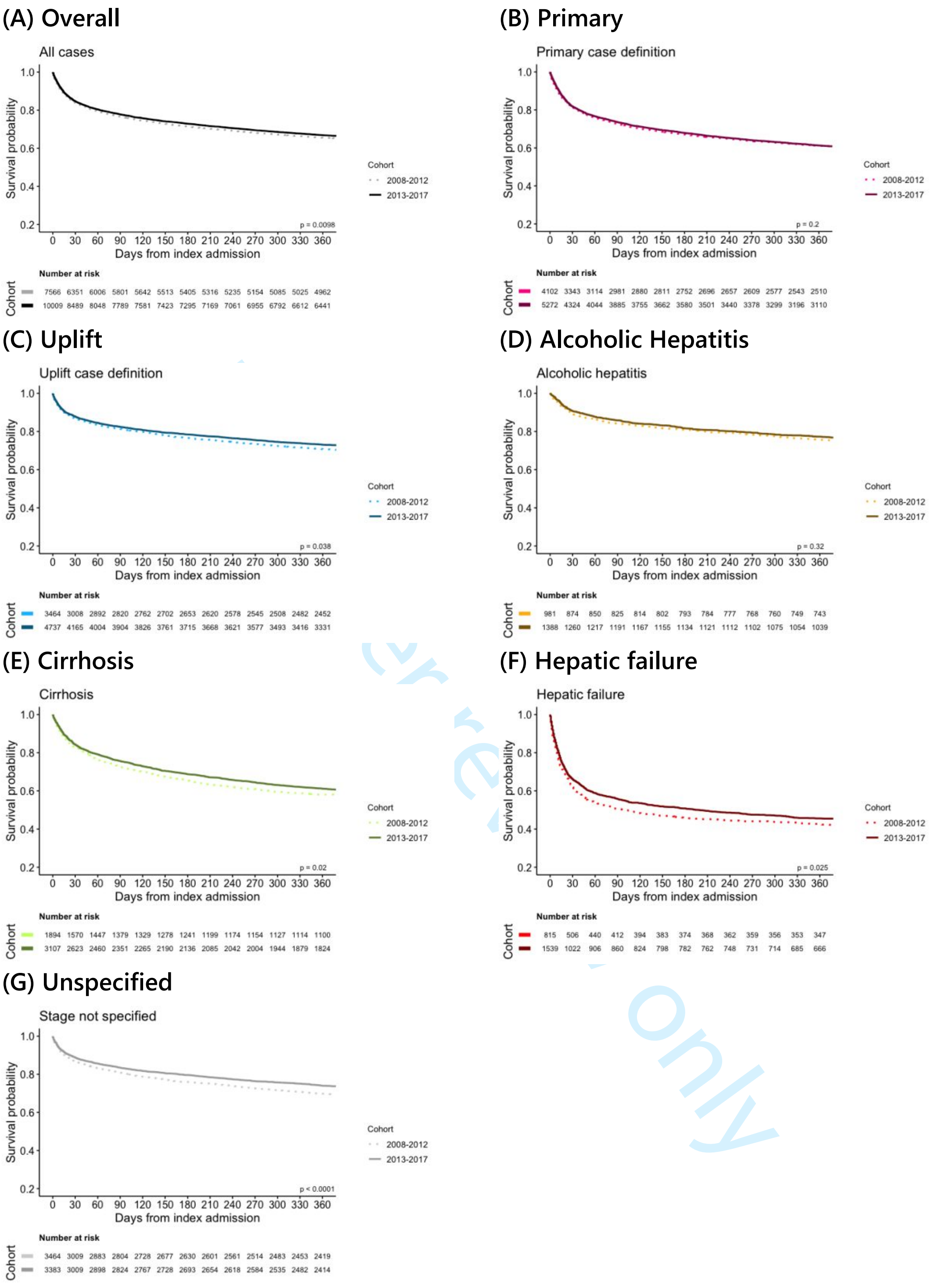


**Figure 2. Kaplan-Meier survival probability following first emergency admission with a diagnosis of alcohol-related liver disease, categorised into 5-year cohorts.** Cases were derived from the CPRD population of England, 2008/9 to 2017/18. Five-year cohorts based on fiscal year of discharge (2008/9 to 2012/13 versus 20013/14 to 2017/18). **(A)** Overall (all cases, n=17,575), **(B)** Primary case definition (n=9,374), **(C)** Uplift case definition (n=8,201), **(D)** Alcoholic hepatitis (n=2,369), **(E)** Cirrhosis (n=5,001), **(F)** Hepatic failure (n=2,354), **(G)** Unspecified stage of liver disease (n=6,847). p values represent significance level of log rank test.


For peer review only - http://bmjopen.bmj.com/site/about/guidelines.xhtml

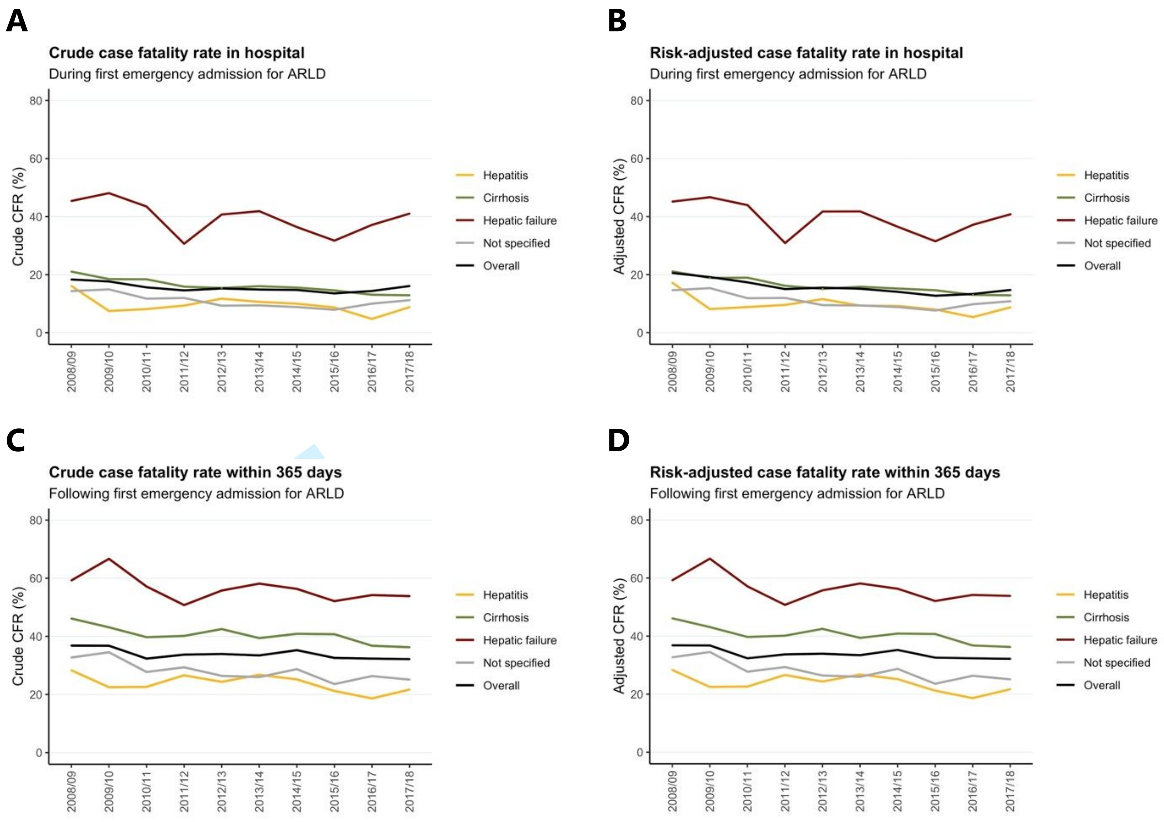


**Figure 3. Time trends in case fatality rates (CFR) for first emergency admissions for alcohol-related liver disease in England, overall and by stage of liver disease. (A)** Crude CFR in-hospital, **(B)** Risk-adjusted CFR in-hospital, **(C)** Crude CFR within 365 days, **(D)** Risk-adjusted CFR within 365 days. Risk-adjustment for overall rates was by age, sex, case definition (primary or uplift), comorbidity score (non-liver), stage of liver disease (liver failure or not) and coding for ascites and varices. Binary logistic regression models confirmed significant downward linear trends over the ten years overall, before and after risk-adjustment (See Table 3). For stage-specific rates, risk-adjustment was by age, sex, case definition and comorbidity score, with additional relevant models confirming significance of trends.



For peer review only - http://bmjopen.bmj.com/site/about/guidelines.xhtml

# Supplementary Figures and Tables

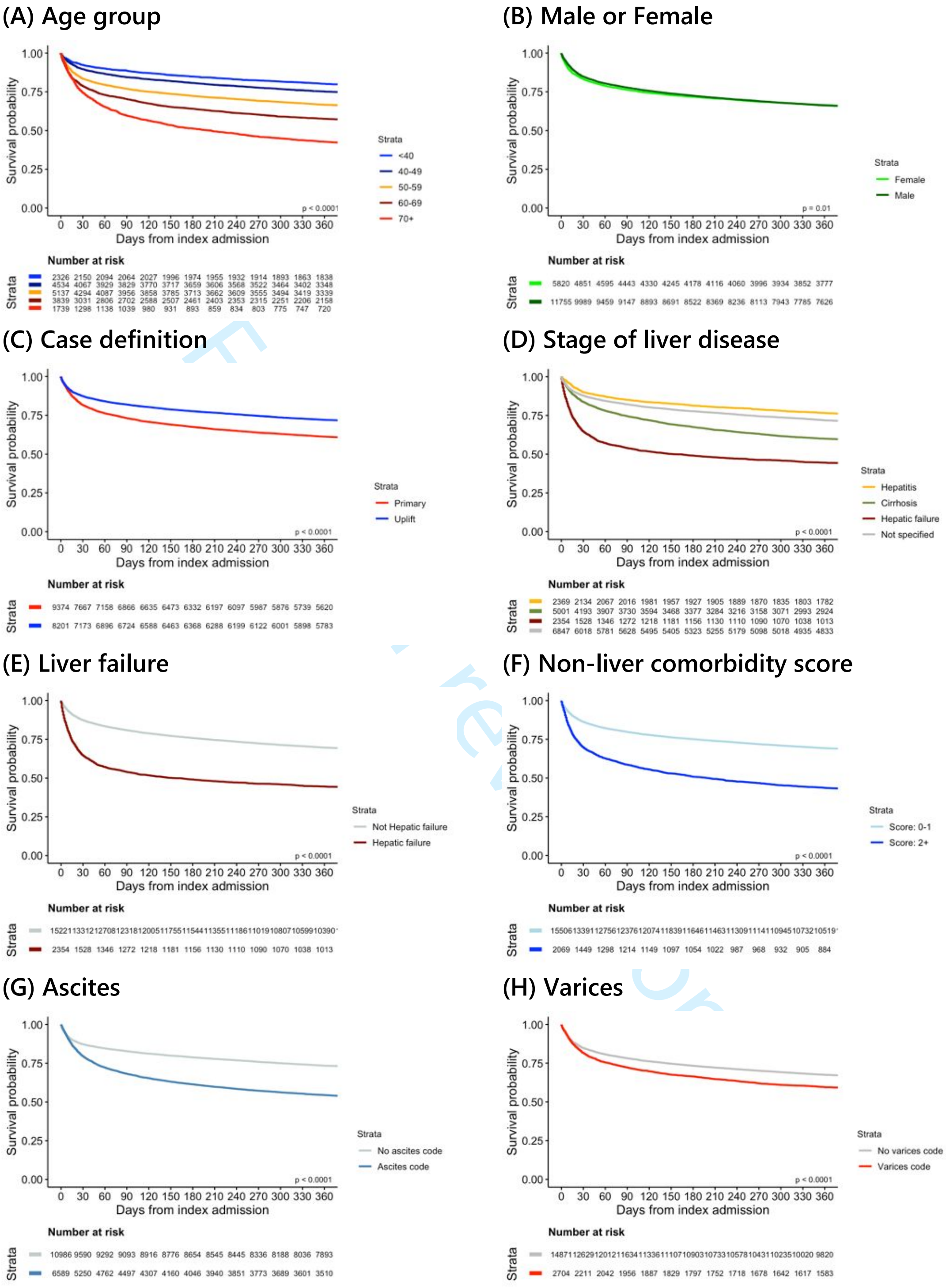


**Supplementary Figure S1. Kaplan-Meier survival probability of first emergency admissions with a diagnosis of alcohol-related liver disease, stratified by patient-level covariates.** **(A)** Age group, **(B)** Sex, **(C)** Case definition, **(D)** Stage of liver disease (selected stages only), **(E)** Liver failure or Not, **(F)** Non-liver comorbidity score, **(G)** Presence of codes for ascites, **(H)** Presence of codes for varices. All p values were significant between strata by log rank test.



For peer review only - http://bmjopen.bmj.com/site/about/guidelines.xhtml

**Supplementary Table S1.** Demographics, clinical characteristics, care processes and outcomes of patients admitted as an emergency for the first time with alcohol-related liver disease between 2008/9 and 2017/18. Patients registered with an English CPRD practice. Stratified by stage of liver disease. Excludes fatty liver (n=942) and fibrosis & sclerosis (n=62).

| Characteristic | Hepatitis (n=2,369) | Cirrhosis (n=5,001) | Hepatic failure (n=2,354) | Not specified (n=6,847) | p |
|---|---|---|---|---|---|
| Age, mean (sd) | 48 (12) | 57 (12) | 55 (12) | 53 (12) | ** |
| Sex | | | | | |
| *Female* | 848 (35.8%) | 1,562 (31.2%) | 839 (35.6%) | 2,227 (32.5%) | ** |
| *Male* | 1,521 (64.2%) | 3,439 (68.8%) | 1,515 (64.4%) | 4,620 (67.5%) | |
| Deprivation quintile | | | | | |
| *1 - Least deprived* | 332 (14.0%) | 769 (15.4%) | 307 (13.0%) | 883 (12.9%) | ** |
| *2* | 337 (14.2%) | 831 (16.6%) | 357 (15.2%) | 1,054 (15.4%) | |
| *3* | 404 (17.1%) | 882 (17.6%) | 404 (17.2%) | 1,176 (17.2%) | |
| *4* | 560 (23.6%) | 1,084 (21.7%) | 533 (22.6%) | 1,437 (21.0%) | |
| *5 - Most deprived* | 736 (31.1%) | 1,435 (28.7%) | 753 (32.0%) | 2,297 (33.5%) | |
| Case definition | | | | | |
| *Primary* | 1,729 (73.0%) | 2,759 (55.2%) | 1,982 (84.2%) | 2,694 (39.3%) | ** |
| *Uplift* | 640 (27.0%) | 2,242 (44.8%) | 372 (15.8%) | 4,153 (60.7%) | |
| Ascites | 531 (22.4%) | 2,472 (49.4%) | 1,383 (58.8%) | 2,109 (30.8%) | ** |
| Varices | 154 (6.5%) | 1,261 (25.2%) | 415 (17.6%) | 845 (12.3%) | ** |
| Comorbidity score (non-liver) | | | | | |
| *0-1* | 2,255 (95.2%) | 4,150 (83.0%) | 2,040 (86.7%) | 6,132 (89.6%) | ** |
| *2+* | 114 (4.8%) | 851 (17.0%) | 314 (13.3%) | 715 (10.4%) | |
| Five-year period of discharge | | | | | |
| *2008/9 – 2012/13* | 981 (41.4%) | 1,894 (37.9%) | 815 (34.6%) | 3,464 (50.6%) | ** |
| *2013/14 – 2017/18* | 1,388 (58.6%) | 3,107 (62.1%) | 1,539 (65.4%) | 3,383 (49.4%) | |
| GP contact in last year | 1,268 (53.5%) | 2,921 (58.4%) | 1,336 (56.8%) | 3,887 (56.8%) | ** |
| *With alcohol codes recorded §* | 661 (52.1%) | 1302 (44.6%) | 619 (46.3%) | 1849 (47.6%) | ** |
| *With liver codes recorded §* | 94 (7.4%) | 593 (20.3%) | 228 (17.1%) | 514 (13.2%) | *ns* |
| Emergency admission in last year | 918 (38.8%) | 2,011 (40.2%) | 832 (35.3%) | 3,029 (44.2%) | ** |
| *With alcohol codes recorded ¶* | 771 (84.0%) | 1,407 (70.0%) | 593 (71.3%) | 2,352 (77.7%) | ** |
| Length of stay, mean (sd) | 12 (15) | 12 (16) | 16 (18) | 10 (13) | ** |
| Higher level care during admission | 138 (5.8%) | 508 (10.2%) | 496 (21.1%) | 556 (8.1%) | ** |
| Died in-hospital | 221 (9.3%) | 781 (15.6%) | 912 (38.7%) | 761 (11.1%) | ** |
| Died within 365 days of admission | 557 (23.5%) | 2,003 (40.1%) | 1,309 (55.6%) | 1,931 (28.2%) | ** |

* p<0.05; **p<0.001 between groups; ns = not significant; na = not applicable; § = percentages based on those with a GP contact within the last year; ¶ = percentages based on those with an emergency admission within the last year.

**Supplementary Table S2.** Demographics, clinical characteristics, care processes and outcomes of patients admitted as an emergency for the first time with alcohol-related liver disease between 2008/9 and 2017/18. Patients registered with an English CPRD practice. Stratified by 5-year period of discharge.

| Characteristic | Total | 5 Year Period of Discharge | | p |
|---|---|---|---|---|
| | **08/09 to 17/18** (n=17,575) | **08/09 to 12/13** (n=7,566) | **13/14 to 17/18** (n=10,009) | |
| Age, *mean (sd)* | 53 (12) | 53 (12) | 54 (12) | ** |
| Sex | | | | |
| *Female* | 5,820 (33.1%) | 2,477 (33%) | 3,343 (33%) | *ns* |
| *Male* | 11,755 (66.9%) | 5,089 (67%) | 6,666 (67%) | |
| Deprivation quintile | | | | |
| *1 - Least deprived* | 2,435 (13.9%) | 977 (13%) | 1,458 (15%) | ** |
| *2* | 2,735 (15.6%) | 1,162 (15%) | 1,573 (16%) | |
| *3* | 3,028 (17.2%) | 1,223 (16%) | 1,805 (18%) | |
| *4* | 3,837 (21.8%) | 1,674 (22%) | 2,163 (22%) | |
| *5 - Most deprived* | 5,540 (31.5%) | 2,530 (33%) | 3,010 (30%) | |
| Case definition | | | | |
| Primary | 7,595 (51.1%) | 4,102 (54%) | 5,272 (53%) | * |
| Uplift | 7,272 (48.9%) | 3,464 (46%) | 4,737 (47%) | |
| Stage of liver disease | | | | |
| *Fatty liver* | 942 (5.4%) | 385 (5.1%) | 557 (5.6%) | ** |
| *Hepatitis* | 2,369 (13.5%) | 981 (13%) | 1,388 (14%) | |
| *Fibrosis and sclerosis* | 62 (0.4%) | 27 (0.4%) | 35 (0.3%) | |
| *Cirrhosis* | 5,001 (28.5%) | 1,894 (25%) | 3,107 (31%) | |
| *Hepatic failure* | 2,354 (13.4%) | 815 (11%) | 1,539 (15%) | |
| *Not specified* | 6,847 (39.0%) | 3,464 (46%) | 3,383 (34%) | |
| Ascites | 6,589 (37.5%) | 2,799 (37%) | 3,790 (38%) | *ns* |
| Varices | 2,704 (15.4%) | 1,113 (15%) | 1,591 (16%) | * |
| Comorbidity score (non-liver) | | | | |
| *0-1* | 15,506 (88.2%) | 6,773 (90%) | 8,733 (87%) | ** |
| *2+* | 2,069 (11.8%) | 793 (10%) | 1,276 (13%) | |
| GP contact in last year | 9,936 (56.5%) | 4,513 (59.6%) | 5,423 (54.2%) | ** |
| *With alcohol codes recorded §* | 4702 (47.3%) | 2,173 (48.1%) | 2,529 (46.6%) | *ns* |
| *With liver codes recorded §* | 1465 (14.7%) | 724 (16.0%) | 741 (13.7%) | ** |
| Emergency admission in last year | 7,265 (41.3%) | 3,127 (41%) | 4,138 (41%) | *ns* |
| *With alcohol codes recorded ¶* | 5,525 (76.0%) | 2,398 (76.7%) | 3,127 (75.6%) | *ns* |
| Length of stay, mean (sd) | 12 (15) | 12 (15) | 11 (14) | ** |
| Higher level care during admission | 1,750 (10.0%) | 652 (8.6%) | 1,098 (11%) | ** |
| Died in-hospital | 2,708 (15.4%) | 1,222 (16%) | 1,486 (15%) | * |
| Died within 365 days of admission | 5,938 (33.8%) | 2,614 (35%) | 3,324 (33%) | *ns* |

* $p<0.05$; ** $p<0.001$ between time periods; ns = not significant; § = percentages based on those with a GP contact within the last year; ¶ = percentages based on those with an emergency admission within the last year.



For peer review only - http://bmjopen.bmj.com/site/about/guidelines.xhtml

**Supplementary Table S3.** Demographics, clinical characteristics, care processes and outcomes of patients admitted as an emergency for the first time with alcohol-related liver disease between 2008/9 and 2017/18. Patients registered with an English CPRD practice. Stratified by fiscal year of discharge.

| **Characteristic** | **2008/09** | **2009/10** | **2010/11** | **2011/12** | **2012/13** | **2013/14** | **2014/15** | **2015/16** | **2016/17** | **2017/18** |
|---|---|---|---|---|---|---|---|---|---|---|
| | N = 1,494 | N = 1,512 | N = 1,645 | N = 1,626 | N = 1,692 | N = 1,741 | N = 1,771 | N = 1,992 | N = 2,044 | N = 2,058 |
| **Age**, mean (sd) | 52 (12) | 52 (12) | 52 (12) | 53 (13) | 53 (12) | 54 (12) | 54 (12) | 54 (12) | 54 (12) | 55 (13) |
| **Age group** | | | | | | | | | | |
| *<40* | 220 (14.7%) | 232 (15.3%) | 257 (15.6%) | 244 (15.0%) | 236 (13.9%) | 209 (12.0%) | 225 (12.7%) | 228 (11.4%) | 236 (11.5%) | 239 (11.6%) |
| *40-49* | 429 (28.7%) | 419 (27.7%) | 439 (26.7%) | 431 (26.5%) | 451 (26.7%) | 440 (25.3%) | 444 (25.1%) | 486 (24.4%) | 518 (25.3%) | 477 (23.2%) |
| *50-59* | 423 (28.3%) | 403 (26.7%) | 491 (29.8%) | 433 (26.6%) | 490 (29.0%) | 542 (31.1%) | 536 (30.3%) | 580 (29.1%) | 629 (30.8%) | 610 (29.6%) |
| *60-69* | 293 (19.6%) | 337 (22.3%) | 326 (19.8%) | 352 (21.6%) | 361 (21.3%) | 390 (22.4%) | 382 (21.6%) | 486 (24.4%) | 451 (22.1%) | 461 (22.4%) |
| *70+* | 129 (8.6%) | 121 (8.0%) | 132 (8.0%) | 166 (10.2%) | 154 (9.1%) | 160 (9.2%) | 184 (10.4%) | 212 (10.6%) | 210 (10.3%) | 271 (13.2%) |
| **Sex** | | | | | | | | | | |
| *Female* | 505 (33.8%) | 488 (32.3%) | 527 (32.0%) | 526 (32.3%) | 559 (33.0%) | 533 (30.6%) | 607 (34.3%) | 669 (33.6%) | 697 (34.1%) | 709 (34.5%) |
| *Male* | 989 (66.2%) | 1,024 (67.7%) | 1,118 (68.0%) | 1,100 (67.7%) | 1,133 (67.0%) | 1,208 (69.4%) | 1,164 (65.7%) | 1,323 (66.4%) | 1,347 (65.9%) | 1,349 (65.5%) |
| **Deprivation quintile** | | | | | | | | | | |
| *1 - Least deprived* | 174 (11.6%) | 209 (13.8%) | 201 (12.2%) | 207 (12.7%) | 243 (14.4%) | 253 (14.5%) | 240 (13.6%) | 287 (14.4%) | 320 (15.7%) | 301 (14.6%) |
| *2* | 236 (15.8%) | 222 (14.7%) | 243 (14.8%) | 250 (15.4%) | 264 (15.6%) | 274 (15.7%) | 267 (15.1%) | 331 (16.6%) | 289 (14.1%) | 359 (17.4%) |
| *3* | 264 (17.7%) | 219 (14.5%) | 282 (17.1%) | 259 (15.9%) | 264 (15.6%) | 298 (17.1%) | 336 (19.0%) | 362 (18.2%) | 376 (18.4%) | 368 (17.9%) |
| *4* | 334 (22.4%) | 339 (22.4%) | 361 (21.9%) | 344 (21.2%) | 398 (23.5%) | 369 (21.2%) | 365 (20.6%) | 424 (21.3%) | 438 (21.4%) | 465 (22.6%) |
| *5 - Most deprived* | 486 (32.5%) | 523 (34.6%) | 558 (33.9%) | 566 (34.8%) | 523 (30.9%) | 547 (31.4%) | 563 (31.8%) | 588 (29.5%) | 621 (30.4%) | 565 (27.5%) |
| **Case definition** | | | | | | | | | | |
| *Primary* | 843 (56.4%) | 819 (54.2%) | 878 (53.4%) | 870 (53.5%) | 906 (53.5%) | 897 (51.5%) | 959 (54.2%) | 1,054 (52.9%) | 1,092 (53.4%) | 1,056 (51.3%) |
| *Uplift* | 651 (43.6%) | 693 (45.8%) | 767 (46.6%) | 756 (46.5%) | 786 (46.5%) | 844 (48.5%) | 812 (45.8%) | 938 (47.1%) | 952 (46.6%) | 1,002 (48.7%) |
| **Stage of liver disease** | | | | | | | | | | |
| *Fatty liver* | 59 (3.9%) | 82 (5.4%) | 94 (5.7%) | 75 (4.6%) | 95 (5.6%) | 103 (5.9%) | 98 (5.5%) | 113 (5.7%) | 116 (5.7%) | 107 (5.2%) |
| *Hepatitis* | 180 (12.0%) | 200 (13.2%) | 221 (13.4%) | 203 (12.5%) | 230 (13.6%) | 254 (14.6%) | 250 (14.1%) | 264 (13.3%) | 295 (14.4%) | 272 (13.2%) |
| *Fibrosis and sclerosis* | 7 (0.5%) | 3 (0.2%) | 6 (0.4%) | 6 (0.4%) | 6 (0.4%) | 5 (0.3%) | 10 (0.6%) | 5 (0.3%) | 6 (0.3%) | 8 (0.4%) |
| *Cirrhosis* | 323 (21.6%) | 341 (22.6%) | 413 (25.1%) | 441 (27.1%) | 480 (28.4%) | 518 (29.8%) | 553 (31.2%) | 631 (31.7%) | 650 (31.8%) | 651 (31.6%) |
| *Hepatic failure* | 130 (8.7%) | 156 (10.3%) | 161 (9.8%) | 199 (12.2%) | 226 (13.4%) | 203 (11.7%) | 261 (14.7%) | 309 (15.5%) | 358 (17.5%) | 351 (17.1%) |
| *Not specified* | 795 (53.2%) | 730 (48.3%) | 750 (45.6%) | 702 (43.2%) | 655 (38.7%) | 658 (37.8%) | 599 (33.8%) | 670 (33.6%) | 619 (30.3%) | 669 (32.5%) |
| **Ascites** | 219 (14.7%) | 205 (13.6%) | 234 (14.2%) | 263 (16.2%) | 250 (14.8%) | 294 (16.9%) | 329 (18.6%) | 305 (15.3%) | 307 (15.0%) | 298 (14.5%) |
| **Varices** | 14 (0.9%) | 16 (1.1%) | 20 (1.2%) | 27 (1.7%) | 26 (1.5%) | 36 (2.1%) | 26 (1.5%) | 31 (1.6%) | 33 (1.6%) | 29 (1.4%) |
| **Comorbidity Score (non-liver)** | | | | | | | | | | |
| *0-1* | 1,341 (89.8%) | 1,357 (89.7%) | 1,469 (89.3%) | 1,456 (89.5%) | 1,505 (88.9%) | 1,515 (87.0%) | 1,538 (86.8%) | 1,746 (87.7%) | 1,792 (87.7%) | 1,787 (86.8%) |
| *2+* | 153 (10.2%) | 155 (10.3%) | 176 (10.7%) | 170 (10.5%) | 187 (11.1%) | 226 (13.0%) | 233 (13.2%) | 246 (12.3%) | 252 (12.3%) | 271 (13.2%) |
| **GP contact in last year** | 940 (62.9%) | 922 (61.0%) | 967 (58.8%) | 966 (59.4%) | 950 (56.1%) | 983 (56.5%) | 959 (54.2%) | 1,113 (55.9%) | 1,078 (52.7%) | 1,058 (51.4%) |
| *With alcohol codes recorded §* | 433 (46.1%) | 439 (47.6%) | 486 (50.3%) | 460 (47.6%) | 465 (48.9%) | 505 (51.4%) | 449 (46.8%) | 527 (47.3%) | 480 (44.5%) | 458 (43.3%) |
| *With liver codes recorded §* | 164 (17.4%) | 161 (17.5%) | 164 (17.0%) | 138 (14.3%) | 137 (14.4%) | 163 (16.6%) | 129 (13.5%) | 155 (13.9%) | 143 (13.3%) | 111 (10.5%) |
| **Emergency admission in last year** | 583 (39.0%) | 665 (44.0%) | 689 (41.9%) | 662 (40.7%) | 702 (41.5%) | 718 (41.2%) | 740 (41.8%) | 821 (41.2%) | 837 (40.9%) | 848 (41.2%) |
| *With alcohol codes ¶* | 443 (76.0%) | 506 (76.1%) | 541 (78.5%) | 502 (75.8%) | 538 (76.6%) | 556 (77.4%) | 543 (73.4%) | 616 (75.0%) | 631 (75.4%) | 649 (76.5%) |
| **Length of stay**, mean (sd) | 13 (16) | 13 (17) | 12 (15) | 11 (15) | 12 (15) | 12 (15) | 12 (15) | 11 (14) | 11 (14) | 11 (14) |
| **Higher level care during admission** | 92 (6.2%) | 149 (9.9%) | 157 (9.5%) | 146 (9.0%) | 149 (8.8%) | 192 (11.0%) | 211 (11.9%) | 198 (9.9%) | 224 (11.0%) | 232 (11.3%) |
| **Died in-hospital** | 274 (18.3%) | 267 (17.7%) | 257 (15.6%) | 237 (14.6%) | 258 (15.2%) | 259 (14.9%) | 261 (14.7%) | 270 (13.6%) | 294 (14.4%) | 331 (16.1%) |
| **Died with 365 days of admission** | 550 (36.8%) | 556 (36.8%) | 532 (32.3%) | 548 (33.7%) | 574 (33.9%) | 582 (33.4%) | 624 (35.2%) | 649 (32.6%) | 661 (32.3%) | 662 (32.2%) |

§ = percentages based on those with a GP contact within the last year; ¶ = percentages based on those with an emergency admission within the last year.

review only

For peer review only - http://bmjopen.bmj.com/site/about/guidelines.xhtml

**Supplementary Table S4. Demographics and clinical characteristics of those who survived or died following first emergency admission with alcohol-related liver disease between 2008/09 and 2017/18.** Data are shown for in-hospital deaths and cumulative deaths at 365 days from admission.

| Characteristic | In-hospital | | | Within 365 days | | |
|---|---|---|---|---|---|---|
| | Survived N=14,867 | Died N=2,708 | p | Survived N=11,637 | Died N=5,939 | p |
| Age, mean (sd) | 53 (12) | 58 (12) | ** | 51 (12) | 57 (12) | ** |
| Age Group | | | | | | |
| <40 | 2,153 (14.5%) | 173 (6.4%) | ** | 1,862 (16.0%) | 464 (7.8%) | ** |
| 40-49 | 4,071 (27.4%) | 463 (17.1%) | | 3,405 (29.3%) | 1,129 (19.0%) | |
| 50-59 | 4,318 (29.0%) | 819 (30.2%) | | 3,421 (29.4%) | 1,716 (28.9%) | |
| 60-69 | 3,018 (20.3%) | 821 (30.3%) | | 2,207 (19.0%) | 1,632 (27.5%) | |
| 70+ | 1,307 (8.8%) | 432 (16.0%) | | 742 (6.4%) | 997 (16.8%) | |
| Sex | | | | | | |
| Female | 4,844 (32.6%) | 976 (36.0%) | ** | 3,857 (33.1%) | 1,963 (33.1%) | ns |
| Male | 10,023 (67.4%) | 1,732 (64.0%) | | 7,780 (66.9%) | 3,975 (66.9%) | |
| Deprivation quintile | | | | | | |
| 1 - Least deprived | 2,040 (13.7%) | 395 (14.6%) | * | 1,544 (13.3%) | 891 (15.0%) | ** |
| 2 | 2,289 (15.4%) | 446 (16.5%) | | 1,767 (15.2%) | 968 (16.3%) | |
| 3 | 2,530 (17.0%) | 498 (18.4%) | | 1,954 (16.8%) | 1,074 (18.1%) | |
| 4 | 3,251 (21.9%) | 586 (21.6%) | | 2,582 (22.2%) | 1,255 (21.1%) | |
| 5 - Most deprived | 4,757 (32.0%) | 783 (28.9%) | | 3,790 (32.6%) | 1,750 (29.5%) | |
| Case definition | | | | | | |
| Primary | 7,595 (51.1%) | 1,779 (65.7%) | ** | 5,732 (49.3%) | 3,642 (61.3%) | ** |
| Uplift | 7,272 (48.9%) | 929 (34.3%) | | 5,905 (50.7%) | 2,296 (38.7%) | |
| Stage of liver disease | | | | | | |
| Fatty liver | 916 (6.2%) | 26 (1.0%) | ** | 818 (7.0%) | 124 (2.1%) | ** |
| Hepatitis | 2,148 (14.4%) | 221 (8.2%) | | 1,812 (15.6%) | 557 (9.4%) | |
| Fibrosis and sclerosis | 55 (0.4%) | 7 (0.3%) | | 48 (0.4%) | 14 (0.2%) | |
| Cirrhosis | 4,220 (28.4%) | 781 (28.8%) | | 2,998 (25.8%) | 2,003 (33.7%) | |
| Hepatic failure | 1,442 (9.7%) | 912 (33.7%) | | 1,045 (9.0%) | 1,309 (22.0%) | |
| Not specified | 6,086 (40.9%) | 761 (28.1%) | | 4,916 (42.2%) | 1,931 (32.5%) | |
| Ascites | 5,179 (34.8%) | 1,410 (52.1%) | ** | 3,580 (30.8%) | 3,009 (50.7%) | ** |
| Varices | 2,191 (14.7%) | 513 (18.9%) | * | 1,609 (13.8%) | 1,095 (18.4%) | ** |
| Comorbidity score (non-liver) | | | | | | |
| 0-1 | 13,368 (89.9%) | 2,138 (79.0%) | ** | 10,736 (92.3%) | 4,770 (80.3%) | ** |
| 2+ | 1,499 (10.1%) | 570 (21.0%) | | 901 (7.7%) | 1,168 (19.7%) | |
| Length of stay, mean (sd) | 11 (15) | 14 (16) | ** | 11 (14) | 13 (16) | ** |
| Higher level care | 773 (5.2%) | 977 (36.1%) | ** | 601 (5.2%) | 1,149 (19.3%) | ** |

* p<0.05; **p<0.001 between deaths and survivors; ns= not statistically significant.



For peer review only - http://bmjopen.bmj.com/site/about/guidelines.xhtml

**Supplementary Table S5.** Medcodes and corresponding readterms used for primary care records to identify entries relating to alcohol misuse and/or alcohol-related liver disease (ARLD). Codes for ARLD are marked with an asterix (*).

| medcode | readterm |
|---|---|
| 322 | Moderate drinker - 3-6u/day |
| 669 | Nondependent alcohol abuse, unspecified |
| 1399 | Alcohol problem drinking |
| 1618 | Heavy drinker - 7-9u/day |
| 2081 | Alcoholism |
| 2082 | Alcohol withdrawal syndrome |
| 2083 | Alcohol detoxification |
| 2084 | Alcohol dependence syndrome |
| 2925 | Alcoholic polyneuropathy |
| 3216 | Acute alcoholic hepatitis* |
| 3782 | Intoxication – alcohol |
| 3782 | Intoxication – alcohol |
| 4500 | Korsakov's alcoholic psychosis |
| 4506 | Alcoholic gastritis |
| 4743 | Alcoholic cirrhosis of liver* |
| 4915 | Alcoholic cardiomyopathy |
| 5611 | [X]Mental and behavioural disorders due to use of alcohol |
| 5740 | Acute alcoholic intoxication in alcoholism |
| 5758 | [X]Chronic alcoholism |
| 6169 | Alcohol dependence syndrome NOS |
| 6467 | [X]Alcoholic hallucinosis |
| 7123 | [V]Personal history of alcoholism |
| 7602 | Chronic alcoholic hepatitis* |
| 7692 | Patient advised about alcohol |
| 7746 | Nondependent alcohol abuse |
| 7885 | Alcoholic liver damage unspecified |
| 7943 | Alcoholic hepatitis* |
| 8030 | [V]Alcohol abuse counselling and surveillance |
| 8363 | Oesophageal varices in alcoholic cirrhosis of the liver* |
| 8388 | [V]Alcohol rehabilitation |
| 8430 | H/O: alcoholism |
| 8984 | Ethyl alcohol causing toxic effect |
| 8999 | Heavy drinker |
| 9169 | [D]Alcohol blood level excessive |
| 9489 | Under care of community alcohol team |
| 9508 | [X]Acute alcoholic drunkenness |
| 9849 | Referral to community alcohol team |
| 10161 | O/E - alcoholic breath |
| 10463 | [X]Intent self poison/exposure to alcohol |
| 10658 | HoNOS item 3 - alcohol/drug problem |

For peer review only

| | |
|---|---|
| 10691 | Alcoholic fatty liver* |
| 11106 | Korsakov's alcoholic psychosis with peripheral neuritis |
| 11140 | Advice on alcohol consumption |
| 11263 | Wood alcohol causing toxic effect |
| 11491 | Health ed. – alcohol |
| 11670 | [X]Korsakov's psychosis, alcohol induced |
| 11740 | Alcohol misuse - enhanced services administration |
| 12353 | [X]Mental & behav dis due to use alcohol: psychotic disorder |
| 12442 | Alcohol disorder monitoring |
| 12554 | Referral to community drug and alcohol team |
| 12974 | Nondependent alcohol abuse, episodic |
| 12976 | Suspect alcohol abuse – denied |
| 12977 | Very heavy drinker - >9u/day |
| 12982 | Alcohol intake above recommended sensible limits |
| 12984 | Very heavy drinker |
| 12985 | Moderate drinker |
| 13709 | Blood ethanol level |
| 14422 | Urine ethanol |
| 15503 | Accidental poisoning by other ethyl alcohol and its products |
| 16225 | Alcohol withdrawal delirium |
| 16237 | Alcoholic psychoses |
| 16587 | [V]Problems related to lifestyle alcohol use |
| 17149 | [V]Blood-alcohol and blood-drug test |
| 17259 | [X]Delirium tremens, alcohol induced |
| 17330 | Alcoholic hepatic failure* |
| 17607 | [X]Alcoholic psychosis NOS |
| 18156 | Alcoholics anonymous |
| 18252 | Accidental poisoning by alcohol, NEC |
| 18711 | Lifestyle advice regarding alcohol |
| 19217 | Alcohol causing toxic effect |
| 19401 | Binge drinker |
| 19489 | Alcohol consumption screen |
| 19494 | Hazardous alcohol use |
| 19754 | Serum ethanol level |
| 20514 | [X]Mental and behav dis due to use alcohol: withdrawal state |
| 20762 | Alcohol amnestic syndrome |
| 21624 | Episodic acute alcoholic intoxication in alcoholism |
| 21650 | Admitted to alcohol detoxification centre |
| 21713 | Alcoholic fibrosis and sclerosis of liver* |
| 21879 | [X]Mental and behav dis due to use of alcohol: harmful use |
| 22707 | Drinking problem scale |
| 23610 | Nondependent alcohol abuse, continuous |
| 23978 | [X]Evid of alcohol involv determind by level of intoxication |
| 24064 | Continuous chronic alcoholism |

For peer review only

| | |
|---|---|
| 24735 | O/E - breath - alcohol smell |
| 24984 | Alcohol-induced chronic pancreatitis |
| 25110 | Alcohol withdrawal hallucinosis |
| 26106 | Episodic chronic alcoholism |
| 26323 | [X]Alcoholic dementia NOS |
| 27342 | Alcoholic dementia NOS |
| 27518 | Hangover (alcohol) |
| 28150 | Nondependent alcohol abuse NOS |
| 28780 | [X]Alcohol addiction |
| 29691 | Aversion therapy - alcoholism |
| 30036 | [X]Poisoning/exposure, ? intent, to alcohol |
| 30162 | [X]Alcoholic paranoia |
| 30404 | Alcoholic paranoia |
| 30460 | Alcoholism counselling |
| 30604 | Alcohol-induced epilepsy |
| 30695 | Harmful alcohol use |
| 31443 | Chronic alcoholism |
| 31605 | [X]Accident poisoning/exposure to alcohol |
| 31742 | Alcoholic myopathy |
| 32850 | Alcohol use disorders identification test |
| 32927 | [X]Alcohol withdrawal-induced seizure |
| 32964 | Alcohol abuse monitoring |
| 33635 | Chronic alcoholism NOS |
| 33670 | Other alcoholic psychosis |
| 33839 | Cerebellar ataxia due to alcoholism |
| 35330 | Alcohol consumption counselling |
| 35859 | Pregnancy alcohol advice |
| 36296 | Acute alcoholic intoxication in alcoholism NOS |
| 36499 | Alcohol causing toxic effect NOS |
| 36714 | Ethanol causing toxic effect |
| 36748 | Alcoholic encephalopathy |
| 37264 | Alcohol leaflet given |
| 37691 | [X]Chronic alcoholic brain syndrome |
| 37946 | Chronic alcoholic brain syndrome |
| 38012 | Accidental poisoning by ethanol, NOS |
| 38061 | Alcohol induced hallucinations |
| 39327 | [X]Mental and behav dis due to use alcohol: dependence syndr |
| 39799 | [X]Mental and behav dis due to use alcohol: amnesic syndrome |
| 40530 | Acute alcoholic intoxication, unspecified, in alcoholism |
| 40541 | Accidental poisoning by alcoholic beverages |
| 40602 | ADS - Alcohol dependence scale |
| 41251 | BMAST - Brief Michigan alcoholism screening test |
| 41638 | [X]Int self poison/exposure to alcohol at home |
| 41920 | Alcohol amnestic syndrome NOS |

For peer review only

| | |
|---|---|
| 41983 | Alcohol detoxification |
| 42305 | Severity of alcohol dependence questionnaire |
| 43193 | Unspecified chronic alcoholism |
| 43813 | Police:venesect-alcohol |
| 44299 | [X]Mental & behav dis due to use alcohol: acute intoxication |
| 44783 | Pain in lymph nodes after alcohol consumption |
| 45169 | [X]Men & behav dis due to use alcohol: oth men & behav dis |
| 46677 | Alcohol withdrawal regime |
| 46684 | Plasma ethanol level |
| 47123 | Alcohol counselling by other agencies |
| 47555 | Cerebral degeneration due to alcoholism |
| 48514 | Denatured alcohol causing toxic effect |
| 48545 | Alcohol questionnaire completed |
| 50507 | Alcohol dependence scale |
| 53139 | Accidental poisoning by other alcohols |
| 54209 | Advice to change alcohol intake |
| 54505 | Other alcoholic dementia |
| 55415 | Accidental poisoning by alcohol NOS |
| 55536 | Ethyl alcohol causing toxic effect NOS |
| 56410 | Delivery of rehabilitation for alcohol addiction |
| 56441 | Michigan alcoholism screening test |
| 56947 | Continuous acute alcoholic intoxication in alcoholism |
| 57714 | Alcohol dependence with acute alcoholic intoxication |
| 57939 | Pathological alcohol intoxication |
| 59414 | [X]Intent self poison alcohol unspecif place |
| 59776 | Last six months of drinking questionnaire |
| 59873 | AUDIT - Alcohol use disorders identification test |
| 61187 | [X]Accid poison/expos to alcohol unspecif place |
| 61190 | [X]Pois/expos ?intent to alcohol unspecif place |
| 61583 | [V]Medicolegal blood alcohol test |
| 61750 | Comprehensive drinker profile |
| 62299 | SADD - Short alcohol dependence data |
| 62300 | Short alcohol dependence data |
| 63306 | [X]Accident poison/exposure to alcohol at home |
| 63457 | CDP - Comprehensive drinker profile |
| 63529 | Alcohol misuse - enhanced service completed |
| 63876 | [X]Accid poison/expos alcohol in street/highway |
| 64389 | [X]Ment & behav dis due use alcohol: unsp ment & behav dis |
| 65754 | Alcohol-induced pseudo-Cushing's syndrome |
| 65932 | [X]Alcoholic jealousy |
| 66831 | SADQ - Severity of alcohol dependence questionnaire |
| 67651 | Alcoholic psychosis NOS |
| 68111 | Other alcoholic psychosis NOS |
| 68159 | [X]Poison/exposure ?intent, to alcohol at home |



For peer review only - http://bmjopen.bmj.com/site/about/guidelines.xhtml

| | |
|---|---|
| 69331 | MAST - Michigan alcoholism screening test |
| 69407 | [X]Pois/exp ?intent alcohol school/pub admin area |
| 70939 | Munich alcoholism test |
| 84218 | Disqualified from driving due to excess alcohol |
| 90714 | Alcohol screen - AUDIT completed |
| 92908 | [X]Accid poison/expos alcohol trade/service area |
| 93415 | Alcohol units per week |
| 93624 | Fast alcohol screening test |
| 94485 | Alcohol screen - AUDIT PC completed |
| 94553 | Referral to specialist alcohol treatment service |
| 94669 | Alcohol consumption screening test declined |
| 94670 | Alcohol misuse |
| 94838 | Alcohol use disorders identification test |
| 94963 | Alcohol screen - fast alcohol screening test completed |
| 95181 | Alcohol reduction programme |
| 95650 | Advice to change drink intake |
| 95663 | Alcohol screen - AUDIT C completed |
| 95744 | Alcohol use disorder identificatn test consumptn questionnre |
| 95944 | Alcohol assesment declined - enhanced services admin |
| 96053 | Brief intervention for excessive alcohol consumptn completed |
| 96054 | Extended intervention for excessive alcohol consumptn complt |
| 96107 | Single alcohol screening questionnaire |
| 96219 | [X]Pois/expos ?intent alcohol in street/highway |
| 96259 | Alcohol assessment declined |
| 96993 | Referral to alcohol brief intervention service |
| 97085 | [X]Int self poison alcohol other spec place |
| 97126 | Alcohol units consumed on heaviest drinking day |
| 97163 | Advice to change alcoholic drink intake |
| 97163 | Advice to change alcoholic drink intake |
| 97261 | Brief intervention for excessive alcohol consumptn declined |
| 97309 | Advised to contact primary care alcohol worker |
| 97680 | Declined referral to specialist alcohol treatment service |
| 99877 | Feels should cut down drinking |
| 99985 | Brief Michigan alcoholism screening test |
| 100493 | Five-shot questionnaire on heavy drinking |
| 100989 | Breath alcohol level |
| 101426 | Accidental poisoning by ethyl alcohol NOS |
| 101543 | Other alcohol causing toxic effect |
| 101718 | Drinks in morning to get rid of hangover |
| 102121 | Police:venesect-alcohol |
| 102247 | Extended interven for excessive alcohol consumption declined |
| 102448 | Higher risk drinking |
| 102564 | Advised to abstain from alcohol consumption |
| 102577 | Severity of alcohol dependence questionnaire |


For peer review only - http://bmjopen.bmj.com/site/about/guidelines.xhtml

| | |
|---|---|
| 102665 | Increasing risk drinking |
| 102770 | SADQ - Severity of alcohol dependence questionnaire |
| 103459 | Referral to community alcohol team declined |
| 104458 | Breath ethanol level |
| 104611 | Alcohol-induced acute pancreatitis |
| 104702 | [X]Accid pois/expos alcohol in sport/athletic area |
| 104734 | [X]Accid pois/expos to alcohol other spec place |
| 108205 | Clinical Institute Withdrawal Assessment for Alcohol, revisd |
| 108644 | In-house alcohol detoxification |
| 109241 | Alcohol Use Disorders Identification Test declined |
| 109668 | Specialist alcohol treatment service signposted |
| 109675 | Hospital alcohol liaison team report received |
| 109800 | Hospital attendance related to personal alcohol consumption |
| 110494 | Alcohol harm reduction programme |
| 110624 | Alcohol relapse prevention |
| 110911 | Accidental poisoning by denatured alcohol |
| 111683 | Removal of alcohol |
| 111933 | Family wellbeing discussion about alcohol |
| 112448 | [X]Pois/expos ?intent to alcohol at res institut |
| 112645 | [X]Intent self pois alcohol in street/highway |
| 112648 | [X]Acc pois/expos alcohol indust/construct area |

For peer review only

Figure 3A

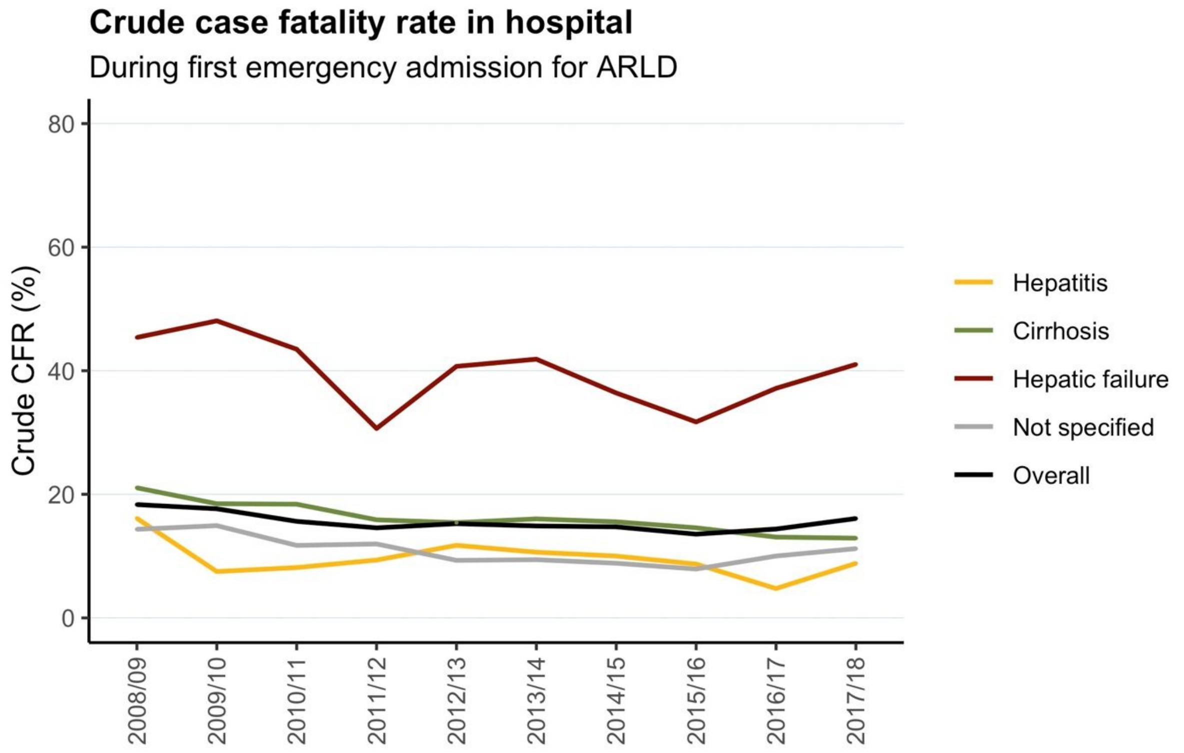


Figure 3B

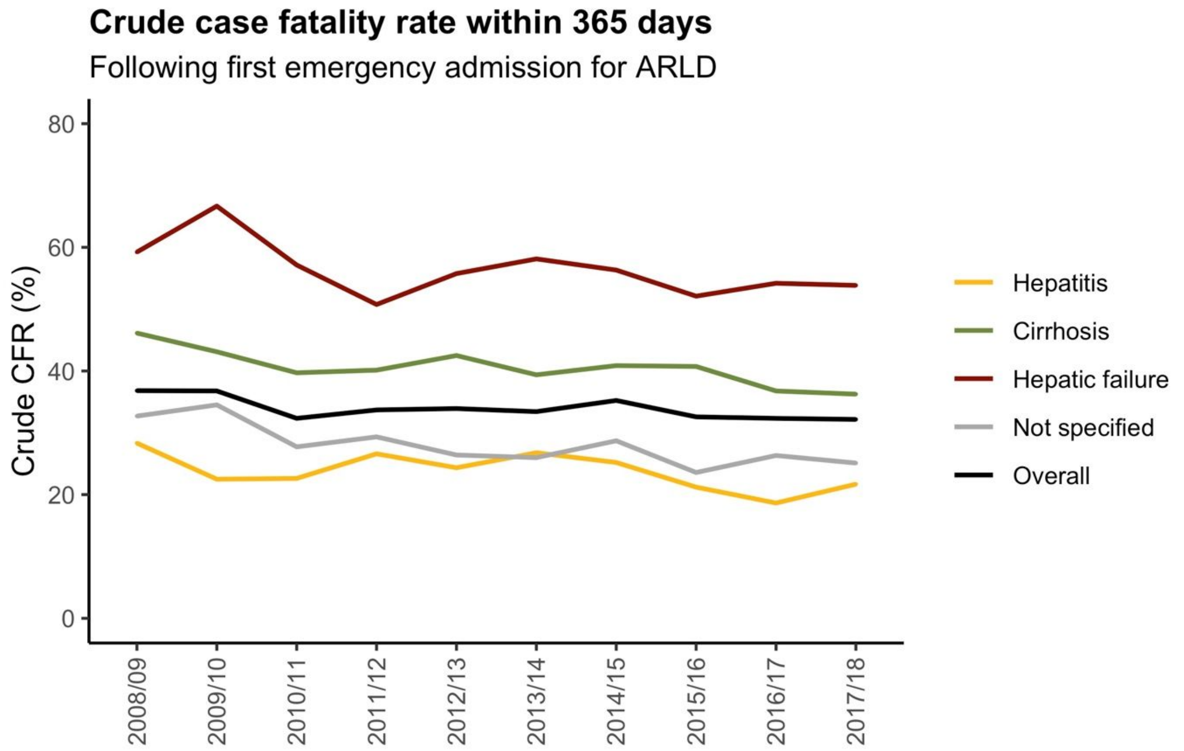



For peer review only - http://bmjopen.bmj.com/site/about/guidelines.xhtml

Figure 3C

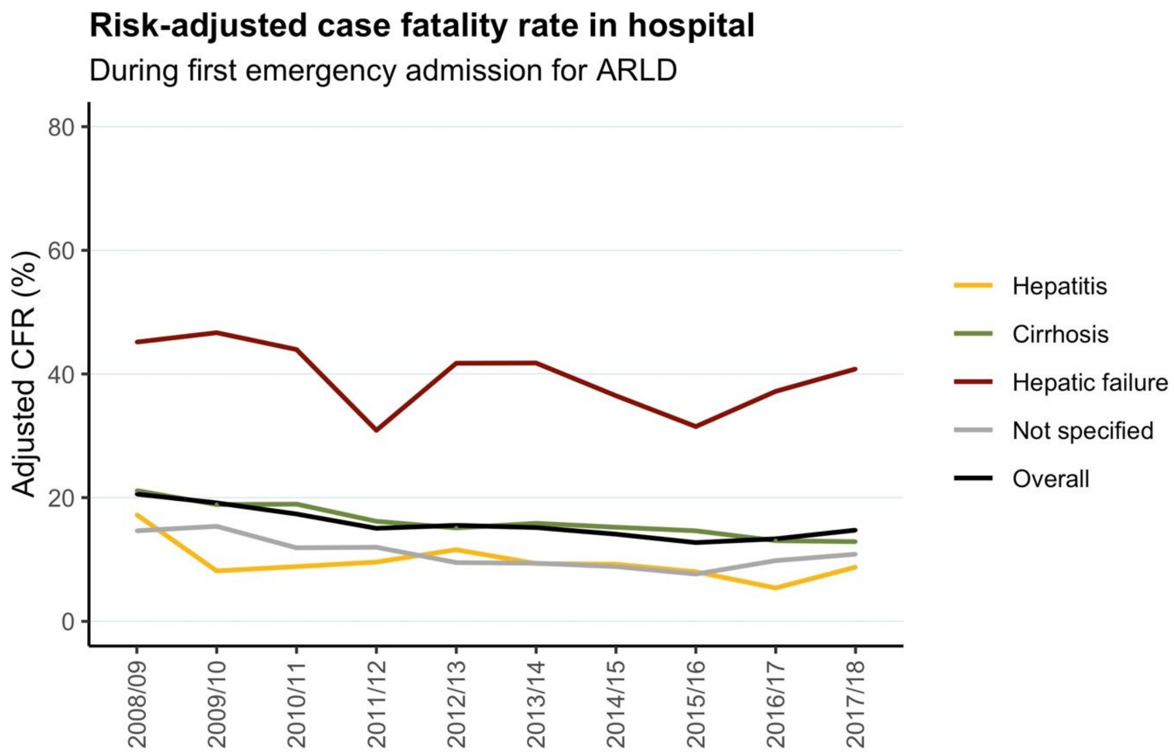


Figure 3D

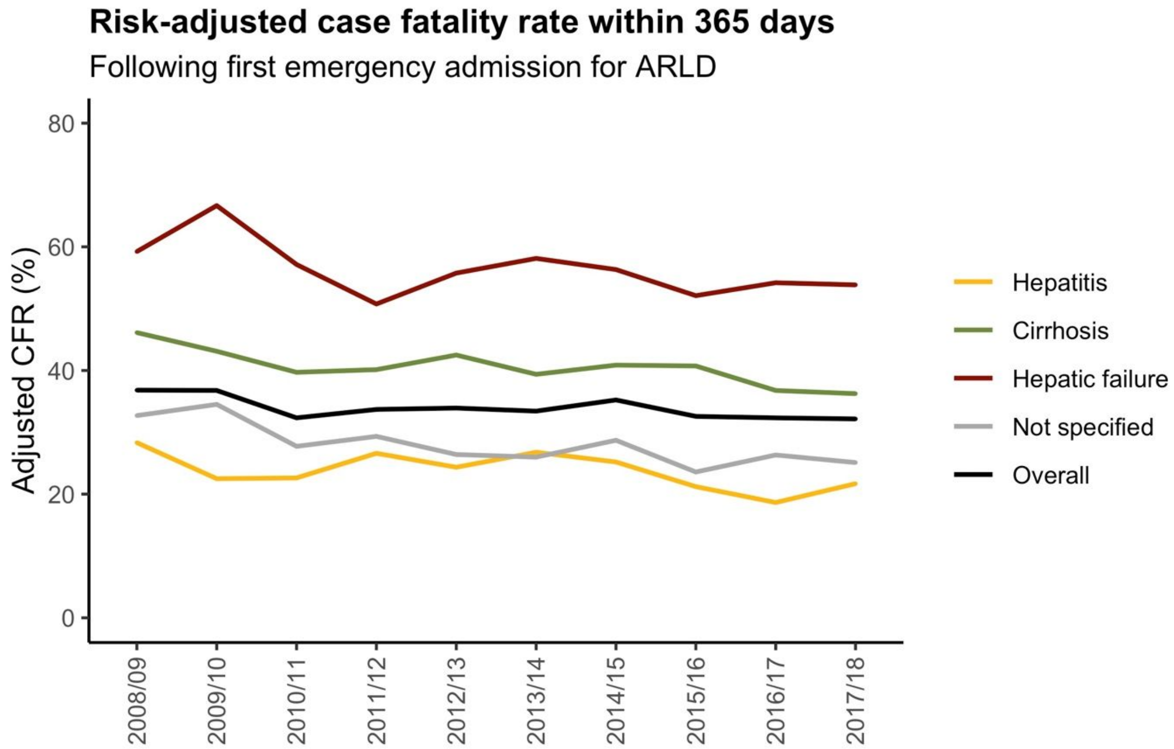

STROBE Statement—checklist of items that should be included in reports of observational studies

| | Item No | Recommendation |
|---|---|---|
| **Title and abstract** | 1 | (*a*) Indicate the study's design with a commonly used term in the title or the abstract [Done] |
| | | (*b*) Provide in the abstract an informative and balanced summary of what was done and what was found. Page 1 |
| **Introduction** | | |
| Background/rationale | 2 | Explain the scientific background and rationale for the investigation being reported. Page 3 (para 1-3) |
| Objectives | 3 | State specific objectives, including any prespecified hypotheses. Page 3 (para 4) |
| **Methods** | | |
| Study design | 4 | Present key elements of study design early in the paper. Page 3-4 |
| Setting | 5 | Describe the setting, locations, and relevant dates, including periods of recruitment, exposure, follow-up, and data collection. Page 3-4 |
| Participants | 6 | (*a*) *Cohort study*—Give the eligibility criteria, and the sources and methods of selection of participants. Describe methods of follow-up. Page 4 (para 2-3)<br>*Case-control study*—Give the eligibility criteria, and the sources and methods of case ascertainment and control selection. Give the rationale for the choice of cases and controls<br>*Cross-sectional study*—Give the eligibility criteria, and the sources and methods of selection of participants |
| | | (*b*) *Cohort study*—For matched studies, give matching criteria and number of exposed and unexposed<br>*Case-control study*—For matched studies, give matching criteria and the number of controls per case |
| Variables | 7 | Clearly define all outcomes, exposures, predictors, potential confounders, and effect modifiers. Give diagnostic criteria, if applicable Page 4-6 |
| Data sources/ measurement | 8* | For each variable of interest, give sources of data and details of methods of assessment (measurement). Describe comparability of assessment methods if there is more than one group. Page 4-5 |
| Bias | 9 | Describe any efforts to address potential sources of bias. Page 5-6 |
| Study size | 10 | Explain how the study size was arrived at. N/A all eligible cases were included |
| Quantitative variables | 11 | Explain how quantitative variables were handled in the analyses. If applicable, describe which groupings were chosen and why Page 6 |
| Statistical methods | 12 | (*a*) Describe all statistical methods, including those used to control for confounding Page 6 |
| | | (*b*) Describe any methods used to examine subgroups and interactions. Page 6 |
| | | (*c*) Explain how missing data were addressed. N/A No missing data |
| | | (*d*) *Cohort study*—If applicable, explain how loss to follow-up was addressed. N/A. Outcome was known for all cases, tracked for 365 days from admission.<br>*Case-control study*—If applicable, explain how matching of cases and controls was addressed<br>*Cross-sectional study*—If applicable, describe analytical methods taking account of sampling strategy |
| | | (*e*) Describe any sensitivity analyses. Pages 6 and 11 |

Continued on next page



For peer review only - http://bmjopen.bmj.com/site/about/guidelines.xhtml

| | | |
|---|---|---|
| **Results** | | |
| Participants | 13* | (a) Report numbers of individuals at each stage of study—eg numbers potentially eligible, examined for eligibility, confirmed eligible, included in the study, completing follow-up, and analysed. Page 7 and Table 1, Suppl. Tables 1-4 |
| | | (b) Give reasons for non-participation at each stage. N/A |
| | | (c) Consider use of a flow diagram. N/A |
| Descriptive data | 14* | (a) Give characteristics of study participants (eg demographic, clinical, social) and information on exposures and potential confounders. Page 7, Table 1, Suppl. Tables S1-S5 |
| | | (b) Indicate number of participants with missing data for each variable of interest. N/A |
| | | (c) *Cohort study*—Summarise follow-up time (eg, average and total amount). Page 5 - all cases were followed-up for 365 days post-admission (or death). Table 2 and Figure 2 |
| Outcome data | 15* | *Cohort study*—Report numbers of outcome events or summary measures over time. Page 9-10, Table 1, Figure 2, Suppl. Fig. 1, Suppl. Figs S2-S4 |
| | | *Case-control study*—Report numbers in each exposure category, or summary measures of exposure |
| | | *Cross-sectional study*—Report numbers of outcome events or summary measures |
| Main results | 16 | (*a*) Give unadjusted estimates and, if applicable, confounder-adjusted estimates and their precision (eg, 95% confidence interval). Make clear which confounders were adjusted for and why they were included. Page 10, Table 2, Suppl. Fig S1 |
| | | (*b*) Report category boundaries when continuous variables were categorized |
| | | (*c*) If relevant, consider translating estimates of relative risk into absolute risk for a meaningful time period. N/A |
| Other analyses | 17 | Report other analyses done—eg analyses of subgroups and interactions, and sensitivity analyses. Page 11 |
| **Discussion** | | |
| Key results | 18 | Summarise key results with reference to study objectives. Page 11 |
| Limitations | 19 | Discuss limitations of the study, taking into account sources of potential bias or imprecision. Discuss both direction and magnitude of any potential bias. Page 12-13 |
| Interpretation | 20 | Give a cautious overall interpretation of results considering objectives, limitations, multiplicity of analyses, results from similar studies, and other relevant evidence. Page 11-12 |
| Generalisability | 21 | Discuss the generalisability (external validity) of the study results. Page 13, para 2 |
| **Other information** | | |
| Funding | 22 | Give the source of funding and the role of the funders for the present study and, if applicable, for the original study on which the present article is based. Page 13 |

*Give information separately for cases and controls in case-control studies and, if applicable, for exposed and unexposed groups in cohort and cross-sectional studies.

**Note:** An Explanation and Elaboration article discusses each checklist item and gives methodological background and published examples of transparent reporting. The STROBE checklist is best used in conjunction with this article (freely available on the Web sites of PLoS Medicine at http://www.plosmedicine.org/, Annals of Internal Medicine at http://www.annals.org/, and Epidemiology at http://www.epidem.com/). Information on the STROBE Initiative is available at www.strobe-statement.org.

For peer review only

# Outcomes of first emergency admissions for alcohol-related liver disease in England over a ten year period: Retrospective observational cohort study using linked electronic databases


| | |
|---|---|
| Journal: | *BMJ Open* |
| Manuscript ID | bmjopen-2023-076955.R1 |
| Article Type: | Original research |
| Date Submitted by the Author: | 06-Oct-2023 |


| | |
|---|---|
| Complete List of Authors: | Bodger, Keith; University of Liverpool, Dept. of Health Data Science, Insitute of Population Health; Liverpool University Hospitals NHS Foundation Trust, Gastroenterology Dept.<br>Mair, Thomas; University of Liverpool, Dept. of Health Data Science, Insitute of Population Health<br>Schofield, Peità; University of Liverpool, Department of Public Health & Policy<br>Silberberg, Benjamin; University of Liverpool, Dept. of Health Data Science, Insitute of Population Health<br>Hood, Steve; Liverpool University Hospitals NHS Foundation Trust, Gastroenterology Dept.<br>Fleming, Kate M; NHS England, Data & Analytics Transformation Directorate |


| | |
|---|---|
| <b>Primary Subject Heading</b>: | Gastroenterology and hepatology |
| Secondary Subject Heading: | Gastroenterology and hepatology |
| Keywords: | Electronic Health Records, Hospitalization, EPIDEMIOLOGY, Hepatology < INTERNAL MEDICINE, Primary Care < Primary Health Care |
| | |


SCHOLARONE™
Manuscripts

*I, the Submitting Author has the right to grant and does grant on behalf of all authors of the Work (as defined in the below author licence), an exclusive licence and/or a non-exclusive licence for contributions from authors who are: i) UK Crown employees; ii) where BMJ has agreed a CC-BY licence shall apply, and/or iii) in accordance with the terms applicable for US Federal Government officers or employees acting as part of their official duties; on a worldwide, perpetual, irrevocable, royalty-free basis to BMJ Publishing Group Ltd ("BMJ") its licensees and where the relevant Journal is co-owned by BMJ to the co-owners of the Journal, to publish the Work in this journal and any other BMJ products and to exploit all rights, as set out in our licence.*

*The Submitting Author accepts and understands that any supply made under these terms is made by BMJ to the Submitting Author unless you are acting as an employee on behalf of your employer or a postgraduate student of an affiliated institution which is paying any applicable article publishing charge ("APC") for Open Access articles. Where the Submitting Author wishes to make the Work available on an Open Access basis (and intends to pay the relevant APC), the terms of reuse of such Open Access shall be governed by a Creative Commons licence – details of these licences and which Creative Commons licence will apply to this Work are set out in our licence referred to above.*

*Other than as permitted in any relevant BMJ Author's Self Archiving Policies, I confirm this Work has not been accepted for publication elsewhere, is not being considered for publication elsewhere and does not duplicate material already published. I confirm all authors consent to publication of this Work and authorise the granting of this licence.*

For peer review only

# Outcomes of first emergency admissions for alcohol-related liver disease in England over a ten year period: Retrospective observational cohort study using linked electronic databases

Keith Bodger[1,3], Thomas Mair[1], Pieta Schofield[2], Benjamin Silberberg[1], Steve Hood[3], Kate M Fleming[4]

1. Dept. of Health Data Science, Insitute of Population Health, University of Liverpool, UK
2. Dept. of Public Health & Policy, Insitute of Population Health, University of Liverpool, UK
3. Gastroenterology Dept., Liverpool University Hospitals NHS Foundation Trust, UK
4. National Disease Registration Service, Data & Analytics Transformation Directorate, NHS England, UK

## Abstract

**Objectives:** To examine time trends in patient characteristics, care processes and case fatality of first emergency admission for alcohol-related liver disease (ARLD) in England.

**Design:** National population-based, retrospective observational cohort study.

**Setting:** Clinical Practice Research Datalink population of England, 2008/9-17/18. First emergency admissions were identified using the Liverpool ARLD Algorithm. We applied survival analyses and binary logistic regression to study prognostic trends.

**Outcome measures:** Patient characteristics; "recent" GP consultations and hospital admissions (preceding year); higher level care; deaths in-hospital (including certified cause) and within 365 days. Covariates were age, sex, deprivation status, coding pattern, ARLD stage, non-liver comorbidity, coding for ascites and varices.

**Results:** 17,575 first admissions (mean age: 53 years; 33% female; 32% from most deprived quintile). Almost half had codes suggesting advanced liver disease. In year before admission, only 47% of GP consulters had alcohol-related problems recorded; alcohol-specific diagnostic codes were absent in 24% of recent admission records. Overall, case fatality rate was 15% in-hospital and 34% at one year. Case-mix-adjusted odds of in-hospital death reduced by 6% per year (aOR: 0.94; 95% CI: 0.93-0.96) and 4% per year at 365 days (aOR: 0.96; 95% CI: 0.95-0.97). Exploratory analyses suggested the possibility of regional inequalities in outcome.

**Conclusions:** Despite improving prognosis of first admissions, we found missed opportunities for earlier recognition and intervention in primary and secondary care. In 2017/18, one in seven were still dying during index admission, rising to one third within a year. Nationwide efforts are needed to promote earlier detection and intervention, and to minimise avoidable mortality after first emergency presentation. Regional variation requires further investigation.

**Keywords:** Liver Diseases, Alcoholic; Algorithms; Epidemiology; Primary Health Care; Electronic Health Records; Hospitalization; Hospital Mortality; Emergency Service; Quality of Health Care

**Corresponding author:** Keith Bodger, Reader & Consultant Gastroenterologist (Honorary), Department of Health Data Science, Institute of Population Health, University of Liverpool, kbodger@liverpool.ac.uk



For peer review only - http://bmjopen.bmj.com/site/about/guidelines.xhtml

## STRENGTHS AND LIMITATIONS OF THIS STUDY

- Nationwide population-based study sample covering all regions of England
- Application of a novel cohort discovery algorithm and the use of linked datasets covering primary and secondary care and death certification
- The datasets lack some important case mix information such as physiological or biochemical measures of disease severity which would allow for further risk-adjustment of case fatality rates.
- The cases are drawn from a large, representative sample of the English population but the findings may not be generalisable to all parts of the country.

For peer review only



For peer review only - http://bmjopen.bmj.com/site/about/guidelines.xhtml

## Introduction

Alcohol-related liver disease (ARLD) is a leading cause of premature death and is set to overtake ischaemic heart disease for working years-of-life-lost in the UK.(1) Major shortcomings in acute hospital treatment were highlighted by the National Confidential Enquiry into Patient Outcome and Death (NCEPOD).(2) This widely publicised enquiry, combined with reports from the *Lancet Standing Commission* on Liver Disease,(3) prompted UK initiatives to promote better care.(4–8) Similar calls for action have been raised in other countries.(9) However, in their final report the commission's authors highlighted continuing concerns about late diagnosis and unacceptably high levels of mortality in hospitalised patients.(10)

A patient's first emergency hospital admission is often the point of initial diagnosis.(11) High quality care is crucial to minimise early mortality and allow time for engagement of services promoting abstinence and recovery. However, knowledge of characteristics and outcomes of first admissions is limited. Nor is it known whether case-mix has changed or prognosis improved over time.

Hospital administrative data is the main source of national statistics (12) and underpins proposed metrics.(13) However, identifying and characterising cases using routine data is challenging.(11) Patients present to hospital with varied clinical manifestations and co-existing alcohol-related conditions.(14) Analysis of administrative data in our region revealed that standard case definitions (based on primary discharge diagnoses alone)(12) identified only half of true admissions.(11) We developed an algorithm to identify combinations of primary and secondary codes compatible with acute presentations of ARLD.(11) Recent national testing confirmed a doubling of estimates of annual admissions using this method,(15) representing an under-recording of NHS hospital activity of £70-127 million for 2020.(15) This prompted calls for revising methods for national statistics.(16)

We undertook a retrospective cohort study using linked electronic datasets and the Liverpool ARLD algorithm (LAA).(11,15) We aimed to describe sociodemographics, clinical features, care processes (prior healthcare contacts in preceding year, length of stay and provision of higher level care) and case fatality of patients presenting with their first emergency admission for ARLD in England. We examined trends over a ten year period, spanning five years before and after the NCEPOD report.(2) We also undertook exploratory analyses of regional variation in admission outcome.

## Methods

### Data Sources

The cohort was derived from the Clinical Practice Research Datalink (CPRD) ~~which~~ containing linked data from primary and secondary care and death certification (Office for National Statistics).(17) This anonymized database covers a broadly representative


For peer review only - http://bmjopen.bmj.com/site/about/guidelines.xhtml

sample of UK residents. The primary care consultations records contain diagnoses encoded as READ codes. Each case in the present study had linkage to Hospital Episode Statistics (HES), comprising administrative data for inpatient care delivered in NHS hospitals (available for the period April 1997 to Mark 2021). Care episodes in HES contain diagnostic codes generated after discharge using the international classification of diseases (ICD-10) system. The dataset also included the Adult Critical Care Minimum dataset.

**Population of interest: First emergency admissions for ARLD**

We extracted data for all adult patients (≥18 years) with a first unplanned (non-elective) hospital admission (spell) for ARLD. We defined a "first" (or index) admission as having no such admission within the preceding ten years. We included all completed discharge records between 1st April 2008 and 31st March 2018, thereby covering ten fiscal years (2008/9 to 2017/18). The cohort was restricted to residents with a record of CPRD practice registration ≥1 year.

**Case definitions for ARLD admissions**

We used the LAA to identify admissions, requiring discharge codes to comply with one of two alterntive case definitions ('primary' or 'uplift' subgroups).(11) The primary definition selects cases with a K70.x code as primary diagnosis, as in national statistics.(12) The 'uplift' definition identifies additional admissions with non-primary coding patterns (11,15) Briefly, uplift cases have a primary code for either a symptom, sign or complication of liver disease or for another alcohol-specific condition, accompanied by secondary codes compatible with ARLD (either K70.x or code for unspecified liver disease provided additional alcohol-specific conditions are listed).

**Stage of liver disease**

We further characterised cases by assigning "recorded-stage" of liver disease based on the K70.x code present in the index admission (i.e. alcoholic fatty liver [K70.0], hepatitis [K70.1], sclerosis and fibrosis [K70.2], cirrhosis [K70.3], hepatic failure [K70.4] or "unspecified" [K70.9]). All primary and most uplift admission records contain a K70.x code. A minority of uplift cases lack a K70.x code, whereby a non-specific liver disease code (e.g. "Hepatic failure, unspecified" or "Other and unspecified cirrhosis of liver") is accompanied by codes for other alcohol-specific conditions. In these cases, the relevant stage was taken from the liver code descriptor.

**Patient-level covariates derived from the index admission**

Covariates were age, age group, sex, ethnicity, deprivation status, case definition (primary or uplift), recorded-stage of liver disease, coding for specific features of advanced liver disease (varices and ascites) and comorbidities. Socioeconomic deprivation was defined by the English indices of deprivation 2015 and grouped into quintiles (1 = Least deprived, 5 = Most deprived), as described previously.(11,18) Comorbidities listed in the Charlson Comorbidity Index (CCI)(19) were identified but



For peer review only - http://bmjopen.bmj.com/site/about/guidelines.xhtml

we excluded liver disease codes (mild and moderate-to-severe liver disease categegories) and dichotomised cases into two groups (scores 0-1 and ≥2).

**Contacts in the year prior to index admission**

The earliest stage of ARLD (fatty liver) may develop over months but more severe liver disease (hepatitis, cirrhosis or liver failure) requiring hospitalisation requires several years of harmful drinking.(14,20) We focused on contacts within a year before index emergency admission, assuming all such patients would have an established history of alcohol excess. The absence of relevant codes within such encounters would be taken as a marker of "missed opportunities" to screen, detect and/or intervene during the year before liver-related emergency hospitalization.

**Consultations in primary care** - All available primary care contacts within the prior year were extracted and screened. For each contact, we created two binary variables. Firstly, a variable to indicate whether any alcohol-related code was present – signifying whether alcohol misuse or harms were suspected/recognised and recorded at the time. We used an extensive list of READ codes (See **Supplementary Appendix 1**). Second, whether a diagnosis of liver disease was recorded – a marker of prior suspicion, recognition or elective diagnosis of ARLD.

**Emergency hospital admissions** - We identified all episodes of unplanned hospital care in the year prior to index admission and screened the diagnosis fields for any alcohol-specific ICD-10 code. Hence, we determined whether any non-liver related alcohol problems were diagnosed and recorded during those prior admissions.

**Care processes**

We determined length of hospital stay. Using the Adult Critical Care Minimum Dataset we determined whether the patient received higher level care during their index admission, including high dependency (category 2) and intensive care (category 3).

**Time periods of interest**

We defined two 5-year time periods to compare cases discharged in the first and second half of the decade (Period 1: April 2008 to March 2013; Period 2: April 2013 to March 2018). We also created a variable to indicate fiscal year of discharge.

**Outcomes**

We examined all-cause case fatality identified from death certification. We created binary variables for death during index admission (in-hospital death) and tracked all cases for death within 365-days. For survival analysis we calculated time-to-death (days from admission). We used case fatality rates, as opposed to population-based relative mortality measures, since our purpose was to generate metrics focused on effectiveness of acute care after adjusting for severity of presenting illness.



For peer review only - http://bmjopen.bmj.com/site/about/guidelines.xhtml

**Certified cause of in-hospital death**
We accessed linked records of death certification for all in-hospital deaths during index admissions to identify deaths attributable to ARLD. This was based on screening all available "cause" fields for descriptors relating to deaths attributable to liver disease and alcohol. See **Supplementary Appendix 2** for details.

**Statistical analyses**
First, we summarised characteristics overall and for relevant strata and examined time trends. Categorical variables are reported as counts and percentages (groups compared using Chi Squared statistics). Continuous variables are reported as mean (sd), or median (IQR), as appropriate with comparisons made using parametric (t-test or ANOVA) or non-parametric tests (Mann-Whitney U or Kruskal-Wallis).

Second, we calculated crude case fatality rates (CFR) as count of deaths (numerator) divided by number of first admissions (denominator) per time period. We report deaths in-hospital and at 365 days. We compared 5-year periods (Chi-square Test) and significance of monotonic year-on-year trends (Mann Kendall Trend Test). Kaplan-Meier survival analysis compared cumulative probability of death up to 365 days, using log-rank test to determine significance between strata. All cases had at least one year follow-up (no censoring).

Third, we determined factors associated with death using binary logistic regression. Models were used to adjust CFR for significant covariates. The dependent variables were all-cause death (either in-hospital or at 365 days). Covariates were added sequentially based on clinical relevance, previous research(11) and significant univariate associations ($p<0.10$), starting with age and sex and building a series of models to explore alternative risk-adjustment approaches. We generated time series of risk-adjusted CFRs by calculating standardised mortality ratios (SMR) for each time period, dividing the number of observed deaths by the number of expected deaths (sum of probabilities from relevant model). Each model used pooled data across the ten year period to allow comparisons over time. Risk-adjusted rates were calculated by multiplying the crude rate across 10 years by the SMR for each period (equivalent to indirect standardisation). The C-statistic and AIC values were calculated to assess model fit.

Fourth, we tested significance of national time trends by introducing five-year period or fiscal year of discharge into models. For year-on-year trends, the year variable was treated as continuous, hence odds ratios reflect average change *per one-year increment* across the decade (assuming a linear trend). Sensitivity analyses were undertaken using variants of model covariates (continuous rather than categorical variables for age and non-liver comorbidity score; recorded-stage of liver disease rather than binary variable).


For peer review only - http://bmjopen.bmj.com/site/about/guidelines.xhtml

Finally, we undertook exploratory analysis of regional variation focusing on the main outcome of in-hospital case fatality. CPRD region of residence was based on Strategic Health Authority boundaries. We used Funnel Plots to evaluate variation in crude rates and added region to models to compare risk-adjusted CFR.

All analyses were undertaken in R (Version 4.1.2, R Core Team 2021, R Foundation for statistical computing, Vienna, Austria)

**Data Governance and Ethics Statements**

The CPRD has obtained ethical approval from the UK's National Research Ethics Service (NRES) Committee for observational research using anonymised data. Our study was approved by the Independent Scientific Advisory Committee (ISAC) for Medicines and Healthcare Products Regulatory Agency (MHRA) database research (protocol number 19_133). Patient consent for publication is not applicable.

## Results

**Characteristics of first emergency admissions**

The algorithm detected 17,575 index admissions with characteristics summarised in **Table 1** (overall, and by case definition). 9,374 (53%) were in the primary subgroup and 8,201 (47%) in the uplift subgroup (non-primary coding patterns). The magnitude of 'uplift' is consistent with previous findings.(11,15) The mean age was 53.4 years (range: 18-98), one third (33.1%) were female, 32% were residents of most deprived quintile. Less than 5% had non-white ethnicity recorded (small numbers precluded more detailed reporting).

**Prior primary care consultations** – 9,936 patients had a record of ≥1 GP consultation in the year before admission (**Table 1**). Of this subgroup, 4,702 (47.3%) had a READ code relating to either alcohol consumption or any alcohol-specific condition recorded at that time, suggesting harmful drinking remained undetected or unrecorded in just over half of those encounters. Only 1,465 (14.7% of 'recent' GP consulters) had a code relevant to liver disease recorded at the time, suggesting that ARLD usually remained unsuspected or undiagnosed during these consultations.

**Prior emergency admissions** – forty one percent of the cohort (7,265 patients) had been admitted as an emergency in preceding year. Of these, 5,525 (76%) had a non-liver-related, alcohol-specific condition recorded in their list of discharge diagnoses. Hence, one in four (24%) admitted cases had no record of an alcohol-related diagnosis in their preceding emergency admission. This was equally true of cases with cirrhosis or liver failure at index admission, suggesting serious alcohol-related harm may have gone unrecognised (**Table 1**).


For peer review only - http://bmjopen.bmj.com/site/about/guidelines.xhtml

**Stage of liver disease** – stratification by recorded-stage is shown in **Figure 1** and **Supplementary Table S1**. These data demonstrate the high proportion of advanced liver disease recorded at index hospitalisation (cirrhosis: 28%; hepatic failure: 13%). We found frequent use of K70.9 (alcoholic liver disease, unspecified) to encode first admissions, whereby disease stage was not specified in 39%. The presence of additional codes for ascites (31%) and varices (12%) in many such cases suggests a significant proportion had severe disease (**Supplementary Table S1**).

Older groups had lower proportions with alcoholic hepatitis and a higher proportion with cirrhosis, as would be expected (**Figure 1A**). The proportion with "severe liver disease" codes (cirrhosis or hepatic failure) was similar among men and women despite statistical differences overall (**Figure 1B**). Interestingly, the proportion of first admissions with severe disease showed limited variation across deprivation quintiles and was slightly lower among those from most deprived areas (**Figure 1C**).

**Time trends in case-mix –** we found small differences in sociodemographics between patients discharged in Period 2 compared with Period 1 (**Supplementary Table S2**). Mean age was one year older (54.1 versus 52.5 years, $p<0.001$) and there was a slight reduction in proportion from most deprived areas (30% vs. 33%, $p<0.001$). There was an increased contribution of cirrhosis (31% vs. 25%, $p<0.001$) and hepatic failure (15% vs. 11%, $p<0.05$) but a reduction in unspecified stage (34% vs. 46%, $p<0.001$) (**Fig. 1D**). There was also a slight increase in proportion with codes for varices (16% vs. 15%, $p=0.031$). Levels of non-liver comorbidity were also higher (13% vs. 10% with score ≥2, $p<0.001$). Collectively, these findings suggest patients at index admission were presenting with somewhat more advanced disease and greater burden of comorbidities.

Among patients with prior GP contacts (preceding year), we found no change in the proportion of consultations with alcohol-related READ codes (46.6% vs. 48.1%, $p=0.13$) and a small reduction in liver disease codes in the final period (13.7% vs. 16.0%, $p<0.001$). There was no change in recording of alcohol-specific codes in prior emergency admissions (75.6% vs. 76.7%, $p=0.3$).

The characteristics of patients discharged within each fiscal year are summarised in **Suppl. Table S3.**

**Care processes during first admission**

Overall mean length of stay was 12 days (**Table 1**), with higher duration for hepatic failure (16 days, $p<0.001$). Ten percent received higher level care (21% of those coded as hepatic failure). Length of stay was one day shorter in the second half of the decade (11 days versus 12 days, $p<0.001$, **Supplementary Table S2**), and a significantly greater proportion received higher level care (11% versus 8.6%, $p<0.001$).


For peer review only - http://bmjopen.bmj.com/site/about/guidelines.xhtml

## Deaths following first admission

Overall, 2,708 deaths were recorded giving a crude case fatality rate (CFR) of 15.4% (**Table 1**). Median time to in-hospital death was nine days (mean: 14). One-in-five deaths occurred before day three of admission. Thirty nine percent of cases with a recorded-stage of hepatic failure died in hospital. Within 365 days of admission the cumulative death toll rose to 5,938 patients (34%).

Of the total in-hospital deaths, 2,569 (95%) had descriptors listed in one or more fields that were consistent with an ARLD-specific cause. The commonest descriptor was "alcoholic liver disease, unspecified" (**Supplementary Appendix 2**). Of these deaths, 1,738 (66.8%) were in the primary group and 831 (32.2%) in the uplift group.

Among patients with GP consultations in preceding year (n=9,936), those dying in hospital were less likely to have had coding of alcohol misuse or harms (39.3% vs 48.8%, p<0.01) or liver disease (10.1% vs 15.5%, p<0.05) than survivors. Patients with fatal admission were less likely to have had an emergency hospitalization in the past year than survivors (35.9% vs 42.2%, p<0.01). However, among those previously hospitalized, alcohol-specific diagnoses were recorded less often in those subsequently dying than survivors (68.7% vs 77.2%, p<0.01). Only 36.1% of in-hospital deaths received higher level care.

## Factors associated with death

Characteristics of patients who had died, or survived, at the two study end-points are shown in **Suppl. Table S4**. In bivariate analyses we found significant associations with increasing age, female sex, primary (as opposed to uplift) case definition, increased non-liver comorbidity, advanced recorded-stage of liver disease and coding for ascites or varices. Stratified survival analyses confirmed these bivariate associations (**Suppl. Fig. 1**). Multivariable models confirmed independent associations between the selected case-mix factors and odds of death (**Table 2**). Interestingly, deprivation status (IMD quintile) was not independently associated with outcomes and not retained in models – suggesting severity of illness at presentation dominated prognosis. Given the high prevalence of unspecified stage we used a binary variable for recorded-stage (Liver failure or Not) for final risk-adjustment.

## Time trends in case fatality

***Five-year periods*** – Comparison of unadjusted outcomes between Period 1 and Period 2 revealed lower crude in-hospital CFR (14.8% vs 16.2%, p=0.018). This did not quite reach significance at 365 days (33.2% vs 35.4%, p=0.063). Unadjusted rates of in-hospital death were numerically lower for each recorded-stage but not all comparisons were significant (hepatitis: 8.9% vs 9.9%, p=0.4; cirrhosis: 14.4% vs 17.7%, p=0.002; hepatic failure: 37.4% vs 41.2%, p=0.07; not specified: 9.7% vs 12.5%, p<0.001). Similarly, reductions in crude CFRs at 365 days did not all reach significance (hepatitis: 22.8% vs 24.5%, p=0.4; cirrhosis: 38.9% vs 41.9%, p=0.035; hepatic failure:



For peer review only - http://bmjopen.bmj.com/site/about/guidelines.xhtml

54.5% vs 57.7%, p=0.14; not specified: 26.0% vs 30.3%, p<0.001). Survival probabilities during the year after index admission were consistently higher for those discharged in Period 2– period for the cohort as a whole and by recorded stage (**Fig. 2)**. After adjusting for case-mix factors in relevant models, the adjusted odds of both in-hospital and 365 day death were significantly lower for those discharged in Period 2 (**Table 2**).

***Year-to-year trends*** – Time series are shown in **Figure 3**. Overall, crude in-hospital CFR declined from 18.3% in 2008/9 to 16.1% in 2017/18 (p=0.03) (**Figure 3A**). Corresponding changes in stage-specific crude rates were 45.4% to 41.0% for hepatic failure (p=0.13), 21.1% to 13.0% for cirrhosis (p=0.0006) and 16.1% to 8.8% for alcoholic hepatitis (p=0.47). Case-mix-adjusted rates for in-hospital death showed significant reductions overall and in stage-specific analyses (**Figure 3B** and **Table 2**). In the final risk-adjusted model (**Table 2)**, each one-year increment across the decade was associated (on average) with a 6% reduction in odds of in-hospital death (aOR: 0.94 [95% CI: 0.93-0.96], p<0.001). Stage-specific reductions in odds of dying during first admission were 3% per year for hepatic failure (p=0.004), 6% for cirrhosis (p<0.001) and 7% for alcoholic hepatitis (p<0.01).

Corresponding trends for 365-day death are shown (**Figure 3C** and **Figure 3D)**. Overall crude rate reduced from 36.8% to 32.2% (p=0.02) and stage-specific crude rates showed downward trends – hepatic failure reducing from 59.2% to 53.9% (p=0.07), cirrhosis from 46.1% to 36.3% (p=0.02) and alcoholic hepatitis from 28.3% to 21.7% (p=0.15). After case-mix adjustment, each one-year increment was associated with a 4% reduction in odds of dying within a year of admission overall (aOR: 0.96 [95% CI: 0.95-0.97], p<0.001). Stage-specific reductions in adjusted odds of death were also 4% per year for hepatic failure (p=0.011), cirrhosis (p<0.001) and alcoholic hepatitis (p=0.027).

**Sensitivity analyses**

Key findings were unchanged in terms of the significance or magnitude of trends when risk adjustment models were fitted using variants of patient-level covariates (e.g. continuous variables for age or comorbidity scores, individual categories for each stage of liver disease) (**Supplementary Figure S2**).

**Regional variations in case fatality rate of first admissions**

**Supplementary Table S5** shows distribution of cases and case-mix across the ten regions of England. Crude CFR varied from 13.4% (London) to 20.1% (South East Coast). Funnel charts identified two regions as potential "outliers" (i.e. higher than the average) on the basis of unadjusted rates – the West Midlands and South East Coast (**Supplementary Figure 3**) – whereas London had a lower-than-average crude rate. When region was added to relevant models (**Supplementary Table S6**), these two parts of the country had a significantly increased odds of in-hospital death relative to

For peer review only

London, with the West Midlands having an odds ratio of 1.27 (95% CI: 1.09 – 1.49) and South East Coast an odds ratio of 1.36 (95% CI: 1.12 – 1.66). There was no significant difference between the other regions. Overall national time trends were not altered by including region in the five-year and year-to-year models.

## Discussion

Our study represents the first detailed description of characteristics and outcomes of index emergency admissions in the English population. The predominance of males and disproportionate contribution of people living in more deprived areas is consistent with known epidemiological patterns.(1) These admissions encompass diverse manifestations of liver disease and alcohol misuse, ranging from early-stage liver disease to rapidly fatal liver failure. However, at least half had advanced disease.

Our data suggest ARLD is frequently diagnosed for the first time during unplanned hospitalization. Only 14.7% of cases with GP contacts in the year before admission had liver disease recorded during those encounters, suggesting underlying liver problems were not suspected or diagnosed at that time. A previous study examined the earliest instance of coding for cirrhosis (regardless of aetiology) within linked CPRD-HES records (1998-2009), reporting this occurred first within an emergency record in half the cases.(21) We examined recent GP consultations for codes relating to alcohol excess, finding them absent in half. These data imply missed opportunities to screen for, or detect, alcohol-related harms at a point when patients would be expected to have had well-established harmful drinking.

Similar issues were apparent in secondary care with alcohol-related codes absent from almost a quarter of unplanned hospitalisations in preceding year. The corresponding figure was 31.3% for patients who subsequently died in-hospital. It seems unlikely such patients lacked evidence of alcohol-specific conditions during those prior admissions. Across the decade we found no improvement in rates of recognition/recording of alcohol-related problems in primary or secondary care contacts during the year before index hospitalisation. Deficiencies in early detection ~~of~~ have been recognised internationally.(22)

We found improvements in short and medium term prognosis of first admissions over time but the magnitude was small. In 2017/18, one in seven were still dying during their first hospitalisation, rising to one third within a year. Whilst survival was better in the post-NCEPOD era, yearly time series suggested no step-change or accelerated reduction in case fatality during this sustained period of national initiatives.(2–5,7) Directly comparable statistics for other countries are lacking.

Only a third of in-hospital deaths (36%) had received higher level care. This is comparable to 39% in the NCEPOD review where 31.2% of those deemed eligible (by reviewers) for higher level care didn't receive it.(2) This prompted recommendations

For peer review only

for improved decision-making and access. Universal prognostic pessimism about organ support in severe liver disease has been challenged.(23) We cannot judge suitability of cases for escalation in our study but we did find a small increase in the proportion receiving such care over a ten year period.

Exploratory analyses suggest two regions had higher crude case fatality rates overall and differences persisted after risk-adjustment. However, most regions were comparable on these measures. Caution is needed when interpreting local-level outcome metrics derived from associations in national data (constant-risk fallacy). Geographical variation in population-based mortality rates (as opposed to CFRs) for liver disease are well-recognised but further exploration of possible inequalities in outcome of first admissions *per se* is merited.(24)

We confirm limitations of simple case definitions for defining workload and outcome.(11,15,16,21) Recent studies of international burden of "decompensated cirrhosis" continue to rely on primary discharge diagnosis.(25) Our analysis of deaths shows that many of the sickest patients had a non-primary coding pattern. Our findings on "recorded-stage" question the merits of defining "severe" liver disease based solely on primary codes (cirrhosis or liver failure).(26) Unspecified stage accounted for 28% of admissions and one in four deaths. If administrative data are to be used to quantify burden, evaluate interventions and benchmark performance then case identification and risk-adjustment requires care.(16) The LAA-based approach,(11,15) combined with case-mix classification reported here, could support development of improved metrics as recommended by the *Lancet* commission and in a recent NCEPOD update.(13,27) Existing statistics require caveats for interpretation.(24)

Our study has several limitations. First, the available data allowed us to define "first" emergency admission based on an admission-free interval of ten years. We cannot rule out inclusion of patients admitted over a decade earlier who then avoided any subsequent emergency readmission or death. Given the prognosis and typical trajectory of ARLD, we believe such cases are rare.

Second, HES data lacks true disease severity measures (e.g. clinical scores or laboratory parameters), limiting risk-adjustment. However, we applied both recorded-stage and condition-specific markers (ascites and varices) in addition to the usual generic covariates. We omitted hepatic encephalopathy – an important complication of severe disease (14) – based on experience of LAA development.(11) We found relevant codes (G92X Toxic encephalopathy; G934 Encephalopathy unspecified) were seldom recorded. Codes for altered consciousness or confusion were common but could indicate alcohol intoxication or withdrawal. Only 441 cases (2.5%) in the present study had encephalopathy coded, of which just 164 (<1%) lacked additional coding for ascites or varices.



For peer review only - http://bmjopen.bmj.com/site/about/guidelines.xhtml

Over the decade first admissions appeared to have somewhat more severe disease and higher burden of comorbidities. The possibility of changing coding practices over time cannot be excluded in any long term study of administrative data. However, our time trends for CFR were significant both for crude data and in sensitivity analyses omitting relevant covariates which suggests prognostic improvement is genuine rather than artefactual.

Third, whilst CPRD practices serve a representative population of English residents,(17) our results are not generalisable to every locality. A key aim was to examine events in primary care during the year before index admission but linked data are not available for the entire country. Further work using complete national HES is needed to quantify national trends and explore regional and provider variation.

Finally, although LAA performance in detecting true cases of ARLD against hospital records has been reported previously,(11,15) we could not conduct formal validity testing as inpatient data was limited to HES. However, linkage to death certification suggested the majority of in-hospital deaths in the uplift subgroup were related to alcoholic liver disease. The LAA is based on coding patterns observed in English HES data. It is unknown whether such variations exist in other countries.

In conclusion, we found evidence of gradual improvement in prognosis for first emergency admissions in England over a ten year period. Nevertheless, at least one in seven patients were still dying during index hospitalization and a third within a year. Many patients had contacts with health services in the year prior to admission but without apparent recognition of alcohol-related harms, adding to concerns about missed opportunities for screening and intervention.(20) Geographical variations require further investigation. Our observations support much of the pessimism expressed in the *Lancet Commission*'s final report(10) This highlights the need for concerted action in public health interventions and primary and secondary care to improve prevention, early detection and inpatient treatment of alcohol-related harms.

## Funding Statement

This work was funded by the UK Department of Health (Connected Health Cities programme) and delivered by the Northern Health Science Alliance. The funding sponsors had no role in the design and conduct of the study; in the collection, management, analysis and interpretation of the data; or in the preparation, review or approval of the manuscript.

## Data Availability Statement

This study is based in part on data from the Clinical Practice Research Datalink obtained under licence from the UK Medicines and Healthcare products Regulatory Agency. The data is provided by patients and collected by the NHS as part of their care


For peer review only - http://bmjopen.bmj.com/site/about/guidelines.xhtml

and support. The interpretation and conclusions contained in this study are those of the author/s alone. Copyright ©2023, re-used with the permission of The Health & Social Care Information Centre. All rights reserved. CPRD data are not publicy or freely available but can be obtained under special licence (https://cprd.com/data-access)

Code lists for the LAA are available at: https://onlinelibrary.wiley.com/action/downloadSupplement?doi=10.1111%2Fapt.15781&file=apt15781-sup-0001-Supinfo.docx

## Patient and Public Involvement

Patients or the public were not involved in the design, conduct, reporting or dissemination plans of our research.

## Authorship

Guarantor of the article: K.B.

Author contributions: K.B. wrote the manuscript and all authors reviewed and approved the final submission. K.B., K.F., B.S. and P.S. designed the research with input from all authors (T.M. and S.H.). K.B., B.S. and T.M. curated and analysed the data, with input from P.S. and K.F.

## Competing interests

No competing interest

## References


1. Williams R, Alexander G, Armstrong I, Baker A, Bhala N, Camps-Walsh G, et al. Disease burden and costs from excess alcohol consumption, obesity, and viral hepatitis: fourth report of the Lancet Standing Commission on Liver Disease in the UK. Lancet Lond Engl. 2018 Mar 17;391(10125):1097–107.

2. National confidential enquiry into patient outcome and death. Measuring the units: a review of patients who died with alcohol-related liver disease [Internet]. 2013 [cited 2019 Oct 1]. Available from: https://www.ncepod.org.uk/2013report1/downloads/MeasuringTheUnits_FullReport.pdf

3. Williams R, Horton R. Liver disease in the UK: a Lancet Commission. Lancet Lond Engl. 2013 Nov 9;382(9904):1537–8.

4. Williams R, Aspinall R, Bellis M, Camps-Walsh G, Cramp M, Dhawan A, et al. Addressing liver disease in the UK: a blueprint for attaining excellence in health care and reducing premature mortality from lifestyle issues of excess consumption of alcohol, obesity, and viral hepatitis. Lancet Lond Engl. 2014 Nov 29;384(9958):1953–97.

5. Williams R, Ashton K, Aspinall R, Bellis MA, Bosanquet J, Cramp ME, et al. Implementation of the Lancet Standing Commission on Liver Disease in the UK. Lancet Lond Engl. 2015 Nov 21;386(10008):2098–111.




For peer review only - http://bmjopen.bmj.com/site/about/guidelines.xhtml

6. Dyson JK, Rajasekhar P, Wetten A, Ashraf HH, Ng S, Paremal S, et al. Implementation of a 'care bundle' improves the management of patients admitted to hospital with decompensated cirrhosis. Aliment Pharmacol Ther. 2016;44(10):1030–8.

7. McPherson S, Dyson J, Austin A, Hudson M. Response to the NCEPOD report: development of a care bundle for patients admitted with decompensated cirrhosis-the first 24 h. Frontline Gastroenterol. 2016 Jan;7(1):16–23.

8. Kiat C, O'Mahony S. Tackling the epidemic: the NCEPOD report on alcohol-related liver disease and the Lancet Commission on Liver Disease. J R Coll Physicians Edinb. 2018 Dec;48(4):291–2.

9. Kanwal F, Kramer JR, Buchanan P, Asch SM, Assioun Y, Bacon BR, et al. The quality of care provided to patients with cirrhosis and ascites in the Department of Veterans Affairs. Gastroenterology. 2012 Jul;143(1):70–7.

10. Williams R, Aithal G, Alexander GJ, Allison M, Armstrong I, Aspinall R, et al. Unacceptable failures: the final report of the Lancet Commission into liver disease in the UK. Lancet Lond Engl. 2020 18;395(10219):226–39.

11. Kallis C, Dixon P, Silberberg B, Affarah L, Shawihdi M, Grainger R, et al. Reducing variation in hospital mortality for alcohol-related liver disease in North West England. Aliment Pharmacol Ther. 2020 Jul;52(1):182–95.

12. NHS Digital [Internet]. [cited 2022 Sep 28]. 1.8 Emergency admissions for alcohol related liver disease. Available from: https://digital.nhs.uk/data-and-information/publications/statistical/ccg-outcomes-indicator-set/june-2020/domain-1-preventing-people-from-dying-prematurely-ccg/1-8-emergency-admissions-for-alcohol-related-liver-disease

13. Williams R, Alexander G, Aspinall R, Bosanquet J, Camps-Walsh G, Cramp M, et al. New metrics for the Lancet Standing Commission on Liver Disease in the UK. Lancet Lond Engl. 2017 May 20;389(10083):2053–80.

14. McPherson S, Lucey MR, Moriarty KJ. Decompensated alcohol related liver disease: acute management. BMJ. 2016 Jan 26;352:i124.

15. Dhanda A, Bodger K, Hood S, Henn C, Allison M, Amasiatu C, et al. The Liverpool alcohol-related liver disease algorithm identifies twice as many emergency admissions compared to standard methods when applied to Hospital Episode Statistics for England. Aliment Pharmacol Ther. 2022;(In Press).

16. King J, Walker K, Van Der Meulen J, Bernal W. Editorial: the burden of alcohol-related liver disease emergency admissions in England may be twice as high as previously thought. Aliment Pharmacol Ther. 2022 In Press;

17. Wolf A, Dedman D, Campbell J, Booth H, Lunn D, Chapman J, et al. Data resource profile: Clinical Practice Research Datalink (CPRD) Aurum. Int J Epidemiol. 2019 Dec 1;48(6):1740–1740g.

For peer review only

18. Shawihdi M, Dodd S, Kallis C, Dixon P, Grainger R, Bloom S, et al. Nationwide improvement in outcomes of emergency admission for ulcerative colitis in England, 2005-2013. Aliment Pharmacol Ther. 2019 Jul;50(2):176–92.

19. Charlson ME, Pompei P, Ales KL, MacKenzie CR. A new method of classifying prognostic comorbidity in longitudinal studies: development and validation. J Chronic Dis. 1987;40(5):373–83.

20. Burton R, Sheron N. Missed opportunities for intervention in alcohol-related liver disease in the UK. Lancet Gastroenterol Hepatol. 2017;2(7):469–71.

21. Ratib S, West J, Fleming KM. Liver cirrhosis in England-an observational study: are we measuring its burden occurrence correctly? BMJ Open. 2017 Jul 13;7(7):e013752.

22. Shah ND, Ventura-Cots M, Abraldes JG, Alboraie M, Alfadhli A, Argemi J, et al. Alcohol-Related Liver Disease Is Rarely Detected at Early Stages Compared With Liver Diseases of Other Etiologies Worldwide. Clin Gastroenterol Hepatol Off Clin Pract J Am Gastroenterol Assoc. 2019 Oct;17(11):2320-2329.e12.

23. McPhail MJW, Parrott F, Wendon JA, Harrison DA, Rowan KA, Bernal W. Incidence and Outcomes for Patients With Cirrhosis Admitted to the United Kingdom Critical Care Units. Crit Care Med. 2018 May;46(5):705–12.

24. Liver Atlas of Variation [Internet]. [cited 2023 Sep 23]. Available from: http://tools.england.nhs.uk/images/LiverAtlas17/atlas.html

25. GBD 2017 Cirrhosis Collaborators. The global, regional, and national burden of cirrhosis by cause in 195 countries and territories, 1990-2017: a systematic analysis for the Global Burden of Disease Study 2017. Lancet Gastroenterol Hepatol. 2020 Mar;5(3):245–66.

26. Roberts SE, John A, Brown J, Napier DJ, Lyons RA, Williams JG. Early and late mortality following unscheduled admissions for severe liver disease across England and Wales. Aliment Pharmacol Ther. 2019;49(10):1334–45.

27. National Confidential Enquiry into Patient Outcome and Death. Remeasuring the Units: An update on the organisation of alcohol-related liver disease services [Internet]. 2022. Available from: https://www.ncepod.org.uk/pdf/current/Remeasuring%20the%20Units_full%20report.pdf

For peer review only



For peer review only - http://bmjopen.bmj.com/site/about/guidelines.xhtml

**Table 1. Demographics, clinical characteristics, care processes and outcomes of patients admitted as an emergency for the first time with alcohol-related liver disease between 2008/9 and 2017/18**. Patients registered with an English CPRD practice. Data are shown for the cohort overall ('Total') and by case definition based on the Liverpool ARLD Algorithm ('Primary' or 'Uplift').

| Characteristic | Total | Case definition | | p |
|---|---|---|---|---|
| | (n=17,575) | **Primary** (n=9,374) | **Uplift** (n=8,201) | |
| Age, *mean (sd)* | 53.4 (12) | 53.2 (12) | 53.6 (13) | * |
| Sex | | | | |
| *Female* | 5,820 (33.1%) | 3,335 (35.6%) | 2,485 (30.3%) | ** |
| *Male* | 11,755 (66.9%) | 6,039 (64.4%) | 5,716 (69.7%) | |
| Deprivation quintile | | | | |
| *1 - Least deprived* | 2,435 (13.9%) | 1,330 (14.2%) | 1,105 (13.5%) | *ns* |
| *2* | 2,735 (15.6%) | 1,481 (15.8%) | 1,254 (15.3%) | |
| *3* | 3,028 (17.2%) | 1,613 (17.2%) | 1,415 (17.3%) | |
| *4* | 3,837 (21.8%) | 2,077 (22.2%) | 1,760 (21.5%) | |
| *5 - Most deprived* | 5,540 (31.5%) | 2,873 (30.6%) | 2,667 (32.5%) | |
| Stage of liver disease | | | | |
| *Fatty liver* | 942 (5.4%) | 182 (1.9%) | 760 (9.3%) | ** |
| *Hepatitis* | 2,369 (13.5%) | 1,729 (18.4%) | 640 (7.8%) | |
| *Fibrosis and sclerosis* | 62 (0.4%) | 28 (0.3%) | 34 (0.4%) | |
| *Cirrhosis* | 5,001 (28.5%) | 2,759 (29.4%) | 2,242 (27.3%) | |
| *Hepatic failure* | 2,354 (13.4%) | 1,982 (21.1%) | 372 (4.5%) | |
| *Not specified* | 6,847 (39.0%) | 2,694 (28.7%) | 4,153 (50.6%) | |
| Ascites | 6,589 (37.5%) | 4,648 (49.6%) | 1,941 (23.7%) | ** |
| Varices | 2,704 (15.4%) | 1,508 (16.1%) | 1,196 (14.6%) | * |
| Comorbidity score (non-liver) | | | | |
| *0-1* | 15,506 (88.2%) | 8,440 (90.0%) | 7,066 (86.2%) | * |
| *2+* | 2,069 (11.8%) | 934 (10.0%) | 1,135 (13.8%) | |
| Five year period of discharge | | | | |
| *2008/9 - 2012/13* | 7,566 (43.0%) | 4,102 (43.8%) | 3,464 (42.2%) | * |
| *2013/14 - 2017/18* | 10,009 (57.0%) | 5,272 (56.2%) | 4,737 (57.8%) | |
| GP contact in last year | 9,936 (56.5%) | 5,346 (57.0%) | 4,590 (56.0%) | *ns* |
| *With alcohol codes recorded §* | 4702 (47.3%) | 2528 (47.3%) | 2174 (47.4%) | *ns* |
| *With liver codes recorded §* | 1465 (14.7%) | 865 (16.2%) | 600 (13.1%) | ** |
| Emergency admission in last year | 7,265 (41.3%) | 3,240 (34.6%) | 4,025 (49.1%) | * |
| *With alcohol codes recorded ¶* | 5,525 (76.0%) | 2,393 (73.9%) | 3,132 (77.8.%) | ** |
| Length of stay, mean (sd) | 12 (15) | 14 (16) | 9 (13) | ** |
| Higher level care during admission | 1,750 (10.0%) | 970 (10.3%) | 780 (9.5%) | *ns* |
| Died in-hospital | 2,708 (15.4%) | 1,779 (19.0%) | 929 (11.3%) | ** |
| Died within 365 days of admission | 5,938 (33.8%) | 3,642 (38.9%) | 2,296 (28.0%) | ** |

* p<0.05; **p<0.001 between primary and uplift groups; ns = not statistically significant; na = not applicable; § = percentages based on those with a GP contact within the last year; ¶ = percentages based on those with an emergency admission within the last year.

**Table 2. Factors associated with death following first emergency admission for alcohol-related liver disease among people registered with CPRD practices in England, 2008/9 to 2017/18.** The final case-mix adjustment models included seven patient level baseline covariates. Additional time-related variables were then added to investigate time trends, using either fiscal year of discharge (continuous) or 5-year time period. Figure 3 illustrates the time trends in crude and case-mix adjusted case fatality rates derived using these models.

| **Covariate** | **Death during first admission** | | | | **Death within 365 days** | | | |
|---|---|---|---|---|---|---|---|---|
| | **OR** | **95% CI** | **aOR** | **95% CI** | **OR** | **95% CI** | **aOR** | **95% CI** |
| **Age Group** *(Ref: <40 years)* | | | | | | | | |
| *40-49* | **1.42** | 1.18, 1.70 | **1.31** | 1.09, 1.58 | **1.33** | 1.18, 1.50 | **1.2** | 1.06, 1.37 |
| *50-59* | **2.36** | 1.99, 2.81 | **2.02** | 1.70, 2.43 | **2.01** | 1.79, 2.26 | **1.67** | 1.48, 1.88 |
| *60-69* | **3.39** | 2.85, 4.04 | **2.86** | 2.39, 3.43 | **2.97** | 2.63, 3.35 | **2.36** | 2.08, 2.68 |
| *70+* | **4.11** | 3.41, 4.98 | **3.33** | 2.73, 4.07 | **5.39** | 4.69, 6.20 | **4.14** | 3.57, 4.80 |
| **Sex** *(Ref: Female)* | | | | | | | | |
| *Male* | **0.86** | 0.79, 0.93 | **0.79** | 0.72, 0.87 | 1.00 | 0.94, 1.07 | **0.93** | 0.86, 0.99 |
| **Case Definition** *(Ref: Primary)* | | | | | | | | |
| *Uplift* | **0.55** | 0.50, 0.59 | **0.76** | 0.69, 0.84 | **0.61** | 0.57, 0.65 | **0.75** | 0.70, 0.81 |
| **Non-liver comorbidity** *(Ref: 0-1)* | | | | | | | | |
| *2+* | **2.38** | 2.14, 2.64 | **1.97** | 1.75, 2.21 | **2.92** | 2.66, 3.20 | **1.97** | 1.75, 2.21 |
| **Hepatic Failure** *(Ref: Any other stage)* | | | | | | | | |
| *Hepatic failure* | **4.73** | 4.29, 5.21 | **4.03** | 3.64, 4.48 | **2.87** | 2.62, 3.13 | **2.32** | 2.11, 2.55 |
| **Ascites** *(Ref: No)* | | | | | | | | |
| *Yes* | **2.03** | 1.87, 2.21 | **1.48** | 1.35, 1.62 | **2.31** | 2.17, 2.47 | **1.86** | 1.74, 2.00 |
| **Varices** *(Ref: No)* | | | | | | | | |
| *Yes* | **1.35** | 1.21, 1.50 | **1.2** | 1.07, 1.34 | **1.41** | 1.30, 1.53 | **1.21** | 1.11, 1.33 |
| | Model 1, C-statistic: 0.733 | | | | Model 2, C-statistic: 0.710 | | | |
| **Fiscal Year** *(per year)* | | | | | | | | |
| | **0.98** | 0.96, 0.99 | **0.94** | 0.93, 0.96 | **0.98** | 0.97, 0.99 | **0.96** | 0.95, 0.97 |
| | Fiscal year added to Model 1, C-statistic: 0.737 | | | | Fiscal year added to Model 2, C-statistic: 0.710 | | | |
| **Five Year Period** *(Ref: 2008/9 to 2012/13)* | | | | | | | | |
| *2013/14 to 2017/18* | **0.91** | 0.83, 0.98 | **0.76** | 0.69, 0.83 | **0.91** | 0.83, 0.98 | **0.83** | 0.77, 0.88 |
| | Five Year Period added to Model 1, C-statistic: 0.736 | | | | Five Year Period added to Model 2, C-statistic: 0.708 | | | |

OR=unadjusted odds ratio; aOR =risk-adjusted odds ratio; significant ORs are in bold text (p values <0.001 in all cases)



For peer review only - http://bmjopen.bmj.com/site/about/guidelines.xhtml

## Legends to Figures

**Figure 1. Distribution of stage of alcohol-related liver disease at first emergency admission identified from the CPRD population of England.** Stage is based on the ICD-10 codes recorded on discharge (n=17,575 cases). Stratified by: **(A)** Age group **(B)** Sex, **(C)** Deprivation Quintile (1=Least, 5=Most deprived areas) and **(D)** Five-year period of discharge. All p values <0.001 (Chi Squared).

.

**Figure 2. Kaplan-Meier survival probability following first emergency admission for alcohol-related liver disease.** **(A)** to **(G)** are stratified into five-year cohorts (2008/9 to 2012/13 versus 20013/14 to 2017/18): **(A)** Overall (n=17,575), **(B)** Primary case definition (n=9,374), **(C)** Uplift case definition (n=8,201), **(D)** Alcoholic hepatitis (n=2,369), **(E)** Cirrhosis (n=5,001), **(F)** Hepatic failure (n=2,354), **(G)** Unspecified stage of liver disease (n=6,847). **(H)** shows all cases stratified by recorded-stage of liver disease. p values represent significance level of log rank test.

**Figure 3. Time trends in case fatality rates (CFR) for first emergency admissions for alcohol-related liver disease, overall and by stage of liver disease. (A)** Crude CFR in-hospital, **(B)** Risk-adjusted CFR in-hospital, **(C)** Crude CFR within 365 days, **(D)** Risk-adjusted CFR within 365 days. Risk-adjustment for overall rates was by age, sex, case definition (primary or uplift), comorbidity score (non-liver), stage of liver disease (liver failure or not) and coding for ascites and varices. Binary logistic regression models confirmed significant downward linear trends over the ten years overall, before and after risk-adjustment (See Table 2). For stage-specific rates, risk-adjustment was by age, sex, case definition and comorbidity score, with additional relevant models confirming significance of trends.

For peer review only



For peer review only - http://bmjopen.bmj.com/site/about/guidelines.xhtml

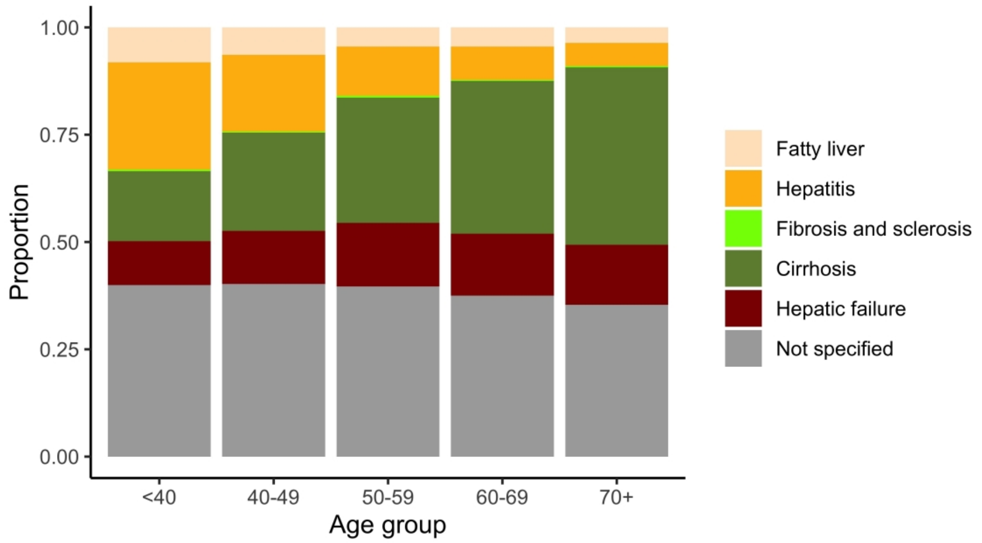


1A

1270x846mm (72 x 72 DPI)

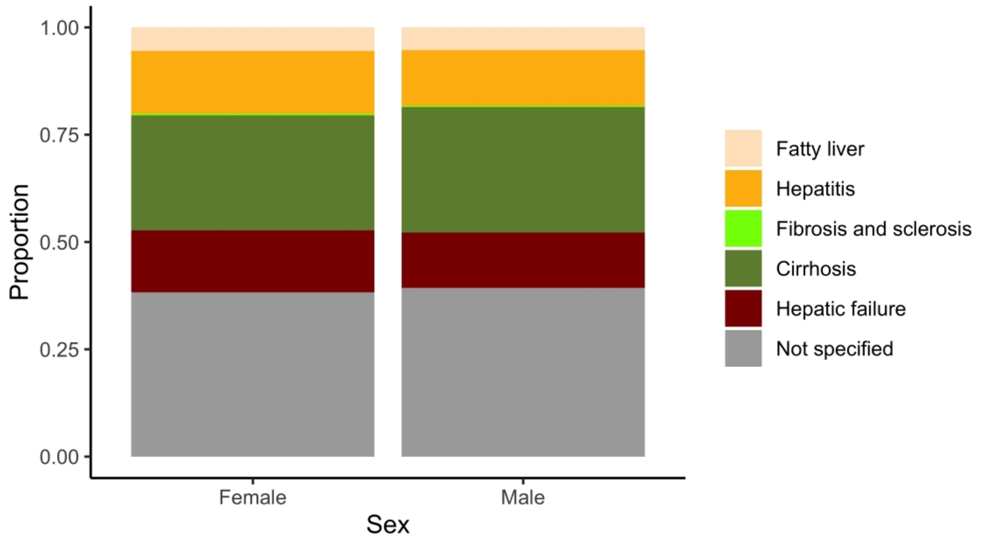


1B

1270x846mm (72 x 72 DPI)

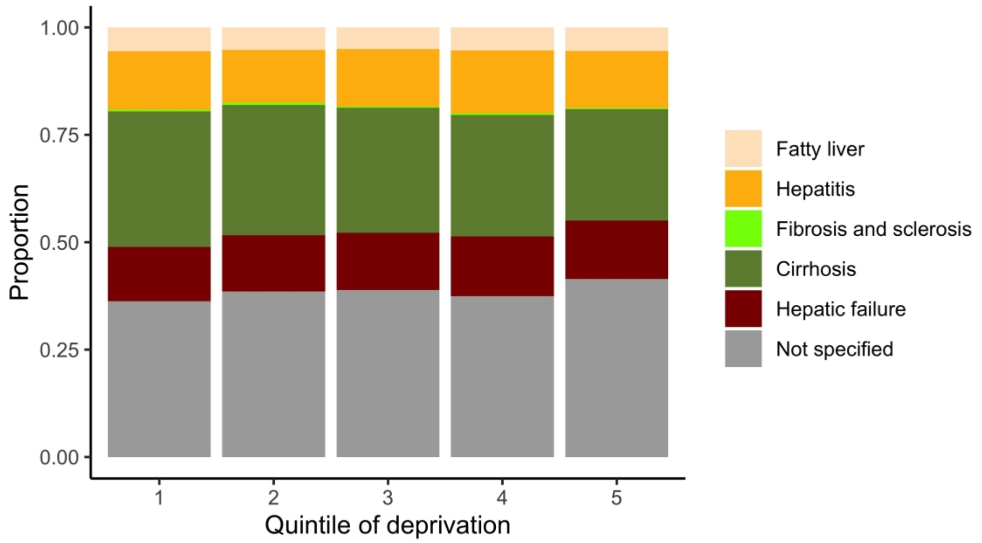


1C

1270x846mm (72 x 72 DPI)

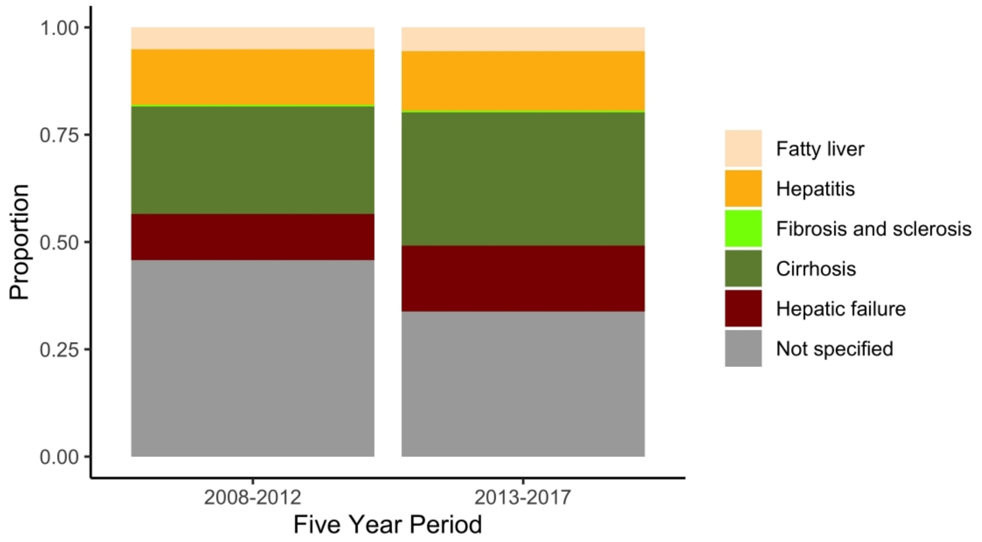


1D

1270x846mm (72 x 72 DPI)

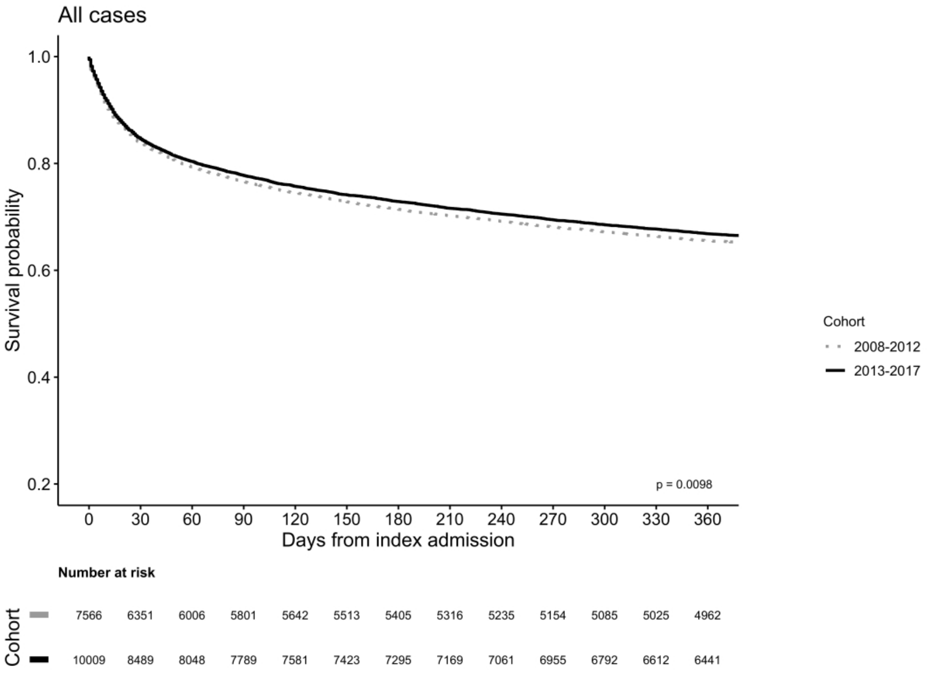


2A

2116x1481mm (72 x 72 DPI)

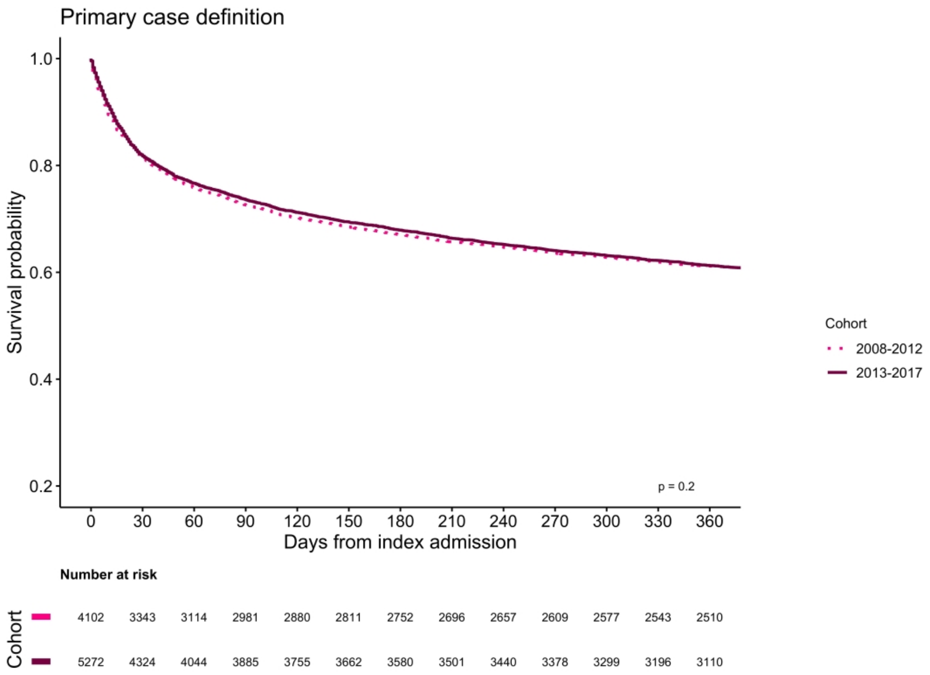


2B

2116x1481mm (72 x 72 DPI)

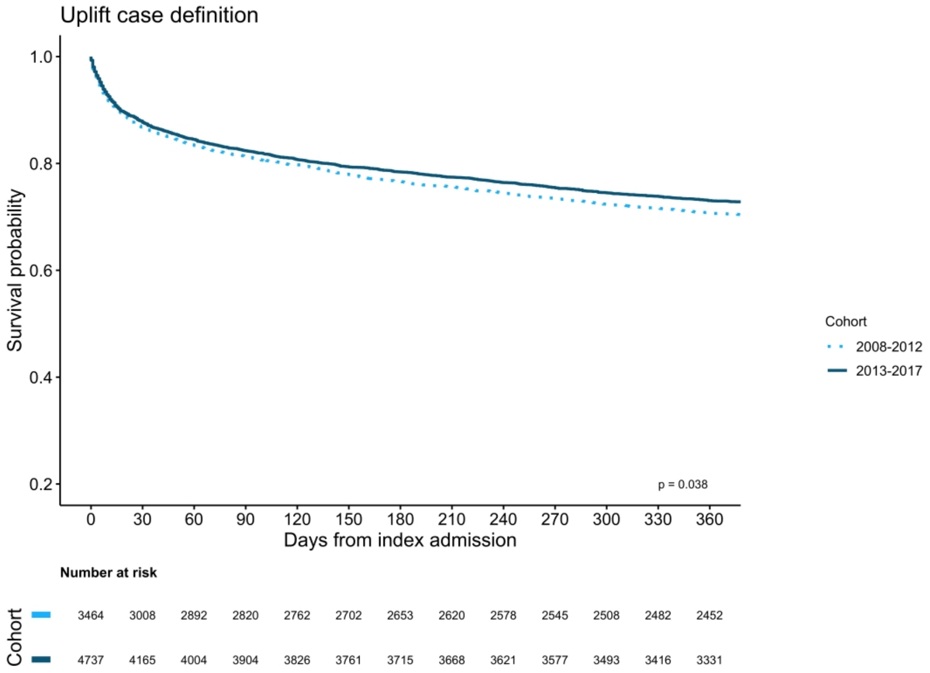


2C

2116x1481mm (72 x 72 DPI)

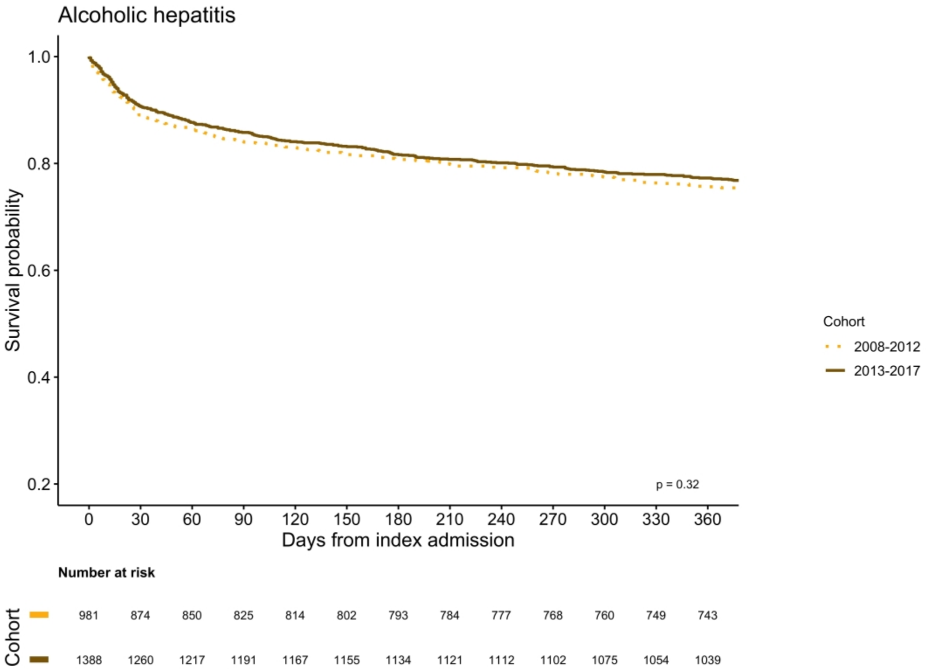


2D

2116x1481mm (72 x 72 DPI)

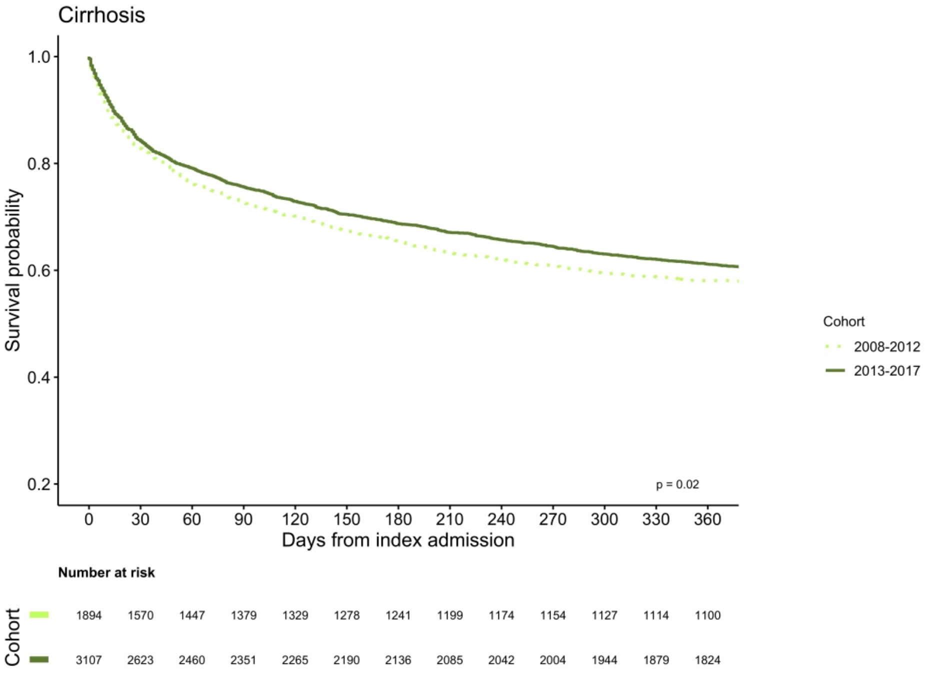


2E

2116x1481mm (72 x 72 DPI)

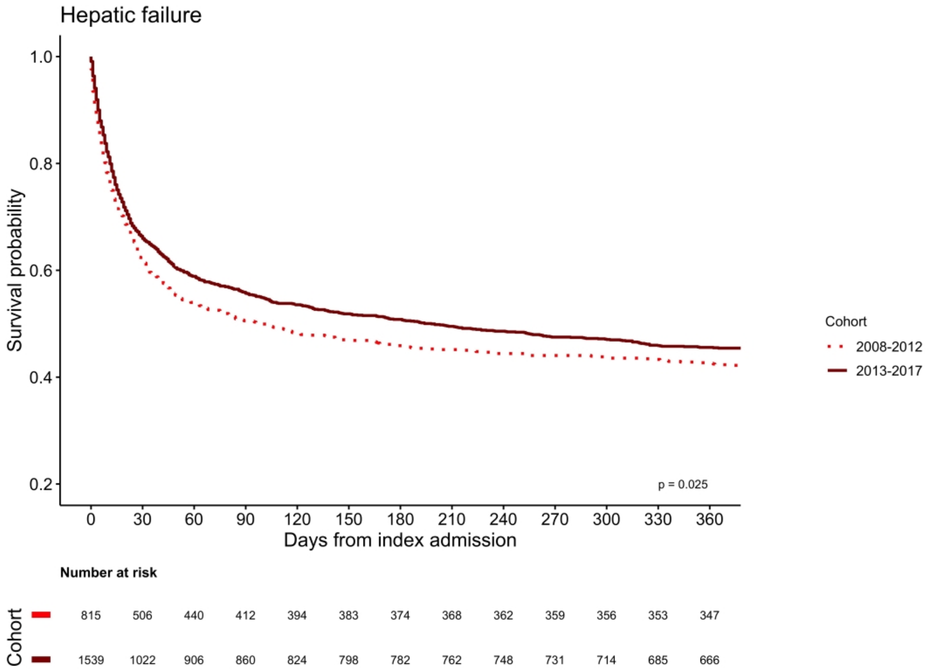


2F

2116x1481mm (72 x 72 DPI)

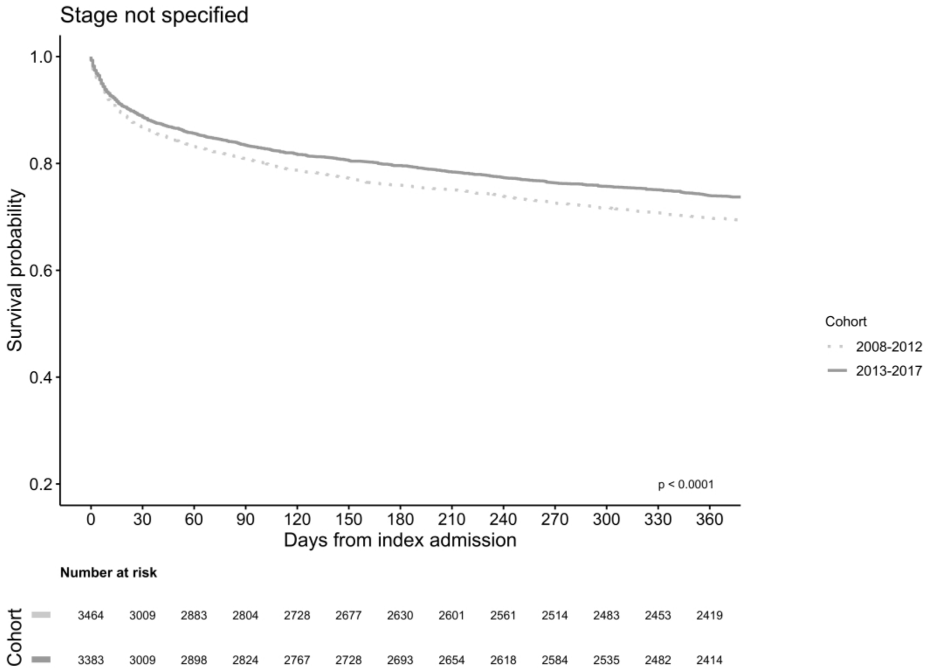


2G

2116x1481mm (72 x 72 DPI)

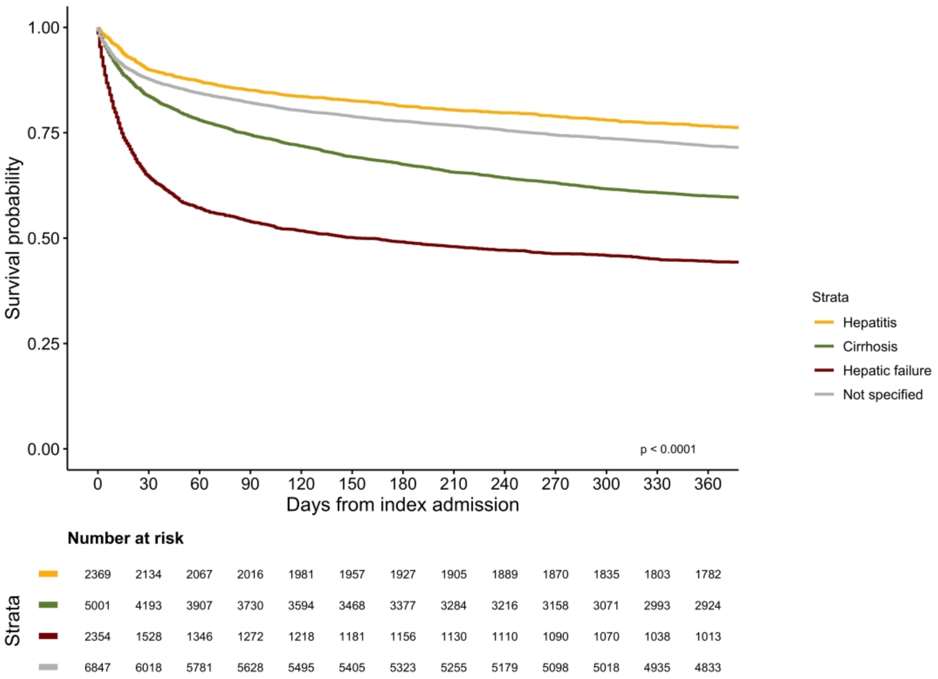


2H

2116x1481mm (72 x 72 DPI)

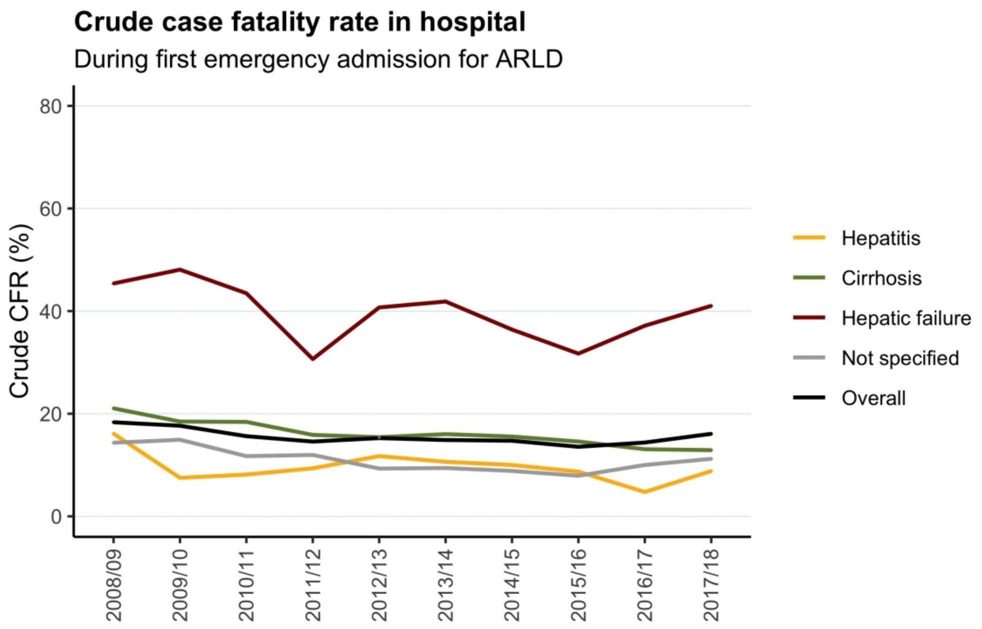


3A

846x564mm (72 x 72 DPI)

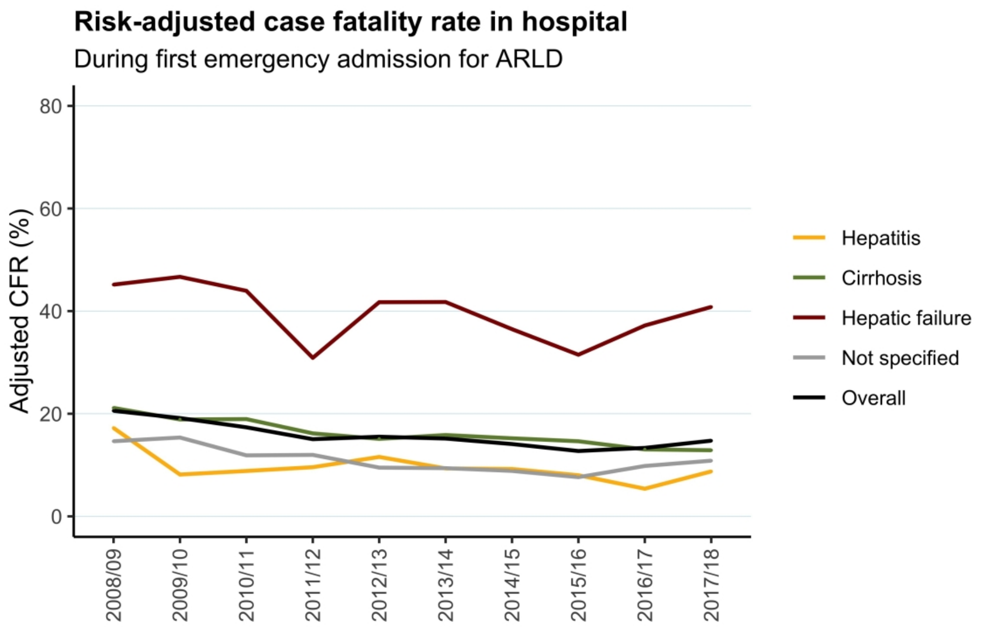


3B

1270x846mm (72 x 72 DPI)

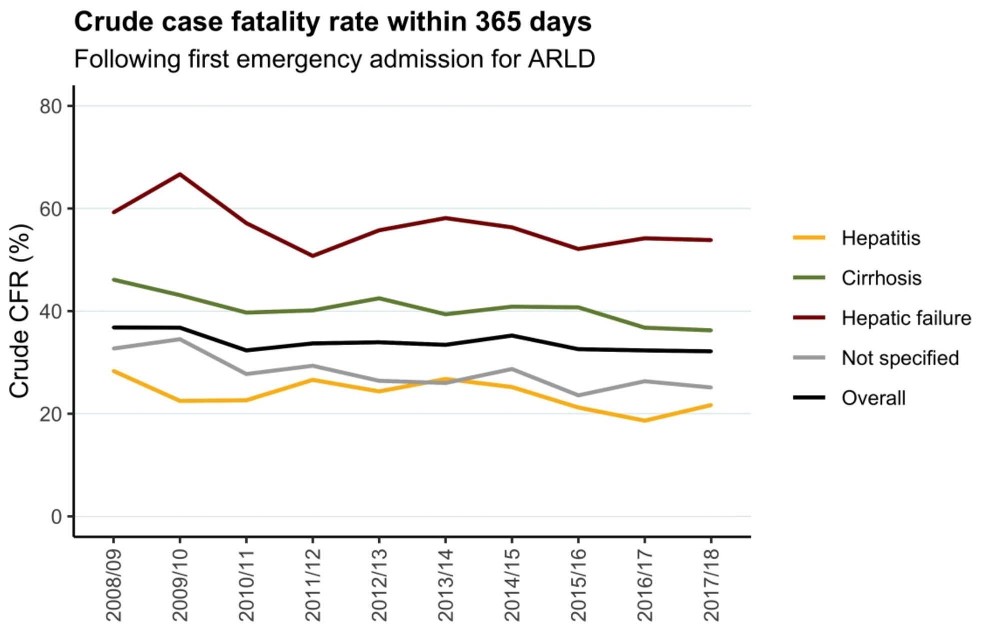


3C

1270x846mm (72 x 72 DPI)

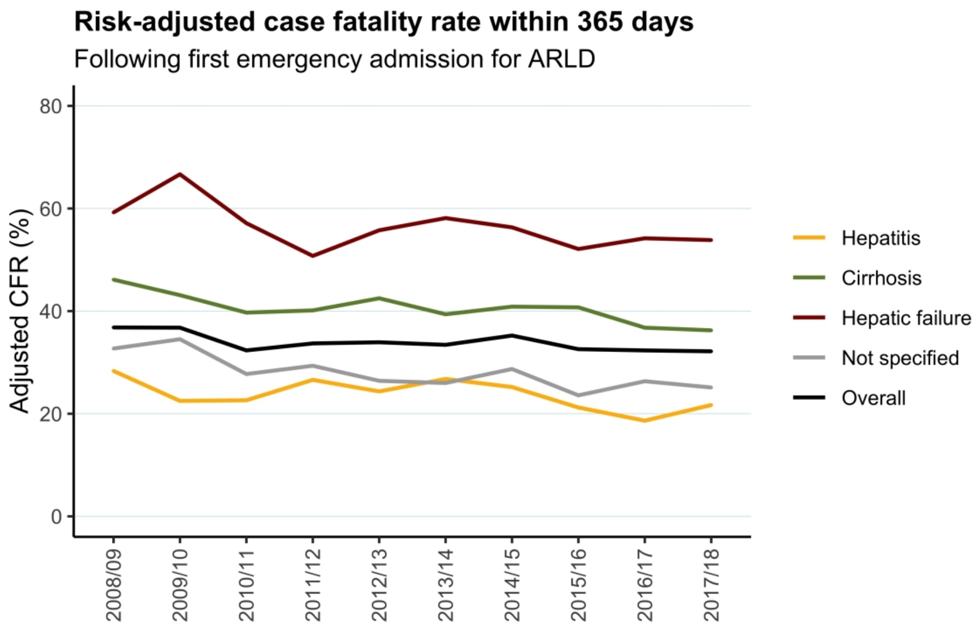


3D

1270x846mm (72 x 72 DPI)

# Supplementary Materials & Appendices: BMJ Open

## Outcomes of first emergency admissions for alcohol-related liver disease in England over a ten year period: Retrospective observational cohort study using linked electronic databases

**Supplementary Figure S1.** Kaplan-Meier survival probability of first emergency admissions with a diagnosis of alcohol-related liver disease, stratified by patient-level covariates

**Supplementary Table S1.** Demographics, clinical characteristics, care processes and outcomes of patients admitted as an emergency for the first time with alcohol-related liver disease between 2008/9 and 2017/18. Stratified by stage of liver disease.

**Supplementary Table S2.** Demographics, clinical characteristics, care processes and outcomes of patients admitted as an emergency for the first time with alcohol-related liver disease between 2008/9 and 2017/18. Stratified by 5-year period of discharge.

**Supplementary Table S3.** Demographics, clinical characteristics, care processes and outcomes of patients admitted as an emergency for the first time with alcohol-related liver disease between 2008/9 and 2017/18. Stratified by fiscal year of discharge.

**Supplementary Table S4.** Demographics and clinical characteristics of those who survived or died following first emergency admission with alcohol-related liver disease between 2008/09 and 2017/18.

**Supplementary Figure S2.** Sensitivity analyses of main findings exploring alternative covariates for risk-adjustment models

**Supplementary Table S5.** Demographics, clinical characteristics, care processes and outcomes of patients admitted as an emergency for the first time with alcohol-related liver disease between 2008/9 and 2017/18. Stratified by region of residence.

**Supplementary Figure S3**. Funnel plot showing regional variation in crude (unadjusted) case fatality rate following first emergency admission for alcohol-related liver disease among people registered with CPRD practices in England, 2008/9 to 2017/18.

**Supplementary Table S6.** Exploratory Analysis: Association between region of residence and death following first emergency admission for alcohol-related liver disease among people registered with CPRD practices in England, 2008/9 to 2017/18.

**Supplementary Appendix 1.** Medcodes and corresponding readterms used for primary care records to identify entries relating to alcohol misuse and/or alcohol-related liver disease (ARLD). Codes for ARLD are marked with an asterix (*).

**Supplementary Appendix 2.** used for screening all diagnoses listed on the death certificate in patients dying during index admission for ARLD.

**Supplementary Appendix 3.** Higher definition images for time serices (**Figure 3**).

For peer review only

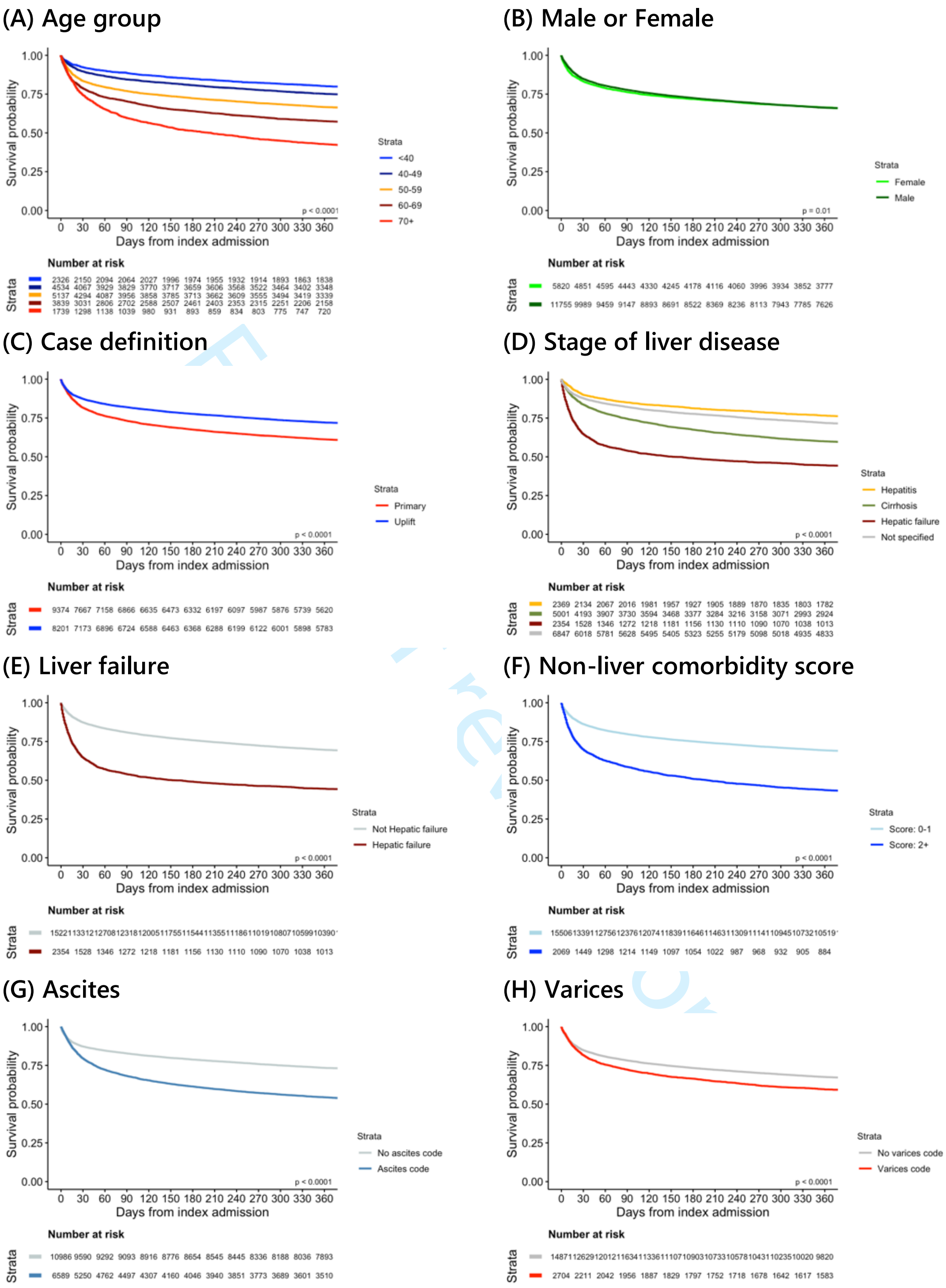


**Supplementary Figure S1. Kaplan-Meier survival probability of first emergency admissions with a diagnosis of alcohol-related liver disease, stratified by patient-level covariates.** (A) Age group, (B) Sex, (C) Case definition, (D) Stage of liver disease (selected stages only), (E) Liver failure or Not, (F) Non-liver comorbidity score, (G) Presence of codes for ascites, (H) Presence of codes for varices. All p values were significant between strata by log rank test.

**Supplementary Table S1.** Demographics, clinical characteristics, care processes and outcomes of patients admitted as an emergency for the first time with alcohol-related liver disease between 2008/9 and 2017/18. Patients registered with an English CPRD practice. Stratified by stage of liver disease. Excludes fatty liver (n=942) and fibrosis & sclerosis (n=62).

| Characteristic | Hepatitis (n=2,369) | Cirrhosis (n=5,001) | Hepatic failure (n=2,354) | Not specified (n=6,847) | p |
|---|---|---|---|---|---|
| Age, mean (sd) | 47.7 (12) | 56.8 (12) | 54.6 (12) | 53.0 (12) | ** |
| Sex | | | | | |
| *Female* | 848 (35.8%) | 1,562 (31.2%) | 839 (35.6%) | 2,227 (32.5%) | ** |
| *Male* | 1,521 (64.2%) | 3,439 (68.8%) | 1,515 (64.4%) | 4,620 (67.5%) | |
| Deprivation quintile | | | | | |
| *1 - Least deprived* | 332 (14.0%) | 769 (15.4%) | 307 (13.0%) | 883 (12.9%) | ** |
| *2* | 337 (14.2%) | 831 (16.6%) | 357 (15.2%) | 1,054 (15.4%) | |
| *3* | 404 (17.1%) | 882 (17.6%) | 404 (17.2%) | 1,176 (17.2%) | |
| *4* | 560 (23.6%) | 1,084 (21.7%) | 533 (22.6%) | 1,437 (21.0%) | |
| *5 - Most deprived* | 736 (31.1%) | 1,435 (28.7%) | 753 (32.0%) | 2,297 (33.5%) | |
| Case definition | | | | | |
| *Primary* | 1,729 (73.0%) | 2,759 (55.2%) | 1,982 (84.2%) | 2,694 (39.3%) | ** |
| *Uplift* | 640 (27.0%) | 2,242 (44.8%) | 372 (15.8%) | 4,153 (60.7%) | |
| Ascites | 531 (22.4%) | 2,472 (49.4%) | 1,383 (58.8%) | 2,109 (30.8%) | ** |
| Varices | 154 (6.5%) | 1,261 (25.2%) | 415 (17.6%) | 845 (12.3%) | ** |
| Comorbidity score (non-liver) | | | | | |
| *0-1* | 2,255 (95.2%) | 4,150 (83.0%) | 2,040 (86.7%) | 6,132 (89.6%) | ** |
| *2+* | 114 (4.8%) | 851 (17.0%) | 314 (13.3%) | 715 (10.4%) | |
| Five-year period of discharge | | | | | |
| *2008/9 – 2012/13* | 981 (41.4%) | 1,894 (37.9%) | 815 (34.6%) | 3,464 (50.6%) | ** |
| *2013/14 – 2017/18* | 1,388 (58.6%) | 3,107 (62.1%) | 1,539 (65.4%) | 3,383 (49.4%) | |
| GP contact in last year | 1,268 (53.5%) | 2,921 (58.4%) | 1,336 (56.8%) | 3,887 (56.8%) | ** |
| *With alcohol codes recorded §* | 661 (52.1%) | 1302 (44.6%) | 619 (46.3%) | 1849 (47.6%) | ** |
| *With liver codes recorded §* | 94 (7.4%) | 593 (20.3%) | 228 (17.1%) | 514 (13.2%) | *ns* |
| Emergency admission in last year | 918 (38.8%) | 2,011 (40.2%) | 832 (35.3%) | 3,029 (44.2%) | ** |
| *With alcohol codes recorded ¶* | 771 (84.0%) | 1,407 (70.0%) | 593 (71.3%) | 2,352 (77.7%) | ** |
| Length of stay, mean (sd) | 12 (15) | 12 (16) | 16 (18) | 10 (13) | ** |
| Higher level care during admission | 138 (5.8%) | 508 (10.2%) | 496 (21.1%) | 556 (8.1%) | ** |
| Died in-hospital | 221 (9.3%) | 781 (15.6%) | 912 (38.7%) | 761 (11.1%) | ** |
| Died within 365 days of admission | 557 (23.5%) | 2,003 (40.1%) | 1,309 (55.6%) | 1,931 (28.2%) | ** |

* $p<0.05$; ** $p<0.001$ between groups; ns = not significant; na = not applicable; § = percentages based on those with a GP contact within the last year; ¶ = percentages based on those with an emergency admission within the last year.



For peer review only - http://bmjopen.bmj.com/site/about/guidelines.xhtml

**Supplementary Table S2.** Demographics, clinical characteristics, care processes and outcomes of patients admitted as an emergency for the first time with alcohol-related liver disease between 2008/9 and 2017/18. Patients registered with an English CPRD practice. Stratified by 5-year period of discharge.

| Characteristic | Total | 5 Year Period of Discharge | | p |
|---|---|---|---|---|
| | **08/09 to 17/18** (n=17,575) | **08/09 to 12/13** (n=7,566) | **13/14 to 17/18** (n=10,009) | |
| Age, *mean (sd)* | 53.4 (12) | 52.5 (12) | 54.1 (12) | ** |
| Sex | | | | |
| *Female* | 5,820 (33.1%) | 2,477 (33%) | 3,343 (33%) | *ns* |
| *Male* | 11,755 (66.9%) | 5,089 (67%) | 6,666 (67%) | |
| Deprivation quintile | | | | |
| *1 - Least deprived* | 2,435 (13.9%) | 977 (13%) | 1,458 (15%) | ** |
| *2* | 2,735 (15.6%) | 1,162 (15%) | 1,573 (16%) | |
| *3* | 3,028 (17.2%) | 1,223 (16%) | 1,805 (18%) | |
| *4* | 3,837 (21.8%) | 1,674 (22%) | 2,163 (22%) | |
| *5 - Most deprived* | 5,540 (31.5%) | 2,530 (33%) | 3,010 (30%) | |
| Case definition | | | | |
| Primary | 7,595 (51.1%) | 4,102 (54%) | 5,272 (53%) | * |
| Uplift | 7,272 (48.9%) | 3,464 (46%) | 4,737 (47%) | |
| Stage of liver disease | | | | |
| *Fatty liver* | 942 (5.4%) | 385 (5.1%) | 557 (5.6%) | ** |
| *Hepatitis* | 2,369 (13.5%) | 981 (13%) | 1,388 (14%) | |
| *Fibrosis and sclerosis* | 62 (0.4%) | 27 (0.4%) | 35 (0.3%) | |
| *Cirrhosis* | 5,001 (28.5%) | 1,894 (25%) | 3,107 (31%) | |
| *Hepatic failure* | 2,354 (13.4%) | 815 (11%) | 1,539 (15%) | |
| *Not specified* | 6,847 (39.0%) | 3,464 (46%) | 3,383 (34%) | |
| Ascites | 6,589 (37.5%) | 2,799 (37%) | 3,790 (38%) | *ns* |
| Varices | 2,704 (15.4%) | 1,113 (15%) | 1,591 (16%) | * |
| Comorbidity score (non-liver) | | | | |
| *0-1* | 15,506 (88.2%) | 6,773 (90%) | 8,733 (87%) | ** |
| *2+* | 2,069 (11.8%) | 793 (10%) | 1,276 (13%) | |
| GP contact in last year | 9,936 (56.5%) | 4,513 (59.6%) | 5,423 (54.2%) | ** |
| *With alcohol codes recorded §* | 4702 (47.3%) | 2,173 (48.1%) | 2,529 (46.6%) | *ns* |
| *With liver codes recorded §* | 1465 (14.7%) | 724 (16.0%) | 741 (13.7%) | ** |
| Emergency admission in last year | 7,265 (41.3%) | 3,127 (41%) | 4,138 (41%) | *ns* |
| *With alcohol codes recorded ¶* | 5,525 (76.0%) | 2,398 (76.7%) | 3,127 (75.6%) | *ns* |
| Length of stay, mean (sd) | 12 (15) | 12 (15) | 11 (14) | ** |
| Higher level care during admission | 1,750 (10.0%) | 652 (8.6%) | 1,098 (11%) | ** |
| Died in-hospital | 2,708 (15.4%) | 1,222 (16%) | 1,486 (15%) | * |
| Died within 365 days of admission | 5,938 (33.8%) | 2,614 (35%) | 3,324 (33%) | *ns* |

* $p<0.05$; ** $p<0.001$ between time periods; ns = not significant; § = percentages based on those with a GP contact within the last year; ¶ = percentages based on those with an emergency admission within the last year.

**Supplementary Table S3.** Demographics, clinical characteristics, care processes and outcomes of patients admitted as an emergency for the first time with alcohol-related liver disease between 2008/9 and 2017/18. Patients registered with an English CPRD practice. Stratified by fiscal year of discharge.

| **Characteristic** | **2008/09** | **2009/10** | **2010/11** | **2011/12** | **2012/13** | **2013/14** | **2014/15** | **2015/16** | **2016/17** | **2017/18** |
|---|---|---|---|---|---|---|---|---|---|---|
| | N = 1,494 | N = 1,512 | N = 1,645 | N = 1,626 | N = 1,692 | N = 1,741 | N = 1,771 | N = 1,992 | N = 2,044 | N = 2,058 |
| **Age**, mean (sd) | 52 (12) | 52 (12) | 52 (12) | 53 (13) | 53 (12) | 54 (12) | 54 (12) | 54 (12) | 54 (12) | 55 (13) |
| **Age group** | | | | | | | | | | |
| *<40* | 220 (14.7%) | 232 (15.3%) | 257 (15.6%) | 244 (15.0%) | 236 (13.9%) | 209 (12.0%) | 225 (12.7%) | 228 (11.4%) | 236 (11.5%) | 239 (11.6%) |
| *40-49* | 429 (28.7%) | 419 (27.7%) | 439 (26.7%) | 431 (26.5%) | 451 (26.7%) | 440 (25.3%) | 444 (25.1%) | 486 (24.4%) | 518 (25.3%) | 477 (23.2%) |
| *50-59* | 423 (28.3%) | 403 (26.7%) | 491 (29.8%) | 433 (26.6%) | 490 (29.0%) | 542 (31.1%) | 536 (30.3%) | 580 (29.1%) | 629 (30.8%) | 610 (29.6%) |
| *60-69* | 293 (19.6%) | 337 (22.3%) | 326 (19.8%) | 352 (21.6%) | 361 (21.3%) | 390 (22.4%) | 382 (21.6%) | 486 (24.4%) | 451 (22.1%) | 461 (22.4%) |
| *70+* | 129 (8.6%) | 121 (8.0%) | 132 (8.0%) | 166 (10.2%) | 154 (9.1%) | 160 (9.2%) | 184 (10.4%) | 212 (10.6%) | 210 (10.3%) | 271 (13.2%) |
| **Sex** | | | | | | | | | | |
| *Female* | 505 (33.8%) | 488 (32.3%) | 527 (32.0%) | 526 (32.3%) | 559 (33.0%) | 533 (30.6%) | 607 (34.3%) | 669 (33.6%) | 697 (34.1%) | 709 (34.5%) |
| *Male* | 989 (66.2%) | 1,024 (67.7%) | 1,118 (68.0%) | 1,100 (67.7%) | 1,133 (67.0%) | 1,208 (69.4%) | 1,164 (65.7%) | 1,323 (66.4%) | 1,347 (65.9%) | 1,349 (65.5%) |
| **Deprivation quintile** | | | | | | | | | | |
| *1 - Least deprived* | 174 (11.6%) | 209 (13.8%) | 201 (12.2%) | 207 (12.7%) | 243 (14.4%) | 253 (14.5%) | 240 (13.6%) | 287 (14.4%) | 320 (15.7%) | 301 (14.6%) |
| *2* | 236 (15.8%) | 222 (14.7%) | 243 (14.8%) | 250 (15.4%) | 264 (15.6%) | 274 (15.7%) | 267 (15.1%) | 331 (16.6%) | 289 (14.1%) | 359 (17.4%) |
| *3* | 264 (17.7%) | 219 (14.5%) | 282 (17.1%) | 259 (15.9%) | 264 (15.6%) | 298 (17.1%) | 336 (19.0%) | 362 (18.2%) | 376 (18.4%) | 368 (17.9%) |
| *4* | 334 (22.4%) | 339 (22.4%) | 361 (21.9%) | 344 (21.2%) | 398 (23.5%) | 369 (21.2%) | 365 (20.6%) | 424 (21.3%) | 438 (21.4%) | 465 (22.6%) |
| *5 - Most deprived* | 486 (32.5%) | 523 (34.6%) | 558 (33.9%) | 566 (34.8%) | 523 (30.9%) | 547 (31.4%) | 563 (31.8%) | 588 (29.5%) | 621 (30.4%) | 565 (27.5%) |
| **Case definition** | | | | | | | | | | |
| *Primary* | 843 (56.4%) | 819 (54.2%) | 878 (53.4%) | 870 (53.5%) | 906 (53.5%) | 897 (51.5%) | 959 (54.2%) | 1,054 (52.9%) | 1,092 (53.4%) | 1,056 (51.3%) |
| *Uplift* | 651 (43.6%) | 693 (45.8%) | 767 (46.6%) | 756 (46.5%) | 786 (46.5%) | 844 (48.5%) | 812 (45.8%) | 938 (47.1%) | 952 (46.6%) | 1,002 (48.7%) |
| **Stage of liver disease** | | | | | | | | | | |
| *Fatty liver* | 59 (3.9%) | 82 (5.4%) | 94 (5.7%) | 75 (4.6%) | 95 (5.6%) | 103 (5.9%) | 98 (5.5%) | 113 (5.7%) | 116 (5.7%) | 107 (5.2%) |
| *Hepatitis* | 180 (12.0%) | 200 (13.2%) | 221 (13.4%) | 203 (12.5%) | 230 (13.6%) | 254 (14.6%) | 250 (14.1%) | 264 (13.3%) | 295 (14.4%) | 272 (13.2%) |
| *Fibrosis and sclerosis* | 7 (0.5%) | 3 (0.2%) | 6 (0.4%) | 6 (0.4%) | 6 (0.4%) | 5 (0.3%) | 10 (0.6%) | 5 (0.3%) | 6 (0.3%) | 8 (0.4%) |
| *Cirrhosis* | 323 (21.6%) | 341 (22.6%) | 413 (25.1%) | 441 (27.1%) | 480 (28.4%) | 518 (29.8%) | 553 (31.2%) | 631 (31.7%) | 650 (31.8%) | 651 (31.6%) |
| *Hepatic failure* | 130 (8.7%) | 156 (10.3%) | 161 (9.8%) | 199 (12.2%) | 226 (13.4%) | 203 (11.7%) | 261 (14.7%) | 309 (15.5%) | 358 (17.5%) | 351 (17.1%) |
| *Not specified* | 795 (53.2%) | 730 (48.3%) | 750 (45.6%) | 702 (43.2%) | 655 (38.7%) | 658 (37.8%) | 599 (33.8%) | 670 (33.6%) | 619 (30.3%) | 669 (32.5%) |
| **Ascites** | 219 (14.7%) | 205 (13.6%) | 234 (14.2%) | 263 (16.2%) | 250 (14.8%) | 294 (16.9%) | 329 (18.6%) | 305 (15.3%) | 307 (15.0%) | 298 (14.5%) |
| **Varices** | 14 (0.9%) | 16 (1.1%) | 20 (1.2%) | 27 (1.7%) | 26 (1.5%) | 36 (2.1%) | 26 (1.5%) | 31 (1.6%) | 33 (1.6%) | 29 (1.4%) |
| **Comorbidity Score (non-liver)** | | | | | | | | | | |
| *0-1* | 1,341 (89.8%) | 1,357 (89.7%) | 1,469 (89.3%) | 1,456 (89.5%) | 1,505 (88.9%) | 1,515 (87.0%) | 1,538 (86.8%) | 1,746 (87.7%) | 1,792 (87.7%) | 1,787 (86.8%) |
| *2+* | 153 (10.2%) | 155 (10.3%) | 176 (10.7%) | 170 (10.5%) | 187 (11.1%) | 226 (13.0%) | 233 (13.2%) | 246 (12.3%) | 252 (12.3%) | 271 (13.2%) |
| **GP contact in last year** | 940 (62.9%) | 922 (61.0%) | 967 (58.8%) | 966 (59.4%) | 950 (56.1%) | 983 (56.5%) | 959 (54.2%) | 1,113 (55.9%) | 1,078 (52.7%) | 1,058 (51.4%) |
| *With alcohol codes recorded §* | 433 (46.1%) | 439 (47.6%) | 486 (50.3%) | 460 (47.6%) | 465 (48.9%) | 505 (51.4%) | 449 (46.8%) | 527 (47.3%) | 480 (44.5%) | 458 (43.3%) |
| *With liver codes recorded §* | 164 (17.4%) | 161 (17.5%) | 164 (17.0%) | 138 (14.3%) | 137 (14.4%) | 163 (16.6%) | 129 (13.5%) | 155 (13.9%) | 143 (13.3%) | 111 (10.5%) |
| **Emergency admission in last year** | 583 (39.0%) | 665 (44.0%) | 689 (41.9%) | 662 (40.7%) | 702 (41.5%) | 718 (41.2%) | 740 (41.8%) | 821 (41.2%) | 837 (40.9%) | 848 (41.2%) |
| *With alcohol codes ¶* | 443 (76.0%) | 506 (76.1%) | 541 (78.5%) | 502 (75.8%) | 538 (76.6%) | 556 (77.4%) | 543 (73.4%) | 616 (75.0%) | 631 (75.4%) | 649 (76.5%) |
| **Length of stay**, mean (sd) | 13 (16) | 13 (17) | 12 (15) | 11 (15) | 12 (15) | 12 (15) | 12 (15) | 11 (14) | 11 (14) | 11 (14) |
| **Higher level care during admission** | 92 (6.2%) | 149 (9.9%) | 157 (9.5%) | 146 (9.0%) | 149 (8.8%) | 192 (11.0%) | 211 (11.9%) | 198 (9.9%) | 224 (11.0%) | 232 (11.3%) |
| **Died in-hospital** | 274 (18.3%) | 267 (17.7%) | 257 (15.6%) | 237 (14.6%) | 258 (15.2%) | 259 (14.9%) | 261 (14.7%) | 270 (13.6%) | 294 (14.4%) | 331 (16.1%) |
| **Died with 365 days of admission** | 550 (36.8%) | 556 (36.8%) | 532 (32.3%) | 548 (33.7%) | 574 (33.9%) | 582 (33.4%) | 624 (35.2%) | 649 (32.6%) | 661 (32.3%) | 662 (32.2%) |

§ = percentages based on those with a GP contact within the last year; ¶ = percentages based on those with an emergency admission within the last year.



For peer review only

**Supplementary Table S4. Demographics and clinical characteristics of those who survived or died following first emergency admission with alcohol-related liver disease between 2008/09 and 2017/18.** Data are shown for in-hospital deaths and cumulative deaths at 365 days from admission.

| Characteristic | In-hospital | | | Within 365 days | | |
|---|---|---|---|---|---|---|
| | Survived N=14,867 | Died N=2,708 | p | Survived N=11,637 | Died N=5,939 | p |
| Age, mean (sd) | 52.6 (12) | 58.1 (12) | ** | 51.4 (12) | 57.5 (12) | ** |
| Age Group | | | | | | |
| <40 | 2,153 (14.5%) | 173 (6.4%) | ** | 1,862 (16.0%) | 464 (7.8%) | ** |
| 40-49 | 4,071 (27.4%) | 463 (17.1%) | | 3,405 (29.3%) | 1,129 (19.0%) | |
| 50-59 | 4,318 (29.0%) | 819 (30.2%) | | 3,421 (29.4%) | 1,716 (28.9%) | |
| 60-69 | 3,018 (20.3%) | 821 (30.3%) | | 2,207 (19.0%) | 1,632 (27.5%) | |
| 70+ | 1,307 (8.8%) | 432 (16.0%) | | 742 (6.4%) | 997 (16.8%) | |
| Sex | | | | | | |
| Female | 4,844 (32.6%) | 976 (36.0%) | ** | 3,857 (33.1%) | 1,963 (33.1%) | ns |
| Male | 10,023 (67.4%) | 1,732 (64.0%) | | 7,780 (66.9%) | 3,975 (66.9%) | |
| Deprivation quintile | | | | | | |
| 1 - Least deprived | 2,040 (13.7%) | 395 (14.6%) | * | 1,544 (13.3%) | 891 (15.0%) | ** |
| 2 | 2,289 (15.4%) | 446 (16.5%) | | 1,767 (15.2%) | 968 (16.3%) | |
| 3 | 2,530 (17.0%) | 498 (18.4%) | | 1,954 (16.8%) | 1,074 (18.1%) | |
| 4 | 3,251 (21.9%) | 586 (21.6%) | | 2,582 (22.2%) | 1,255 (21.1%) | |
| 5 - Most deprived | 4,757 (32.0%) | 783 (28.9%) | | 3,790 (32.6%) | 1,750 (29.5%) | |
| Case definition | | | | | | |
| Primary | 7,595 (51.1%) | 1,779 (65.7%) | ** | 5,732 (49.3%) | 3,642 (61.3%) | ** |
| Uplift | 7,272 (48.9%) | 929 (34.3%) | | 5,905 (50.7%) | 2,296 (38.7%) | |
| Stage of liver disease | | | | | | |
| Fatty liver | 916 (6.2%) | 26 (1.0%) | ** | 818 (7.0%) | 124 (2.1%) | ** |
| Hepatitis | 2,148 (14.4%) | 221 (8.2%) | | 1,812 (15.6%) | 557 (9.4%) | |
| Fibrosis and sclerosis | 55 (0.4%) | 7 (0.3%) | | 48 (0.4%) | 14 (0.2%) | |
| Cirrhosis | 4,220 (28.4%) | 781 (28.8%) | | 2,998 (25.8%) | 2,003 (33.7%) | |
| Hepatic failure | 1,442 (9.7%) | 912 (33.7%) | | 1,045 (9.0%) | 1,309 (22.0%) | |
| Not specified | 6,086 (40.9%) | 761 (28.1%) | | 4,916 (42.2%) | 1,931 (32.5%) | |
| Ascites | 5,179 (34.8%) | 1,410 (52.1%) | ** | 3,580 (30.8%) | 3,009 (50.7%) | ** |
| Varices | 2,191 (14.7%) | 513 (18.9%) | * | 1,609 (13.8%) | 1,095 (18.4%) | ** |
| Comorbidity score (non-liver) | | | | | | |
| 0-1 | 13,368 (89.9%) | 2,138 (79.0%) | ** | 10,736 (92.3%) | 4,770 (80.3%) | ** |
| 2+ | 1,499 (10.1%) | 570 (21.0%) | | 901 (7.7%) | 1,168 (19.7%) | |
| Length of stay, mean (sd) | 11 (15) | 14 (16) | ** | 11 (14) | 13 (16) | ** |
| Higher level care | 773 (5.2%) | 977 (36.1%) | ** | 601 (5.2%) | 1,149 (19.3%) | ** |

* $p<0.05$; ** $p<0.001$ between deaths and survivors; ns= not statistically significant.



For peer review only - http://bmjopen.bmj.com/site/about/guidelines.xhtml

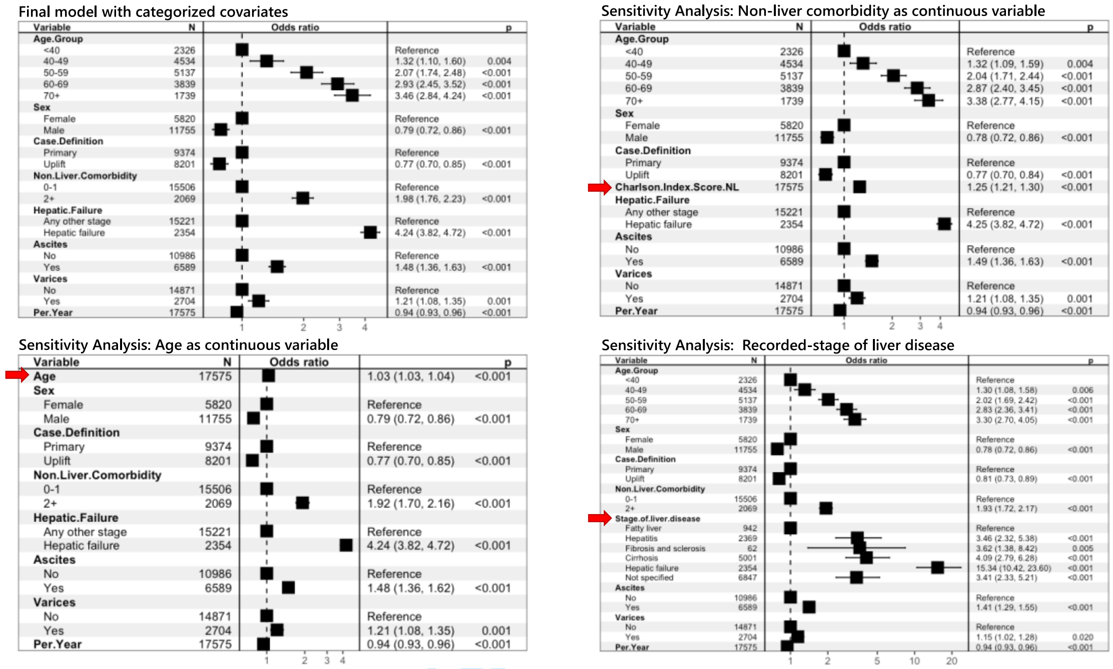


**Supplementary Figure S2. Sensitivity analyses using alternative covariates for risk-adjustment models.** Four alternative models are shown examining factors associated with in-hospital death. The final parsimonious model (upper left) included binary or categorized variables and demonstrates a year-on-year improvement in prognosis (OR 0.94 "per year" through the 10-year observation period). Substituting continuous variables for age (bottom left) or comorbidity score (top right) did not alter the findings (OR 0.94). Similarly, replacing the dichotomous "Hepatic Failure" variable with the individual categories of recorded-stage (bottom right) results in the same conclusions (OR 0.94).

**Supplementary Table S5.** Demographics, clinical characteristics, care processes and outcomes of patients admitted as an emergency for the first time with alcohol-related liver disease between 2008/9 and 2017/18. Stratified by region of residence.

| Characteristic | London N = 2,395 | North West N = 4,058 | Yorkshire & The Humber N = 769 | North East N = 1,113 | West Midlands N = 3,174 | East Midlands N = 416 | East of England N = 642 | South West N = 2,085 | South Central N = 1,648 | South East Coast N = 1,227 |
|---|---|---|---|---|---|---|---|---|---|---|
| Age, mean (SD) | 53.1 (12.0) | 52.5 (12.0) | 51.5 (12.9) | 52.2 (11.9) | 53.7 (12.2) | 52.8 (12.9) | 55.5 (12.5) | 54.1 (12.4) | 54.6 (12.4) | 55.3 (12.9) |
| Female | 717 (30%) | 1,377 (34%) | 259 (34%) | 403 (36%) | 1,052 (33%) | 145 (35%) | 207 (32%) | 694 (33%) | 544 (33%) | 413 (34%) |
| Case definition | | | | | | | | | | |
| Primary | 1,219 (51%) | 2,156 (53%) | 406 (53%) | 601 (54%) | 1,760 (55%) | 211 (51%) | 350 (55%) | 1,121 (54%) | 850 (52%) | 680 (55%) |
| Uplift | 1,176 (49%) | 1,902 (47%) | 363 (47%) | 512 (46%) | 1,414 (45%) | 205 (49%) | 292 (45%) | 964 (46%) | 798 (48%) | 547 (45%) |
| Non-liver comorbidity | | | | | | | | | | |
| 0-1 | 2,115 (88%) | 3,634 (90%) | 678 (88%) | 976 (88%) | 2,818 (89%) | 372 (89%) | 570 (89%) | 1,812 (87%) | 1,441 (87%) | 1,050 (86%) |
| 2+ | 280 (12%) | 424 (10%) | 91 (12%) | 137 (12%) | 356 (11%) | 44 (11%) | 72 (11%) | 273 (13%) | 207 (13%) | 177 (14%) |
| Hepatic failure | 285 (12%) | 675 (17%) | 92 (12%) | 127 (11%) | 438 (14%) | 74 (18%) | 78 (12%) | 217 (10%) | 154 (9.3%) | 205 (17%) |
| Ascites | 836 (35%) | 1,381 (34%) | 291 (38%) | 402 (36%) | 1,311 (41%) | 143 (34%) | 253 (39%) | 804 (39%) | 647 (39%) | 505 (41%) |
| Varices | 379 (16%) | 561 (14%) | 128 (17%) | 134 (12%) | 449 (14%) | 66 (16%) | 117 (18%) | 353 (17%) | 307 (19%) | 205 (17%) |
| Died in hospital | 321 (13.4%) | 605 (14.9%) | 120 (15.6%) | 150 (13.5%) | 549 (17.3%) | 67 (16.1%) | 102 (15.9%) | 295 (14.2%) | 246 (14.9%) | 247 (20.1%) |

For peer review only

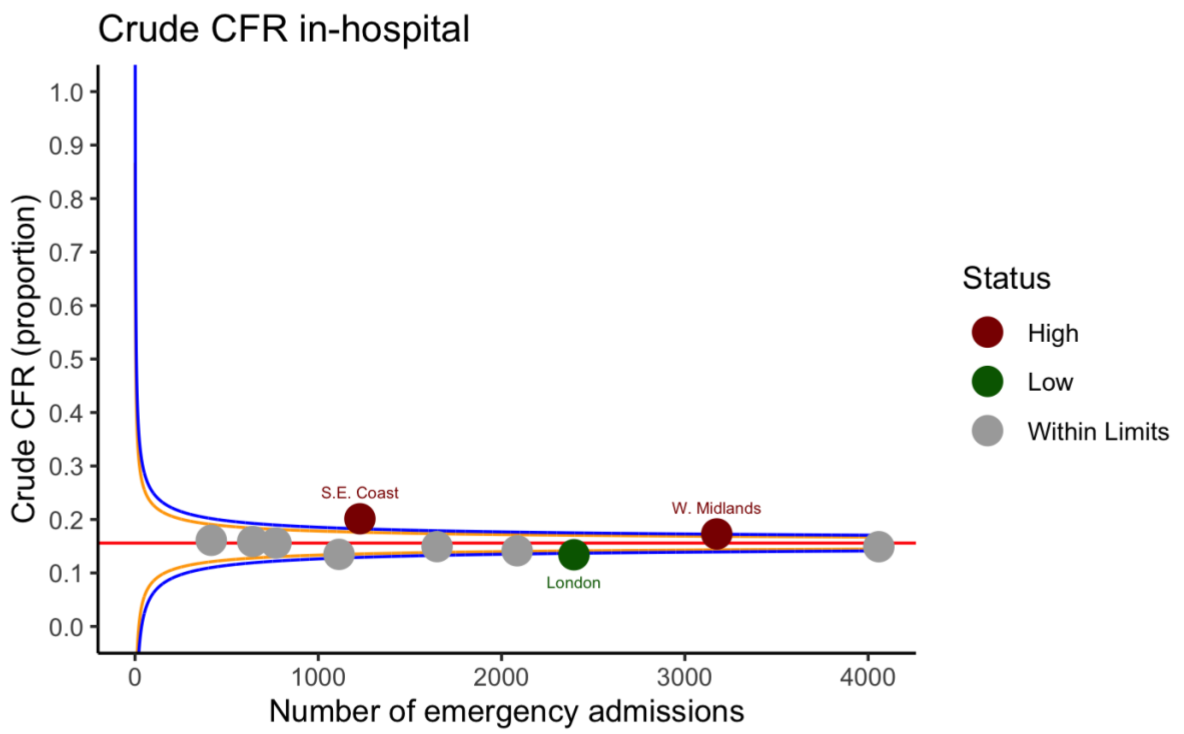


**Supplementary Figure S3 (Exploratory Analysis)**: **Funnel chart showing crude case fatality rate (CFR) in-hospital by region of residence.** Regions marked in red are statistical "outliers" with a higher-than-average crude rate, those in grey are "within limits" and those in green are lower-than-average. The red horizontal line shows average performance across the ten regions. Inner yellow line shows 95% control limits, outer blue line shows 99% control limits based on Spiegelhalter's methodology. Data relate to patients admitted as an emergency for the first time with alcohol-related liver disease (2008/9 and 2017/18), registered with an English CPRD practice. Data tabulated below.



For peer review only - http://bmjopen.bmj.com/site/about/guidelines.xhtml

**Supplementary Table S6 (Exploratory Analysis)**: Association between region of residence and death following first emergency admission for alcohol-related liver disease among people registered with CPRD practices in England, 2008/9 to 2017/18.

| Characteristic | Crude CFR | OR | 95% CI | p | aOR | 95% CI | p |
|---|---|---|---|---|---|---|---|
| London (Ref) | 13.4% | - | - | - | - | - | - |
| North West | 14.9% | 1.13 | 0.98, 1.31 | 0.10 | 1.06 | 0.91, 1.24 | 0.4 |
| Yorkshire & The Humber | 15.6% | 1.19 | 0.95, 1.50 | 0.13 | 1.22 | 0.95, 1.54 | 0.11 |
| North East | 13.5% | 1.01 | 0.82, 1.24 | >0.9 | 1.02 | 0.81, 1.26 | 0.9 |
| **West Midlands** | **17.3%** | **1.35** | **1.16, 1.57** | **<0.001** | **1.27** | **1.09, 1.49** | **0.003** |
| East Midlands | 16.1% | 1.24 | 0.93, 1.64 | 0.14 | 1.13 | 0.83, 1.53 | 0.4 |
| East of England | 15.9% | 1.22 | 0.95, 1.55 | 0.11 | 1.11 | 0.86, 1.43 | 0.4 |
| South West | 14.1% | 1.06 | 0.90, 1.26 | 0.5 | 1.02 | 0.85, 1.22 | 0.8 |
| South Central | 14.9% | 1.13 | 0.95, 1.36 | 0.2 | 1.10 | 0.91, 1.33 | 0.3 |
| **South East Coast** | **20.1%** | **1.63** | **1.36, 1.95** | **<0.001** | **1.36** | **1.12, 1.66** | **0.002** |

OR=unadjusted odds ratio; aOR =risk-adjusted odds ratio; significant values in bold. Unadjusted: C-Statistic 0.536. Adjusted: C-Statistic 0.733. Adjusted for age group, sex, case definition, non-liver comorbidity, hepatic failure, ascites and varices.

For peer review only

**Supplementary Appendix 1.** Medcodes and corresponding readterms used for primary care records to identify entries relating to alcohol misuse and/or alcohol-related liver disease (ARLD). Codes for ARLD are marked with an asterix (*).

| medcode | readterm |
|---|---|
| 322 | Moderate drinker - 3-6u/day |
| 669 | Nondependent alcohol abuse, unspecified |
| 1399 | Alcohol problem drinking |
| 1618 | Heavy drinker - 7-9u/day |
| 2081 | Alcoholism |
| 2082 | Alcohol withdrawal syndrome |
| 2083 | Alcohol detoxification |
| 2084 | Alcohol dependence syndrome |
| 2925 | Alcoholic polyneuropathy |
| 3216 | Acute alcoholic hepatitis* |
| 3782 | Intoxication – alcohol |
| 3782 | Intoxication – alcohol |
| 4500 | Korsakov's alcoholic psychosis |
| 4506 | Alcoholic gastritis |
| 4743 | Alcoholic cirrhosis of liver* |
| 4915 | Alcoholic cardiomyopathy |
| 5611 | [X]Mental and behavioural disorders due to use of alcohol |
| 5740 | Acute alcoholic intoxication in alcoholism |
| 5758 | [X]Chronic alcoholism |
| 6169 | Alcohol dependence syndrome NOS |
| 6467 | [X]Alcoholic hallucinosis |
| 7123 | [V]Personal history of alcoholism |
| 7602 | Chronic alcoholic hepatitis* |
| 7692 | Patient advised about alcohol |
| 7746 | Nondependent alcohol abuse |
| 7885 | Alcoholic liver damage unspecified |
| 7943 | Alcoholic hepatitis* |
| 8030 | [V]Alcohol abuse counselling and surveillance |
| 8363 | Oesophageal varices in alcoholic cirrhosis of the liver* |
| 8388 | [V]Alcohol rehabilitation |
| 8430 | H/O: alcoholism |
| 8984 | Ethyl alcohol causing toxic effect |
| 8999 | Heavy drinker |
| 9169 | [D]Alcohol blood level excessive |
| 9489 | Under care of community alcohol team |
| 9508 | [X]Acute alcoholic drunkenness |
| 9849 | Referral to community alcohol team |
| 10161 | O/E - alcoholic breath |
| 10463 | [X]Intent self poison/exposure to alcohol |
| 10658 | HoNOS item 3 - alcohol/drug problem |

For peer review only



For peer review only - http://bmjopen.bmj.com/site/about/guidelines.xhtml

| | |
|---|---|
| 10691 | Alcoholic fatty liver* |
| 11106 | Korsakov's alcoholic psychosis with peripheral neuritis |
| 11140 | Advice on alcohol consumption |
| 11263 | Wood alcohol causing toxic effect |
| 11491 | Health ed. – alcohol |
| 11670 | [X]Korsakov's psychosis, alcohol induced |
| 11740 | Alcohol misuse - enhanced services administration |
| 12353 | [X]Mental & behav dis due to use alcohol: psychotic disorder |
| 12442 | Alcohol disorder monitoring |
| 12554 | Referral to community drug and alcohol team |
| 12974 | Nondependent alcohol abuse, episodic |
| 12976 | Suspect alcohol abuse – denied |
| 12977 | Very heavy drinker - >9u/day |
| 12982 | Alcohol intake above recommended sensible limits |
| 12984 | Very heavy drinker |
| 12985 | Moderate drinker |
| 13709 | Blood ethanol level |
| 14422 | Urine ethanol |
| 15503 | Accidental poisoning by other ethyl alcohol and its products |
| 16225 | Alcohol withdrawal delirium |
| 16237 | Alcoholic psychoses |
| 16587 | [V]Problems related to lifestyle alcohol use |
| 17149 | [V]Blood-alcohol and blood-drug test |
| 17259 | [X]Delirium tremens, alcohol induced |
| 17330 | Alcoholic hepatic failure* |
| 17607 | [X]Alcoholic psychosis NOS |
| 18156 | Alcoholics anonymous |
| 18252 | Accidental poisoning by alcohol, NEC |
| 18711 | Lifestyle advice regarding alcohol |
| 19217 | Alcohol causing toxic effect |
| 19401 | Binge drinker |
| 19489 | Alcohol consumption screen |
| 19494 | Hazardous alcohol use |
| 19754 | Serum ethanol level |
| 20514 | [X]Mental and behav dis due to use alcohol: withdrawal state |
| 20762 | Alcohol amnestic syndrome |
| 21624 | Episodic acute alcoholic intoxication in alcoholism |
| 21650 | Admitted to alcohol detoxification centre |
| 21713 | Alcoholic fibrosis and sclerosis of liver* |
| 21879 | [X]Mental and behav dis due to use of alcohol: harmful use |
| 22707 | Drinking problem scale |
| 23610 | Nondependent alcohol abuse, continuous |
| 23978 | [X]Evid of alcohol involv determind by level of intoxication |
| 24064 | Continuous chronic alcoholism |


For peer review only - http://bmjopen.bmj.com/site/about/guidelines.xhtml

| | |
|---|---|
| 24735 | O/E - breath - alcohol smell |
| 24984 | Alcohol-induced chronic pancreatitis |
| 25110 | Alcohol withdrawal hallucinosis |
| 26106 | Episodic chronic alcoholism |
| 26323 | [X]Alcoholic dementia NOS |
| 27342 | Alcoholic dementia NOS |
| 27518 | Hangover (alcohol) |
| 28150 | Nondependent alcohol abuse NOS |
| 28780 | [X]Alcohol addiction |
| 29691 | Aversion therapy - alcoholism |
| 30036 | [X]Poisoning/exposure, ? intent, to alcohol |
| 30162 | [X]Alcoholic paranoia |
| 30404 | Alcoholic paranoia |
| 30460 | Alcoholism counselling |
| 30604 | Alcohol-induced epilepsy |
| 30695 | Harmful alcohol use |
| 31443 | Chronic alcoholism |
| 31605 | [X]Accident poisoning/exposure to alcohol |
| 31742 | Alcoholic myopathy |
| 32850 | Alcohol use disorders identification test |
| 32927 | [X]Alcohol withdrawal-induced seizure |
| 32964 | Alcohol abuse monitoring |
| 33635 | Chronic alcoholism NOS |
| 33670 | Other alcoholic psychosis |
| 33839 | Cerebellar ataxia due to alcoholism |
| 35330 | Alcohol consumption counselling |
| 35859 | Pregnancy alcohol advice |
| 36296 | Acute alcoholic intoxication in alcoholism NOS |
| 36499 | Alcohol causing toxic effect NOS |
| 36714 | Ethanol causing toxic effect |
| 36748 | Alcoholic encephalopathy |
| 37264 | Alcohol leaflet given |
| 37691 | [X]Chronic alcoholic brain syndrome |
| 37946 | Chronic alcoholic brain syndrome |
| 38012 | Accidental poisoning by ethanol, NOS |
| 38061 | Alcohol induced hallucinations |
| 39327 | [X]Mental and behav dis due to use alcohol: dependence syndr |
| 39799 | [X]Mental and behav dis due to use alcohol: amnesic syndrome |
| 40530 | Acute alcoholic intoxication, unspecified, in alcoholism |
| 40541 | Accidental poisoning by alcoholic beverages |
| 40602 | ADS - Alcohol dependence scale |
| 41251 | BMAST - Brief Michigan alcoholism screening test |
| 41638 | [X]Int self poison/exposure to alcohol at home |
| 41920 | Alcohol amnestic syndrome NOS |

For peer review only

| | |
|---|---|
| 41983 | Alcohol detoxification |
| 42305 | Severity of alcohol dependence questionnaire |
| 43193 | Unspecified chronic alcoholism |
| 43813 | Police:venesect-alcohol |
| 44299 | [X]Mental & behav dis due to use alcohol: acute intoxication |
| 44783 | Pain in lymph nodes after alcohol consumption |
| 45169 | [X]Men & behav dis due to use alcohol: oth men & behav dis |
| 46677 | Alcohol withdrawal regime |
| 46684 | Plasma ethanol level |
| 47123 | Alcohol counselling by other agencies |
| 47555 | Cerebral degeneration due to alcoholism |
| 48514 | Denatured alcohol causing toxic effect |
| 48545 | Alcohol questionnaire completed |
| 50507 | Alcohol dependence scale |
| 53139 | Accidental poisoning by other alcohols |
| 54209 | Advice to change alcohol intake |
| 54505 | Other alcoholic dementia |
| 55415 | Accidental poisoning by alcohol NOS |
| 55536 | Ethyl alcohol causing toxic effect NOS |
| 56410 | Delivery of rehabilitation for alcohol addiction |
| 56441 | Michigan alcoholism screening test |
| 56947 | Continuous acute alcoholic intoxication in alcoholism |
| 57714 | Alcohol dependence with acute alcoholic intoxication |
| 57939 | Pathological alcohol intoxication |
| 59414 | [X]Intent self poison alcohol unspecif place |
| 59776 | Last six months of drinking questionnaire |
| 59873 | AUDIT - Alcohol use disorders identification test |
| 61187 | [X]Accid poison/expos to alcohol unspecif place |
| 61190 | [X]Pois/expos ?intent to alcohol unspecif place |
| 61583 | [V]Medicolegal blood alcohol test |
| 61750 | Comprehensive drinker profile |
| 62299 | SADD - Short alcohol dependence data |
| 62300 | Short alcohol dependence data |
| 63306 | [X]Accident poison/exposure to alcohol at home |
| 63457 | CDP - Comprehensive drinker profile |
| 63529 | Alcohol misuse - enhanced service completed |
| 63876 | [X]Accid poison/expos alcohol in street/highway |
| 64389 | [X]Ment & behav dis due use alcohol: unsp ment & behav dis |
| 65754 | Alcohol-induced pseudo-Cushing's syndrome |
| 65932 | [X]Alcoholic jealousy |
| 66831 | SADQ - Severity of alcohol dependence questionnaire |
| 67651 | Alcoholic psychosis NOS |
| 68111 | Other alcoholic psychosis NOS |
| 68159 | [X]Poison/exposure ?intent, to alcohol at home |



For peer review only - http://bmjopen.bmj.com/site/about/guidelines.xhtml

| | |
|---|---|
| 69331 | MAST - Michigan alcoholism screening test |
| 69407 | [X]Pois/exp ?intent alcohol school/pub admin area |
| 70939 | Munich alcoholism test |
| 84218 | Disqualified from driving due to excess alcohol |
| 90714 | Alcohol screen - AUDIT completed |
| 92908 | [X]Accid poison/expos alcohol trade/service area |
| 93415 | Alcohol units per week |
| 93624 | Fast alcohol screening test |
| 94485 | Alcohol screen - AUDIT PC completed |
| 94553 | Referral to specialist alcohol treatment service |
| 94669 | Alcohol consumption screening test declined |
| 94670 | Alcohol misuse |
| 94838 | Alcohol use disorders identification test |
| 94963 | Alcohol screen - fast alcohol screening test completed |
| 95181 | Alcohol reduction programme |
| 95650 | Advice to change drink intake |
| 95663 | Alcohol screen - AUDIT C completed |
| 95744 | Alcohol use disorder identificatn test consumptn questionnre |
| 95944 | Alcohol assesment declined - enhanced services admin |
| 96053 | Brief intervention for excessive alcohol consumptn completed |
| 96054 | Extended intervention for excessive alcohol consumptn complt |
| 96107 | Single alcohol screening questionnaire |
| 96219 | [X]Pois/expos ?intent alcohol in street/highway |
| 96259 | Alcohol assessment declined |
| 96993 | Referral to alcohol brief intervention service |
| 97085 | [X]Int self poison alcohol other spec place |
| 97126 | Alcohol units consumed on heaviest drinking day |
| 97163 | Advice to change alcoholic drink intake |
| 97163 | Advice to change alcoholic drink intake |
| 97261 | Brief intervention for excessive alcohol consumptn declined |
| 97309 | Advised to contact primary care alcohol worker |
| 97680 | Declined referral to specialist alcohol treatment service |
| 99877 | Feels should cut down drinking |
| 99985 | Brief Michigan alcoholism screening test |
| 100493 | Five-shot questionnaire on heavy drinking |
| 100989 | Breath alcohol level |
| 101426 | Accidental poisoning by ethyl alcohol NOS |
| 101543 | Other alcohol causing toxic effect |
| 101718 | Drinks in morning to get rid of hangover |
| 102121 | Police:venesect-alcohol |
| 102247 | Extended interven for excessive alcohol consumption declined |
| 102448 | Higher risk drinking |
| 102564 | Advised to abstain from alcohol consumption |
| 102577 | Severity of alcohol dependence questionnaire |



For peer review only - http://bmjopen.bmj.com/site/about/guidelines.xhtml

| | |
|---|---|
| 102665 | Increasing risk drinking |
| 102770 | SADQ - Severity of alcohol dependence questionnaire |
| 103459 | Referral to community alcohol team declined |
| 104458 | Breath ethanol level |
| 104611 | Alcohol-induced acute pancreatitis |
| 104702 | [X]Accid pois/expos alcohol in sport/athletic area |
| 104734 | [X]Accid pois/expos to alcohol other spec place |
| 108205 | Clinical Institute Withdrawal Assessment for Alcohol, revisd |
| 108644 | In-house alcohol detoxification |
| 109241 | Alcohol Use Disorders Identification Test declined |
| 109668 | Specialist alcohol treatment service signposted |
| 109675 | Hospital alcohol liaison team report received |
| 109800 | Hospital attendance related to personal alcohol consumption |
| 110494 | Alcohol harm reduction programme |
| 110624 | Alcohol relapse prevention |
| 110911 | Accidental poisoning by denatured alcohol |
| 111683 | Removal of alcohol |
| 111933 | Family wellbeing discussion about alcohol |
| 112448 | [X]Pois/expos ?intent to alcohol at res institut |
| 112645 | [X]Intent self pois alcohol in street/highway |
| 112648 | [X]Acc pois/expos alcohol indust/construct area |

For peer review only

**Supplementary Appendix 2.** Methods used for screening of "cause" fields listed on the death certificate among patients dying during index admission for ARLD.

Screening was applied to codes recorded in any "cause" field on the death certificates of in-hospital fatalities. To be flagged as a "cause-specific" death from ARLD, we required the presence of both liver and alcohol descriptor in one or more coded causes.

Hence, this included either one of K70.x codes or the appearance of a non-specific liver code in combination with at least one other alcohol-specific condition on the certificate (please refer to code lists for the LAA).

The commonest recorded K70.x code in the *first field* (cause_label) was K70.9 ALCOHOLIC LIVER DISEASE, UNSPECIFIED (accounting for 24.7% of in-hospital deaths), followed by K70.4 ALCOHOLIC HEPATIC FAILURE (20.6%), K70.3 ALCOHOLIC CIRRHOSIS OF LIVER (12.1%), K70.1 ALCOHOLIC HEPATITIS (6.0%) AND K70.0 ALCOHOLIC FATTY LIVER (0.4%). The commonest non-specific liver disease codes recorded in the *first field* (cause_label) were K74.6 OTHER AND UNSPECIFIED CIRRHOSIS OF LIVER (6.8%), K72.9 HEPATIC FAILURE, UNSPECIFIED (3%).

For peer review only

STROBE Statement—checklist of items that should be included in reports of observational studies

| | Item No | Recommendation |
|---|---|---|
| **Title and abstract** | 1 | (*a*) Indicate the study's design with a commonly used term in the title or the abstract [Done] |
| | | (*b*) Provide in the abstract an informative and balanced summary of what was done and what was found. Page 1 |
| **Introduction** | | |
| Background/rationale | 2 | Explain the scientific background and rationale for the investigation being reported. Page 3 (para 1-3) |
| Objectives | 3 | State specific objectives, including any prespecified hypotheses. Page 3 (para 4) |
| **Methods** | | |
| Study design | 4 | Present key elements of study design early in the paper. Page 3-4 |
| Setting | 5 | Describe the setting, locations, and relevant dates, including periods of recruitment, exposure, follow-up, and data collection. Page 3-4 |
| Participants | 6 | (*a*) *Cohort study*—Give the eligibility criteria, and the sources and methods of selection of participants. Describe methods of follow-up. Page 4 (para 2-3)<br>*Case-control study*—Give the eligibility criteria, and the sources and methods of case ascertainment and control selection. Give the rationale for the choice of cases and controls<br>*Cross-sectional study*—Give the eligibility criteria, and the sources and methods of selection of participants |
| | | (*b*) *Cohort study*—For matched studies, give matching criteria and number of exposed and unexposed<br>*Case-control study*—For matched studies, give matching criteria and the number of controls per case |
| Variables | 7 | Clearly define all outcomes, exposures, predictors, potential confounders, and effect modifiers. Give diagnostic criteria, if applicable Page 4-6 |
| Data sources/ measurement | 8* | For each variable of interest, give sources of data and details of methods of assessment (measurement). Describe comparability of assessment methods if there is more than one group. Page 4-5 |
| Bias | 9 | Describe any efforts to address potential sources of bias. Page 5-6 |
| Study size | 10 | Explain how the study size was arrived at. N/A all eligible cases were included |
| Quantitative variables | 11 | Explain how quantitative variables were handled in the analyses. If applicable, describe which groupings were chosen and why Page 6 |
| Statistical methods | 12 | (*a*) Describe all statistical methods, including those used to control for confounding Page 6 |
| | | (*b*) Describe any methods used to examine subgroups and interactions. Page 6 |
| | | (*c*) Explain how missing data were addressed. N/A No missing data |
| | | (*d*) *Cohort study*—If applicable, explain how loss to follow-up was addressed. N/A. Outcome was known for all cases, tracked for 365 days from admission.<br>*Case-control study*—If applicable, explain how matching of cases and controls was addressed<br>*Cross-sectional study*—If applicable, describe analytical methods taking account of sampling strategy |
| | | (*e*) Describe any sensitivity analyses. Pages 6, 10 and Supplementary Materials |

Continued on next page

For peer review only

| | | |
|---|---|---|
| **Results** | | |
| Participants | 13* | (a) Report numbers of individuals at each stage of study—eg numbers potentially eligible, examined for eligibility, confirmed eligible, included in the study, completing follow-up, and analysed. Page 7 and Table 1, Suppl. Tables |
| | | (b) Give reasons for non-participation at each stage. N/A |
| | | (c) Consider use of a flow diagram. N/A |
| Descriptive data | 14* | (a) Give characteristics of study participants (eg demographic, clinical, social) and information on exposures and potential confounders. Page 7, Table 1, Suppl. Tables |
| | | (b) Indicate number of participants with missing data for each variable of interest. N/A |
| | | (c) *Cohort study*—Summarise follow-up time (eg, average and total amount). Page 5 - all cases were followed-up for 365 days post-admission (or death). Table 2 and Figure 2 |
| Outcome data | 15* | *Cohort study*—Report numbers of outcome events or summary measures over time. Page 9-10, Table 1, Figure 2, Suppl. Fig. 1, Suppl. Figs S2-S4 |
| | | *Case-control study*—Report numbers in each exposure category, or summary measures of exposure |
| | | *Cross-sectional study*—Report numbers of outcome events or summary measures |
| Main results | 16 | (*a*) Give unadjusted estimates and, if applicable, confounder-adjusted estimates and their precision (eg, 95% confidence interval). Make clear which confounders were adjusted for and why they were included. Page 10, Table 2 and Suppl. Figures |
| | | (*b*) Report category boundaries when continuous variables were categorized |
| | | (*c*) If relevant, consider translating estimates of relative risk into absolute risk for a meaningful time period. N/A |
| Other analyses | 17 | Report other analyses done—eg analyses of subgroups and interactions, and sensitivity analyses. Page 10 and Supplementary Materials |
| **Discussion** | | |
| Key results | 18 | Summarise key results with reference to study objectives. Page 11 |
| Limitations | 19 | Discuss limitations of the study, taking into account sources of potential bias or imprecision. Discuss both direction and magnitude of any potential bias. Page 12-13 |
| Interpretation | 20 | Give a cautious overall interpretation of results considering objectives, limitations, multiplicity of analyses, results from similar studies, and other relevant evidence. Page 11-12 |
| Generalisability | 21 | Discuss the generalisability (external validity) of the study results. Page 13, para 2 |
| **Other information** | | |
| Funding | 22 | Give the source of funding and the role of the funders for the present study and, if applicable, for the original study on which the present article is based. Page 13 |

*Give information separately for cases and controls in case-control studies and, if applicable, for exposed and unexposed groups in cohort and cross-sectional studies.

**Note:** An Explanation and Elaboration article discusses each checklist item and gives methodological background and published examples of transparent reporting. The STROBE checklist is best used in conjunction with this article (freely available on the Web sites of PLoS Medicine at http://www.plosmedicine.org/, Annals of Internal Medicine at http://www.annals.org/, and Epidemiology at http://www.epidem.com/). Information on the STROBE Initiative is available at www.strobe-statement.org.

For peer review only

BMJ Open

# Outcomes of first emergency admissions for alcohol-related liver disease in England over a ten year period: Retrospective observational cohort study using linked electronic databases

| | |
|---|---|
| Journal: | *BMJ Open* |
| Manuscript ID | bmjopen-2023-076955.R2 |
| Article Type: | Original research |
| Date Submitted by the Author: | 01-Nov-2023 |
| Complete List of Authors: | Bodger, Keith; University of Liverpool, Dept. of Health Data Science, Insitute of Population Health; Liverpool University Hospitals NHS Foundation Trust, Gastroenterology Dept.<br>Mair, Thomas; University of Liverpool, Dept. of Health Data Science, Insitute of Population Health<br>Schofield, Peità; University of Liverpool, Department of Public Health & Policy<br>Silberberg, Benjamin; University of Liverpool, Dept. of Health Data Science, Insitute of Population Health<br>Hood, Steve; Liverpool University Hospitals NHS Foundation Trust, Gastroenterology Dept.<br>Fleming, Kate M; NHS England, Data & Analytics Transformation Directorate |
| <b>Primary Subject Heading</b>: | Gastroenterology and hepatology |
| Secondary Subject Heading: | Gastroenterology and hepatology |
| Keywords: | Electronic Health Records, Hospitalization, EPIDEMIOLOGY, Hepatology < INTERNAL MEDICINE, Primary Care < Primary Health Care |
| | |

SCHOLARONE™
Manuscripts

*I, the Submitting Author has the right to grant and does grant on behalf of all authors of the Work (as defined in the below author licence), an exclusive licence and/or a non-exclusive licence for contributions from authors who are: i) UK Crown employees; ii) where BMJ has agreed a CC-BY licence shall apply, and/or iii) in accordance with the terms applicable for US Federal Government officers or employees acting as part of their official duties; on a worldwide, perpetual, irrevocable, royalty-free basis to BMJ Publishing Group Ltd ("BMJ") its licensees and where the relevant Journal is co-owned by BMJ to the co-owners of the Journal, to publish the Work in this journal and any other BMJ products and to exploit all rights, as set out in our licence.*

*The Submitting Author accepts and understands that any supply made under these terms is made by BMJ to the Submitting Author unless you are acting as an employee on behalf of your employer or a postgraduate student of an affiliated institution which is paying any applicable article publishing charge ("APC") for Open Access articles. Where the Submitting Author wishes to make the Work available on an Open Access basis (and intends to pay the relevant APC), the terms of reuse of such Open Access shall be governed by a Creative Commons licence – details of these licences and which Creative Commons licence will apply to this Work are set out in our licence referred to above.*

*Other than as permitted in any relevant BMJ Author's Self Archiving Policies, I confirm this Work has not been accepted for publication elsewhere, is not being considered for publication elsewhere and does not duplicate material already published. I confirm all authors consent to publication of this Work and authorise the granting of this licence.*

For peer review only

# Outcomes of first emergency admissions for alcohol-related liver disease in England over a ten year period: Retrospective observational cohort study using linked electronic databases

Keith Bodger[1,3], Thomas Mair[1], Pieta Schofield[2], Benjamin Silberberg[1], Steve Hood[3], Kate M Fleming[4]

1. Dept. of Health Data Science, Insitute of Population Health, University of Liverpool, UK
2. Dept. of Public Health & Policy, Insitute of Population Health, University of Liverpool, UK
3. Gastroenterology Dept., Liverpool University Hospitals NHS Foundation Trust, UK
4. National Disease Registration Service, Data & Analytics Transformation Directorate, NHS England, UK

## Abstract

**Objectives:** To examine time trends in patient characteristics, care processes and case fatality of first emergency admission for alcohol-related liver disease (ARLD) in England.

**Design:** National population-based, retrospective observational cohort study.

**Setting:** Clinical Practice Research Datalink population of England, 2008/9-17/18. First emergency admissions were identified using the Liverpool ARLD Algorithm. We applied survival analyses and binary logistic regression to study prognostic trends.

**Outcome measures:** Patient characteristics; "recent" GP consultations and hospital admissions (preceding year); higher level care; deaths in-hospital (including certified cause) and within 365 days. Covariates were age, sex, deprivation status, coding pattern, ARLD stage, non-liver comorbidity, coding for ascites and varices.

**Results:** 17,575 first admissions (mean age: 53 years; 33% female; 32% from most deprived quintile). Almost half had codes suggesting advanced liver disease. In year before admission, only 47% of GP consulters had alcohol-related problems recorded; alcohol-specific diagnostic codes were absent in 24% of recent admission records. Overall, case fatality rate was 15% in-hospital and 34% at one year. Case-mix-adjusted odds of in-hospital death reduced by 6% per year (aOR: 0.94; 95% CI: 0.93-0.96) and 4% per year at 365 days (aOR: 0.96; 95% CI: 0.95-0.97). Exploratory analyses suggested the possibility of regional inequalities in outcome.

**Conclusions:** Despite improving prognosis of first admissions, we found missed opportunities for earlier recognition and intervention in primary and secondary care. In 2017/18, one in seven were still dying during index admission, rising to one third within a year. Nationwide efforts are needed to promote earlier detection and intervention, and to minimise avoidable mortality after first emergency presentation. Regional variation requires further investigation.

**Keywords:** Liver Diseases, Alcoholic; Algorithms; Epidemiology; Primary Health Care; Electronic Health Records; Hospitalization; Hospital Mortality; Emergency Service; Quality of Health Care

**Corresponding author:** Keith Bodger, Reader & Consultant Gastroenterologist (Honorary), Department of Health Data Science, Institute of Population Health, University of Liverpool, kbodger@liverpool.ac.uk



For peer review only - http://bmjopen.bmj.com/site/about/guidelines.xhtml

## STRENGTHS AND LIMITATIONS OF THIS STUDY

- Nationwide population-based study sample covering all regions of England
- Application of a novel cohort discovery algorithm and the use of linked datasets covering primary and secondary care and death certification
- The datasets lack some important case mix information such as physiological or biochemical measures of disease severity which would allow for further risk-adjustment of case fatality rates.
- The cases are drawn from a large, representative sample of the English population but the findings may not be generalisable to all parts of the country.

For peer review only



For peer review only - http://bmjopen.bmj.com/site/about/guidelines.xhtml

## Introduction

Alcohol-related liver disease (ARLD) is a leading cause of premature death and is set to overtake ischaemic heart disease for working years-of-life-lost in the UK.(1) Major shortcomings in acute hospital treatment were highlighted by the National Confidential Enquiry into Patient Outcome and Death (NCEPOD).(2) This widely publicised enquiry, combined with reports from the *Lancet Standing Commission* on Liver Disease,(3) prompted UK initiatives to promote better care.(4–8) Similar calls for action have been raised in other countries.(9) However, in their final report the commission's authors highlighted continuing concerns about late diagnosis and unacceptably high levels of mortality in hospitalised patients.(10)

A patient's first emergency hospital admission is often the point of initial diagnosis.(11) High quality care is crucial to minimise early mortality and allow time for engagement of services promoting abstinence and recovery. However, knowledge of characteristics and outcomes of first admissions is limited. Nor is it known whether case-mix has changed or prognosis improved over time.

Hospital administrative data is the main source of national statistics (12) and underpins proposed metrics.(13) However, identifying and characterising cases using routine data is challenging.(11) Patients present to hospital with varied clinical manifestations and co-existing alcohol-related conditions.(14) Analysis of administrative data in our region revealed that standard case definitions (based on primary discharge diagnoses alone)(12) identified only half of true admissions.(11) We developed an algorithm to identify combinations of primary and secondary codes compatible with acute presentations of ARLD.(11) Recent national testing confirmed a doubling of estimates of annual admissions using this method,(15) representing an under-recording of NHS hospital activity of £70-127 million for 2020.(15) This prompted calls for revising methods for national statistics.(16)

We undertook a retrospective cohort study using linked electronic datasets and the Liverpool ARLD algorithm (LAA).(11,15) We aimed to describe sociodemographics, clinical features, care processes (prior healthcare contacts in preceding year, length of stay and provision of higher level care) and case fatality of patients presenting with their first emergency admission for ARLD in England. We examined trends over a ten year period, spanning five years before and after the NCEPOD report.(2) We also undertook exploratory analyses of regional variation in admission outcome.

## Methods

### Data Sources

The cohort was derived from the Clinical Practice Research Datalink (CPRD) containing linked data from primary and secondary care and death certification (Office for National Statistics).(17) This anonymized database covers a broadly representative

For peer review only

sample of UK residents. The primary care consultations records contain diagnoses encoded as READ codes. Each case in the present study had linkage to Hospital Episode Statistics (HES), comprising administrative data for inpatient care delivered in NHS hospitals (available for the period April 1997 to March 2021). Care episodes in HES contain diagnostic codes generated after discharge using the international classification of diseases (ICD-10) system. The dataset also included the Adult Critical Care Minimum dataset.

**Population of interest: First emergency admissions for ARLD**

We extracted data for all adult patients (≥18 years) with a first unplanned (non-elective) hospital admission (spell) for ARLD. We defined a "first" (or index) admission as having no such emergency admission within the preceding ten years. We focused on prior emergency hospitalisations (i.e. we did not screen elective admissions). We included all completed index emergency discharge records between 1st April 2008 and 31st March 2018, thereby covering ten fiscal years (2008/9 to 2017/18). The cohort was restricted to residents with a record of CPRD practice registration ≥1 year.

**Case definitions for ARLD admissions**

We used the LAA to identify admissions, requiring discharge codes to comply with one of two alternative case definitions ('primary' or 'uplift' subgroups).(11) The primary definition selects cases with a K70.x code as primary diagnosis, as in national statistics.(12) The 'uplift' definition identifies additional admissions with non-primary coding patterns (11,15) Briefly, uplift cases have a primary code for either a symptom, sign or complication of liver disease or for another alcohol-specific condition, accompanied by secondary codes compatible with ARLD (either K70.x or code for unspecified liver disease provided additional alcohol-specific conditions are listed).

**Stage of liver disease**

We further characterised cases by assigning "recorded-stage" of liver disease based on the K70.x code present in the index emergency admission (i.e. alcoholic fatty liver [K70.0], hepatitis [K70.1], sclerosis and fibrosis [K70.2], cirrhosis [K70.3], hepatic failure [K70.4] or "unspecified" [K70.9]). All primary and most uplift admission records contain a K70.x code. A minority of uplift cases lack a K70.x code, whereby a non-specific liver disease code (e.g. "Hepatic failure, unspecified" or "Other and unspecified cirrhosis of liver") is accompanied by codes for other alcohol-specific conditions. In these cases, the relevant stage was taken from the liver code descriptor.

**Patient-level covariates derived from the index admission**

Covariates were age, age group, sex, ethnicity, deprivation status, case definition (primary or uplift), recorded-stage of liver disease, coding for specific features of advanced liver disease (varices and ascites) and comorbidities. Socioeconomic deprivation was defined by the English indices of deprivation 2015 and grouped into quintiles (1 = Least deprived, 5 = Most deprived), as described previously.(11,18)


For peer review only - http://bmjopen.bmj.com/site/about/guidelines.xhtml

Comorbidities listed in the Charlson Comorbidity Index (CCI)(19) were identified but we excluded liver disease codes (mild and moderate-to-severe liver disease categegories) and dichotomised cases into two groups (scores 0-1 and ≥2).

**Contacts in the year prior to index admission**
The earliest stage of ARLD (fatty liver) may develop over months but more severe liver disease (hepatitis, cirrhosis or liver failure) requiring hospitalisation requires several years of harmful drinking.(14,20) We focused on contacts within a year before index emergency admission, assuming all such patients would have an established history of alcohol excess. The absence of relevant codes within such encounters would be taken as a marker of "missed opportunities" to screen, detect and/or intervene during the year before liver-related emergency hospitalization.

**Consultations in primary care** - All available primary care contacts within the prior year were extracted and screened. For each contact, we created two binary variables. Firstly, a variable to indicate whether any alcohol-related code was present – signifying whether alcohol misuse or harms were suspected/recognised and recorded at the time. We used an extensive list of READ codes (See **Supplementary Appendix 1**). Second, whether a diagnosis of liver disease was recorded – a marker of prior suspicion, recognition or elective diagnosis of ARLD.

**Emergency hospital admissions** - We identified all episodes of unplanned hospital care in the year prior to index emergency admission and screened the diagnosis fields for any alcohol-specific ICD-10 code. Hence, we determined whether any non-liver related alcohol problems were diagnosed and recorded during those prior admissions.

**Care processes**
We determined length of hospital stay. Using the Adult Critical Care Minimum Dataset we determined whether the patient received higher level care during their index admission, including high dependency (category 2) and intensive care (category 3).

**Time periods of interest**
We defined two 5-year time periods to compare cases discharged in the first and second half of the decade (Period 1: April 2008 to March 2013; Period 2: April 2013 to March 2018). We also created a variable to indicate fiscal year of discharge.

**Outcomes**
We examined all-cause case fatality identified from death certification. We created binary variables for death during index emergency admission (in-hospital death) and tracked all cases for death within 365-days. For survival analysis we calculated time-to-death (days from admission). We used case fatality rates, as opposed to population-based relative mortality measures, since our purpose was to generate


For peer review only - http://bmjopen.bmj.com/site/about/guidelines.xhtml

metrics focused on effectiveness of acute care after adjusting for severity of presenting illness.

### Certified cause of in-hospital death

We accessed linked records of death certification for all in-hospital deaths during index admissions to identify deaths attributable to ARLD. This was based on screening all available "cause" fields for descriptors relating to deaths attributable to liver disease and alcohol. See **Supplementary Appendix 2** for details.

### Statistical analyses

First, we summarised characteristics overall and for relevant strata and examined time trends. Categorical variables are reported as counts and percentages (groups compared using Chi Squared statistics). Continuous variables are reported as mean (sd), or median (IQR), as appropriate with comparisons made using parametric (t-test or ANOVA) or non-parametric tests (Mann-Whitney U or Kruskal-Wallis).

Second, we calculated crude case fatality rates (CFR) as count of deaths (numerator) divided by number of first admissions (denominator) per time period. We report deaths in-hospital and at 365 days. We compared 5-year periods (Chi-square Test) and significance of monotonic year-on-year trends (Mann Kendall Trend Test). Kaplan-Meier survival analysis compared cumulative probability of death up to 365 days, using log-rank test to determine significance between strata. All cases had at least one year follow-up (no censoring).

Third, we determined factors associated with death using binary logistic regression. Models were used to adjust CFR for significant covariates. The dependent variables were all-cause death (either in-hospital or at 365 days). Covariates were added sequentially based on clinical relevance, previous research(11) and significant univariate associations ($p<0.10$), starting with age and sex and building a series of models to explore alternative risk-adjustment approaches. We generated time series of risk-adjusted CFRs by calculating standardised mortality ratios (SMR) for each time period, dividing the number of observed deaths by the number of expected deaths (sum of probabilities from relevant model). Each model used pooled data across the ten year period to allow comparisons over time. Risk-adjusted rates were calculated by multiplying the crude rate across 10 years by the SMR for each period (equivalent to indirect standardisation). The C-statistic and AIC values were calculated to assess model fit.

Fourth, we tested significance of national time trends by introducing five-year period or fiscal year of discharge into models. For year-on-year trends, the year variable was treated as continuous, hence odds ratios reflect average change *per one-year increment* across the decade (assuming a linear trend). Sensitivity analyses were undertaken using variants of model covariates (continuous rather than categorical



For peer review only - http://bmjopen.bmj.com/site/about/guidelines.xhtml

variables for age and non-liver comorbidity score; recorded-stage of liver disease rather than binary variable).

Finally, we undertook exploratory analysis of regional variation focusing on the main outcome of in-hospital case fatality. CPRD region of residence was based on Strategic Health Authority boundaries. We used Funnel Plots to evaluate variation in crude rates and added region to models to compare risk-adjusted CFR.

All analyses were undertaken in R (Version 4.1.2, R Core Team 2021, R Foundation for statistical computing, Vienna, Austria)

### Data Governance and Ethics Statements

The CPRD has obtained ethical approval from the UK's National Research Ethics Service (NRES) Committee for observational research using anonymised data. Our study was approved by the Independent Scientific Advisory Committee (ISAC) for Medicines and Healthcare Products Regulatory Agency (MHRA) database research (protocol number 19_133). Patient consent for publication is not applicable.

## Patient and Public Involvement

Patients or the public were not involved in the design, conduct, reporting or dissemination plans of our research.

## Results

### Characteristics of first emergency admissions

The algorithm detected 17,575 index emergency admissions with characteristics summarised in **Table 1** (overall, and by case definition). 9,374 (53%) were in the primary subgroup and 8,201 (47%) in the uplift subgroup (non-primary coding patterns). The magnitude of 'uplift' is consistent with previous findings.(11,15) The mean age was 53.4 years (range: 18-98), one third (33.1%) were female, 32% were residents of most deprived quintile. Less than 5% had non-white ethnicity recorded (small numbers precluded more detailed reporting).

**Prior primary care consultations** – 9,936 patients had a record of ≥1 GP consultation in the year before admission (**Table 1**). Of this subgroup, 4,702 (47.3%) had a READ code relating to either alcohol consumption or any alcohol-specific condition recorded at that time, suggesting harmful drinking remained undetected or unrecorded in just over half of those encounters. Only 1,465 (14.7% of 'recent' GP consulters) had a code relevant to liver disease recorded at the time, suggesting that ARLD usually remained unsuspected or undiagnosed during these consultations.

**Prior emergency admissions** – forty one percent of the cohort (7,265 patients) had been admitted as an emergency in preceding year. Of these, 5,525 (76%) had a non-


For peer review only - http://bmjopen.bmj.com/site/about/guidelines.xhtml

liver-related, alcohol-specific condition recorded in their list of discharge diagnoses. Hence, one in four (24%) admitted cases had no record of an alcohol-related diagnosis in their preceding emergency admission. This was equally true of cases with cirrhosis or liver failure at index admission, suggesting serious alcohol-related harm may have gone unrecognised (**Table 1**).

**Stage of liver disease** – stratification by recorded-stage is shown in **Figure 1** and **Supplementary Table S1**. These data demonstrate the high proportion of advanced liver disease recorded at index hospitalisation (cirrhosis: 28%; hepatic failure: 13%). We found frequent use of K70.9 (alcoholic liver disease, unspecified) to encode first admissions, whereby disease stage was not specified in 39%. The presence of additional codes for ascites (31%) and varices (12%) in many such cases suggests a significant proportion had severe disease (**Supplementary Table S1**).

Older groups had lower proportions with alcoholic hepatitis and a higher proportion with cirrhosis, as would be expected (**Figure 1A**). The proportion with "severe liver disease" codes (cirrhosis or hepatic failure) was similar among men and women despite statistical differences overall (**Figure 1B**). Interestingly, the proportion of first admissions with severe disease showed limited variation across deprivation quintiles and was slightly lower among those from most deprived areas (**Figure 1C**).

**Time trends in case-mix –** we found small differences in sociodemographics between patients discharged in Period 2 compared with Period 1 (**Supplementary Table S2**). Mean age was one year older (54.1 versus 52.5 years, $p<0.001$) and there was a slight reduction in proportion from most deprived areas (30% vs. 33%, $p<0.001$). There was an increased contribution of cirrhosis (31% vs. 25%, $p<0.001$) and hepatic failure (15% vs. 11%, $p<0.05$) but a reduction in unspecified stage (34% vs. 46%, $p<0.001$) (**Fig. 1D**). There was also a slight increase in proportion with codes for varices (16% vs. 15%, $p=0.031$). Levels of non-liver comorbidity were also higher (13% vs. 10% with score ≥2, $p<0.001$). Collectively, these findings suggest patients at index admission were presenting with somewhat more advanced disease and greater burden of comorbidities.

Among patients with prior GP contacts (preceding year), we found no change in the proportion of consultations with alcohol-related READ codes (46.6% vs. 48.1%, $p=0.13$) and a small reduction in liver disease codes in the final period (13.7% vs. 16.0%, $p<0.001$). There was no change in recording of alcohol-specific codes in prior emergency admissions (75.6% vs. 76.7%, $p=0.3$).

The characteristics of patients discharged within each fiscal year are summarised in **Suppl. Table S3.**

## Care processes during first admission



For peer review only - http://bmjopen.bmj.com/site/about/guidelines.xhtml

Overall mean length of stay was 12 days (**Table 1**), with higher duration for hepatic failure (16 days, p<0.001). Ten percent received higher level care (21% of those coded as hepatic failure). Length of stay was one day shorter in the second half of the decade (11 days versus 12 days, p<0.001, **Supplementary Table S2**), and a significantly greater proportion received higher level care (11% versus 8.6%, p<0.001).

**Deaths following first admission**

Overall, 2,708 deaths were recorded giving a crude case fatality rate (CFR) of 15.4% (**Table 1**). Median time to in-hospital death was nine days (mean: 14). One-in-five deaths occurred before day three of admission. Thirty nine percent of cases with a recorded-stage of hepatic failure died in hospital. Within 365 days of admission the cumulative death toll rose to 5,938 patients (34%).

Of the total in-hospital deaths, 2,569 (95%) had descriptors listed in one or more fields that were consistent with an ARLD-specific cause. The commonest descriptor was "alcoholic liver disease, unspecified" (**Supplementary Appendix 2**). Of these deaths, 1,738 (66.8%) were in the primary group and 831 (32.2%) in the uplift group.

Among patients with GP consultations in preceding year (n=9,936), those dying in hospital were less likely to have had coding of alcohol misuse or harms (39.3% vs 48.8%, p<0.01) or liver disease (10.1% vs 15.5%, p<0.05) than survivors. Patients with fatal admission were less likely to have had an emergency hospitalization in the past year than survivors (35.9% vs 42.2%, p<0.01). However, among those previously hospitalized, alcohol-specific diagnoses were recorded less often in those subsequently dying than survivors (68.7% vs 77.2%, p<0.01). Only 36.1% of in-hospital deaths received higher level care.

**Factors associated with death**

Characteristics of patients who had died, or survived, at the two study end-points are shown in **Suppl. Table S4**. In bivariate analyses we found significant associations with increasing age, female sex, primary (as opposed to uplift) case definition, increased non-liver comorbidity, advanced recorded-stage of liver disease and coding for ascites or varices. Stratified survival analyses confirmed these bivariate associations (**Suppl. Fig. 1)**. Multivariable models confirmed independent associations between the selected case-mix factors and odds of death (**Table 2**). Interestingly, deprivation status (IMD quintile) was not independently associated with outcomes and not retained in models – suggesting severity of illness at presentation dominated prognosis. Given the high prevalence of unspecified stage we used a binary variable for recorded-stage (Liver failure or Not) for final risk-adjustment.

**Time trends in case fatality**

***Five-year periods*** – Comparison of unadjusted outcomes between Period 1 and Period 2 revealed lower crude in-hospital CFR (14.8% vs 16.2%, p=0.018). This did not



For peer review only - http://bmjopen.bmj.com/site/about/guidelines.xhtml

quite reach significance at 365 days (33.2% vs 35.4%, p=0.063). Unadjusted rates of in-hospital death were numerically lower for each recorded-stage but not all comparisons were significant (hepatitis: 8.9% vs 9.9%, p=0.4; cirrhosis: 14.4% vs 17.7%, p=0.002; hepatic failure: 37.4% vs 41.2%, p=0.07; not specified: 9.7% vs 12.5%, p<0.001). Similarly, reductions in crude CFRs at 365 days did not all reach significance (hepatitis: 22.8% vs 24.5%, p=0.4; cirrhosis: 38.9% vs 41.9%, p=0.035; hepatic failure: 54.5% vs 57.7%, p=0.14; not specified: 26.0% vs 30.3%, p<0.001). Survival probabilities during the year after index admission were consistently higher for those discharged in Period 2– period for the cohort as a whole and by recorded stage (**Fig. 2)**. After adjusting for case-mix factors in relevant models, the adjusted odds of both in-hospital and 365 day death were significantly lower for those discharged in Period 2 (**Table 2**).

***Year-to-year trends*** – Time series are shown in **Figure 3**. Overall, crude in-hospital CFR declined from 18.3% in 2008/9 to 16.1% in 2017/18 (p=0.03) (**Figure 3A**). Corresponding changes in stage-specific crude rates were 45.4% to 41.0% for hepatic failure (p=0.13), 21.1% to 13.0% for cirrhosis (p=0.0006) and 16.1% to 8.8% for alcoholic hepatitis (p=0.47). Case-mix-adjusted rates for in-hospital death showed significant reductions overall and in stage-specific analyses (**Figure 3B** and **Table 2**). In the final risk-adjusted model (**Table 2)**, each one-year increment across the decade was associated (on average) with a 6% reduction in odds of in-hospital death (aOR: 0.94 [95% CI: 0.93-0.96], p<0.001). Stage-specific reductions in odds of dying during first admission were 3% per year for hepatic failure (p=0.004), 6% for cirrhosis (p<0.001) and 7% for alcoholic hepatitis (p<0.01).

Corresponding trends for 365-day death are shown (**Figure 3C** and **Figure 3D)**. Overall crude rate reduced from 36.8% to 32.2% (p=0.02) and stage-specific crude rates showed downward trends – hepatic failure reducing from 59.2% to 53.9% (p=0.07), cirrhosis from 46.1% to 36.3% (p=0.02) and alcoholic hepatitis from 28.3% to 21.7% (p=0.15). After case-mix adjustment, each one-year increment was associated with a 4% reduction in odds of dying within a year of admission overall (aOR: 0.96 [95% CI: 0.95-0.97], p<0.001). Stage-specific reductions in adjusted odds of death were also 4% per year for hepatic failure (p=0.011), cirrhosis (p<0.001) and alcoholic hepatitis (p=0.027).

**Sensitivity analyses**

Key findings were unchanged in terms of the significance or magnitude of trends when risk adjustment models were fitted using variants of patient-level covariates (e.g. continuous variables for age or comorbidity scores, individual categories for each stage of liver disease) (**Supplementary Figure S2**).

**Regional variations in case fatality rate of first admissions**



For peer review only - http://bmjopen.bmj.com/site/about/guidelines.xhtml

**Supplementary Table S5** shows distribution of cases and case-mix across the ten regions of England. Crude CFR varied from 13.4% (London) to 20.1% (South East Coast). Funnel charts identified two regions as potential "outliers" (i.e. higher than the average) on the basis of unadjusted rates – the West Midlands and South East Coast (**Supplementary Figure 3**) – whereas London had a lower-than-average crude rate. When region was added to relevant models (**Supplementary Table S6**), these two parts of the country had a significantly increased odds of in-hospital death relative to London, with the West Midlands having an odds ratio of 1.27 (95% CI: 1.09 – 1.49) and South East Coast an odds ratio of 1.36 (95% CI: 1.12 – 1.66). There was no significant difference between the other regions. Overall national time trends were not altered by including region in the five-year and year-to-year models.

## Discussion

Our study represents the first detailed description of characteristics and outcomes of index emergency admissions with ARLD in the English population. The predominance of males and disproportionate contribution of people living in more deprived areas is consistent with known epidemiological patterns.(1) These admissions encompass diverse manifestations of liver disease and alcohol misuse, ranging from early-stage liver disease to rapidly fatal liver failure. However, at least half had advanced disease.

Our data suggest ARLD is frequently diagnosed for the first time during unplanned hospitalization. Only 14.7% of cases with GP contacts in the year before index emergency admission for ARLD had liver disease recorded during those encounters, suggesting underlying liver problems were not suspected or diagnosed at that time. A previous study examined the earliest instance of coding for cirrhosis (regardless of aetiology) within linked CPRD-HES records (1998-2009), reporting this occurred first within an emergency record in half the cases.(21) We examined recent GP consultations for codes relating to alcohol excess, finding them absent in half. These data imply missed opportunities to screen for, or detect, alcohol-related harms at a point when patients would be expected to have had well-established harmful drinking.

Similar issues were apparent in secondary care with alcohol-related codes absent from almost a quarter of unplanned hospitalisations in preceding year. The corresponding figure was 31.3% for patients who subsequently died in-hospital. It seems unlikely such patients lacked evidence of alcohol-specific conditions during those prior admissions. Across the decade we found no improvement in rates of recognition/recording of alcohol-related problems in primary or secondary care contacts during the year before index hospitalisation. Deficiencies in early detection ~~of~~ have been recognised internationally.(22)

We found improvements in short and medium term prognosis of index emergency admissions over time but the magnitude was small. In 2017/18, one in seven were still dying during their first hospitalisation, rising to one third within a year. Whilst survival

For peer review only

was better in the post-NCEPOD era, yearly time series suggested no step-change or accelerated reduction in case fatality during this sustained period of national initiatives.(2–5,7) Directly comparable statistics for other countries are lacking.

Only a third of in-hospital deaths (36%) had received higher level care. This is comparable to 39% in the NCEPOD review where 31.2% of those deemed eligible (by reviewers) for higher level care didn't receive it.(2) This prompted recommendations for improved decision-making and access. Universal prognostic pessimism about organ support in severe liver disease has been challenged.(23) We cannot judge suitability of cases for escalation in our study but we did find a small increase in the proportion receiving such care over a ten year period.

Exploratory analyses suggest two regions had higher crude case fatality rates overall and differences persisted after risk-adjustment. However, most regions were comparable on these measures. Caution is needed when interpreting local-level outcome metrics derived from associations in national data (constant-risk fallacy). Geographical variation in population-based mortality rates (as opposed to CFRs) for liver disease are well-recognised but further exploration of possible inequalities in outcome of first admissions *per se* is merited.(24)

We confirm limitations of simple case definitions for defining workload and outcome.(11,15,16,21) Recent studies of international burden of "decompensated cirrhosis" continue to rely on primary discharge diagnosis.(25) Our analysis of deaths shows that many of the sickest patients had a non-primary coding pattern. Our findings on "recorded-stage" question the merits of defining "severe" liver disease based solely on primary codes (cirrhosis or liver failure).(26) Unspecified stage accounted for 28% of admissions and one in four deaths. If administrative data are to be used to quantify burden, evaluate interventions and benchmark performance then case identification and risk-adjustment requires care.(16) The LAA-based approach,(11,15) combined with case-mix classification reported here, could support development of improved metrics as recommended by the *Lancet* commission and in a recent NCEPOD update.(13,27) Existing statistics require caveats for interpretation.(24)

Our study has several limitations. First, the available data allowed us to define "first" emergency admission based on an admission-free interval of ten years. We cannot rule out inclusion of patients admitted as an emegency over a decade earlier who then avoided any subsequent emergency readmission or death. Given the prognosis and typical trajectory of ARLD, we believe such cases are rare.

Second, HES data lacks true disease severity measures (e.g. clinical scores or laboratory parameters), limiting risk-adjustment. However, we applied both recorded-stage and condition-specific markers (ascites and varices) in addition to the usual generic

For peer review only

covariates. We omitted hepatic encephalopathy – an important complication of severe disease (14) – based on experience of LAA development.(11) We found relevant codes (G92X Toxic encephalopathy; G934 Encephalopathy unspecified) were seldom recorded. Codes for altered consciousness or confusion were common but could indicate alcohol intoxication or withdrawal. Only 441 cases (2.5%) in the present study had encephalopathy coded, of which just 164 (<1%) lacked additional coding for ascites or varices.

Over the decade first admissions appeared to have somewhat more severe disease and higher burden of comorbidities. The possibility of changing coding practices over time cannot be excluded in any long term study of administrative data. However, our time trends for CFR were significant both for crude data and in sensitivity analyses omitting relevant covariates which suggests prognostic improvement is genuine rather than artefactual.

Third, whilst CPRD practices serve a representative population of English residents,(17) our results are not generalisable to every locality. A key aim was to examine events in primary care during the year before index admission but linked data are not available for the entire country. Further work using complete national HES is needed to quantify national trends and explore regional and provider variation.

Finally, although LAA performance in detecting true cases of ARLD against hospital records has been reported previously,(11,15) we could not conduct formal validity testing as inpatient data was limited to HES. However, linkage to death certification suggested the majority of in-hospital deaths in the uplift subgroup were related to alcoholic liver disease. The LAA is based on coding patterns observed in English HES data. It is unknown whether such variations exist in other countries.

In conclusion, we found evidence of gradual improvement in prognosis for first emergency admissions in England over a ten year period. Nevertheless, at least one in seven patients were still dying during index hospitalization and a third within a year. Many patients had contacts with health services in the year prior to admission but without apparent recognition of alcohol-related harms, adding to concerns about missed opportunities for screening and intervention.(20) Geographical variations require further investigation. Our observations support much of the pessimism expressed in the *Lancet Commission*'s final report(10) This highlights the need for concerted action in public health interventions and primary and secondary care to improve prevention, early detection and inpatient treatment of alcohol-related harms.

## Funding Statement

This work was funded by the UK Department of Health (Connected Health Cities programme) and delivered by the Northern Health Science Alliance. The funding sponsors had no role in the design and conduct of the study; in the collection,



For peer review only - http://bmjopen.bmj.com/site/about/guidelines.xhtml

management, analysis and interpretation of the data; or in the preparation, review or approval of the manuscript.

## Data Availability Statement

This study is based in part on data from the Clinical Practice Research Datalink obtained under licence from the UK Medicines and Healthcare products Regulatory Agency. The data is provided by patients and collected by the NHS as part of their care and support. The interpretation and conclusions contained in this study are those of the author/s alone. Copyright ©2023, re-used with the permission of The Health & Social Care Information Centre. All rights reserved. CPRD data are not publicy or freely available but can be obtained under special licence (https://cprd.com/data-access)

Code lists for the LAA are available at:
https://onlinelibrary.wiley.com/action/downloadSupplement?doi=10.1111%2Fapt.15781&file=apt15781-sup-0001-Supinfo.docx

## Authorship

Guarantor of the article: K.B.
Author contributions: K.B. wrote the manuscript and all authors reviewed and approved the final submission. K.B., K.F., B.S. and P.S. designed the research with input from all authors (T.M. and S.H.). K.B., B.S. and T.M. curated and analysed the data, with input from P.S. and K.F.

## Competing interests

No competing interest

## References

1. Williams R, Alexander G, Armstrong I, Baker A, Bhala N, Camps-Walsh G, et al. Disease burden and costs from excess alcohol consumption, obesity, and viral hepatitis: fourth report of the Lancet Standing Commission on Liver Disease in the UK. Lancet Lond Engl. 2018 Mar 17;391(10125):1097–107.

2. National confidential enquiry into patient outcome and death. Measuring the units: a review of patients who died with alcohol-related liver disease [Internet]. 2013 [cited 2019 Oct 1]. Available from: https://www.ncepod.org.uk/2013report1/downloads/MeasuringTheUnits_FullReport.pdf

3. Williams R, Horton R. Liver disease in the UK: a Lancet Commission. Lancet Lond Engl. 2013 Nov 9;382(9904):1537–8.

4. Williams R, Aspinall R, Bellis M, Camps-Walsh G, Cramp M, Dhawan A, et al. Addressing liver disease in the UK: a blueprint for attaining excellence in health care and reducing

premature mortality from lifestyle issues of excess consumption of alcohol, obesity, and viral hepatitis. Lancet Lond Engl. 2014 Nov 29;384(9958):1953–97.

5. Williams R, Ashton K, Aspinall R, Bellis MA, Bosanquet J, Cramp ME, et al. Implementation of the Lancet Standing Commission on Liver Disease in the UK. Lancet Lond Engl. 2015 Nov 21;386(10008):2098–111.

6. Dyson JK, Rajasekhar P, Wetten A, Ashraf HH, Ng S, Paremal S, et al. Implementation of a 'care bundle' improves the management of patients admitted to hospital with decompensated cirrhosis. Aliment Pharmacol Ther. 2016;44(10):1030–8.

7. McPherson S, Dyson J, Austin A, Hudson M. Response to the NCEPOD report: development of a care bundle for patients admitted with decompensated cirrhosis-the first 24 h. Frontline Gastroenterol. 2016 Jan;7(1):16–23.

8. Kiat C, O'Mahony S. Tackling the epidemic: the NCEPOD report on alcohol-related liver disease and the Lancet Commission on Liver Disease. J R Coll Physicians Edinb. 2018 Dec;48(4):291–2.

9. Kanwal F, Kramer JR, Buchanan P, Asch SM, Assioun Y, Bacon BR, et al. The quality of care provided to patients with cirrhosis and ascites in the Department of Veterans Affairs. Gastroenterology. 2012 Jul;143(1):70–7.

10. Williams R, Aithal G, Alexander GJ, Allison M, Armstrong I, Aspinall R, et al. Unacceptable failures: the final report of the Lancet Commission into liver disease in the UK. Lancet Lond Engl. 2020 18;395(10219):226–39.

11. Kallis C, Dixon P, Silberberg B, Affarah L, Shawihdi M, Grainger R, et al. Reducing variation in hospital mortality for alcohol-related liver disease in North West England. Aliment Pharmacol Ther. 2020 Jul;52(1):182–95.

12. NHS Digital [Internet]. [cited 2022 Sep 28]. 1.8 Emergency admissions for alcohol related liver disease. Available from: https://digital.nhs.uk/data-and-information/publications/statistical/ccg-outcomes-indicator-set/june-2020/domain-1-preventing-people-from-dying-prematurely-ccg/1-8-emergency-admissions-for-alcohol-related-liver-disease

13. Williams R, Alexander G, Aspinall R, Bosanquet J, Camps-Walsh G, Cramp M, et al. New metrics for the Lancet Standing Commission on Liver Disease in the UK. Lancet Lond Engl. 2017 May 20;389(10083):2053–80.

14. McPherson S, Lucey MR, Moriarty KJ. Decompensated alcohol related liver disease: acute management. BMJ. 2016 Jan 26;352:i124.

15. Dhanda A, Bodger K, Hood S, Henn C, Allison M, Amasiatu C, et al. The Liverpool alcohol-related liver disease algorithm identifies twice as many emergency admissions compared to standard methods when applied to Hospital Episode Statistics for England. Aliment Pharmacol Ther. 2022;(In Press).



For peer review only - http://bmjopen.bmj.com/site/about/guidelines.xhtml

16. King J, Walker K, Van Der Meulen J, Bernal W. Editorial: the burden of alcohol-related liver disease emergency admissions in England may be twice as high as previously thought. Aliment Pharmacol Ther. 2022 In Press;

17. Wolf A, Dedman D, Campbell J, Booth H, Lunn D, Chapman J, et al. Data resource profile: Clinical Practice Research Datalink (CPRD) Aurum. Int J Epidemiol. 2019 Dec 1;48(6):1740–1740g.

18. Shawihdi M, Dodd S, Kallis C, Dixon P, Grainger R, Bloom S, et al. Nationwide improvement in outcomes of emergency admission for ulcerative colitis in England, 2005-2013. Aliment Pharmacol Ther. 2019 Jul;50(2):176–92.

19. Charlson ME, Pompei P, Ales KL, MacKenzie CR. A new method of classifying prognostic comorbidity in longitudinal studies: development and validation. J Chronic Dis. 1987;40(5):373–83.

20. Burton R, Sheron N. Missed opportunities for intervention in alcohol-related liver disease in the UK. Lancet Gastroenterol Hepatol. 2017;2(7):469–71.

21. Ratib S, West J, Fleming KM. Liver cirrhosis in England-an observational study: are we measuring its burden occurrence correctly? BMJ Open. 2017 Jul 13;7(7):e013752.

22. Shah ND, Ventura-Cots M, Abraldes JG, Alboraie M, Alfadhli A, Argemi J, et al. Alcohol-Related Liver Disease Is Rarely Detected at Early Stages Compared With Liver Diseases of Other Etiologies Worldwide. Clin Gastroenterol Hepatol Off Clin Pract J Am Gastroenterol Assoc. 2019 Oct;17(11):2320-2329.e12.

23. McPhail MJW, Parrott F, Wendon JA, Harrison DA, Rowan KA, Bernal W. Incidence and Outcomes for Patients With Cirrhosis Admitted to the United Kingdom Critical Care Units. Crit Care Med. 2018 May;46(5):705–12.

24. Liver Atlas of Variation [Internet]. [cited 2023 Sep 23]. Available from: http://tools.england.nhs.uk/images/LiverAtlas17/atlas.html

25. GBD 2017 Cirrhosis Collaborators. The global, regional, and national burden of cirrhosis by cause in 195 countries and territories, 1990-2017: a systematic analysis for the Global Burden of Disease Study 2017. Lancet Gastroenterol Hepatol. 2020 Mar;5(3):245–66.

26. Roberts SE, John A, Brown J, Napier DJ, Lyons RA, Williams JG. Early and late mortality following unscheduled admissions for severe liver disease across England and Wales. Aliment Pharmacol Ther. 2019;49(10):1334–45.

27. National Confidential Enquiry into Patient Outcome and Death. Remeasuring the Units: An update on the organisation of alcohol-related liver disease services [Internet]. 2022. Available from: https://www.ncepod.org.uk/pdf/current/Remeasuring%20the%20Units_full%20report.pdf

For peer review only

**Table 1. Demographics, clinical characteristics, care processes and outcomes of patients admitted as an emergency for the first time with alcohol-related liver disease between 2008/9 and 2017/18**. Patients registered with an English CPRD practice. Data are shown for the cohort overall ('Total') and by case definition based on the Liverpool ARLD Algorithm ('Primary' or 'Uplift').

| **Characteristic** | **Total** | **Case definition** | | **p** |
|---|---|---|---|---|
| | (n=17,575) | **Primary** (n=9,374) | **Uplift** (n=8,201) | |
| Age, *mean (sd)* | 53.4 (12) | 53.2 (12) | 53.6 (13) | * |
| Sex | | | | |
| *Female* | 5,820 (33.1%) | 3,335 (35.6%) | 2,485 (30.3%) | ** |
| *Male* | 11,755 (66.9%) | 6,039 (64.4%) | 5,716 (69.7%) | |
| Deprivation quintile | | | | |
| *1 - Least deprived* | 2,435 (13.9%) | 1,330 (14.2%) | 1,105 (13.5%) | *ns* |
| *2* | 2,735 (15.6%) | 1,481 (15.8%) | 1,254 (15.3%) | |
| *3* | 3,028 (17.2%) | 1,613 (17.2%) | 1,415 (17.3%) | |
| *4* | 3,837 (21.8%) | 2,077 (22.2%) | 1,760 (21.5%) | |
| *5 - Most deprived* | 5,540 (31.5%) | 2,873 (30.6%) | 2,667 (32.5%) | |
| Stage of liver disease | | | | |
| *Fatty liver* | 942 (5.4%) | 182 (1.9%) | 760 (9.3%) | ** |
| *Hepatitis* | 2,369 (13.5%) | 1,729 (18.4%) | 640 (7.8%) | |
| *Fibrosis and sclerosis* | 62 (0.4%) | 28 (0.3%) | 34 (0.4%) | |
| *Cirrhosis* | 5,001 (28.5%) | 2,759 (29.4%) | 2,242 (27.3%) | |
| *Hepatic failure* | 2,354 (13.4%) | 1,982 (21.1%) | 372 (4.5%) | |
| *Not specified* | 6,847 (39.0%) | 2,694 (28.7%) | 4,153 (50.6%) | |
| Ascites | 6,589 (37.5%) | 4,648 (49.6%) | 1,941 (23.7%) | ** |
| Varices | 2,704 (15.4%) | 1,508 (16.1%) | 1,196 (14.6%) | * |
| Comorbidity score (non-liver) | | | | |
| *0-1* | 15,506 (88.2%) | 8,440 (90.0%) | 7,066 (86.2%) | * |
| *2+* | 2,069 (11.8%) | 934 (10.0%) | 1,135 (13.8%) | |
| Five year period of discharge | | | | |
| *2008/9 - 2012/13* | 7,566 (43.0%) | 4,102 (43.8%) | 3,464 (42.2%) | * |
| *2013/14 - 2017/18* | 10,009 (57.0%) | 5,272 (56.2%) | 4,737 (57.8%) | |
| GP contact in last year | 9,936 (56.5%) | 5,346 (57.0%) | 4,590 (56.0%) | *ns* |
| *With alcohol codes recorded §* | 4702 (47.3%) | 2528 (47.3%) | 2174 (47.4%) | *ns* |
| *With liver codes recorded §* | 1465 (14.7%) | 865 (16.2%) | 600 (13.1%) | ** |
| Emergency admission in last year | 7,265 (41.3%) | 3,240 (34.6%) | 4,025 (49.1%) | * |
| *With alcohol codes recorded ¶* | 5,525 (76.0%) | 2,393 (73.9%) | 3,132 (77.8.%) | ** |
| Length of stay, mean (sd) | 12 (15) | 14 (16) | 9 (13) | ** |
| Higher level care during admission | 1,750 (10.0%) | 970 (10.3%) | 780 (9.5%) | *ns* |
| Died in-hospital | 2,708 (15.4%) | 1,779 (19.0%) | 929 (11.3%) | ** |
| Died within 365 days of admission | 5,938 (33.8%) | 3,642 (38.9%) | 2,296 (28.0%) | ** |

* p<0.05; **p<0.001 between primary and uplift groups; ns = not statistically significant; na = not applicable; § = percentages based on those with a GP contact within the last year; ¶ = percentages based on those with an emergency admission within the last year.


For peer review only - http://bmjopen.bmj.com/site/about/guidelines.xhtml

**Table 2. Factors associated with death following first emergency admission for alcohol-related liver disease among people registered with CPRD practices in England, 2008/9 to 2017/18.** The final case-mix adjustment models included seven patient level baseline covariates. Additional time-related variables were then added to investigate time trends, using either fiscal year of discharge (continuous) or 5-year time period. Figure 3 illustrates the time trends in crude and case-mix adjusted case fatality rates derived using these models.

| Covariate | Death during first admission | | | | Death within 365 days | | | |
|---|---|---|---|---|---|---|---|---|
| | OR | 95% CI | aOR | 95% CI | OR | 95% CI | aOR | 95% CI |
| **Age Group** *(Ref: <40 years)* | | | | | | | | |
| *40-49* | **1.42** | 1.18, 1.70 | **1.31** | 1.09, 1.58 | **1.33** | 1.18, 1.50 | **1.2** | 1.06, 1.37 |
| *50-59* | **2.36** | 1.99, 2.81 | **2.02** | 1.70, 2.43 | **2.01** | 1.79, 2.26 | **1.67** | 1.48, 1.88 |
| *60-69* | **3.39** | 2.85, 4.04 | **2.86** | 2.39, 3.43 | **2.97** | 2.63, 3.35 | **2.36** | 2.08, 2.68 |
| *70+* | **4.11** | 3.41, 4.98 | **3.33** | 2.73, 4.07 | **5.39** | 4.69, 6.20 | **4.14** | 3.57, 4.80 |
| **Sex** *(Ref: Female)* | | | | | | | | |
| *Male* | **0.86** | 0.79, 0.93 | **0.79** | 0.72, 0.87 | 1.00 | 0.94, 1.07 | **0.93** | 0.86, 0.99 |
| **Case Definition** *(Ref: Primary)* | | | | | | | | |
| *Uplift* | **0.55** | 0.50, 0.59 | **0.76** | 0.69, 0.84 | **0.61** | 0.57, 0.65 | **0.75** | 0.70, 0.81 |
| **Non-liver comorbidity** *(Ref: 0-1)* | | | | | | | | |
| *2+* | **2.38** | 2.14, 2.64 | **1.97** | 1.75, 2.21 | **2.92** | 2.66, 3.20 | **1.97** | 1.75, 2.21 |
| **Hepatic Failure** *(Ref: Any other stage)* | | | | | | | | |
| *Hepatic failure* | **4.73** | 4.29, 5.21 | **4.03** | 3.64, 4.48 | **2.87** | 2.62, 3.13 | **2.32** | 2.11, 2.55 |
| **Ascites** *(Ref: No)* | | | | | | | | |
| *Yes* | **2.03** | 1.87, 2.21 | **1.48** | 1.35, 1.62 | **2.31** | 2.17, 2.47 | **1.86** | 1.74, 2.00 |
| **Varices** *(Ref: No)* | | | | | | | | |
| *Yes* | **1.35** | 1.21, 1.50 | **1.2** | 1.07, 1.34 | **1.41** | 1.30, 1.53 | **1.21** | 1.11, 1.33 |
| | Model 1, C-statistic: 0.733 | | | | Model 2, C-statistic: 0.710 | | | |
| **Fiscal Year** *(per year)* | | | | | | | | |
| | **0.98** | 0.96, 0.99 | **0.94** | 0.93, 0.96 | **0.98** | 0.97, 0.99 | **0.96** | 0.95, 0.97 |
| | Fiscal year added to Model 1, C-statistic: 0.737 | | | | Fiscal year added to Model 2, C-statistic: 0.710 | | | |
| **Five Year Period** *(Ref: 2008/9 to 2012/13)* | | | | | | | | |
| *2013/14 to 2017/18* | **0.91** | 0.83, 0.98 | **0.76** | 0.69, 0.83 | **0.91** | 0.83, 0.98 | **0.83** | 0.77, 0.88 |
| | Five Year Period added to Model 1, C-statistic: 0.736 | | | | Five Year Period added to Model 2, C-statistic: 0.708 | | | |

OR=unadjusted odds ratio; aOR =risk-adjusted odds ratio; significant ORs are in bold text (p values <0.001 in all cases)

### Legends to Figures

**Figure 1. Distribution of stage of alcohol-related liver disease at first emergency admission identified from the CPRD population of England.** Stage is based on the ICD-10 codes recorded on discharge (n=17,575 cases). Stratified by: (A) Age group (B) Sex, (C) Deprivation Quintile (1=Least, 5=Most deprived areas) and (D) Five-year period of discharge. All p values <0.001 (Chi Squared).

.

**Figure 2. Kaplan-Meier survival probability following first emergency admission for alcohol-related liver disease.** (A) to (G) are stratified into five-year cohorts (2008/9 to 2012/13 versus 20013/14 to 2017/18): (A) Overall (n=17,575), (B) Primary case definition (n=9,374), (C) Uplift case definition (n=8,201), (D) Alcoholic hepatitis (n=2,369), (E) Cirrhosis (n=5,001), (F) Hepatic failure (n=2,354), (G) Unspecified stage of liver disease (n=6,847). (H) shows all cases stratified by recorded-stage of liver disease. p values represent significance level of log rank test.

**Figure 3. Time trends in case fatality rates (CFR) for first emergency admissions for alcohol-related liver disease, overall and by stage of liver disease. (A)** Crude CFR in-hospital, **(B)** Risk-adjusted CFR in-hospital, **(C)** Crude CFR within 365 days, **(D)** Risk-adjusted CFR within 365 days. Risk-adjustment for overall rates was by age, sex, case definition (primary or uplift), comorbidity score (non-liver), stage of liver disease (liver failure or not) and coding for ascites and varices. Binary logistic regression models confirmed significant downward linear trends over the ten years overall, before and after risk-adjustment (See Table 2). For stage-specific rates, risk-adjustment was by age, sex, case definition and comorbidity score, with additional relevant models confirming significance of trends.

For peer review only

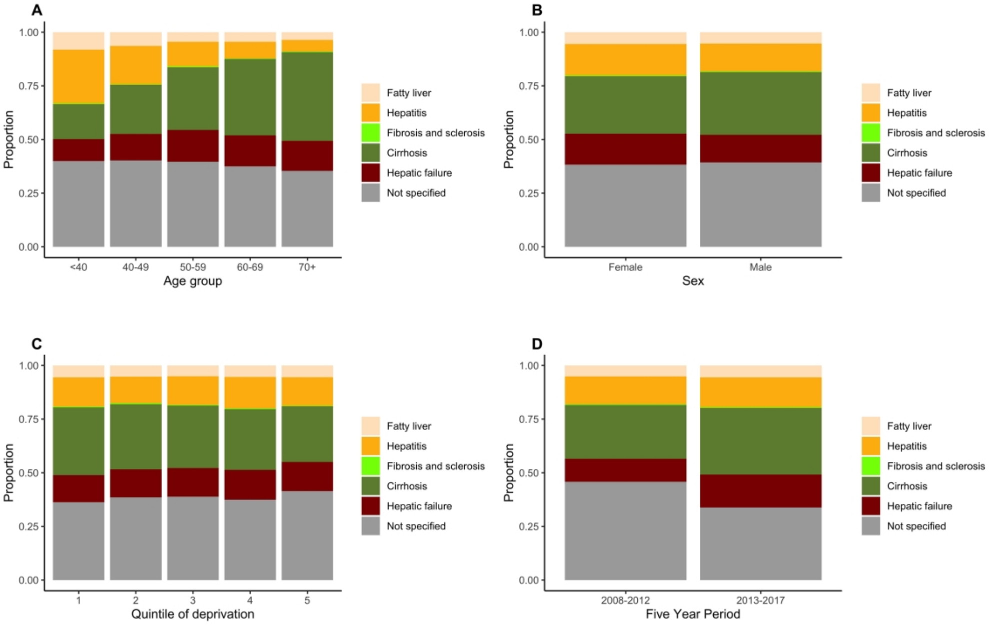


Figure 1

304x203mm (300 x 300 DPI)

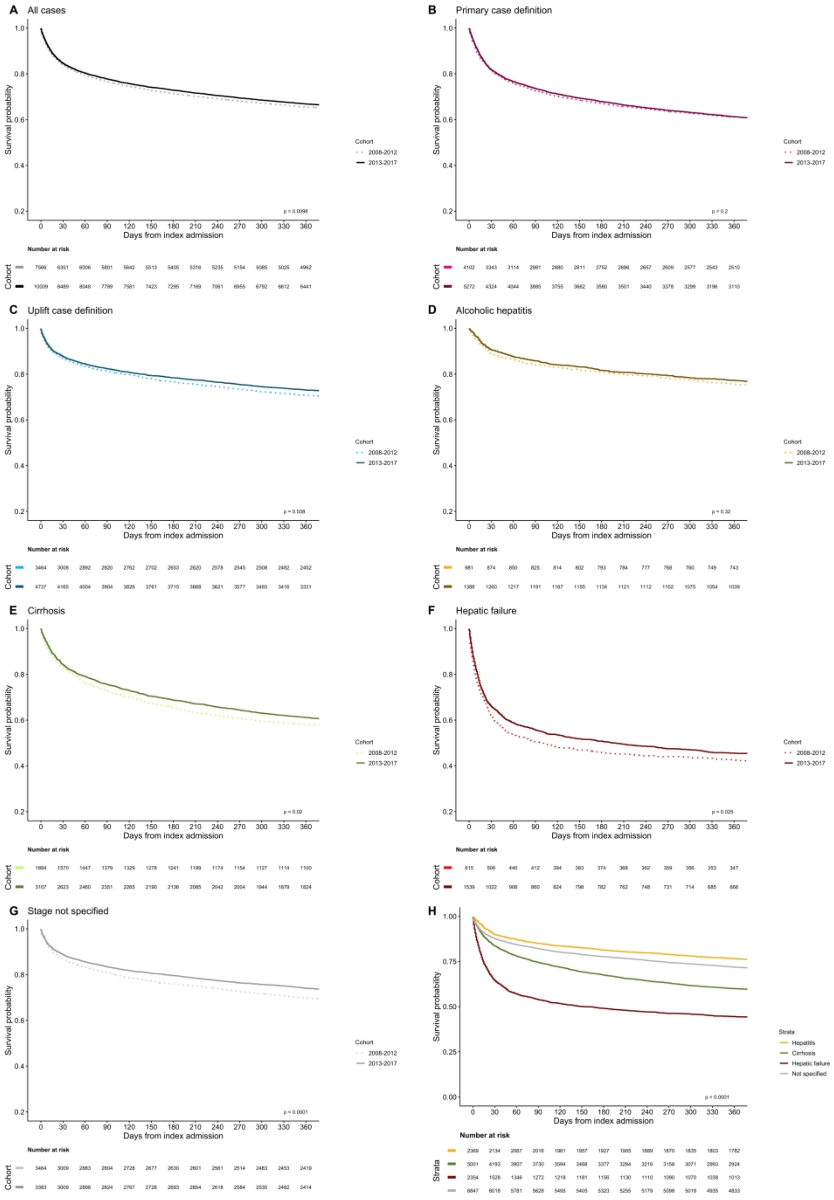


Figure 2

362x508mm (300 x 300 DPI)

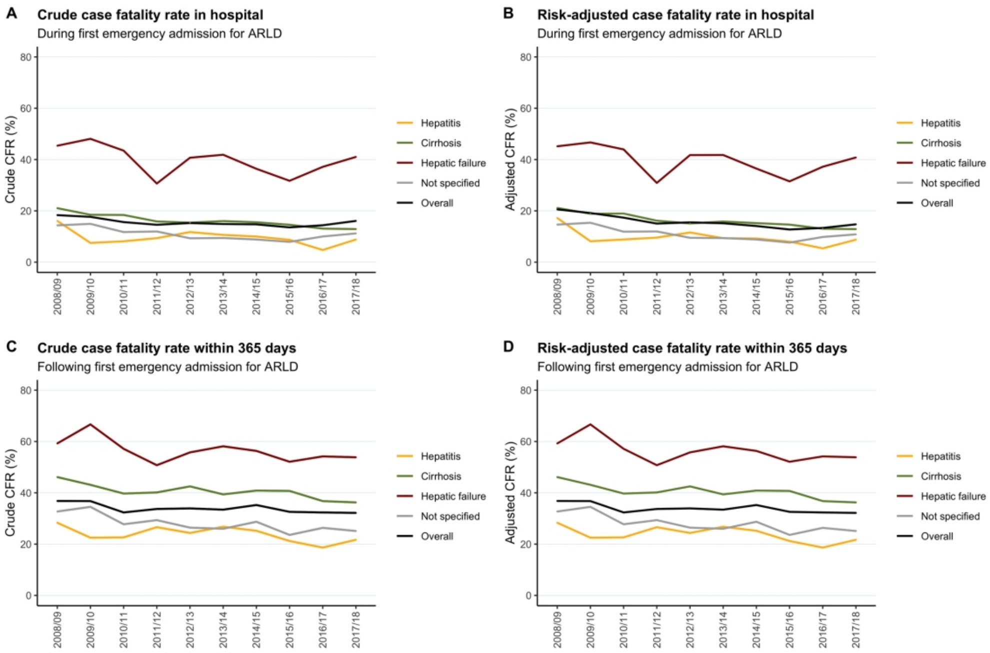


Figure 3

203x135mm (300 x 300 DPI)

# Supplementary Materials & Appendices: BMJ Open

## Outcomes of first emergency admissions for alcohol-related liver disease in England over a ten year period: Retrospective observational cohort study using linked electronic databases

**Supplementary Figure S1.** Kaplan-Meier survival probability of first emergency admissions with a diagnosis of alcohol-related liver disease, stratified by patient-level covariates

**Supplementary Table S1.** Demographics, clinical characteristics, care processes and outcomes of patients admitted as an emergency for the first time with alcohol-related liver disease between 2008/9 and 2017/18. Stratified by stage of liver disease.

**Supplementary Table S2.** Demographics, clinical characteristics, care processes and outcomes of patients admitted as an emergency for the first time with alcohol-related liver disease between 2008/9 and 2017/18. Stratified by 5-year period of discharge.

**Supplementary Table S3.** Demographics, clinical characteristics, care processes and outcomes of patients admitted as an emergency for the first time with alcohol-related liver disease between 2008/9 and 2017/18. Stratified by fiscal year of discharge.

**Supplementary Table S4.** Demographics and clinical characteristics of those who survived or died following first emergency admission with alcohol-related liver disease between 2008/09 and 2017/18.

**Supplementary Figure S2.** Sensitivity analyses of main findings exploring alternative covariates for risk-adjustment models

**Supplementary Table S5.** Demographics, clinical characteristics, care processes and outcomes of patients admitted as an emergency for the first time with alcohol-related liver disease between 2008/9 and 2017/18. Stratified by region of residence.

**Supplementary Figure S3**. Funnel plot showing regional variation in crude (unadjusted) case fatality rate following first emergency admission for alcohol-related liver disease among people registered with CPRD practices in England, 2008/9 to 2017/18.

**Supplementary Table S6.** Exploratory Analysis: Association between region of residence and death following first emergency admission for alcohol-related liver disease among people registered with CPRD practices in England, 2008/9 to 2017/18.

**Supplementary Appendix 1.** Medcodes and corresponding readterms used for primary care records to identify entries relating to alcohol misuse and/or alcohol-related liver disease (ARLD). Codes for ARLD are marked with an asterix (*).

**Supplementary Appendix 2.** used for screening all diagnoses listed on the death certificate in patients dying during index admission for ARLD.

**Supplementary Appendix 3.** Higher definition images for time serices (**Figure 3**).



For peer review only - http://bmjopen.bmj.com/site/about/guidelines.xhtml

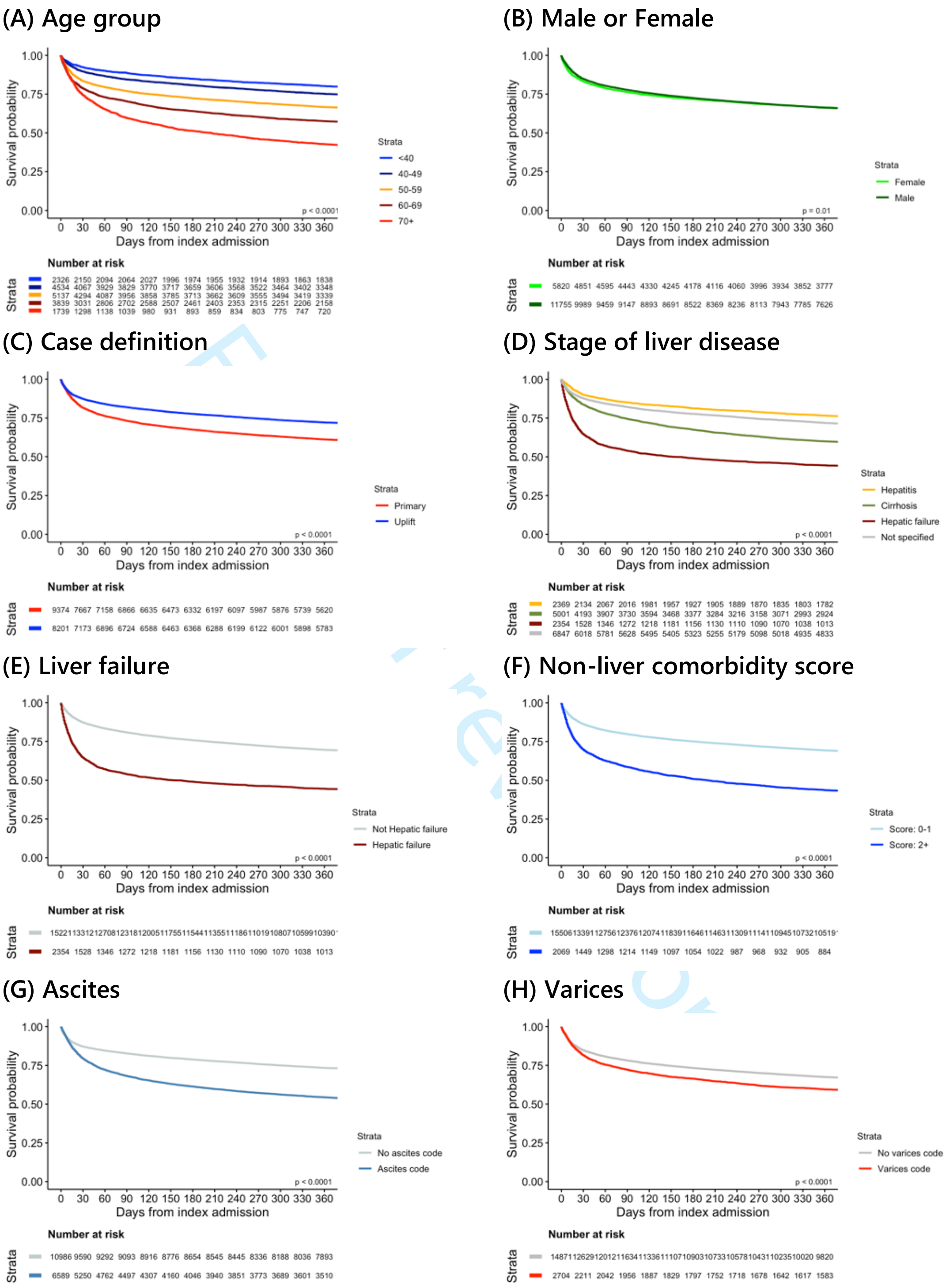


**Supplementary Figure S1. Kaplan-Meier survival probability of first emergency admissions with a diagnosis of alcohol-related liver disease, stratified by patient-level covariates.** (A) Age group, (B) Sex, (C) Case definition, (D) Stage of liver disease (selected stages only), (E) Liver failure or Not, (F) Non-liver comorbidity score, (G) Presence of codes for ascites, (H) Presence of codes for varices. All p values were significant between strata by log rank test.

**Supplementary Table S1.** Demographics, clinical characteristics, care processes and outcomes of patients admitted as an emergency for the first time with alcohol-related liver disease between 2008/9 and 2017/18. Patients registered with an English CPRD practice. Stratified by stage of liver disease. Excludes fatty liver (n=942) and fibrosis & sclerosis (n=62).

| Characteristic | Hepatitis (n=2,369) | Cirrhosis (n=5,001) | Hepatic failure (n=2,354) | Not specified (n=6,847) | p |
|---|---|---|---|---|---|
| Age, mean (sd) | 47.7 (12) | 56.8 (12) | 54.6 (12) | 53.0 (12) | ** |
| Sex | | | | | |
| *Female* | 848 (35.8%) | 1,562 (31.2%) | 839 (35.6%) | 2,227 (32.5%) | ** |
| *Male* | 1,521 (64.2%) | 3,439 (68.8%) | 1,515 (64.4%) | 4,620 (67.5%) | |
| Deprivation quintile | | | | | |
| *1 - Least deprived* | 332 (14.0%) | 769 (15.4%) | 307 (13.0%) | 883 (12.9%) | ** |
| *2* | 337 (14.2%) | 831 (16.6%) | 357 (15.2%) | 1,054 (15.4%) | |
| *3* | 404 (17.1%) | 882 (17.6%) | 404 (17.2%) | 1,176 (17.2%) | |
| *4* | 560 (23.6%) | 1,084 (21.7%) | 533 (22.6%) | 1,437 (21.0%) | |
| *5 - Most deprived* | 736 (31.1%) | 1,435 (28.7%) | 753 (32.0%) | 2,297 (33.5%) | |
| Case definition | | | | | |
| *Primary* | 1,729 (73.0%) | 2,759 (55.2%) | 1,982 (84.2%) | 2,694 (39.3%) | ** |
| *Uplift* | 640 (27.0%) | 2,242 (44.8%) | 372 (15.8%) | 4,153 (60.7%) | |
| Ascites | 531 (22.4%) | 2,472 (49.4%) | 1,383 (58.8%) | 2,109 (30.8%) | ** |
| Varices | 154 (6.5%) | 1,261 (25.2%) | 415 (17.6%) | 845 (12.3%) | ** |
| Comorbidity score (non-liver) | | | | | |
| *0-1* | 2,255 (95.2%) | 4,150 (83.0%) | 2,040 (86.7%) | 6,132 (89.6%) | ** |
| *2+* | 114 (4.8%) | 851 (17.0%) | 314 (13.3%) | 715 (10.4%) | |
| Five-year period of discharge | | | | | |
| *2008/9 – 2012/13* | 981 (41.4%) | 1,894 (37.9%) | 815 (34.6%) | 3,464 (50.6%) | ** |
| *2013/14 – 2017/18* | 1,388 (58.6%) | 3,107 (62.1%) | 1,539 (65.4%) | 3,383 (49.4%) | |
| GP contact in last year | 1,268 (53.5%) | 2,921 (58.4%) | 1,336 (56.8%) | 3,887 (56.8%) | ** |
| *With alcohol codes recorded §* | 661 (52.1%) | 1302 (44.6%) | 619 (46.3%) | 1849 (47.6%) | ** |
| *With liver codes recorded §* | 94 (7.4%) | 593 (20.3%) | 228 (17.1%) | 514 (13.2%) | *ns* |
| Emergency admission in last year | 918 (38.8%) | 2,011 (40.2%) | 832 (35.3%) | 3,029 (44.2%) | ** |
| *With alcohol codes recorded ¶* | 771 (84.0%) | 1,407 (70.0%) | 593 (71.3%) | 2,352 (77.7%) | ** |
| Length of stay, mean (sd) | 12 (15) | 12 (16) | 16 (18) | 10 (13) | ** |
| Higher level care during admission | 138 (5.8%) | 508 (10.2%) | 496 (21.1%) | 556 (8.1%) | ** |
| Died in-hospital | 221 (9.3%) | 781 (15.6%) | 912 (38.7%) | 761 (11.1%) | ** |
| Died within 365 days of admission | 557 (23.5%) | 2,003 (40.1%) | 1,309 (55.6%) | 1,931 (28.2%) | ** |

* p<0.05; **p<0.001 between groups; ns = not significant; na = not applicable; § = percentages based on those with a GP contact within the last year; ¶ = percentages based on those with an emergency admission within the last year.

**Supplementary Table S2.** Demographics, clinical characteristics, care processes and outcomes of patients admitted as an emergency for the first time with alcohol-related liver disease between 2008/9 and 2017/18. Patients registered with an English CPRD practice. Stratified by 5-year period of discharge.

| Characteristic | Total | 5 Year Period of Discharge | | p |
|---|---|---|---|---|
| | **08/09 to 17/18** (n=17,575) | **08/09 to 12/13** (n=7,566) | **13/14 to 17/18** (n=10,009) | |
| Age, *mean (sd)* | 53.4 (12) | 52.5 (12) | 54.1 (12) | ** |
| Sex | | | | |
| *Female* | 5,820 (33.1%) | 2,477 (33%) | 3,343 (33%) | *ns* |
| *Male* | 11,755 (66.9%) | 5,089 (67%) | 6,666 (67%) | |
| Deprivation quintile | | | | |
| *1 - Least deprived* | 2,435 (13.9%) | 977 (13%) | 1,458 (15%) | ** |
| *2* | 2,735 (15.6%) | 1,162 (15%) | 1,573 (16%) | |
| *3* | 3,028 (17.2%) | 1,223 (16%) | 1,805 (18%) | |
| *4* | 3,837 (21.8%) | 1,674 (22%) | 2,163 (22%) | |
| *5 - Most deprived* | 5,540 (31.5%) | 2,530 (33%) | 3,010 (30%) | |
| Case definition | | | | |
| Primary | 7,595 (51.1%) | 4,102 (54%) | 5,272 (53%) | * |
| Uplift | 7,272 (48.9%) | 3,464 (46%) | 4,737 (47%) | |
| Stage of liver disease | | | | |
| *Fatty liver* | 942 (5.4%) | 385 (5.1%) | 557 (5.6%) | ** |
| *Hepatitis* | 2,369 (13.5%) | 981 (13%) | 1,388 (14%) | |
| *Fibrosis and sclerosis* | 62 (0.4%) | 27 (0.4%) | 35 (0.3%) | |
| *Cirrhosis* | 5,001 (28.5%) | 1,894 (25%) | 3,107 (31%) | |
| *Hepatic failure* | 2,354 (13.4%) | 815 (11%) | 1,539 (15%) | |
| *Not specified* | 6,847 (39.0%) | 3,464 (46%) | 3,383 (34%) | |
| Ascites | 6,589 (37.5%) | 2,799 (37%) | 3,790 (38%) | *ns* |
| Varices | 2,704 (15.4%) | 1,113 (15%) | 1,591 (16%) | * |
| Comorbidity score (non-liver) | | | | |
| *0-1* | 15,506 (88.2%) | 6,773 (90%) | 8,733 (87%) | ** |
| *2+* | 2,069 (11.8%) | 793 (10%) | 1,276 (13%) | |
| GP contact in last year | 9,936 (56.5%) | 4,513 (59.6%) | 5,423 (54.2%) | ** |
| *With alcohol codes recorded §* | 4702 (47.3%) | 2,173 (48.1%) | 2,529 (46.6%) | *ns* |
| *With liver codes recorded §* | 1465 (14.7%) | 724 (16.0%) | 741 (13.7%) | ** |
| Emergency admission in last year | 7,265 (41.3%) | 3,127 (41%) | 4,138 (41%) | *ns* |
| *With alcohol codes recorded ¶* | 5,525 (76.0%) | 2,398 (76.7%) | 3,127 (75.6%) | *ns* |
| Length of stay, mean (sd) | 12 (15) | 12 (15) | 11 (14) | ** |
| Higher level care during admission | 1,750 (10.0%) | 652 (8.6%) | 1,098 (11%) | ** |
| Died in-hospital | 2,708 (15.4%) | 1,222 (16%) | 1,486 (15%) | * |
| Died within 365 days of admission | 5,938 (33.8%) | 2,614 (35%) | 3,324 (33%) | *ns* |

* p<0.05; **p<0.001 between time periods; ns = not significant; § = percentages based on those with a GP contact within the last year; ¶ = percentages based on those with an emergency admission within the last year.



For peer review only - http://bmjopen.bmj.com/site/about/guidelines.xhtml

**Supplementary Table S3.** Demographics, clinical characteristics, care processes and outcomes of patients admitted as an emergency for the first time with alcohol-related liver disease between 2008/9 and 2017/18. Patients registered with an English CPRD practice. Stratified by fiscal year of discharge.

| Characteristic | 2008/09 | 2009/10 | 2010/11 | 2011/12 | 2012/13 | 2013/14 | 2014/15 | 2015/16 | 2016/17 | 2017/18 |
|---|---|---|---|---|---|---|---|---|---|---|
| | N = 1,494 | N = 1,512 | N = 1,645 | N = 1,626 | N = 1,692 | N = 1,741 | N = 1,771 | N = 1,992 | N = 2,044 | N = 2,058 |
| **Age**, mean (sd) | 52 (12) | 52 (12) | 52 (12) | 53 (13) | 53 (12) | 54 (12) | 54 (12) | 54 (12) | 54 (12) | 55 (13) |
| **Age group** | | | | | | | | | | |
| *<40* | 220 (14.7%) | 232 (15.3%) | 257 (15.6%) | 244 (15.0%) | 236 (13.9%) | 209 (12.0%) | 225 (12.7%) | 228 (11.4%) | 236 (11.5%) | 239 (11.6%) |
| *40-49* | 429 (28.7%) | 419 (27.7%) | 439 (26.7%) | 431 (26.5%) | 451 (26.7%) | 440 (25.3%) | 444 (25.1%) | 486 (24.4%) | 518 (25.3%) | 477 (23.2%) |
| *50-59* | 423 (28.3%) | 403 (26.7%) | 491 (29.8%) | 433 (26.6%) | 490 (29.0%) | 542 (31.1%) | 536 (30.3%) | 580 (29.1%) | 629 (30.8%) | 610 (29.6%) |
| *60-69* | 293 (19.6%) | 337 (22.3%) | 326 (19.8%) | 352 (21.6%) | 361 (21.3%) | 390 (22.4%) | 382 (21.6%) | 486 (24.4%) | 451 (22.1%) | 461 (22.4%) |
| *70+* | 129 (8.6%) | 121 (8.0%) | 132 (8.0%) | 166 (10.2%) | 154 (9.1%) | 160 (9.2%) | 184 (10.4%) | 212 (10.6%) | 210 (10.3%) | 271 (13.2%) |
| **Sex** | | | | | | | | | | |
| *Female* | 505 (33.8%) | 488 (32.3%) | 527 (32.0%) | 526 (32.3%) | 559 (33.0%) | 533 (30.6%) | 607 (34.3%) | 669 (33.6%) | 697 (34.1%) | 709 (34.5%) |
| *Male* | 989 (66.2%) | 1,024 (67.7%) | 1,118 (68.0%) | 1,100 (67.7%) | 1,133 (67.0%) | 1,208 (69.4%) | 1,164 (65.7%) | 1,323 (66.4%) | 1,347 (65.9%) | 1,349 (65.5%) |
| **Deprivation quintile** | | | | | | | | | | |
| *1 - Least deprived* | 174 (11.6%) | 209 (13.8%) | 201 (12.2%) | 207 (12.7%) | 243 (14.4%) | 253 (14.5%) | 240 (13.6%) | 287 (14.4%) | 320 (15.7%) | 301 (14.6%) |
| *2* | 236 (15.8%) | 222 (14.7%) | 243 (14.8%) | 250 (15.4%) | 264 (15.6%) | 274 (15.7%) | 267 (15.1%) | 331 (16.6%) | 289 (14.1%) | 359 (17.4%) |
| *3* | 264 (17.7%) | 219 (14.5%) | 282 (17.1%) | 259 (15.9%) | 264 (15.6%) | 298 (17.1%) | 336 (19.0%) | 362 (18.2%) | 376 (18.4%) | 368 (17.9%) |
| *4* | 334 (22.4%) | 339 (22.4%) | 361 (21.9%) | 344 (21.2%) | 398 (23.5%) | 369 (21.2%) | 365 (20.6%) | 424 (21.3%) | 438 (21.4%) | 465 (22.6%) |
| *5 - Most deprived* | 486 (32.5%) | 523 (34.6%) | 558 (33.9%) | 566 (34.8%) | 523 (30.9%) | 547 (31.4%) | 563 (31.8%) | 588 (29.5%) | 621 (30.4%) | 565 (27.5%) |
| **Case definition** | | | | | | | | | | |
| *Primary* | 843 (56.4%) | 819 (54.2%) | 878 (53.4%) | 870 (53.5%) | 906 (53.5%) | 897 (51.5%) | 959 (54.2%) | 1,054 (52.9%) | 1,092 (53.4%) | 1,056 (51.3%) |
| *Uplift* | 651 (43.6%) | 693 (45.8%) | 767 (46.6%) | 756 (46.5%) | 786 (46.5%) | 844 (48.5%) | 812 (45.8%) | 938 (47.1%) | 952 (46.6%) | 1,002 (48.7%) |
| **Stage of liver disease** | | | | | | | | | | |
| *Fatty liver* | 59 (3.9%) | 82 (5.4%) | 94 (5.7%) | 75 (4.6%) | 95 (5.6%) | 103 (5.9%) | 98 (5.5%) | 113 (5.7%) | 116 (5.7%) | 107 (5.2%) |
| *Hepatitis* | 180 (12.0%) | 200 (13.2%) | 221 (13.4%) | 203 (12.5%) | 230 (13.6%) | 254 (14.6%) | 250 (14.1%) | 264 (13.3%) | 295 (14.4%) | 272 (13.2%) |
| *Fibrosis and sclerosis* | 7 (0.5%) | 3 (0.2%) | 6 (0.4%) | 6 (0.4%) | 6 (0.4%) | 5 (0.3%) | 10 (0.6%) | 5 (0.3%) | 6 (0.3%) | 8 (0.4%) |
| *Cirrhosis* | 323 (21.6%) | 341 (22.6%) | 413 (25.1%) | 441 (27.1%) | 480 (28.4%) | 518 (29.8%) | 553 (31.2%) | 631 (31.7%) | 650 (31.8%) | 651 (31.6%) |
| *Hepatic failure* | 130 (8.7%) | 156 (10.3%) | 161 (9.8%) | 199 (12.2%) | 226 (13.4%) | 203 (11.7%) | 261 (14.7%) | 309 (15.5%) | 358 (17.5%) | 351 (17.1%) |
| *Not specified* | 795 (53.2%) | 730 (48.3%) | 750 (45.6%) | 702 (43.2%) | 655 (38.7%) | 658 (37.8%) | 599 (33.8%) | 670 (33.6%) | 619 (30.3%) | 669 (32.5%) |
| **Ascites** | 219 (14.7%) | 205 (13.6%) | 234 (14.2%) | 263 (16.2%) | 250 (14.8%) | 294 (16.9%) | 329 (18.6%) | 305 (15.3%) | 307 (15.0%) | 298 (14.5%) |
| **Varices** | 14 (0.9%) | 16 (1.1%) | 20 (1.2%) | 27 (1.7%) | 26 (1.5%) | 36 (2.1%) | 26 (1.5%) | 31 (1.6%) | 33 (1.6%) | 29 (1.4%) |
| **Comorbidity Score (non-liver)** | | | | | | | | | | |
| *0-1* | 1,341 (89.8%) | 1,357 (89.7%) | 1,469 (89.3%) | 1,456 (89.5%) | 1,505 (88.9%) | 1,515 (87.0%) | 1,538 (86.8%) | 1,746 (87.7%) | 1,792 (87.7%) | 1,787 (86.8%) |
| *2+* | 153 (10.2%) | 155 (10.3%) | 176 (10.7%) | 170 (10.5%) | 187 (11.1%) | 226 (13.0%) | 233 (13.2%) | 246 (12.3%) | 252 (12.3%) | 271 (13.2%) |
| **GP contact in last year** | 940 (62.9%) | 922 (61.0%) | 967 (58.8%) | 966 (59.4%) | 950 (56.1%) | 983 (56.5%) | 959 (54.2%) | 1,113 (55.9%) | 1,078 (52.7%) | 1,058 (51.4%) |
| *With alcohol codes recorded §* | 433 (46.1%) | 439 (47.6%) | 486 (50.3%) | 460 (47.6%) | 465 (48.9%) | 505 (51.4%) | 449 (46.8%) | 527 (47.3%) | 480 (44.5%) | 458 (43.3%) |
| *With liver codes recorded §* | 164 (17.4%) | 161 (17.5%) | 164 (17.0%) | 138 (14.3%) | 137 (14.4%) | 163 (16.6%) | 129 (13.5%) | 155 (13.9%) | 143 (13.3%) | 111 (10.5%) |
| **Emergency admission in last year** | 583 (39.0%) | 665 (44.0%) | 689 (41.9%) | 662 (40.7%) | 702 (41.5%) | 718 (41.2%) | 740 (41.8%) | 821 (41.2%) | 837 (40.9%) | 848 (41.2%) |
| *With alcohol codes ¶* | 443 (76.0%) | 506 (76.1%) | 541 (78.5%) | 502 (75.8%) | 538 (76.6%) | 556 (77.4%) | 543 (73.4%) | 616 (75.0%) | 631 (75.4%) | 649 (76.5%) |
| **Length of stay**, mean (sd) | 13 (16) | 13 (17) | 12 (15) | 11 (15) | 12 (15) | 12 (15) | 12 (15) | 11 (14) | 11 (14) | 11 (14) |
| **Higher level care during admission** | 92 (6.2%) | 149 (9.9%) | 157 (9.5%) | 146 (9.0%) | 149 (8.8%) | 192 (11.0%) | 211 (11.9%) | 198 (9.9%) | 224 (11.0%) | 232 (11.3%) |
| **Died in-hospital** | 274 (18.3%) | 267 (17.7%) | 257 (15.6%) | 237 (14.6%) | 258 (15.2%) | 259 (14.9%) | 261 (14.7%) | 270 (13.6%) | 294 (14.4%) | 331 (16.1%) |
| **Died with 365 days of admission** | 550 (36.8%) | 556 (36.8%) | 532 (32.3%) | 548 (33.7%) | 574 (33.9%) | 582 (33.4%) | 624 (35.2%) | 649 (32.6%) | 661 (32.3%) | 662 (32.2%) |

§ = percentages based on those with a GP contact within the last year; ¶ = percentages based on those with an emergency admission within the last year.



For peer review only - http://bmjopen.bmj.com/site/about/guidelines.xhtml

**Supplementary Table S4. Demographics and clinical characteristics of those who survived or died following first emergency admission with alcohol-related liver disease between 2008/09 and 2017/18.** Data are shown for in-hospital deaths and cumulative deaths at 365 days from admission.

| Characteristic | In-hospital | | | Within 365 days | | |
|---|---|---|---|---|---|---|
| | Survived N=14,867 | Died N=2,708 | p | Survived N=11,637 | Died N=5,939 | p |
| Age, mean (sd) | 52.6 (12) | 58.1 (12) | ** | 51.4 (12) | 57.5 (12) | ** |
| Age Group | | | | | | |
| <40 | 2,153 (14.5%) | 173 (6.4%) | ** | 1,862 (16.0%) | 464 (7.8%) | ** |
| 40-49 | 4,071 (27.4%) | 463 (17.1%) | | 3,405 (29.3%) | 1,129 (19.0%) | |
| 50-59 | 4,318 (29.0%) | 819 (30.2%) | | 3,421 (29.4%) | 1,716 (28.9%) | |
| 60-69 | 3,018 (20.3%) | 821 (30.3%) | | 2,207 (19.0%) | 1,632 (27.5%) | |
| 70+ | 1,307 (8.8%) | 432 (16.0%) | | 742 (6.4%) | 997 (16.8%) | |
| Sex | | | | | | |
| Female | 4,844 (32.6%) | 976 (36.0%) | ** | 3,857 (33.1%) | 1,963 (33.1%) | ns |
| Male | 10,023 (67.4%) | 1,732 (64.0%) | | 7,780 (66.9%) | 3,975 (66.9%) | |
| Deprivation quintile | | | | | | |
| 1 - Least deprived | 2,040 (13.7%) | 395 (14.6%) | * | 1,544 (13.3%) | 891 (15.0%) | ** |
| 2 | 2,289 (15.4%) | 446 (16.5%) | | 1,767 (15.2%) | 968 (16.3%) | |
| 3 | 2,530 (17.0%) | 498 (18.4%) | | 1,954 (16.8%) | 1,074 (18.1%) | |
| 4 | 3,251 (21.9%) | 586 (21.6%) | | 2,582 (22.2%) | 1,255 (21.1%) | |
| 5 - Most deprived | 4,757 (32.0%) | 783 (28.9%) | | 3,790 (32.6%) | 1,750 (29.5%) | |
| Case definition | | | | | | |
| Primary | 7,595 (51.1%) | 1,779 (65.7%) | ** | 5,732 (49.3%) | 3,642 (61.3%) | ** |
| Uplift | 7,272 (48.9%) | 929 (34.3%) | | 5,905 (50.7%) | 2,296 (38.7%) | |
| Stage of liver disease | | | | | | |
| Fatty liver | 916 (6.2%) | 26 (1.0%) | ** | 818 (7.0%) | 124 (2.1%) | ** |
| Hepatitis | 2,148 (14.4%) | 221 (8.2%) | | 1,812 (15.6%) | 557 (9.4%) | |
| Fibrosis and sclerosis | 55 (0.4%) | 7 (0.3%) | | 48 (0.4%) | 14 (0.2%) | |
| Cirrhosis | 4,220 (28.4%) | 781 (28.8%) | | 2,998 (25.8%) | 2,003 (33.7%) | |
| Hepatic failure | 1,442 (9.7%) | 912 (33.7%) | | 1,045 (9.0%) | 1,309 (22.0%) | |
| Not specified | 6,086 (40.9%) | 761 (28.1%) | | 4,916 (42.2%) | 1,931 (32.5%) | |
| Ascites | 5,179 (34.8%) | 1,410 (52.1%) | ** | 3,580 (30.8%) | 3,009 (50.7%) | ** |
| Varices | 2,191 (14.7%) | 513 (18.9%) | * | 1,609 (13.8%) | 1,095 (18.4%) | ** |
| Comorbidity score (non-liver) | | | | | | |
| 0-1 | 13,368 (89.9%) | 2,138 (79.0%) | ** | 10,736 (92.3%) | 4,770 (80.3%) | ** |
| 2+ | 1,499 (10.1%) | 570 (21.0%) | | 901 (7.7%) | 1,168 (19.7%) | |
| Length of stay, mean (sd) | 11 (15) | 14 (16) | ** | 11 (14) | 13 (16) | ** |
| Higher level care | 773 (5.2%) | 977 (36.1%) | ** | 601 (5.2%) | 1,149 (19.3%) | ** |

* p<0.05; **p<0.001 between deaths and survivors; ns= not statistically significant.

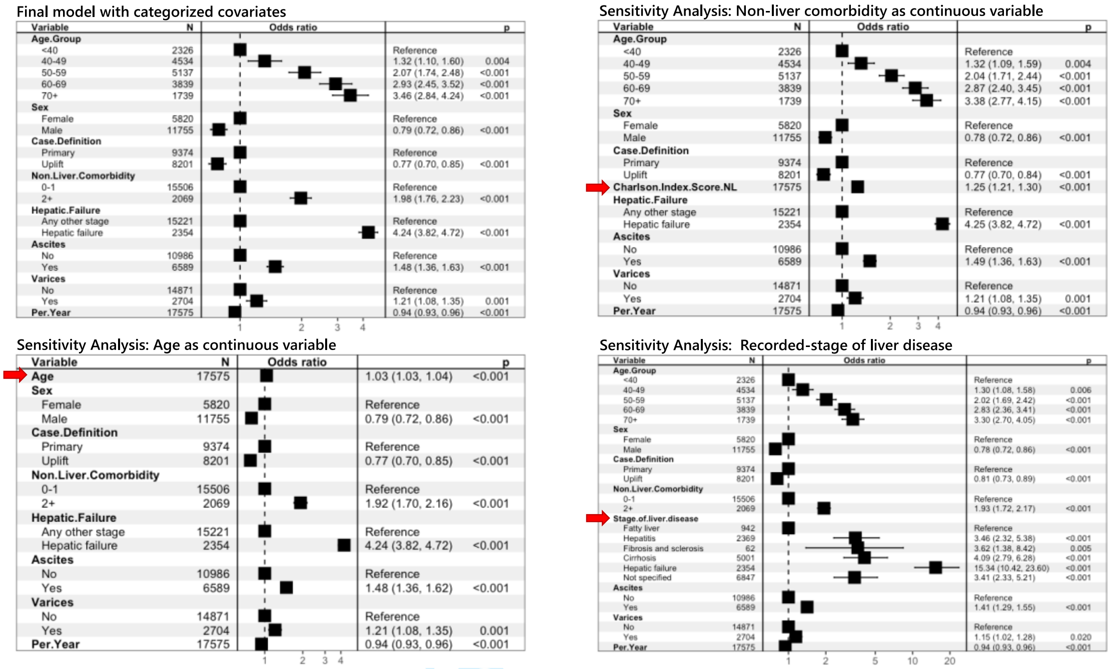


**Supplementary Figure S2. Sensitivity analyses using alternative covariates for risk-adjustment models.** Four alternative models are shown examining factors associated with in-hospital death. The final parsimonious model (upper left) included binary or categorized variables and demonstrates a year-on-year improvement in prognosis (OR 0.94 "per year" through the 10-year observation period). Substituting continuous variables for age (bottom left) or comorbidity score (top right) did not alter the findings (OR 0.94). Similarly, replacing the dichotomous "Hepatic Failure" variable with the individual categories of recorded-stage (bottom right) results in the same conclusions (OR 0.94).

For peer review only


For peer review only - http://bmjopen.bmj.com/site/about/guidelines.xhtml

**Supplementary Table S5.** Demographics, clinical characteristics, care processes and outcomes of patients admitted as an emergency for the first time with alcohol-related liver disease between 2008/9 and 2017/18. Stratified by region of residence.

| Characteristic | London N = 2,395 | North West N = 4,058 | Yorkshire & The Humber N = 769 | North East N = 1,113 | West Midlands N = 3,174 | East Midlands N = 416 | East of England N = 642 | South West N = 2,085 | South Central N = 1,648 | South East Coast N = 1,227 |
|---|---|---|---|---|---|---|---|---|---|---|
| Age, mean (SD) | 53.1 (12.0) | 52.5 (12.0) | 51.5 (12.9) | 52.2 (11.9) | 53.7 (12.2) | 52.8 (12.9) | 55.5 (12.5) | 54.1 (12.4) | 54.6 (12.4) | 55.3 (12.9) |
| Female | 717 (30%) | 1,377 (34%) | 259 (34%) | 403 (36%) | 1,052 (33%) | 145 (35%) | 207 (32%) | 694 (33%) | 544 (33%) | 413 (34%) |
| Case definition | | | | | | | | | | |
| Primary | 1,219 (51%) | 2,156 (53%) | 406 (53%) | 601 (54%) | 1,760 (55%) | 211 (51%) | 350 (55%) | 1,121 (54%) | 850 (52%) | 680 (55%) |
| Uplift | 1,176 (49%) | 1,902 (47%) | 363 (47%) | 512 (46%) | 1,414 (45%) | 205 (49%) | 292 (45%) | 964 (46%) | 798 (48%) | 547 (45%) |
| Non-liver comorbidity | | | | | | | | | | |
| 0-1 | 2,115 (88%) | 3,634 (90%) | 678 (88%) | 976 (88%) | 2,818 (89%) | 372 (89%) | 570 (89%) | 1,812 (87%) | 1,441 (87%) | 1,050 (86%) |
| 2+ | 280 (12%) | 424 (10%) | 91 (12%) | 137 (12%) | 356 (11%) | 44 (11%) | 72 (11%) | 273 (13%) | 207 (13%) | 177 (14%) |
| Hepatic failure | 285 (12%) | 675 (17%) | 92 (12%) | 127 (11%) | 438 (14%) | 74 (18%) | 78 (12%) | 217 (10%) | 154 (9.3%) | 205 (17%) |
| Ascites | 836 (35%) | 1,381 (34%) | 291 (38%) | 402 (36%) | 1,311 (41%) | 143 (34%) | 253 (39%) | 804 (39%) | 647 (39%) | 505 (41%) |
| Varices | 379 (16%) | 561 (14%) | 128 (17%) | 134 (12%) | 449 (14%) | 66 (16%) | 117 (18%) | 353 (17%) | 307 (19%) | 205 (17%) |
| Died in hospital | 321 (13.4%) | 605 (14.9%) | 120 (15.6%) | 150 (13.5%) | 549 (17.3%) | 67 (16.1%) | 102 (15.9%) | 295 (14.2%) | 246 (14.9%) | 247 (20.1%) |



For peer review only - http://bmjopen.bmj.com/site/about/guidelines.xhtml

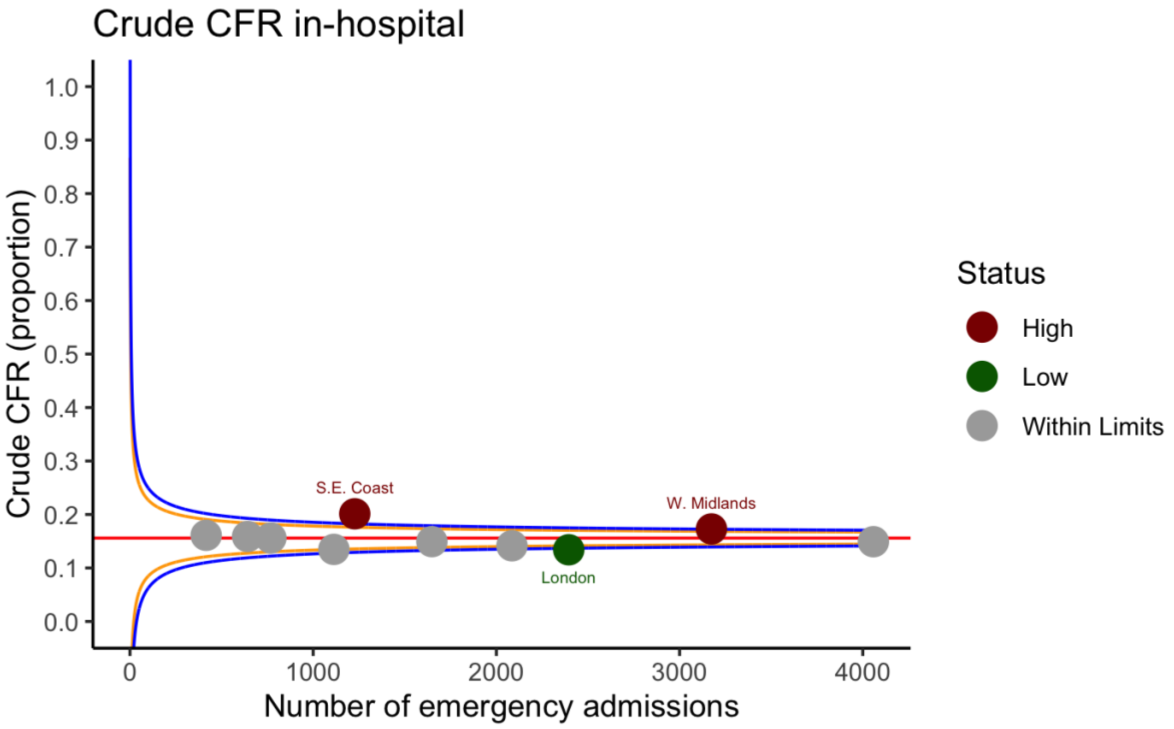


**Supplementary Figure S3 (Exploratory Analysis)**: **Funnel chart showing crude case fatality rate (CFR) in-hospital by region of residence.** Regions marked in red are statistical "outliers" with a higher-than-average crude rate, those in grey are "within limits" and those in green are lower-than-average. The red horizontal line shows average performance across the ten regions. Inner yellow line shows 95% control limits, outer blue line shows 99% control limits based on Spiegelhalter's methodology. Data relate to patients admitted as an emergency for the first time with alcohol-related liver disease (2008/9 and 2017/18), registered with an English CPRD practice. Data tabulated below.



For peer review only - http://bmjopen.bmj.com/site/about/guidelines.xhtml

**Supplementary Table S6 (Exploratory Analysis)**: Association between region of residence and death following first emergency admission for alcohol-related liver disease among people registered with CPRD practices in England, 2008/9 to 2017/18.

| Characteristic | Crude CFR | OR | 95% CI | p | aOR | 95% CI | p |
|---|---|---|---|---|---|---|---|
| London (Ref) | 13.4% | - | - | - | - | - | - |
| North West | 14.9% | 1.13 | 0.98, 1.31 | 0.10 | 1.06 | 0.91, 1.24 | 0.4 |
| Yorkshire & The Humber | 15.6% | 1.19 | 0.95, 1.50 | 0.13 | 1.22 | 0.95, 1.54 | 0.11 |
| North East | 13.5% | 1.01 | 0.82, 1.24 | >0.9 | 1.02 | 0.81, 1.26 | 0.9 |
| **West Midlands** | **17.3%** | **1.35** | **1.16, 1.57** | **<0.001** | **1.27** | **1.09, 1.49** | **0.003** |
| East Midlands | 16.1% | 1.24 | 0.93, 1.64 | 0.14 | 1.13 | 0.83, 1.53 | 0.4 |
| East of England | 15.9% | 1.22 | 0.95, 1.55 | 0.11 | 1.11 | 0.86, 1.43 | 0.4 |
| South West | 14.1% | 1.06 | 0.90, 1.26 | 0.5 | 1.02 | 0.85, 1.22 | 0.8 |
| South Central | 14.9% | 1.13 | 0.95, 1.36 | 0.2 | 1.10 | 0.91, 1.33 | 0.3 |
| **South East Coast** | **20.1%** | **1.63** | **1.36, 1.95** | **<0.001** | **1.36** | **1.12, 1.66** | **0.002** |

OR=unadjusted odds ratio; aOR =risk-adjusted odds ratio; significant values in bold. Unadjusted: C-Statistic 0.536. Adjusted: C-Statistic 0.733. Adjusted for age group, sex, case definition, non-liver comorbidity, hepatic failure, ascites and varices.

For peer review only

**Supplementary Appendix 1.** Medcodes and corresponding readterms used for primary care records to identify entries relating to alcohol misuse and/or alcohol-related liver disease (ARLD). Codes for ARLD are marked with an asterix (*).

| medcode | readterm |
|---|---|
| 322 | Moderate drinker - 3-6u/day |
| 669 | Nondependent alcohol abuse, unspecified |
| 1399 | Alcohol problem drinking |
| 1618 | Heavy drinker - 7-9u/day |
| 2081 | Alcoholism |
| 2082 | Alcohol withdrawal syndrome |
| 2083 | Alcohol detoxification |
| 2084 | Alcohol dependence syndrome |
| 2925 | Alcoholic polyneuropathy |
| 3216 | Acute alcoholic hepatitis* |
| 3782 | Intoxication – alcohol |
| 3782 | Intoxication – alcohol |
| 4500 | Korsakov's alcoholic psychosis |
| 4506 | Alcoholic gastritis |
| 4743 | Alcoholic cirrhosis of liver* |
| 4915 | Alcoholic cardiomyopathy |
| 5611 | [X]Mental and behavioural disorders due to use of alcohol |
| 5740 | Acute alcoholic intoxication in alcoholism |
| 5758 | [X]Chronic alcoholism |
| 6169 | Alcohol dependence syndrome NOS |
| 6467 | [X]Alcoholic hallucinosis |
| 7123 | [V]Personal history of alcoholism |
| 7602 | Chronic alcoholic hepatitis* |
| 7692 | Patient advised about alcohol |
| 7746 | Nondependent alcohol abuse |
| 7885 | Alcoholic liver damage unspecified |
| 7943 | Alcoholic hepatitis* |
| 8030 | [V]Alcohol abuse counselling and surveillance |
| 8363 | Oesophageal varices in alcoholic cirrhosis of the liver* |
| 8388 | [V]Alcohol rehabilitation |
| 8430 | H/O: alcoholism |
| 8984 | Ethyl alcohol causing toxic effect |
| 8999 | Heavy drinker |
| 9169 | [D]Alcohol blood level excessive |
| 9489 | Under care of community alcohol team |
| 9508 | [X]Acute alcoholic drunkenness |
| 9849 | Referral to community alcohol team |
| 10161 | O/E - alcoholic breath |
| 10463 | [X]Intent self poison/exposure to alcohol |
| 10658 | HoNOS item 3 - alcohol/drug problem |



For peer review only - http://bmjopen.bmj.com/site/about/guidelines.xhtml

| | |
|---|---|
| 10691 | Alcoholic fatty liver* |
| 11106 | Korsakov's alcoholic psychosis with peripheral neuritis |
| 11140 | Advice on alcohol consumption |
| 11263 | Wood alcohol causing toxic effect |
| 11491 | Health ed. – alcohol |
| 11670 | [X]Korsakov's psychosis, alcohol induced |
| 11740 | Alcohol misuse - enhanced services administration |
| 12353 | [X]Mental & behav dis due to use alcohol: psychotic disorder |
| 12442 | Alcohol disorder monitoring |
| 12554 | Referral to community drug and alcohol team |
| 12974 | Nondependent alcohol abuse, episodic |
| 12976 | Suspect alcohol abuse – denied |
| 12977 | Very heavy drinker - >9u/day |
| 12982 | Alcohol intake above recommended sensible limits |
| 12984 | Very heavy drinker |
| 12985 | Moderate drinker |
| 13709 | Blood ethanol level |
| 14422 | Urine ethanol |
| 15503 | Accidental poisoning by other ethyl alcohol and its products |
| 16225 | Alcohol withdrawal delirium |
| 16237 | Alcoholic psychoses |
| 16587 | [V]Problems related to lifestyle alcohol use |
| 17149 | [V]Blood-alcohol and blood-drug test |
| 17259 | [X]Delirium tremens, alcohol induced |
| 17330 | Alcoholic hepatic failure* |
| 17607 | [X]Alcoholic psychosis NOS |
| 18156 | Alcoholics anonymous |
| 18252 | Accidental poisoning by alcohol, NEC |
| 18711 | Lifestyle advice regarding alcohol |
| 19217 | Alcohol causing toxic effect |
| 19401 | Binge drinker |
| 19489 | Alcohol consumption screen |
| 19494 | Hazardous alcohol use |
| 19754 | Serum ethanol level |
| 20514 | [X]Mental and behav dis due to use alcohol: withdrawal state |
| 20762 | Alcohol amnestic syndrome |
| 21624 | Episodic acute alcoholic intoxication in alcoholism |
| 21650 | Admitted to alcohol detoxification centre |
| 21713 | Alcoholic fibrosis and sclerosis of liver* |
| 21879 | [X]Mental and behav dis due to use of alcohol: harmful use |
| 22707 | Drinking problem scale |
| 23610 | Nondependent alcohol abuse, continuous |
| 23978 | [X]Evid of alcohol involv determind by level of intoxication |
| 24064 | Continuous chronic alcoholism |

For peer review only



For peer review only - http://bmjopen.bmj.com/site/about/guidelines.xhtml

| | |
|---|---|
| 24735 | O/E - breath - alcohol smell |
| 24984 | Alcohol-induced chronic pancreatitis |
| 25110 | Alcohol withdrawal hallucinosis |
| 26106 | Episodic chronic alcoholism |
| 26323 | [X]Alcoholic dementia NOS |
| 27342 | Alcoholic dementia NOS |
| 27518 | Hangover (alcohol) |
| 28150 | Nondependent alcohol abuse NOS |
| 28780 | [X]Alcohol addiction |
| 29691 | Aversion therapy - alcoholism |
| 30036 | [X]Poisoning/exposure, ? intent, to alcohol |
| 30162 | [X]Alcoholic paranoia |
| 30404 | Alcoholic paranoia |
| 30460 | Alcoholism counselling |
| 30604 | Alcohol-induced epilepsy |
| 30695 | Harmful alcohol use |
| 31443 | Chronic alcoholism |
| 31605 | [X]Accident poisoning/exposure to alcohol |
| 31742 | Alcoholic myopathy |
| 32850 | Alcohol use disorders identification test |
| 32927 | [X]Alcohol withdrawal-induced seizure |
| 32964 | Alcohol abuse monitoring |
| 33635 | Chronic alcoholism NOS |
| 33670 | Other alcoholic psychosis |
| 33839 | Cerebellar ataxia due to alcoholism |
| 35330 | Alcohol consumption counselling |
| 35859 | Pregnancy alcohol advice |
| 36296 | Acute alcoholic intoxication in alcoholism NOS |
| 36499 | Alcohol causing toxic effect NOS |
| 36714 | Ethanol causing toxic effect |
| 36748 | Alcoholic encephalopathy |
| 37264 | Alcohol leaflet given |
| 37691 | [X]Chronic alcoholic brain syndrome |
| 37946 | Chronic alcoholic brain syndrome |
| 38012 | Accidental poisoning by ethanol, NOS |
| 38061 | Alcohol induced hallucinations |
| 39327 | [X]Mental and behav dis due to use alcohol: dependence syndr |
| 39799 | [X]Mental and behav dis due to use alcohol: amnesic syndrome |
| 40530 | Acute alcoholic intoxication, unspecified, in alcoholism |
| 40541 | Accidental poisoning by alcoholic beverages |
| 40602 | ADS - Alcohol dependence scale |
| 41251 | BMAST - Brief Michigan alcoholism screening test |
| 41638 | [X]Int self poison/exposure to alcohol at home |
| 41920 | Alcohol amnestic syndrome NOS |



For peer review only - http://bmjopen.bmj.com/site/about/guidelines.xhtml

| | |
|---|---|
| 41983 | Alcohol detoxification |
| 42305 | Severity of alcohol dependence questionnaire |
| 43193 | Unspecified chronic alcoholism |
| 43813 | Police:venesect-alcohol |
| 44299 | [X]Mental & behav dis due to use alcohol: acute intoxication |
| 44783 | Pain in lymph nodes after alcohol consumption |
| 45169 | [X]Men & behav dis due to use alcohol: oth men & behav dis |
| 46677 | Alcohol withdrawal regime |
| 46684 | Plasma ethanol level |
| 47123 | Alcohol counselling by other agencies |
| 47555 | Cerebral degeneration due to alcoholism |
| 48514 | Denatured alcohol causing toxic effect |
| 48545 | Alcohol questionnaire completed |
| 50507 | Alcohol dependence scale |
| 53139 | Accidental poisoning by other alcohols |
| 54209 | Advice to change alcohol intake |
| 54505 | Other alcoholic dementia |
| 55415 | Accidental poisoning by alcohol NOS |
| 55536 | Ethyl alcohol causing toxic effect NOS |
| 56410 | Delivery of rehabilitation for alcohol addiction |
| 56441 | Michigan alcoholism screening test |
| 56947 | Continuous acute alcoholic intoxication in alcoholism |
| 57714 | Alcohol dependence with acute alcoholic intoxication |
| 57939 | Pathological alcohol intoxication |
| 59414 | [X]Intent self poison alcohol unspecif place |
| 59776 | Last six months of drinking questionnaire |
| 59873 | AUDIT - Alcohol use disorders identification test |
| 61187 | [X]Accid poison/expos to alcohol unspecif place |
| 61190 | [X]Pois/expos ?intent to alcohol unspecif place |
| 61583 | [V]Medicolegal blood alcohol test |
| 61750 | Comprehensive drinker profile |
| 62299 | SADD - Short alcohol dependence data |
| 62300 | Short alcohol dependence data |
| 63306 | [X]Accident poison/exposure to alcohol at home |
| 63457 | CDP - Comprehensive drinker profile |
| 63529 | Alcohol misuse - enhanced service completed |
| 63876 | [X]Accid poison/expos alcohol in street/highway |
| 64389 | [X]Ment & behav dis due use alcohol: unsp ment & behav dis |
| 65754 | Alcohol-induced pseudo-Cushing's syndrome |
| 65932 | [X]Alcoholic jealousy |
| 66831 | SADQ - Severity of alcohol dependence questionnaire |
| 67651 | Alcoholic psychosis NOS |
| 68111 | Other alcoholic psychosis NOS |
| 68159 | [X]Poison/exposure ?intent, to alcohol at home |



For peer review only - http://bmjopen.bmj.com/site/about/guidelines.xhtml

| | |
|---|---|
| 69331 | MAST - Michigan alcoholism screening test |
| 69407 | [X]Pois/exp ?intent alcohol school/pub admin area |
| 70939 | Munich alcoholism test |
| 84218 | Disqualified from driving due to excess alcohol |
| 90714 | Alcohol screen - AUDIT completed |
| 92908 | [X]Accid poison/expos alcohol trade/service area |
| 93415 | Alcohol units per week |
| 93624 | Fast alcohol screening test |
| 94485 | Alcohol screen - AUDIT PC completed |
| 94553 | Referral to specialist alcohol treatment service |
| 94669 | Alcohol consumption screening test declined |
| 94670 | Alcohol misuse |
| 94838 | Alcohol use disorders identification test |
| 94963 | Alcohol screen - fast alcohol screening test completed |
| 95181 | Alcohol reduction programme |
| 95650 | Advice to change drink intake |
| 95663 | Alcohol screen - AUDIT C completed |
| 95744 | Alcohol use disorder identificatn test consumptn questionnre |
| 95944 | Alcohol assesment declined - enhanced services admin |
| 96053 | Brief intervention for excessive alcohol consumptn completed |
| 96054 | Extended intervention for excessive alcohol consumptn complt |
| 96107 | Single alcohol screening questionnaire |
| 96219 | [X]Pois/expos ?intent alcohol in street/highway |
| 96259 | Alcohol assessment declined |
| 96993 | Referral to alcohol brief intervention service |
| 97085 | [X]Int self poison alcohol other spec place |
| 97126 | Alcohol units consumed on heaviest drinking day |
| 97163 | Advice to change alcoholic drink intake |
| 97163 | Advice to change alcoholic drink intake |
| 97261 | Brief intervention for excessive alcohol consumptn declined |
| 97309 | Advised to contact primary care alcohol worker |
| 97680 | Declined referral to specialist alcohol treatment service |
| 99877 | Feels should cut down drinking |
| 99985 | Brief Michigan alcoholism screening test |
| 100493 | Five-shot questionnaire on heavy drinking |
| 100989 | Breath alcohol level |
| 101426 | Accidental poisoning by ethyl alcohol NOS |
| 101543 | Other alcohol causing toxic effect |
| 101718 | Drinks in morning to get rid of hangover |
| 102121 | Police:venesect-alcohol |
| 102247 | Extended interven for excessive alcohol consumption declined |
| 102448 | Higher risk drinking |
| 102564 | Advised to abstain from alcohol consumption |
| 102577 | Severity of alcohol dependence questionnaire |

For peer review only

| | |
|---|---|
| 102665 | Increasing risk drinking |
| 102770 | SADQ - Severity of alcohol dependence questionnaire |
| 103459 | Referral to community alcohol team declined |
| 104458 | Breath ethanol level |
| 104611 | Alcohol-induced acute pancreatitis |
| 104702 | [X]Accid pois/expos alcohol in sport/athletic area |
| 104734 | [X]Accid pois/expos to alcohol other spec place |
| 108205 | Clinical Institute Withdrawal Assessment for Alcohol, revisd |
| 108644 | In-house alcohol detoxification |
| 109241 | Alcohol Use Disorders Identification Test declined |
| 109668 | Specialist alcohol treatment service signposted |
| 109675 | Hospital alcohol liaison team report received |
| 109800 | Hospital attendance related to personal alcohol consumption |
| 110494 | Alcohol harm reduction programme |
| 110624 | Alcohol relapse prevention |
| 110911 | Accidental poisoning by denatured alcohol |
| 111683 | Removal of alcohol |
| 111933 | Family wellbeing discussion about alcohol |
| 112448 | [X]Pois/expos ?intent to alcohol at res institut |
| 112645 | [X]Intent self pois alcohol in street/highway |
| 112648 | [X]Acc pois/expos alcohol indust/construct area |

For peer review only

**Supplementary Appendix 2.** Methods used for screening of "cause" fields listed on the death certificate among patients dying during index admission for ARLD.

Screening was applied to codes recorded in any "cause" field on the death certificates of in-hospital fatalities. To be flagged as a "cause-specific" death from ARLD, we required the presence of both liver and alcohol descriptor in one or more coded causes.

Hence, this included either one of K70.x codes or the appearance of a non-specific liver code in combination with at least one other alcohol-specific condition on the certificate (please refer to code lists for the LAA).

The commonest recorded K70.x code in the *first field* (cause_label) was K70.9 ALCOHOLIC LIVER DISEASE, UNSPECIFIED (accounting for 24.7% of in-hospital deaths), followed by K70.4 ALCOHOLIC HEPATIC FAILURE (20.6%), K70.3 ALCOHOLIC CIRRHOSIS OF LIVER (12.1%), K70.1 ALCOHOLIC HEPATITIS (6.0%) AND K70.0 ALCOHOLIC FATTY LIVER (0.4%). The commonest non-specific liver disease codes recorded in the *first field* (cause_label) were K74.6 OTHER AND UNSPECIFIED CIRRHOSIS OF LIVER (6.8%), K72.9 HEPATIC FAILURE, UNSPECIFIED (3%).

For peer review only

STROBE Statement—checklist of items that should be included in reports of observational studies

| | Item No | Recommendation |
|---|---|---|
| **Title and abstract** | 1 | (*a*) Indicate the study's design with a commonly used term in the title or the abstract [Done] |
| | | (*b*) Provide in the abstract an informative and balanced summary of what was done and what was found. Page 1 |
| **Introduction** | | |
| Background/rationale | 2 | Explain the scientific background and rationale for the investigation being reported. Page 3 (para 1-3) |
| Objectives | 3 | State specific objectives, including any prespecified hypotheses. Page 3 (para 4) |
| **Methods** | | |
| Study design | 4 | Present key elements of study design early in the paper. Page 3-4 |
| Setting | 5 | Describe the setting, locations, and relevant dates, including periods of recruitment, exposure, follow-up, and data collection. Page 3-4 |
| Participants | 6 | (*a*) *Cohort study*—Give the eligibility criteria, and the sources and methods of selection of participants. Describe methods of follow-up. Page 4 (para 2-3)<br>*Case-control study*—Give the eligibility criteria, and the sources and methods of case ascertainment and control selection. Give the rationale for the choice of cases and controls<br>*Cross-sectional study*—Give the eligibility criteria, and the sources and methods of selection of participants |
| | | (*b*) *Cohort study*—For matched studies, give matching criteria and number of exposed and unexposed<br>*Case-control study*—For matched studies, give matching criteria and the number of controls per case |
| Variables | 7 | Clearly define all outcomes, exposures, predictors, potential confounders, and effect modifiers. Give diagnostic criteria, if applicable Page 4-6 |
| Data sources/ measurement | 8* | For each variable of interest, give sources of data and details of methods of assessment (measurement). Describe comparability of assessment methods if there is more than one group. Page 4-5 |
| Bias | 9 | Describe any efforts to address potential sources of bias. Page 5-6 |
| Study size | 10 | Explain how the study size was arrived at. N/A all eligible cases were included |
| Quantitative variables | 11 | Explain how quantitative variables were handled in the analyses. If applicable, describe which groupings were chosen and why Page 6 |
| Statistical methods | 12 | (*a*) Describe all statistical methods, including those used to control for confounding Page 6 |
| | | (*b*) Describe any methods used to examine subgroups and interactions. Page 6 |
| | | (*c*) Explain how missing data were addressed. N/A No missing data |
| | | (*d*) *Cohort study*—If applicable, explain how loss to follow-up was addressed. N/A. Outcome was known for all cases, tracked for 365 days from admission.<br>*Case-control study*—If applicable, explain how matching of cases and controls was addressed<br>*Cross-sectional study*—If applicable, describe analytical methods taking account of sampling strategy |
| | | (*e*) Describe any sensitivity analyses. Pages 6, 10 and Supplementary Materials |

Continued on next page


For peer review only - http://bmjopen.bmj.com/site/about/guidelines.xhtml

| | | |
|---|---|---|
| **Results** | | |
| Participants | 13* | (a) Report numbers of individuals at each stage of study—eg numbers potentially eligible, examined for eligibility, confirmed eligible, included in the study, completing follow-up, and analysed. Page 7 and Table 1, Suppl. Tables |
| | | (b) Give reasons for non-participation at each stage. N/A |
| | | (c) Consider use of a flow diagram. N/A |
| Descriptive data | 14* | (a) Give characteristics of study participants (eg demographic, clinical, social) and information on exposures and potential confounders. Page 7, Table 1, Suppl. Tables |
| | | (b) Indicate number of participants with missing data for each variable of interest. N/A |
| | | (c) *Cohort study*—Summarise follow-up time (eg, average and total amount). Page 5 - all cases were followed-up for 365 days post-admission (or death). Table 2 and Figure 2 |
| Outcome data | 15* | *Cohort study*—Report numbers of outcome events or summary measures over time. Page 9-10, Table 1, Figure 2, Suppl. Fig. 1, Suppl. Figs S2-S4 |
| | | *Case-control study*—Report numbers in each exposure category, or summary measures of exposure |
| | | *Cross-sectional study*—Report numbers of outcome events or summary measures |
| Main results | 16 | (*a*) Give unadjusted estimates and, if applicable, confounder-adjusted estimates and their precision (eg, 95% confidence interval). Make clear which confounders were adjusted for and why they were included. Page 10, Table 2 and Suppl. Figures |
| | | (*b*) Report category boundaries when continuous variables were categorized |
| | | (*c*) If relevant, consider translating estimates of relative risk into absolute risk for a meaningful time period. N/A |
| Other analyses | 17 | Report other analyses done—eg analyses of subgroups and interactions, and sensitivity analyses. Page 10 and Supplementary Materials |
| **Discussion** | | |
| Key results | 18 | Summarise key results with reference to study objectives. Page 11 |
| Limitations | 19 | Discuss limitations of the study, taking into account sources of potential bias or imprecision. Discuss both direction and magnitude of any potential bias. Page 12-13 |
| Interpretation | 20 | Give a cautious overall interpretation of results considering objectives, limitations, multiplicity of analyses, results from similar studies, and other relevant evidence. Page 11-12 |
| Generalisability | 21 | Discuss the generalisability (external validity) of the study results. Page 13, para 2 |
| **Other information** | | |
| Funding | 22 | Give the source of funding and the role of the funders for the present study and, if applicable, for the original study on which the present article is based. Page 13 |

*Give information separately for cases and controls in case-control studies and, if applicable, for exposed and unexposed groups in cohort and cross-sectional studies.

**Note:** An Explanation and Elaboration article discusses each checklist item and gives methodological background and published examples of transparent reporting. The STROBE checklist is best used in conjunction with this article (freely available on the Web sites of PLoS Medicine at http://www.plosmedicine.org/, Annals of Internal Medicine at http://www.annals.org/, and Epidemiology at http://www.epidem.com/). Information on the STROBE Initiative is available at www.strobe-statement.org.


For peer review only - http://bmjopen.bmj.com/site/about/guidelines.xhtml
